# Adipose tissue-derived human mesenchymal stromal cells can better suppress complement lysis, engraft and inhibit acute graft-versus-host disease in mice

**DOI:** 10.1186/s13287-023-03380-x

**Published:** 2023-06-25

**Authors:** Stanley Chun Ming Wu, Manyu Zhu, Stanley C. C. Chik, Maxwell Kwok, Asif Javed, Laalaa Law, Shing Chan, Kenneth R. Boheler, Yin Ping Liu, Godfrey Chi Fung Chan, Ellen Ngar-Yun Poon

**Affiliations:** 1 Department of Paediatrics and Adolescent Medicine, LKS Faculty of Medicine, The University of Hong Kong, Pokfulam, Hong Kong SAR China; 2 Department of Biomedical Engineering, The Johns Hopkins University, Baltimore, MD 21205 USA; 3 Department of Orthopaedics and Traumatology, LKS Faculty of Medicine, The University of Hong Kong, Pokfulam, Hong Kong SAR China; 4 Department of Pathology, The Johns Hopkins University, Baltimore, MD 21205 USA; 5 Department of Medicine and Therapeutics, The Chinese University of Hong Kong, Shatin, Hong Kong SAR China; 6 Hong Kong Hub of Paediatric Excellence (HK HOPE), The Chinese University of Hong Kong, Kowloon Bay, Hong Kong SAR China; 7 School of Biomedical Science, LKS Faculty of Medicine, The University of Hong Kong, Pokfulam, Hong Kong SAR China; 8 Division of Cardiology, Department of Medicine and Department of Biomedical Engineering, The Johns Hopkins University, Baltimore, MD 21205 USA; 9 Doctors’ Office, 9/F, Tower B, Hong Kong Children’s Hospital, 1 Shing Cheong Road, Kowloon Bay, Hong Kong SAR China; 10 The School of Biomedical Sciences, The Chinese University of Hong Kong, Rm 226A, 2/F, Lo Kwee-Seong Integrated Biomedical Sciences Building, Area 39, Shatin, Hong Kong SAR China; 11 Centre for Cardiovascular Genomics and Medicine, Lui Che Woo Institute of Innovative Medicine, The Chinese University of Hong Kong, Shatin, Hong Kong SAR China

**Keywords:** Human mesenchymal stromal cells, Acute graft-versus-host disease, CD55, Complement, hMSC transplantation, hMSC

## Abstract

**Background:**

Acute graft-versus-host disease (aGvHD) is a life-threatening complication of allogeneic hematopoietic stem cell transplantation (HSCT). Transplantation of immunosuppressive human mesenchymal stromal cells (hMSCs) can protect against aGvHD post-HSCT; however, their efficacy is limited by poor engraftment and survival. Moreover, infused MSCs can be damaged by activated complement, yet strategies to minimise complement injury of hMSCs and improve their survival are limited.

**Methods:**

Human MSCs were derived from bone marrow (BM), adipose tissue (AT) and umbilical cord (UC). In vitro immunomodulatory potential was determined by co-culture experiments between hMSCs and immune cells implicated in aGvHD disease progression. BM-, AT- and UC-hMSCs were tested for their abilities to protect aGvHD in a mouse model of this disease. Survival and clinical symptoms were monitored, and target tissues of aGvHD were examined by histopathology and qPCR. Transplanted cell survival was evaluated by cell tracing and by qPCR. The transcriptome of BM-, AT- and UC-hMSCs was profiled by RNA-sequencing. Focused experiments were performed to compare the expression of complement inhibitors and the abilities of hMSCs to resist complement lysis.

**Results:**

Human MSCs derived from three tissues divergently protected against aGvHD in vivo. AT-hMSCs preferentially suppressed complement in vitro and in vivo, resisted complement lysis and survived better after transplantation when compared to BM- and UC-hMSCs. AT-hMSCs also prolonged survival and improved the symptoms and pathological features of aGvHD. We found that complement-decay accelerating factor (CD55), an inhibitor of complement, is elevated in AT-hMSCs and contributed to reduced complement activation. We further report that atorvastatin and erlotinib could upregulate CD55 and suppress complement in all three types of hMSCs.

**Conclusion:**

CD55, by suppressing complement, contributes to the improved protection of AT-hMSCs against aGvHD. The use of AT-hMSCs or the upregulation of CD55 by small molecules thus represents promising new strategies to promote hMSC survival to improve the efficacy of transplantation therapy. As complement injury is a barrier to all types of hMSC therapy, our findings are of broad significance to enhance the use of hMSCs for the treatment of a wide range of disorders.

**Supplementary Information:**

The online version contains supplementary material available at 10.1186/s13287-023-03380-x.

## Introduction

Acute (a) graft-versus-host disease (GvHD), aGvHD, is a life-threatening complication that can arise after transplantation of allogeneic hematopoietic stem cells. Up to 38% of patients who receive matched, unrelated allogeneic hematopoietic stem cells transplantation can develop aGvHD [1, 2], of which only 50–65% of patients will respond to conventional steroid therapy [3, 4]. The two-year survival rate of patients with severe or steroid-resistant aGvHD is as low as 3–17% [4–7]. Ruxolitinib, a JAK1/JAK2 inhibitor, has recently been approved for the treatment of steroid-resistant GvHD, but a significant number of patients still suffer from the consequence of GvHD [8].

Human (h) multipotent mesenchymal stromal/stem cells (MSCs) are potent suppressors of the immune system [9–12] and are of clinical value for cell therapy against immune disorders. Eight hundred and fifty-seven registered clinical trials have been designed to explore the clinical efficacy of hMSC-based cell therapy [13], of which 52 are/were against aGvHD (data from clinicaltrials.gov through 2021). Early studies on aGvHD showed that hMSC therapy was well tolerated [13]. Thirty out of fifty-five patients achieved complete response, which is defined as the resolution of symptoms of aGvHD [13]. Furthermore, these patients had lower transplantation-related mortality compared to those with partial or no response [13]. Overall, results from clinical studies have indicated favourable outcomes and negligible side effects when hMSCs are used to treat aGvHD [7, 13–16].

The effects of hMSCs on the immune system are often transient [17, 18]. Although hMSCs are commonly considered to be immunoprivileged and can escape from host immune surveillance [9, 10], transplanted hMSCs rarely engraft long-term and only survive for a short period after injection [17, 18]. The mechanisms that inhibit hMSC engraftment are unclear; however, some have attributed this to the triggering of multifaceted adverse innate immune responses, termed instant blood-mediated inflammatory reaction (IBMIR) [19, 20]. Infused hMSCs can be recognised and damaged by the complement system as part of the innate immunity response, which likely reduce the therapeutic efficacy of hMSC transplantation [21, 22]. It is also noteworthy that cryopreserved MSCs in particular may be more vulnerable to damage than fresh cells and are prone to triggering the IBMIR and complement activation [23–26]. Thus, poor survival and inappropriate complement activation are known barriers to hMSC therapy [27, 28].

Human MSCs were traditionally isolated from BM but can also be derived from adipose tissue (AT) and umbilical cord (UC). AT- and UC-hMSCs can suppress many components of the immune system in vitro and may be good alternatives to BM-hMSCs [29–34]. To date, few studies have directly compared the effectiveness of hMSCs from different sources using in vivo models of aGvHD, and none has compared the engraftment and survival of BM-, AT-, and UC-hMSCs after transplantation. A recent transcriptomic profiling study indicates that AT-hMSCs express higher levels of complement regulators compared to BM-hMSCs, but experimental proof is lacking and the functional implications are unclear [35]. Another report also demonstrated differential expression of some complement regulators between hMSCs derived from BM and placental decidua, but AT-hMSCs were not included [34]. We hypothesise that hMSCs that naturally present inhibitors of complement are less susceptible to damage by complement lysis. We further posited that this decrease in susceptibility would improve their transplantation survival and prove more effective against aGvHD in vivo. In this study, we demonstrate that hMSCs protected against aGvHD in a mouse model of this disease; however, AT-hMSCs better ameliorated symptoms and prolonged survival when compared to BM and UC-hMSCs. We attribute this improvement to superior engraftment and survival. Mechanistically, AT-hMSCs expressed higher levels of CD55, an inhibitor of the complement pathway, and this was associated with greater suppression of complement in vitro and in vivo. We further identified two compounds that could upregulate CD55 in hMSCs and improve hMSC protection against complement-induced injury.

## Methods

### Derivation, culture and characterisation of hMSCs

Human MSCs were derived from fresh samples of BM, AT and UC using established methods with some modifications [20, 29, 36]. Human MSCs were characterised based on standards proposed by the Mesenchymal and Tissue Stem Cell Committee of the International Society for Cellular Therapy (ISCT) [37]. The isolation procedures were approved by the local ethics committee (Combined Internal Review Board of The University of Hong Kong and The Hong Kong West Cluster of Hospital of Hospital Authority, # UW 13-429).

BM-hMSCs were isolated from healthy human bone marrow transplantation donors by density-gradient centrifugation with Ficoll-Hypaque solution (Amersham Biosciences). Subcutaneous AT was obtained from patients undergoing planned Caesarean sections and digested with collagenase, followed by the lysis of red cells using Red Blood Cell Lysis Solution (Miltenyi Biotec) as per manufacturer’s instructions. Umbilical cord was collected, and the cord vein was washed out. The vessel was digested with collagenase. After the initial isolation step, all hMSCs were plated and cultured using established methods in Dulbecco's modified Eagle's medium with low glucose, supplemented with 10% human platelet lysate [29, 38]. hMSCs were all incubated at 37 °C in a humidified incubator with 5% CO_2_. The medium was changed every two days. Human MSCs between passages 2–6 were used for all experiments, and after at least one passage post-thawing unless otherwise stated.

### In vitro assays

The interaction between hMSCs and dendritic, T- and natural killer cells was assessed using co-culture experiments followed by ELISA of relevant cytokines [9]. The effect of hMSCs on the proportion of T-regulatory (Treg) was measured by flow analysis [39].

### In vivo assays

**Fig. 1:**
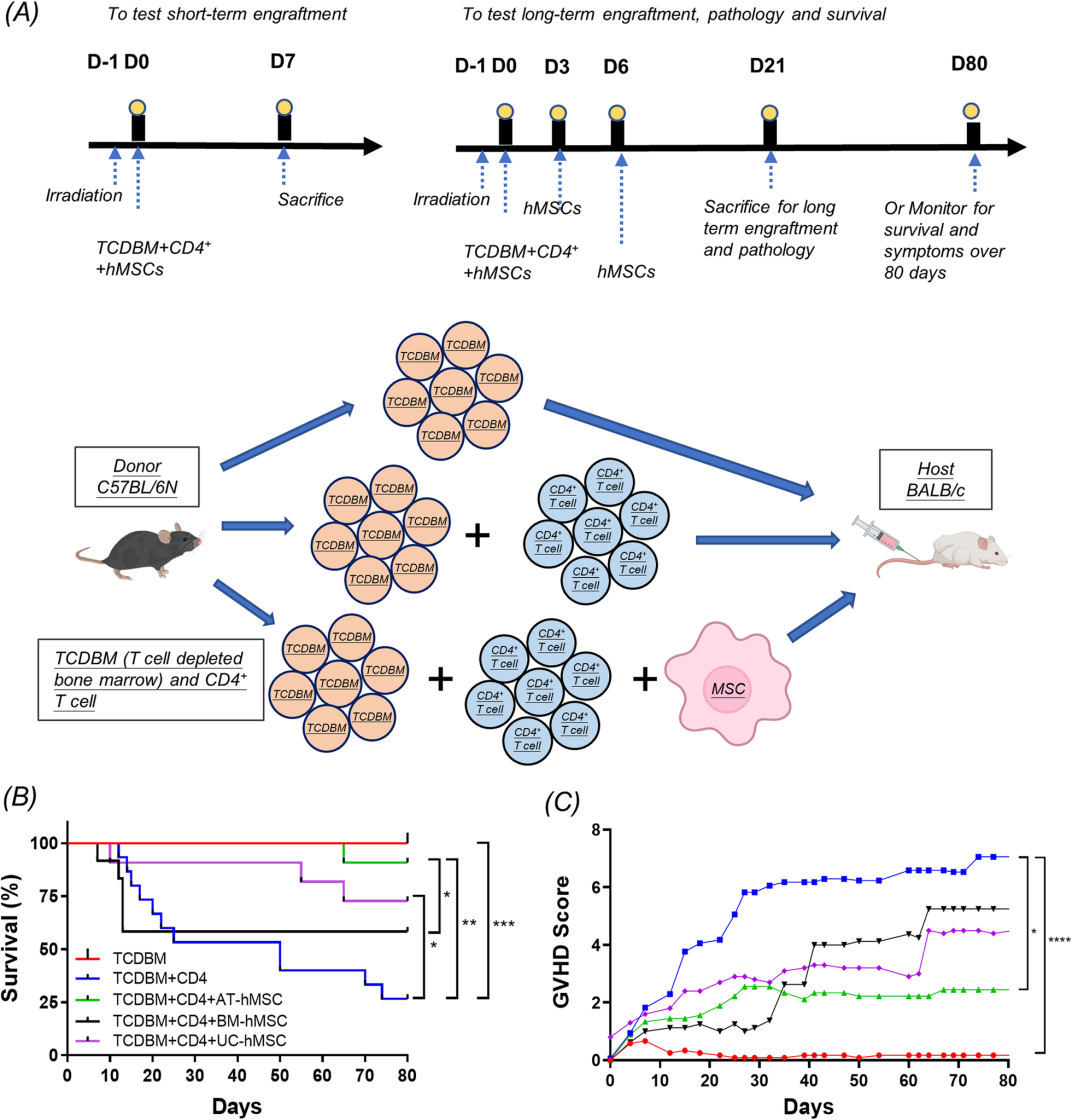
AT-hMSCs conferred superior protection against aGvHD in vivo. **A** Schematic of aGvHD mouse model. BALB/c host mice were exposed to γ-irradiation at 800 cGy around 24 h prior to treatment with T cell-depleted bone marrow (TCDBM) with/without CD4^+^ T cell splenocytes (CD4^+^), with/without hMSCs. Negative control group: TCDBM from C57BL/6N. Positive control group: TCDBM and CD4^+^ from C57BL/6N to induce a graft-versus-host reaction. Human MSCs treatment groups were injected with TCDBM and CD4^+^ T cells with hMSCs. For the evaluation of short-term engraftment, mice were given one hMSC injection on day 0 and killed on day 7. To monitor pathology and long-term engraftment, mice were given 3 doses of hMSCs on days 0, 3, 6 and harvested on day 21. Similarly, 3 injections of hMSCs were administered for the assessment of survival and symptoms, and mice were monitored for 80 days. **B** Survival was monitored over 80 days by Kaplan–Meier analysis. **C** GvHD clinical scores, significance shown against positive control. *n* = 12, 17, 9, 10, 8 for TCDBM, TCDBM + CD4, TCDBM + CD4 + AT-hMSCs, TCDBM + CD4 + UC-hMSCs, TCDBM + CD4 + BM-hMSCs. *p < 0.05, **p < 0.01, ***p < 0.001, ****p < 0.0001

All experimental procedures on animals were performed according to Committee on the Use of Live Animals in Teaching and Research of the University of Hong Kong (38-9316). BALB/C host mice (5–6 weeks) were exposed to lethal γ-irradiation (800 cGy) 24 h before injection. 2 × 10^6^ T cell-depleted bone marrow (TCDBM) and 0.25 × 10^6^ CD4^+^ T cells of C57BL/6N donor mice (5–6 weeks) were injected via the tail vein into BALB/C mice to induce aGvHD [40]. Mice were randomised into five groups. The negative control group consisted of mice injected with TCDBM only, and the positive control was mice injected with TCDBM and CD4^+^ T cells. To evaluate short-term engraftment on day 7, three hMSC treatment groups were injected with TCDBM and CD4^+^ T cells, with 1 × 10^6^ of each type of hMSCs on day 0 and were killed on day 7. To monitor long-term engraftment and survival, three hMSCs treatment groups were injected with TCDBM and CD4^+^ T cells with 1 × 10^6^ hMSCs on day 0, and followed by additional hMSC injections on days 3 and 6 as described in Fig. 1A. Host mice were killed using an overdose of pentobarbital on day 7, 21 and 80 for analysis. Survival was monitored for 80 days by Kaplan–Meier analysis. The mice were examined every two days to monitor signs of aGvHD according to an established clinical scoring system [41]. Human MSC engraftment was monitored by staining the cells with CM-Dil dye, followed by fluorescence imaging of harvested tissues.

### CD55 and complement assays

Transcriptomic profiling of hMSCs was performed by RNA sequencing, followed by gene ontology analysis. The expression of CD55 was assessed by RT-qPCR and flow cytometry. Complement response and membrane leakage were evaluated by C3 deposition [21] and the BCECF dye, respectively [21].

### Data processing and analysis

Experimental parameters from the different groups of hMSCs were expressed as mean ± standard error of the mean (SEM) and compared using one-way ANOVA test. In the absence of normal distribution, the significance between the groups was evaluated with nonparametric Kruskal–Wallis test. Data were analysed using GraphPad Prism software (GraphPad Inc., San Diego, CA, USA), and *p* < 0.05 was considered the threshold of significance.

See Additional file 1: Supplementary methods for additional experimental details. Lists of antibodies and primer sequences are presented in Additional files 2 and 3: Tables S1 and S2, respectively.

## Results

### Human MSCs derived from AT, BM and UC

Human MSCs derived from AT, BM and UC were characterised according to international standards (ISCT) [37]. Specifically, AT-, BM- and UC-hMSCs adhered to plastic and were positive for CD73, CD90, CD105, and human leukocyte antigen (HLA)-DR, but negative for CD14, CD19, CD34, CD45, CD19, or HLA-DR (Additional file 4: Fig. S1A) [37]. AT-, BM- and UC-hMSCs could all undergo tri-lineage differentiation into fat, bone and cartilage when cultured in adipogenic, osteogenic and chondrogenic conditions (Additional file 4: Fig. S1B).

### AT-hMSCs alleviated clinical symptoms and promoted survival in a mouse model of aGvHD better than BM- and UC-hMSCs

We tested the effectiveness of AT-, UC- and BM-hMSCs against aGvHD in vivo using an established mouse model of this disease [40]. Graft-versus-host reaction was induced by co-transplanting T cell-depleted bone marrow (TCDBM) and T cells from the spleen of C57BL/6N donor mice into irradiated BALB/C host mice. For the evaluation of short-term engraftment, mice were given one hMSC injection (1 × 10^6^) on day 0 along with TCDBM and T cells and killed on day 7. To assess long-term effects, mice were given 3 doses of hMSCs on day 0 (with TCDBM and T cells), 3, 6 and harvested on day 21 to monitor pathology and long-term engraftment or were monitored for 80 days for symptoms and survival (Fig. 1A). The effect of AT-, BM- and UC-hMSCs on survival was monitored for 80 days by Kaplan–Meier analysis (Fig. 1B). One hundred percentage of the negative control group, comprising host mice that received donor TCDBM and simulating the disease-free state, survived for 80 days. By contrast, the survival of the positive control group given TCDBM and CD4^+^ splenocytes, simulating untreated aGvHD, was significantly reduced to 27%. All three groups of mice given hMSCs fared better than the positive control. AT-hMSCs transplantation conferred markedly improved survival (91%) at day 80 compared to UC-hMSCs (73%) and BM-hMSCs (58%) (Fig. 1B).

Resolution of symptoms is used as endpoints of clinical studies of aGvHD. To test whether prolonged survival following AT-hMSC transplantation was associated with amelioration of symptoms, we scored the mouse cohorts using established criteria encompassing common aGvHD symptoms including weight loss, posture, activity, fur texture and skin integrity (0 = least severe, 10 = most severe, Additional file 5: Table S3) [40, 41]. Mice injected with both TCDBM and CD4^+^ cells had the highest score (score: 7.1 ± 0.7), indicative of severe disease (Fig. 1C), while mice injected with TCDBM were mostly free of symptoms (score: 0.2 ± 0.2). Mice injected with AT-hMSCs had a significantly lower score (score: 2.4 ± 1.1) compared to positive control, and this was qualitatively lower compared to UC-hMSCs (4.5 ± 1.0) and BM-hMSCs (score: 5.3 ± 1.4), indicating that AT-hMSCs preferentially suppressed symptoms of aGvHD. This is associated with higher body weight in mice transplanted with AT-hMSCs (Additional file 6: Fig. S2A). Representative images of animals are shown in Additional file 6: Fig. S2B.

**Fig. 2:**
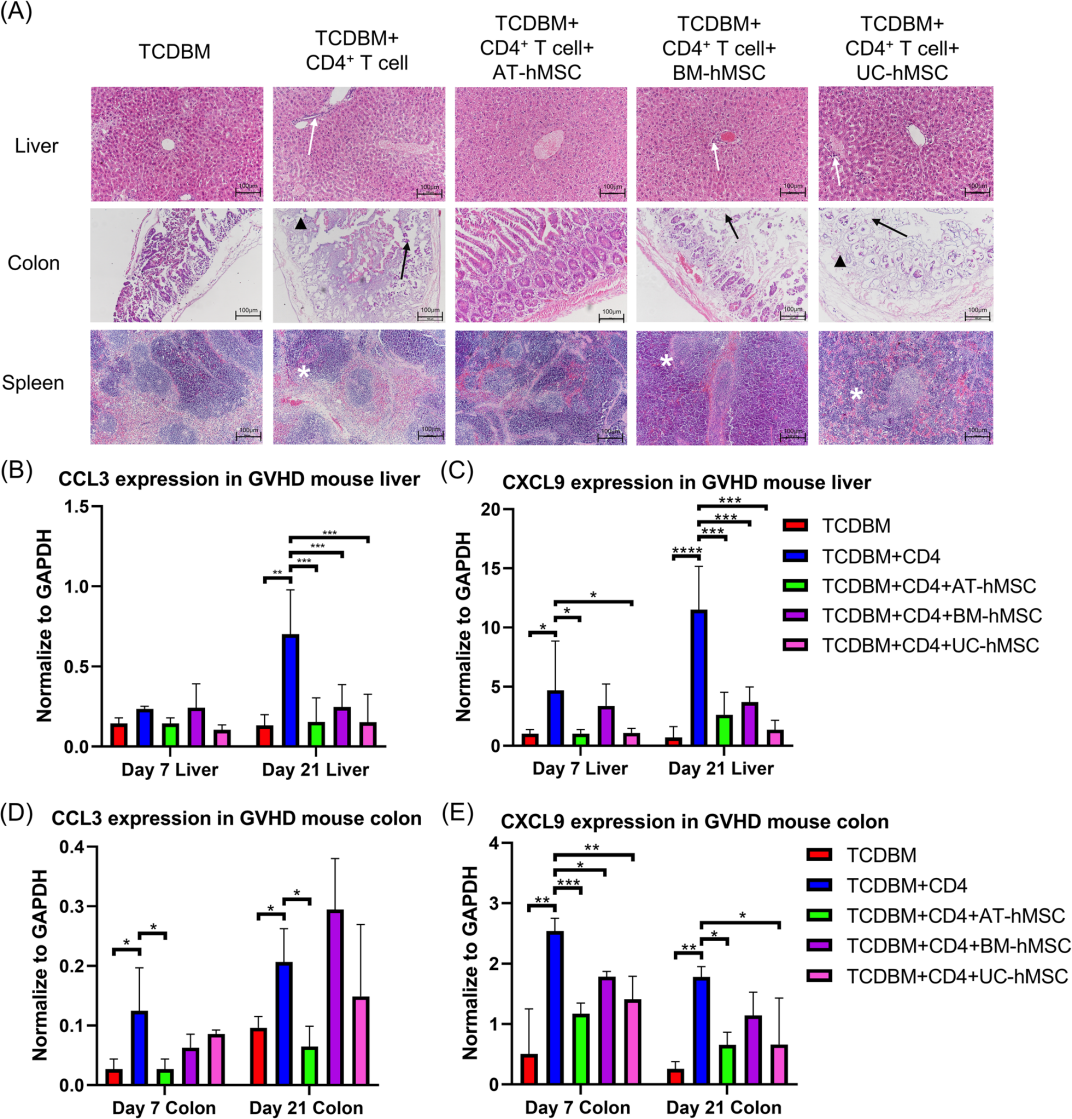
AT-hMSCs better protected target organs of aGvHD. **A** Representative photographs of H&E-stained liver, colon and spleen tissues on day 21. Scale bar = 100 μm. White arrows = apoptotic bodies, black arrows = crypt loss, arrowhead = atrophy of surface epithelium, * = loss of white pulp. **B**, **D** CCL3 and **C**, **E** CXCL9 mRNA expression in mouse liver **B**, **C** and colon **D**, **E** on days 7 and day 21 post-transplantation. Graphs show data from three experiments with mean + / − SEM. Significance was calculated relative to positive control group (TCDBM + CD4) *p < 0.05, **p < 0.01, ***p < 0.001, ****p < 0.0001

Pathologically, aGvHD mainly affects the skin, liver, and gastrointestinal tract. By histopathological examinations, we determined which hMSCs could best protect against tissue damage (Fig. 2A). Consistent with the clinical features of aGvHD, the colon, liver, and spleen were the most affected organs 21 days after transplantation. Colon samples from the positive control group showed severe damage, with widespread crypt destruction and dense lamina propria lymphocytic infiltration. Samples from BM-hMSCs and UC-hMSCs groups had similar pathology with the positive control, showing that BM- and UC-hMSCs conferred limited protection in the colon. By contrast, the AT-hMSC cohort had normal colon structures, indicating that these hMSCs could better suppress colon tissue destruction (Fig. 2A). Similarly, AT-hMSCs could better preserve the tissue structures in the liver and the spleen compared to BM- and UC-hMSCs.

Chemokines such as CCL3 and CXCL9 are strong inducers of leukocyte trafficking and activation, and they contribute to the pathogenesis of aGvHD. All hMSCs significantly suppressed CCL3 and CXCL9 RNA expression in the liver on day 21 compared to the positive control, and similar results were seen on day 7 with varying significance (Fig. 2B and C). In the colon, marked differences were observed among the hMSC groups. Only AT-hMSCs could significantly reduce CCL3 and CXCL9 expression on both days 7 and 21 (Fig. 2D and E). These results are consistent with AT-hMSCs having a higher capacity to modulate host chemokine expression compared to BM- and UC-hMSCs and thereby inhibit the migration of T cell lymphocytes and the development of aGvHD.

### AT-, BM- UC-hMSCs exhibited similar immunosuppressive properties in vitro

Our results thus far show that AT-hMSCs preferentially promote survival and better suppress the symptoms and pathological features of aGvHD in vivo. To explain the differences in therapeutic efficacy among hMSCs, we postulate that AT-hMSCs might have superior intrinsic immunomodulatory capabilities and/or that they could better engraft and survive compared to BM- and UC-hMSCs.

**Fig. 3:**
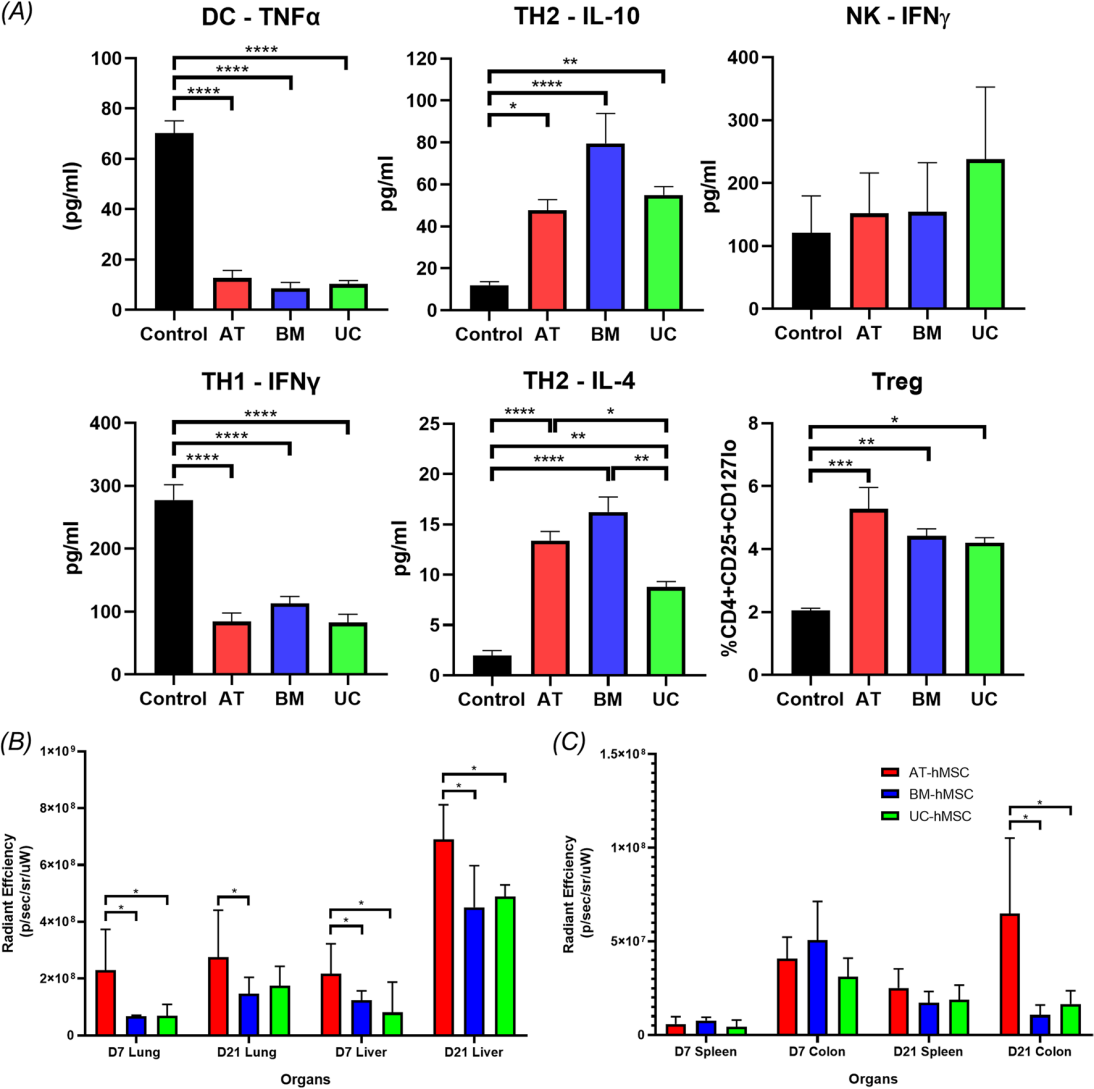
Immunosuppressive effect of hMSCs in vitro and survival of hMSC in vivo. **A** The effect of DC-mediated TNFα secretion was measured by ELISA assay. The effect of hMSCs on TH1 and Th2 was determined by measuring the release of IFNγ (TH1) and, IL-4 and IL-10 (Th2) by ELISA. NK-mediated IFNγ secretion was assessed by ELISA. The proportion of Treg was examined by flow cytometry. *n* = 5. Quantitative analysis of the fluorescently labelled hMSCs in the **B** lung and liver, **C** spleen and colon on days 7 and 21 post-transplantation. Graphs show data of three experiments with mean + / − SEM.*p < 0.05, **p < 0.01, ***p < 0.001, ****p < 0.0001

We tested the immunomodulatory properties of these hMSCs using in vitro co-culture experiments (Fig. 3A). aGvHD involves the activation of antigen-presenting cells such as dendritic cells, and T cells. To assess the immunomodulatory capabilities of hMSCs, we measured the secretion of pro-inflammatory cytokine TNF-α. BM-, UC- and AT-hMSCs significantly and similarly repressed TNF-α secretion relative to dendritic cells cultured alone. The effect of hMSCs on T cell differentiation into T-helper-1 (Th-1) and -2 (Th-2) cells was assessed by measuring the release of effector cytokines (pro-inflammatory IFN-γ for Th-1 and anti-inflammatory IL-4 and IL-10 for Th-2). All hMSCs significantly suppressed IFN-γ secretion and stimulated IL-4 and IL-10 secretion relative to PBMCs. No significant differences were detected among the three groups of hMSCs, except in IL-4 secretion, in which UC-hMSCs had a lower stimulatory effect than AT- and BM-hMSCs. We also assessed the effect of hMSCs on natural killer cells. None of the hMSCs significantly altered natural killer cell-mediated IFN-γ secretion. Natural killer cells are important for mediating the anti-tumour response in transplant patients; thus, the lack of suppression may be beneficial. Lastly, we examined by flow analysis the effect of hMSCs on Treg cells, which can ameliorate aGvHD progression. All three types of hMSCs significantly and similarly increased the proportion of Treg cells compared to control. These co-culture experiments demonstrate that all three types of hMSCs can significantly, and similarly, attenuate multiple components of the immune system in vitro.

### AT-hMSCs engrafted/survived better in host mice compared to BM- and UC-hMSCs

We then determined whether AT-hMSCs could better engraft compared to BM- and UC-hMSCs. We tracked transplanted hMSCs by staining them with a fluorescence dye (CM-Dil) and quantified the presence of hMSCs by monitoring the intensity of fluorescence signal in host tissues 21 days after transplantation. Mice given TCDBM alone or TCDBM with CD4^+^ cells were used to control for background fluorescence intensity. Human MSCs were localised in the spleen, colon, stomach, kidney, liver and lung of host mice, with the majority in the liver and lung (Fig. 3B and C and Additional file 7: Fig. S3A), consistent with previous literature showing the retention of hMSCs in these two organs [42]. Injection of AT-hMSCs resulted in the strongest fluorescence signal, which was 1.53-, 1.87- and 7.30-fold significantly higher in the liver, lung, and colon than that of BM-hMSCs, respectively. Transplantation of UC-hMSCs gave intermediate fluorescence intensity. Similar results were seen at day 7 of transplantation (Fig. 3B and C and Additional file 7: Fig. S3A). RT-qPCR also revealed significantly higher levels of *human* GAPDH in the liver and colon of mice given AT-hMSCs (Additional file 7: Fig. S3B), confirming better retention of transplanted *human* AT-hMSCs in host mouse tissues.

### Transcriptomic profiling revealed higher level of CD55 in AT-hMSCs

**Fig. 4:**
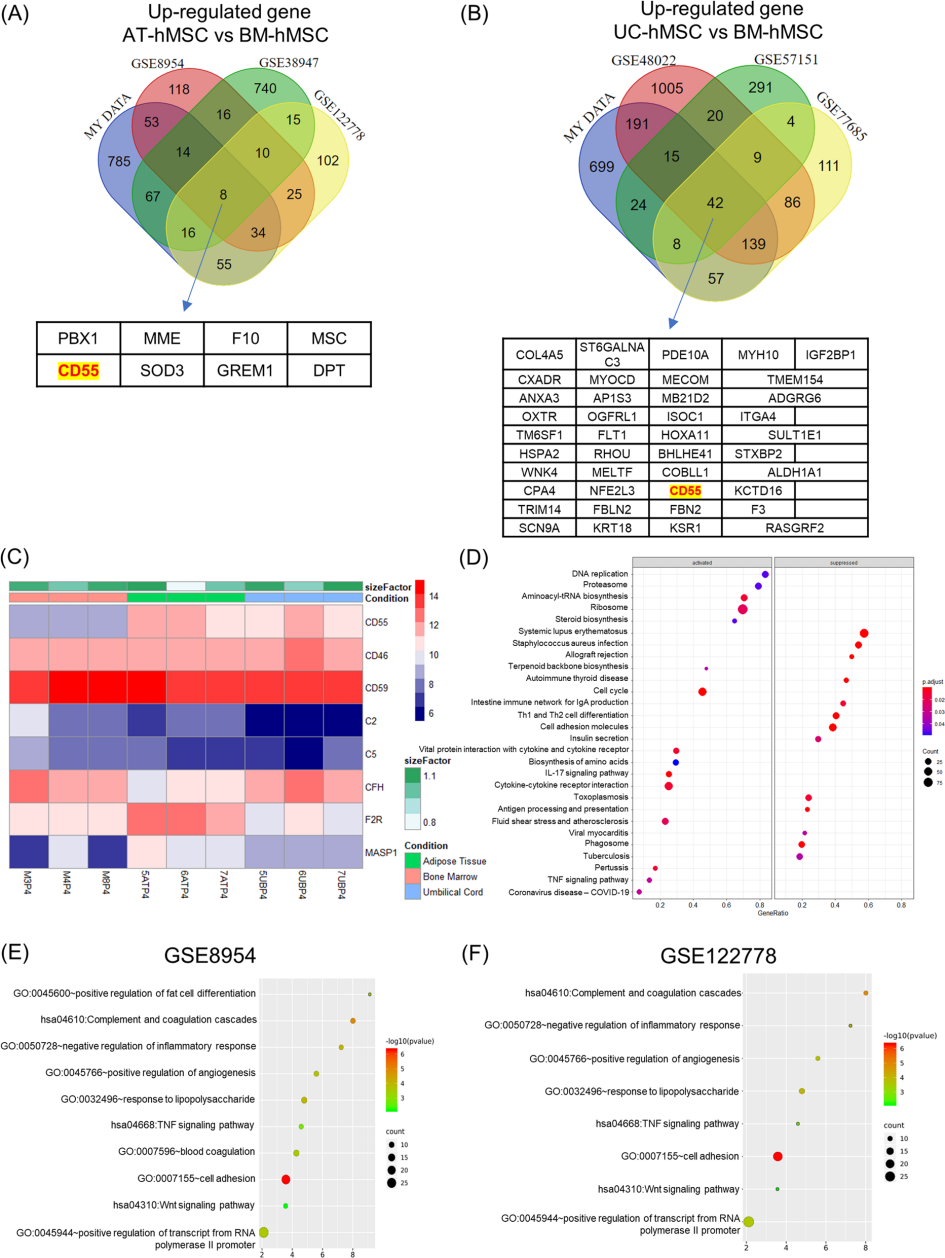
Transcriptomic analysis revealed differentiation expression of CD55 and complement genes. Venn diagram of DEGs of 3 public datasets with our dataset for the comparison between **A** AT-hMSCs versus BM-hMSCs and **B** UC-hMSCs versus BM-hMSCs. Upregulated genes are defined as log fold change > 1, *p* < 0.05. **C** Heatmap expression of genes involved in the complement pathway in our dataset. **D** GSEA analysis of AT-hMSCs versus BM-hMSCs in our dataset. **E** and **F** GO and KEGG analyses of genes upregulated in AT-hMSCs versus BM-hMSCs from two public datasets

To understand the molecular mechanisms that underlie the abilities of AT-hMSCs to engraft and survive, we examined the global transcriptome of hMSCs by RNA-seq. Principal component analysis and hierarchical clustering showed that AT-, BM- and UC-hMSCs were associated with distinct transcriptomic profiles (Additional file 8: Fig. S4A, B). Comparison between AT- versus BM-, AT- versus UC- and UC- versus BM-hMSCs revealed 2318 (7%), 2886 (9%) and 2270 (7%) differentially expressed genes (> twofold) (Additional file 8: Fig. S4C, Additional files 9, 10 and 11: Tables S4, S5 and S6). GSEA analyses showed an upregulation of genes involved in cell growth (e.g. DNA replication, cell cycle, ribosome) and a downregulation of genes implicated in immune disorders (e.g. systemic lupus erythematosus, allograft rejection) in AT-hMSCs versus BM-hMSCs (Fig. 4D).

**Fig. 5:**
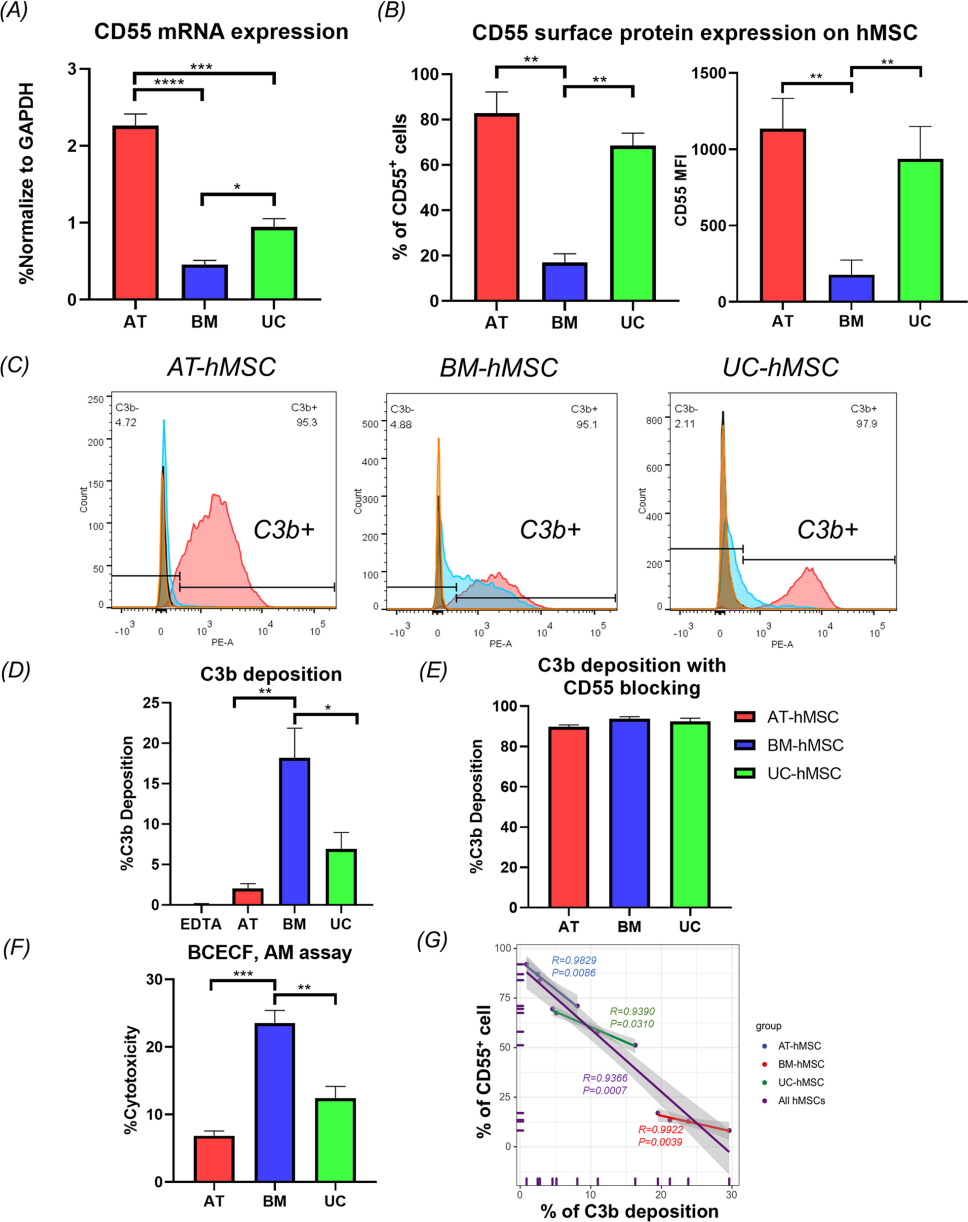
AT-hMSCs expressed higher levels of CD55 and were less prone to complement injury. CD55 **A** mRNA and **B** protein expression by qPCR and flow cytometry analyses. **C** Flow cytometry analysis of C3 deposition on hMSCs after exposure to serum. Orange area indicates the hMSCs without serum (Control). Black area indicates hMSCs treated with serum and EDTA control. Blue area indicates hMSCs treated with serum. Red area indicated hMSCs treated with mouse serum, following pre-incubation with anti-CD55 antibody. Results are summarised in two graphs showing the **D** mean %C3 deposition (%hMSCs positive for C3) **E** mean %C3 deposition following pre-incubation (blocking) with anti-CD55 antibody. **F** Cytotoxicity assay to demonstrate membrane leakage after exposure to serum, assessed by BCECF, AM dye. The graph shows the % cytotoxicity, measured as % BCECF dye that was released into the supernatant **G** Linear regression plot of %C3 deposition and %CD55 + cells in hMSCs. Bar graphs show the summary of three independent experiments with mean +/− SEM. *p < 0.05, **p < 0.01, ***p < 0.001

We next examined our dataset together with three publicly available datasets involving AT-, UC- versus BM-hMSCs to identify differences among these cells that are conserved across multiple studies [35, 43–47]. Venn diagrams revealed that 8 and 42 transcripts were upregulated in AT- and UC-hMSCs compared to BM-hMSCs in all four datasets (Fig. 4A–C). The complement decay-accelerating factor CD55 was the only gene upregulated in both AT-hMSCs and UC-hMSCs compared to BM-hMSCs. Consistent with the role of CD55 as a suppressor of complement activation [48], KEGG pathway analysis revealed complement genes to be among the top differentially expressed transcripts between AT and BM-hMSCs (Fig. 4D–F). Experimentally, CD55 mRNA could be detected by RT-qPCR in all three types of hMSCs, but was significantly more abundant in AT-hMSCs than UC-hMSCs (FC = 2.4) and BM-hMSCs (FC = 4.9) (Fig. 5A). Flow cytometry analysis further revealed that a greater percentage of AT-hMSCs (83.42 ± 9.57%) and UC-MSC (63.4 ± 7.87%) expressed CD55 on the cell surface, and at higher levels, than BM-hMSCs (14.46 ± 4.63%) (Fig. 5B). Conversely, other complement regulatory genes such as CD59 were expressed at similar levels among BM-, AT- and UC-hMSCs [34] (Fig. 4C and Additional file 12: Fig. S5). We also evaluated the level of CD142, which is a mediator of the coagulation pathway. By qPCR analysis, significantly higher mRNA levels of CD142 were observed in AT-BMCs relative to BM-hMSCs. Based on flow cytometry analyses, almost all the AT-, BM-, and UC-hMSCs (> 93%) expressed this protein, but CD142 was present at qualitatively higher levels in AT-hMSCs and UC-hMSCs than in BM-hMSCs (Additional file 12: Fig. S5).

### AT-hMSCs were less susceptible to complement-mediated injury than BM-hMSCs and UC-hMSCs

Previous studies have shown that MSCs are prone to complement lysis upon contact with serum [10, 21, 22, 48, 49] and that CD55 can protect against complement injury. To test the supposition that increased abundance of CD55 on AT-hMSCs would inhibit complement-induced damage, we incubated BM-, AT- and UC-hMSCs with serum and found significantly reduced deposition of the complement activation product C3b on the cell surface of AT-hMSCs (3.57 ± 0.58%) relative to UC-hMSCs (9.26 ± 4.69%) and BM-hMSCs (23.53 ± 3.75%) (Fig. 5C and D). EDTA-inactivated serum was used as a negative control to show that complement activation and deposition of the C3b was dependent on complement activity (Ca^2+^/Mg^2+^ dependent) (Fig. 5C and D). Blocking CD55 by pre-incubating hMSCs with anti-CD55 antibody abolished these differences and significantly increased C3b deposition on all three hMSCs to > 90% (Fig. 5E), implicating a critical role of CD55 in determining complement activation in hMSCs.

Since AT-hMSCs had reduced levels of C3b, which is crucial for the formation of the membrane attack complex, they are expected to be less susceptible to complement lysis. We tested this with the membrane-permeant BCECF ester. Upon intracellular hydrolysis, the dye becomes impermeant and trapped in the cell; thus, its release is used to monitor cell membrane rupture. We loaded hMSCs with BCECF ester, exposed the cells to serum and monitored the release of this dye. Significantly less dye (i.e. reduced membrane leakage) was detected in the supernatant of AT-hMSC cultures (6.85 ± 1.02%) compared to BM-hMSCs (23.53 ± 2.67%) and UC-hMSCs (12.44 ± 2.42%) (Fig. 5F).

Cryopreservation has been shown to impact complement activation. We therefore evaluated the effect of freeze-thawing on the presence of CD55 and deposition of CD3. In these experiments, we utilised cells in culture that had been passaged at least once after thawing (fresh) or which were used immediately upon thawing (frozen). By flow cytometry, we observed a significant, but modest, decrease in the percentage of CD55^+^ cells among frozen AT-hMSCs when compared to fresh AT-hMSCs. Frozen AT-hMSCs also had qualitatively higher C3b deposition after incubation with serum than fresh cells (Additional file 12: Fig. S5). Culturing of hMSCs thus appears to provide better protection against complement than hMSCs that are utilised immediately upon thawing.

We further investigated the correlation between CD55 expression and complement response and observed a strong and statistically significant negative correlation between the per cent of CD55^+^ cells and C3 deposition in each type of hMSCs (Fig. 5G). A similarly strong correlation was detected when all hMSCs were included in the analysis (*R* = 0.9366), showing that CD55 expression could be used to predict complement inhibition.

**Fig. 6:**
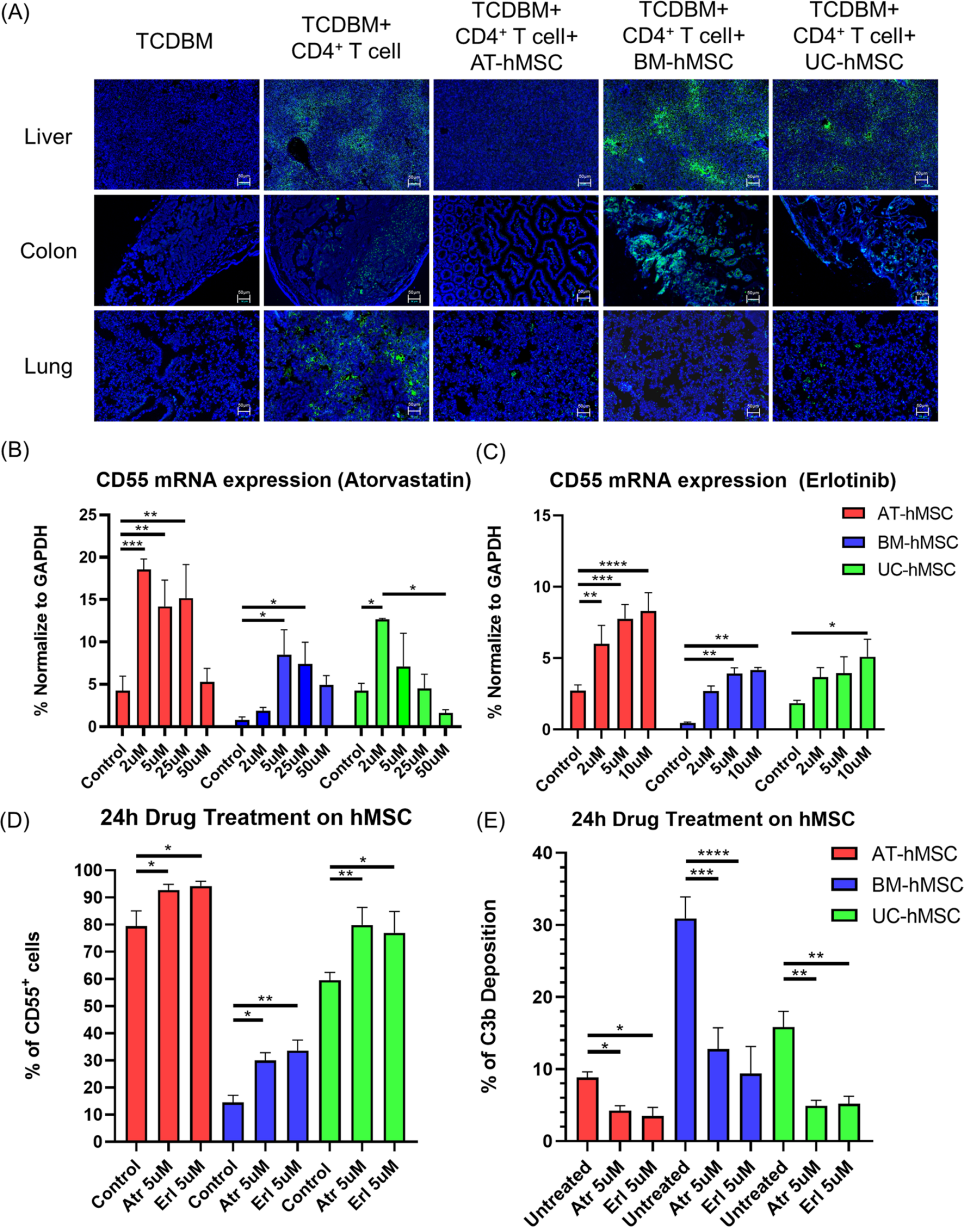
AT-hMSCs can suppress C3 expression in vivo **A** C3 expression in liver, colon and lung samples on day 21 post-transplantation. C3 staining in green, DAPI in blue. Scale bar = 50 μm. Atorvastatin or erlotinib can upregulate CD55 and protect complement lysis of hMSCs upon serum incubation. CD55 mRNA expression (**B** and **C**), and surface protein expression (**D**) in hMSCs upon treatment with atorvastatin or erlotinib for 24 h. Atr: Atorvastatin; Erl: erlotinib. **E** %C3b deposition on hMSCs upon treatment with atorvastatin or erlotinib for 24 h. Graphs show data (mean +/− SEM) of three to four experiments performed in triplicate. Significance was calculated relative to control cells *p < 0.05, **p < 0.01, ***p < 0.001, ****p < 0.0001

Extrapolating from in vitro results, we posited that AT-hMSCs were more resistant to complement injury compared to BM- and UC-hMSCs in vivo*.* We investigated complement activation in our mouse model of aGvHD 21 days post-transplantation by evaluating C3 deposition in different tissues. Immunofluorescence staining revealed strong expression of C3 in the liver, colon and lung from positive mice injected with TCDBM and CD4^+^ T cells. These results are consistent with complement activation contributing to the pathogenesis of aGvHD. Mice transplanted with BM-hMSCs and UC-hMSCs exhibited very strong C3 staining, and this could reflect complement activation against both CD4^+^ T cells and transplanted hMSCs. Conversely, no noticeable C3 staining was observed in the tissues of mice injected with AT-hMSCs (Fig. 6A). These results confirmed that AT-hMSC can actively suppress complement activation in vivo.

### Atorvastatin and erlotinib increased CD55 expression in hMSCs

A previous study reported that atorvastatin, an HMG-coenzyme A inhibitor, could increase CD55 expression in astrocytes [50]. To identify additional putative regulators of CD55 in AT-hMSCs, we performed Pearson correlation analysis with CD55 and other genes in our RNA-Seq dataset. This analysis was used to identify transcripts whose abundance strongly correlated with CD55. Gene ontology analysis revealed enrichment of negative regulators of the EGFR signalling pathway. To test whether erlotinib, which is an EGFR inhibitor, or atorvastatin could upregulate CD55 in hMSCs, we exposed hMSCs to different doses of the two compounds. RT-qPCR and flow analyses showed that erlotinib and atorvastatin increased CD55 mRNA and surface protein levels in a concentration-dependent manner in all three types of hMSCs (Fig. 6B–D). We then incubated BM-, AT- and UC-hMSCs with serum, with and without atorvastatin and erlotinib for 24 h. Following drug treatments, we detected significantly reduced deposition of C3b in these hMSCs relative to controls (Fig. 6E). Inhibition was most pronounced in BM-hMSCs, whose C3 deposition was significantly decreased from 30.9% to 12.8% and 9.4% by atorvastatin and erlotinib, respectively.

## Discussion

The efficacy of hMSC clinical application is limited by poor engraftment and survival [27, 28]. This is partly attributed to the activation of complement lysis cascade, which can damage transplanted cells. Here, we show that AT-hMSCs express higher levels of CD55, an inhibitor of complement, and are more resistant to complement injury compared to BM- and UC-hMSCs. These differences might contribute to the superior effectiveness of AT-hMSCs in promoting survival and in ameliorating the pathological features and symptoms of aGvHD in vivo. We further identified two compounds, erlotinib and atorvastatin, which can upregulate CD55 to suppress complement activation. Our work shows that the use of AT-hMSCs can minimise the adverse effects of complement activation and limit the deleterious effects associated with aGvHD. Furthermore, through the use of small molecules to increase CD55, it may be possible to further promote this protective effect.

Various studies have investigated AT and UC-hMSCs as alternatives to BM-hMSCs. Those studies mostly focused on the paracrine properties of these cells and showed that all three types of hMSCs exhibit immunosuppressive properties in vitro [51, 52]. Consistent with these, we demonstrate that BM-, AT-, and UC-hMSCs all significantly and similarly modulate dendritic cell activation, T-helper cell differentiation, and T-reg proportion in vitro. Whether all three types of hMSCs are similarly efficacious in vivo remains elusive, and there have been few studies to define the benefits of AT-, BM, and UC-hMSCs in vivo against aGvHD. A recent report by Gregoire et al. showed that AT-, UC-, and BM-hMSCs failed to improve overall survival of mice with aGvHD and that AT-MSCs and UC-MSCs were more pro-coagulant than BM-MSCs [53]. However, hMSCs were used as treatment in the aforementioned study and were not infused until symptoms appeared, while our hMSCs were injected prophylactically to prevent aGvHD. The immunosuppressive capacities of hMSCs are affected by inflammatory cytokines [54]. Our own work demonstrated that hMSCs undergo growth arrest and lose their immuno-privilege status upon IFNγ treatment. Therefore, hMSCs given during active inflammation by Gregoire et al. may behave differently from hMSCs administered as prophylaxis [55]. We are thus the first to report the abilities of AT-, UC-hMSCs to protect against aGvHD in vivo, and more importantly, the dichotomy between the similar in vitro immunosuppressive properties of AT-, BM-, and UC-hMSCs and the greater in vivo efficacy of AT-hMSCs in preventing aGvHD.

Our understanding of hMSCs post-transplantation engraftment and survival, particularly in relation to complement, is limited. Here we show that AT-hMSCs could better engraft and survive in host tissues, leading to superior treatment outcomes in vivo*.* This study also implicates CD55 and the suppression of complement activation as major contributors to this phenotype. CD55 has been reported to be upregulated in AT- versus BM-hMSCs by microarray analysis [35]. We confirmed this differential expression using four transcriptomic datasets including our own, along with qPCR and flow cytometry experiments. We further demonstrated that high levels of CD55 played a direct role in protecting AT-hMSCs against complement activation and lysis. It is known that complement activation contributes to allograft rejection. Pratt et al*.* observed a reduction in rejection of kidneys transplanted from C3-deficient donor mice compared to wild-type mice [56], and this was associated with increased survival. C3, thus, can modulate renal transplant rejection. The complement system is also critical for the pathogenesis of aGvHD. The deposition of complement proteins is a major feature of murine aGvHD [57], and C3-deficient mice had a milder aGvHD phenotype compared to the wild-type control [58]. Therefore, the high levels of CD55 in AT-hMSCs can inhibit complement to ameliorate aGvHD both indirectly, by protecting transplanted AT-hMSCs to allow them to exert their immunomodulatory effects, and directly, by suppressing the pathogenesis of aGvHD.

In this study, we demonstrate that AT-hMSCs are a superior alternative to conventionally used BM-hMSCs for the treatment of aGvHD. Although we utilised a mouse model of aGvHD, which may differ from human physiology, our demonstration of improved engraftment and survival supports further investigations of AT-hMSCs. Consistent with the higher in vivo efficacy, we report here against aGvHD, AT-MSCs have been shown to be as good as BM-MSCs at treating Crohn’s disease in mice [59] or more effective in animal models of a wide range of diseases including system sclerosis [17], ischaemic stroke [60], myocardial infarction [61, 62], and spinal injury [63]. While hMSC survival and complement activation were not defined in these studies, it is probable that better survival and complement inhibition by AT-MSCs may contribute to superior outcomes. Clinical trials involving hMSCs are dominated by BM-hMSCs [64]. AT-hMSCs constitute < 10% of clinical trials registered on the NIH registry [64]. These comprise early- and late-stage trials against immune disorders such as Crohn’s disease [17, 65, 66] and acute/chronic GvHD [16, 67] and immune rejection in renal transplantation [68], where AT-hMSCs were generally found to be safe and efficacious. Despite the promising results reported here, coagulation is an important issue for hMSC transplantation [2, 7, 19, 20]. AT-hMSCs have been shown to be more pro-coagulant than BM-hMSCs [28, 69], and we find high levels of procoagulant factor CD142 in this cell population. Regardless, our results show promise for AT-hMSC therapy, but further investigations and clinical trials are needed to confirm the safety and efficacy in the clinic.

Human MSCs are subject to batch-to-batch variations [70]. Hence, there is a critical need for markers or assays that can predict in vivo efficacy [71]. Our data show that the presence of CD55 correlated negatively with complement activation and that assessment of this protein by flow cytometry represents a simple quality control measure to ensure better complement suppression to protect both transplanted hMSCs and host tissues. Lastly, given the prominent role of CD55 in the suppression of complement, increasing the expression of CD55 is a potential way to boost the effectiveness of hMSCs. The pre-treatment (priming) of hMSCs as enhancer for the secretion of anti-inflammatory molecules is not new and here we show that hMSCs can also be primed to increase the expression of complement inhibitors [49, 72]. Soland et al. have shown that transduction of hMSCs with a retrovirus encoding HCMV-US proteins can upregulate CD59 to inhibit complement lysis [73]. Although this provides proof of principle that upregulation of complement inhibitors can protect hMSCs, the aforementioned study utilised genetic manipulation, which may hinder clinical use. Here we identified two FDA-approved compounds that could increase CD55 levels in hMSCs and inhibit complement. Statins, commonly used to treat cardiovascular diseases, have previously been shown to upregulate CD55 and CD59 expression in human umbilical vein endothelial cells. Here, we demonstrated that statin could also upregulate CD55 expression on hMSCs and that erlotinib, an FDA-approved EGFR inhibitor for the treatment of cancer, was able to increase CD55 expression on hMSCs.

## Conclusion

In conclusion, our report identified complement as a crucial barrier to hMSCs transplantation for the treatment of aGvHD. By using AT-hMSCs, which naturally express more CD55, and by priming these cells with atorvastatin or erlotinib, which can enhance the expression of CD55, the activation of complement is suppressed, which directly improves the efficacy of hMSC transplantation. Furthermore, the evaluation of CD55 represents a simple quality control measure to select donor hMSCs, which can best inhibit complement and survive*.* This may achieve the dual benefit of improved survival of transplanted hMSCs and better suppression of complement to inhibit the pathogenesis of aGvHD. Although our research focused on aGvHD, poor engraftment and survival is a limiting factor to cell therapy in general, which may be directly affected by complement activation. Our findings, thus, have the potential to enhance clinical use of hMSCs for the treatment of a wide range of disorders.

## Supplementary Information

**Additional file 1: Supplementary methods.** Supplementary methods for additional experimental details.

**Additional file 2: Table S1.** List of antibodies used in this study.

**Additional file 3: Table S2.** List of primer sequences used in this study.

**Additional file 4: Fig. S1.** **A** Surface protein expression of hMSC-positive antigens: CD44, CD73, CD90 and CD105 and hMSC-negative antigens: HLA-DR, CD11b, CD45, CD14 and CD34. **B** Trilineage differentiation of hMSCs: red staining in the left and middle panels signal adipogenic and osteogenic differentiation, blue pellet indicates chondrogenic differentiation *N* = 3.

**Additional file 5: Table S3.** Mouse aGvHD clinical scoring system.

**Additional file 6: Fig. S2.** **A** Body weightloss in percentage for the 5 cohorts. *n* = 12, 17, 9, 10, 8 for TCDBM, TCDBM+CD4, TCDBM+CD4+AT-hMSCs, TCDBM+ CD4+UC-hMSCs, TCDBM+ CD4+BM-hMSCs. **B** aGvHD clinical manifestations and phenotype of aGvHD mice model. Negative control animalsshowed no abnormality in fur and skin, whereas for positive control animals, loss of fur and scurf was noted. Among the hMSCs treatment groups, AT-hMSCs showed minimal abnormality in skin and fur, whereas BM-hMSC group had a severe loss of fur. Representative image of independent experiments as described in.

**Additional file 7: Fig. S3.** **A** Fluorescence imaging of tissues 21 days after transplantation. Immunofluorescence images of the lung and liver, spleen and colon at 21 days after transplanting CM-Dil labeled hMSCs into mice. **B** Human specific GAPDH mRNA expression of mice liver and colon at day 21 compared with mouse GAPDH. Graphs show data of three experiments with mean +/− SEM. *p < 0.05, **p < 0.01.

**Additional file 8: Fig. S4.** **A** Principal component analysis. **B** Heatmap of sample-sample distancing using Ward’s method. **C** Number of up and down regulated genes in each comparison. Blue bars indicated the upregulated genes, whereas the orange bars indicated downregulated genes. **D**, **E** Volcano plot of AT-hMSCs versus BM-hMSCs and UC-hMSCs versus BM-hMSCs of our dataset.

**Additional file 9: Table S4.** List of Differentially expressed genes between AT-hMSCs and BM-hMSCs. Log2 FoldChange > 1 or < − 1, *p*-value < 0.05.

**Additional file 10: Table S5.** List of Differentially expressed genes between UC-hMSCsand BM-hMSCs. Log2 FoldChange > 1 or < − 1, *p*-value < 0.05.

**Additional file 11: Table S6.** List of Differentially expressed genes between AT-hMSCs and UC-hMSCs. Log2 FoldChange > 1 or < − 1, *p*-value < 0.05.

**Additional file 12: Fig. S5.** Blue = isotype control. Red = anti-CD59.

## Data Availability

For original data, please contact ellen.poon@cuhk.edu.hk or gcfchan@hku.hk. The datasets used in the current study are available from the corresponding authors on reasonable request. RNA sequencing data can be found via GEO accession number GSE219227.

## Abbreviations

aGvHD: Acute graft-versus-host-disease
HSCT: Hematopoietic stem cell transplantation
hMSCs: Human mesenchymal stromal cells
AT: Adipose tissue
BM: Bone marrow
UC: Umbilical cord
CD55: Complement-decay accelerating factor
ISCT: International Society for Cellular Therapy
Treg: T-regulatory
TCDBM: T cell-depleted bone marrow
Th-1: T-helper-1
Th-2: T-helper-2

## References

[1] Shek TWH (1996). The pathology of bone marrow transplantation in Hong Kong Chinese. Hong Kong Med J.

[2] Zeiser R, Blazar BR (2017). Acute graft-versus-host disease biology, prevention and therapy. N Engl J Med.

[3] Axt L (2019). Retrospective single center analysis of outcome, risk factors and therapy in steroid refractory graft-versus-host disease after allogeneic hematopoietic cell transplantation. Bone Marrow Transpl.

[4] Westin JR (2011). Steroid-refractory acute GvHD: predictors and outcomes. Adv Hematol.

[5] Ringden O (2018). Placenta-derived decidua stromal cells for treatment of severe acute graft-versus-host disease. Stem Cells Transl Med.

[6] Moll G, Hoogduijn MJ, Ankrum JA (2020). Editorial: safety, efficacy and mechanisms of action of mesenchymal stem cell therapies. Front Immunol.

[7] Ringdén O, Moll G, Gustafsson B, Sadeghi B (2022). Mesenchymal stromal cells for enhancing hematopoietic engraftment and treatment of graft-versus-host disease, hemorrhages and acute respiratory distress syndrome. Front Immunol.

[8] Zeiser R (2020). Ruxolitinib for glucocorticoid-refractory acute graft-versus-host disease. N Engl J Med.

[9] Aggarwal S, Pittenger MF (2005). Human mesenchymal stem cells modulate allogeneic immune cell responses. Blood.

[10] Li Y, Fung J, Lin F (2016). Local inhibition of complement improves mesenchymal stem cell viability and function after administration. Mol Ther.

[11] Viswanathan S (2019). Mesenchymal stem versus stromal cells: International Society for Cell & gene therapy (ISCT^®^) mesenchymal stromal cell committee position statement on nomenclature. Cytotherapy.

[12] Bianco P (2013). The meaning, the sense and the significance: translating the science of mesenchymal stem cells into medicine. Nat Med.

[13] LeBlanc K (2008). Mesenchymal stem cells for treatment of steroid-resistant, severe, acute graft-versus-host disease: a phase II study. Lancet (Lond, Engl).

[14] Fisher SA (2019). Mesenchymal stromal cells as treatment or prophylaxis for acute or chronic graft-versus-host disease in haematopoietic stem cell transplant (HSCT) recipients with a haematological condition. Cochrane Database Syst Rev.

[15] Döring M (2021). Long-term follow-up after the application of mesenchymal stromal cells in children and adolescents with steroid-refractory graft-versus-host disease. Stem Cells Dev.

[16] Munneke JM (2016). The potential of mesenchymal stromal cells as treatment for severe steroid-refractory acute graft-versus-host disease: a critical review of the literature. Transplantation.

[17] Maria ATJ (2016). Human adipose mesenchymal stem cells as potent anti-fibrosis therapy for systemic sclerosis. J Autoimmun.

[18] VonBahr L (2012). Analysis of tissues following mesenchymal stromal cell therapy in humans indicates limited long-term engraftment and no ectopic tissue formation. Stem Cells.

[19] Moll G (2019). Intravascular mesenchymal stromal/stem cell therapy product diversification: time for new clinical guidelines. Trends Mol Med.

[20] Moll G, Ankrum JA, Olson SD, Nolta JA (2022). Improved MSC minimal criteria to maximize patient safety: a call to embrace tissue factor and hemocompatibility assessment of MSC products. Stem Cells Transl Med.

[21] Li Y, Lin F (2012). Mesenchymal stem cells are injured by complement after their contact with serum. Blood.

[22] Moll G (2011). Mesenchymal stromal cells engage complement and complement receptor bearing innate effector cells to modulate immune responses. PLoS ONE.

[23] Moll G (2012). Are therapeutic human mesenchymal stromal cells compatible with human blood?. Stem Cells.

[24] Moll G (2014). Do cryopreserved mesenchymal stromal cells display impaired immunomodulatory and therapeutic properties?. Stem Cells.

[25] Cottle C (2022). Impact of cryopreservation and freeze-thawing on therapeutic properties of mesenchymal stromal/stem cells and other common cellular therapeutics. Curr Stem Cell Rep.

[26] Moll G (2016). Cryopreserved or fresh mesenchymal stromal cells: Only a matter of taste or key to unleash the full clinical potential of MSC therapy?. Adv Exp Med Biol.

[27] Huerta CT, Ortiz YY, Liu Z-J, Velazquez OC (2022). Methods and limitations of augmenting mesenchymal stem cells for therapeutic applications. Adv Wound Care.

[28] Meng H, Jin J, Wang H, Wang LS, Wu CT (2022). Recent advances in the therapeutic efficacy of hepatocyte growth factor gene-modified mesenchymal stem cells in multiple disease settings. J Cell Mol Med.

[29] Dmitrieva RI (2012). Bone marrow- and subcutaneous adipose tissue-derived mesenchymal stem cells: differences and similarities. Cell Cycle.

[30] Kern S, Eichler H, Stoeve J, Klüter H, Bieback K (2006). Comparative analysis of mesenchymal stem cells from bone marrow, umbilical cord blood, or adipose tissue. Stem Cells.

[31] Ivanova-Todorova E (2009). Adipose tissue-derived mesenchymal stem cells are more potent suppressors of dendritic cells differentiation compared to bone marrow-derived mesenchymal stem cells. Immunol Lett.

[32] Li CY (2015). Comparative analysis of human mesenchymal stem cells from bone marrow and adipose tissue under xeno-free conditions for cell therapy. Stem Cell Res Ther.

[33] Mohamed-Ahmed S (2018). Adipose-derived and bone marrow mesenchymal stem cells: a donor-matched comparison. Stem Cell Res Ther.

[34] Moll G (2015). Different procoagulant activity of therapeutic mesenchymal stromal cells derived from bone marrow and placental decidua. Stem Cells Dev.

[35] Ménard C (2020). Integrated transcriptomic, phenotypic, and functional study reveals tissue-specific immune properties of mesenchymal stromal cells. Stem Cells.

[36] Mo IFY (2008). Prolonged exposure to bacterial toxins downregulated expression of toll-like receptors in mesenchymal stromal cell-derived osteoprogenitors. BMC Cell Biol.

[37] Dominici M (2006). Minimal criteria for defining multipotent mesenchymal stromal cells. The International Society for Cellular Therapy position statement. Cytotherapy.

[38] Nie Y, Lau CS, Lie AKW, Chan GCF, Mok MY (2010). Defective phenotype of mesenchymal stem cells in patients with systemic lupus erythematosus. Lupus.

[39] Moradi B (2014). CD4+CD25+/highCD127low/− regulatory T cells are enriched in rheumatoid arthritis and osteoarthritis joints–analysis of frequency and phenotype in synovial membrane, synovial fluid and peripheral blood. Arthr Res Ther.

[40] Robles DJ (2015). Immunosuppressive mechanisms of human bone marrow derived mesenchymal stromal cells in BALB/c host graft versus host disease murine models. Exp Hematol Oncol.

[41] Cooke KR (1996). An experimental model of idiopathic pneumonia syndrome after bone marrow transplantation: I. The roles of minor H antigens and endotoxin. Blood.

[42] Leibacher J, Henschler R (2016). Biodistribution, migration and homing of systemically applied mesenchymal stem/stromal cells. Stem Cell Res Ther.

[43] Saulnier N (2011). Gene profiling of bone marrow- and adipose tissue-derived stromal cells: a key role of Kruppel-like factor 4 in cell fate regulation. Cytotherapy.

[44] Lim MN (2014). Comparative global gene expression profile of human limbal stromal cells, bone marrow mesenchymal stromal cells, adipose-derived mesenchymal stromal cells and foreskin fibroblasts. Stem Cell Biol Res.

[45] Hsieh JY (2013). Mesenchymal stem cells from human umbilical cord express preferentially secreted factors related to neuroprotection, neurogenesis, and angiogenesis. PLoS ONE.

[46] Reinisch A (2015). Epigenetic and in vivo comparison of diverse MSC sources reveals an endochondral signature for human hematopoietic niche formation. Blood.

[47] Donders R (2018). Human Wharton’s Jelly-derived stem cells display a distinct immunomodulatory and proregenerative transcriptional signature compared to bone marrow-derived stem cells. Stem Cells Dev.

[48] Oglesby TJ, Allen CJ, Liszewski MK, White DJG, Atkinson JP (1992). Membrane cofactor protein (CD46) protects cells from complement-mediated attack by an intrinsic mechanism. J Exp Med.

[49] Pangburn MK, Schreiber RD, Müller-Eberhard HJ (1983). C3b deposition during activation of the alternative complement pathway and the effect of deposition on the activating surface. J Immunol.

[50] Tradtrantip L, Duan T, Yeaman MR, Verkman AS (2019). CD55 upregulation in astrocytes by statins as potential therapy for AQP4-IgG seropositive neuromyelitis optica. J Neuroinflamm.

[51] Yañez R (2006). Adipose tissue-derived mesenchymal stem cells have in vivo immunosuppressive properties applicable for the control of the graft-versus-host disease. Stem Cells.

[52] Yoo KH (2009). Comparison of immunomodulatory properties of mesenchymal stem cells derived from adult human tissues. Cell Immunol.

[53] Grégoire C (2019). Comparison of mesenchymal stromal cells from different origins for the treatment of graft-vs.-host-disease in a humanized mouse model. Front Immunol.

[54] Shi Y (2018). Immunoregulatory mechanisms of mesenchymal stem and stromal cells in inflammatory diseases. Nat Rev Nephrol.

[55] Chan WK (2008). MHC expression kinetics and immunogenicity of mesenchymal stromal cells after short-term IFN-gamma challenge. Exp Hematol.

[56] Pratt JR, Basheer SA, Sacks SH (2002). Local synthesis of complement component C3 regulates acute renal transplant rejection. Nat Med.

[57] Niculescu F (2005). Both apoptosis and complement membrane attack complex deposition are major features of murine acute graft-vs.-host disease. Exp Mol Pathol.

[58] Ma Q (2012). Reduced graft-versus-host disease in C3-deficient mice is associated with decreased donor Th1/Th17 differentiation. Biol Blood Marrow Transpl.

[59] Xie M (2017). Comparison of adipose-derived and bone marrow mesenchymal stromal cells in a murine model of Crohn’s Disease. Dig Dis Sci.

[60] Ikegame Y (2011). Comparison of mesenchymal stem cells from adipose tissue and bone marrow for ischemic stroke therapy. Cytotherapy.

[61] Paul A, Srivastava S, Chen G, Shum-Tim D, Prakash S (2013). Functional assessment of adipose stem cells for xenotransplantation using myocardial infarction immunocompetent models: comparison with bone marrow stem cells. Cell Biochem Biophys.

[62] Rasmussen JG (2014). Comparison of human adipose-derived stem cells and bone marrow-derived stem cells in a myocardial infarction model. Cell Transpl.

[63] Zhou Z (2013). Comparison of mesenchymal stromal cells from human bone marrow and adipose tissue for the treatment of spinal cord injury. Cytotherapy.

[64] Naji A (2019). Biological functions of mesenchymal stem cells and clinical implications. Cell Mol Life Sci.

[65] Li Y (2022). Current status of clinical trials assessing mesenchymal stem cell therapy for graft versus host disease: a systematic review. Stem Cell Res Ther.

[66] Kotze PG (2019). Darvadstrocel for the treatment of patients with perianal fistulas in Crohn’s disease. Drugs Today (Barc).

[67] Jurado M (2017). Adipose tissue-derived mesenchymal stromal cells as part of therapy for chronic graft-versus-host disease: a phase I/II study. Cytotherapy.

[68] Vanikar AV (2014). Co-infusion of donor adipose tissue-derived mesenchymal and hematopoietic stem cells helps safe minimization of immunosuppression in renal transplantation—single center experience. Ren Fail.

[69] Christy BA (2017). Procoagulant activity of human mesenchymal stem cells. J Trauma Acute Care Surg.

[70] Jayaraman P, Lim R, Ng J, Vemuri MC (2021). Acceleration of translational mesenchymal stromal cell therapy through consistent quality GMP manufacturing. Front Cell Dev Biol.

[71] Krampera M, LeBlanc K (2021). Mesenchymal stromal cells: Putative microenvironmental modulators become cell therapy. Cell Stem Cell.

[72] Yang R (2020). IFN-γ promoted exosomes from mesenchymal stem cells to attenuate colitis via miR-125a and miR-125b. Cell Death Dis.

[73] Soland MA (2013). Mesenchymal stem cells engineered to inhibit complement-mediated damage. PLoS ONE.

