# Supplementary material for: Adipose tissue-derived human mesenchymal stromal cells can better suppress complement lysis, engraft and inhibit acute graft-versus-host disease in mice
Source: Stem Cell Res Ther. 2023 Jun 25;14:167. doi: 10.1186/s13287-023-03380-x (PMC10291819; doi:10.1186/s13287-023-03380-x)
Supplement: Supplementary file 1 — Additional file 1: Supplementary methods. Supplementary methods for additional experimental details. [file 13287_2023_3380_MOESM1_ESM.pdf]

Supplementary Methods:

Trilineage differentiation of hMSCs

Human MSCs were directed toward the osteogenic, adipogenic, and chondrogenic lineages with appropriate induction media, and differentiation potential was assessed as previously described [1,2]. **Adipogenic differentiation:** hMSCs were seeded in the control medium at $2x10^5$ cells in a 6-well tissue culture plates. The following day (day 0), hMSCs cultures were induced to adipogenic differentiation by culturing in adipogenic induction (MDI+I) medium containing 1 μM dexamethasone (Sigma-Aldrich) and 0.5 mM methylisobutylxanthine (Sigma-Aldrich), 10μg/ml insulin (Thermo Fisher Scientific), 100 μM indomethacin (Sigma-Aldrich) and 10% FBS in DMEM-high glucose for 2 to 3 weeks. **Osteogenic Differentiation:** hMSCs were seeded at $2x10^5$ cells in a 6-well tissue culture plates. The following day (day 0), hMSC were induced to osteogenic differentiation by culturing with StemPro™ Osteogenesis Differentiation Kit (Thermo Fisher Scientific) for 2 to 3 weeks. **Chondrogenic Differentiation:** hMSCs were seeded at $2x10^5$ cells in a 6-well plate. The following day (day 0), hMSC were induced to osteogenic differentiation by culturing with StemPro™ Chondrogenic Differentiation Kit (Thermo Fisher Scientific) for 2 to 3 weeks.

Monitoring: Adipogenic differentiation was demonstrated by the accumulation of neutral lipid vacuoles indicated by the Oil Red O stain (Sigma Aldrich). For hMSC osteogenic differentiation, differentiation was monitored with Alizarin Red S (Sigma Aldrich). For chondrogenic differentiation, cartilage formation was detected with Alcian Blue staining (Sigma Aldrich).

Flow cytometric analysis

Human MSCs at various passages (P2-P6) were dissociated into single cells with 0.25% Trypsin-EDTA (Thermo Fisher Scientific) and blocked with 10% fetal bovine serum (FBS) in Phosphate buffered saline (PBS) for 15 minutes on ice, then stained with antibodies against CD4, CD11b, CD14, CD25, CD34, CD44, CD45, CD55, CD59, CD73, CD90, CD105, CD127, CD142 and HLA-DR, in 0.1% BSA for 30 min in the dark. Afterward, the cells were washed with PBS twice and resuspended in 0.1% BSA. The cells were analyzed by FACS Canto II or FACSCelesta (BD Biosciences) and the data were analysed with FlowJo 7.0 (Ashland). A list of antibodies can be found in Additional file 2: Table. S1.

Isolation of DC, T-helper cells and NK cells

DC were isolated from human peripheral blood mononuclear cells (PBMC) using human CD14 microbeads (Miltenyl Biotec) following the manufacturer's protocol. T-helper cells were isolated from PBMCs using magnetic cell isolation following the manufacturer's protocol (Miltenyl Biotec). Specifically, PBMCs ($1x10^7$ cells in 40µL of buffer) were incubated with 10 µL of Naïve CD4+ T cell biotin-antibody ((Miltenyl Biotec). Naive CD4+ T Cell MicroBead (Miltenyl Biotec) was then added to the cells for magnetic cell isolation to isolate T-helper cells. NK cells were similarly isolated using NK Cell Biotin-Antibody and microbead (Mitenyl Biotec).

***In vitro* modulatory assays**

The immunomodulatory properties of hMSCs were evaluated as previously described with some modifications [3]. For dendritic cells, hMSCs were co-cultured with dendritic cells from unrelated donors, stimulated with bacterial lipopoly-saccharide. The secretion of pro-inflammatory TNF-α was measured by ELISA assay. For T-cell differentiation, PBMCs were co-cultured with hMSCs in Th-1 or Th-2 inducing conditions. The release of effector cytokines (IFN-γ for Th-1 and IL-4, IL-10 for Th-2) was monitored by ELISA assay. The effect of hMSCs on natural killer cells was assessed by monitoring the secretion of IFN-γ upon interleukin-2 stimulation. Concentration of TNFα, IL-4 , IL-10 and IFN-α were

measured using ELISA kits. (ELISA MAX™ Deluxe Set Human IL-4, Biolegend; ELISA MAX™ Deluxe Set Human IL-10, Biolegend; ELISA MAX™ Deluxe Set Human TNFα, Biolegend; ELISA MAX™ Deluxe Set Human IFN-α2, Biolegend). The plates were then analyzed by Synergy H1 Hybrid microplate reader with appropriate excitation and emission wavelength. To evaluate the effect of hMSCs on T-regulatory (Treg) percentage, hMSCs were cultured with PBMCs. Non-adherent T cells were harvested and the proportion of Tregs present were measured by flow cytometry using anti-CD4, CD25 and CD127 antibodies [4].

Establishment of mouse model of aGVHD

BALB/C host mice was exposed to γ-irradiation at 800 cGy around 24 hours prior to cell treatment. $2x10^6$ T cell depleted bone marrow from the femur and $0.25x10^6$ T cells from the spleen of C57BL/6N donor mice were injected via the tail vein to induce a graft versus host reaction [5]. Survival was monitored for 80 days by Kaplan-Meier analysis. A clinical scoring system was used to monitor signs of aGVHD in host mice [6]. All experiments were performed in accordance with the guidelines and approved protocols of the Committee on the Use of Live Animals in Teaching and Research of the University of Hong Kong.

hMSC survival and homing

Human MSCs were stained with 5µM CM-Dil dye (Thermo Fisher Scientific) for 5 minutes at 37°C and 15 minutes at 4°C, then the cells were washed 3 times with DPBS for injection. At 7- and 21-days post transplantation, the spleen, liver, lung, colon, kidney, and heart were harvested. The fluorescent signal intensity of the stained cells in the tissues was evaluated using the IVIS Spectrum In Vivo Imaging System (CRi Maestro II, Cambridge Research & Instrumentation, Inc., Woburn, MA). The wavelengths of absorption and excitation were set to be 535 nm and 580 nm, respectively. Data were analysed using the Living Image software (PerkinElmer, USA) to calculate the average signal intensity of regions of interest (ROI) for each tissue. The lowest signal was adjusted to the level of autofluorescence background.

Histopathological analysis of GVHD

Mice were sacrificed by cervical dislocation on days 7 and 21 following GVHD induction. Tissue samples from the liver, colon, lung, and spleen were fixed in 4% paraformaldehyde, embedded in paraffin and cut into 5 µm sections by Leica RM2135 microtome (Leica Microsystems) and stained with Haematoxylin and Eosin (H&E) , then

mounted with DPX (Dibutylphthalate Polystyrene Xylene) (Sigma Aldrich) for histologic examination.

Clinical scoring of GVHD mice

The development of aGVHD in our mouse model was evaluated based on a mouse aGVHD scoring system [6]. The scoring system for aGVHD had five clinical criteria, and these are described (maximum score =10) (Additional file 5: Table. S3). Weight loss with less than 10% was scored 0, weight loss with greater than 10% and less than 25% was scored as 1, and weight loss with greater than 25% was scored as 2. For posture and activity, the scoring system denoted 0 as normal, while 1 was used for hunching at rest, and 2 was used for serious hunching. For the fur texture, normal fur texture was scored as 0, 1 mild fur loss and ruffing was scored as 1, and severe loss of fur was scored as 2. Each mouse was accessed 3 times a week to generate the total clinical GVHD score.

Immunohistochemistry

Tissue sections were deparaffinized and treated with heated citrate buffer (CB) at (0.1N HCL, pH = 6) for antigen retrieval. Sections were blocked with protein block solution (DAKO, DK) to minimise endogenous peroxidase activity and incubated with primary

antibodies at 4°C overnight. After washing, secondary antibodies (Thermo Fisher Scientific) were applied for 1 hour at room temperature in the dark. Sections were mounted with ProLong™ Gold Antifade Mountant with DAPI (Thermo Fisher Scientific) and were imaged with LSM 700 Laser Scanning Microscope (Zeiss).

Real time-qPCR

1 μg of total RNA was used for the synthesis of cDNA using PrimeScript RT Reagent Kit (Takara) as per the manufacturer's recommendations. These cDNA was then mixed with qPCR mastermix (Promega) and RT-qPCR was performed using an ABI-Prism 7900HT PCR machine (Applied Biosystems). The relative expression of genes was analysed with $2^{-\Delta Ct}$ method, normalised against GAPDH, where $\Delta Ct = Ct$ target gene – Ct of GAPDH. All the experiments were carried out in triplicate. The primer sequences of specific gene are listed at Additional file 3: Table. S2.

C3 deposition assay

Human MSCs were seeded on a well of a 6-well plate at a concentration of 2×10^5 per well. When cells reached 80% confluence, they were dissociated using 0.25% Trypsin. The cell suspension was then incubated at 37°C for 30 minutes with 30 μl of normal

BALB/c serum in 100 µl gelatin veronal buffer^{++} (GVB^{++}). Cells were spun down and washed with 1 mL of FACS buffer (0.1% BSA in PBS) twice, then incubated with 5 µg/mL of FITC-labeled goat anti-mouse C3 IgG (55500, MpBio). After washing, cells were resuspended with FACS buffer and analyzed by flow cytometry (LSR I or FACSCelesta; BD Bioscience).To examine the role of CD55, single cell suspension was first incubated with anti-human CD55 antibody for 30 minutes before incubation with serum and was analysed as described above.

***In vitro* cytotoxicity assay**

Human MSCs were dissociated, incubated with 5 µM of BCECF-AM (Thermo Fisher Scientific) for 30 minutes at 37°C and washed twice with PBS. Human MSCs incubated with 0.1% Triton X-100 were treated as positive control. The labelled hMSCs were then incubated at 37°C for 30 minutes with 30 µl of normal BALB/c serum in 100 µl gelatin veronal buffer^{++} and spun down. The supernatant was harvested, and the released BCECF was measured by Synergy H1 Hybrid Multi-Mode Reader (BioTek Instruments, Inc, Winooski, VT, USA) with excitation and emission wavelengths of 485 nm and 538 nm. To calculate the percentage of BCECF release (complement mediated injury), the following equation was used: percentage of BCECF release (cytotoxicity) = [(A−B)/(C−B)] ×100%;

where A represents the mean experimental BCECF release, B represents the mean spontaneous BCECF release, and C represents the mean maximum BCECF released that was induced by incubating cells with 0.1% Triton X-100.

RNA-sequencing

Total RNA was isolated using the TRIzol™ Reagent (Thermo Fisher Scientific) as per manufacturer protocol. RNA-seq was carried out at Centre for PanorOmic Sciences of the University of Hong Kong. RNAs with polyA tails were isolated, and double-stranded cDNA libraries were prepared using TruSeq RNA Kit (Illumina) followed by paired-end sequencing using Illumina NovaSeq 6000. The reads were aligned by STAR [7]. Raw counts were annotated into General Transfer Format (GTF) file format. Gene Ontology and KEGG analysis were used based on clusterProfiler packages [8]. All analyses were performed in RStudio 1.4.1717 version. The differentially expressed genes (DEGs) was analyzed by the Deseq2 R package for the normalization of the RNA-seq data [9]. Up regulated genes were defined by log fold change >1, p-value <0.05, whereas down regulated genes were defined by log fold change <1 and p-value <0.05.

Data processing and analysis

Experimental parameters from the different groups of hMSCs were expressed as mean ± SEM and compared using ANOVA test. $P<0.05$ was considered the threshold of significance.

Supplementary Reference:

1. Li, J., Kwong, D. L. W. &Chan, G. C. F. The effects of various irradiation doses on the growth and differentiation of marrow-derived human mesenchymal stromal cells. *Pediatr. Transplant.* **11**, 379–387 (2007).
2. Chan, B. P., Hui, T. Y., Wong, M. Y., Yip, K. H. K. &Chan, G. C. F. Mesenchymal stem cell-encapsulated collagen microspheres for bone tissue engineering. *Tissue Eng. Part C. Methods* **16**, 225–235 (2010).
3. Aggarwal, S. &Pittenger, M. F. Human mesenchymal stem cells modulate allogeneic immune cell responses. *Blood* **105**, 1815–1822 (2005).
4. Maria, A. T. J. *et al.* Human adipose mesenchymal stem cells as potent anti-fibrosis therapy for systemic sclerosis. *J. Autoimmun.* **70**, 31–39 (2016).
5. Cooke, K. R. *et al.* An experimental model of idiopathic pneumonia syndrome after bone marrow transplantation: I. The roles of minor H antigens and endotoxin. *Blood* **88**, 3230–3239 (1996).

6. Robles, D. J. *et al.* Immunosuppressive mechanisms of human bone marrow derived mesenchymal stromal cells in BALB/c host graft versus host disease murine models. *Exp. Hematol. Oncol.* **4**, (2015).
7. Dobin, A. *et al.* STAR: ultrafast universal RNA-seq aligner. *Bioinformatics* **29**, 15–21 (2013).
8. Yu, G., Wang, L. G., Han, Y. &He, Q. Y. clusterProfiler: an R package for comparing biological themes among gene clusters. *OMICS* **16**, 284–287 (2012).
9. Love, M. I., Huber, W. &Anders, S. Moderated estimation of fold change and dispersion for RNA-seq data with DESeq2. *Genome Biol.* **15**, 1–21 (2014).
