# Supplementary material for: Adipose tissue-derived human mesenchymal stromal cells can better suppress complement lysis, engraft and inhibit acute graft-versus-host disease in mice
Source: Stem Cell Res Ther. 2023 Jun 25;14:167. doi: 10.1186/s13287-023-03380-x (PMC10291819; doi:10.1186/s13287-023-03380-x)
Supplement: Supplementary file 2 — Additional file 2: Table S1. List of antibodies used in this study. [file 13287_2023_3380_MOESM2_ESM.pdf]

Table S1. List of antibodies used in this study.

Parameter		Manufacturer	Catalog no.
Pacific blue	CD14	Biolegend	103020
	CD44	BD Biosciences	558121
	CD11b	BD Biosciences	558123
APC	CD19	BD Biosciences	555415
	CD105	Biolegend	323208
	HLA-DR	BD Biosciences	559866
	CD127	Biolegend	351316
PerCP-Cy 5.5	CD34	Biolegend	343612
	CD45	BD Biosciences	564105
	CD90	BD Biosciences	561557
FITC	C3	MP Biomedicals	55510
	CD4	Biolegend	300506
	CD55	Biolegend	311306
PE	CD25	Biolegend	302606
