# Supplementary material for: Adipose tissue-derived human mesenchymal stromal cells can better suppress complement lysis, engraft and inhibit acute graft-versus-host disease in mice
Source: Stem Cell Res Ther. 2023 Jun 25;14:167. doi: 10.1186/s13287-023-03380-x (PMC10291819; doi:10.1186/s13287-023-03380-x)
Supplement: Supplementary file 3 — Additional file 3: Table S2. List of primer sequences used in this study. [file 13287_2023_3380_MOESM3_ESM.pdf]

**Table S2. List of primer sequences used in this study.**

| Gene | Direction | Sequence |
|---|---|---|
| CD55 | Forward | AGAGTTCTGCAATCGTAGCTGC |
| | Reverse | CACAACAGTACCGACTGGAAAAT |
| Mouse CCL3 | Forward | AAGGATACAAGCAGCAGCGAGTA |
| | Reverse | TGCAGAGTGTCATGGTACAGAGAA |
| Mouse CXCL9 | Forward | TGTGGAGTTCGAGGAACCCT |
| | Reverse | TGCCTTGGCTGGTGCTG |
| Human specific GAPDH | Forward | TCAAGGCTGAGAACGGGAAG |
| | Reverse | CGCCCCACTTGATTTTGGAG |
| Mouse GAPDH | Forward | GCGAGACCCCACTAACATCA |
| | Reverse | GGCGGAGATGATGACCCTTT |
| Human GAPDH | Forward | TCACCACCATGGAGAAGGC |
| | Reverse | GCTAAGCAGTTGGTGGTGCA |
