# Supplementary figures and images for: Adipose tissue-derived human mesenchymal stromal cells can better suppress complement lysis, engraft and inhibit acute graft-versus-host disease in mice
Source: Stem Cell Res Ther. 2023 Jun 25;14:167. doi: 10.1186/s13287-023-03380-x (PMC10291819; doi:10.1186/s13287-023-03380-x)

(A)

| % Positive cell | AT-hMSC     | BM-hMSC     | UC-hMSC     |
|-----------------|-------------|-------------|-------------|
| HLA-DR          | 0.71 ± 0.05 | 1.22 ± 0.12 | 1.53 ± 0.23 |
| CD11b           | 0.39 ± 0.06 | 1.68 ± 0.12 | 0.57 ± 0.09 |
| CD45            | 1.37 ± 0.24 | 0.76 ± 0.11 | 2.00 ± 0.49 |
| CD14            | 1.70 ± 0.35 | 1.08 ± 0.31 | 1.75 ± 0.28 |
| CD34            | 1.61 ± 0.15 | 1.97 ± 0.11 | 1.86 ± 0.21 |
| CD90            | 98.7 ± 1.52 | 98.5 ± 1.25 | 98.8 ± 1.36 |
| CD105           | 98.6 ± 0.85 | 98.2 ± 1.45 | 98.6 ± 1.12 |
| CD44            | 98.4 ± 1.01 | 98.4 ± 0.85 | 98.6 ± 0.71 |

(B)

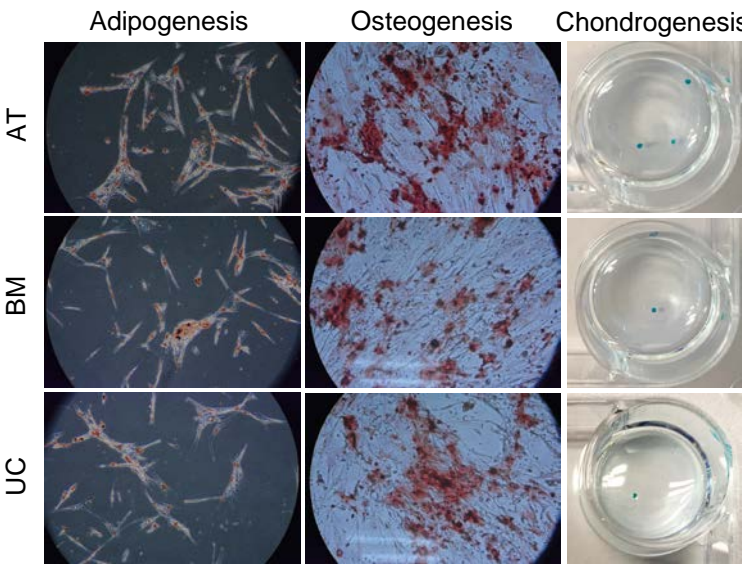

Supplement: Supplementary file 4 — Additional file 4: Fig. S1. A Surface protein expression of hMSC-positive antigens: CD44, CD73, CD90 and CD105 and hMSC-negative antigens: HLA-DR, CD11b, CD45, CD14 and CD34. B Trilineage differentiation of hMSCs: red staining in the left and middle panels signal adipogenic and osteogenic differentiation, blue pellet indicates chondrogenic differentiation N = 3. [file 13287_2023_3380_MOESM4_ESM.pdf]

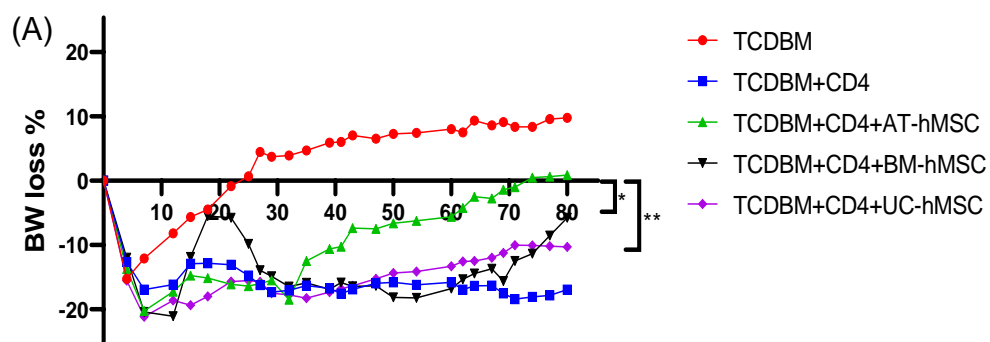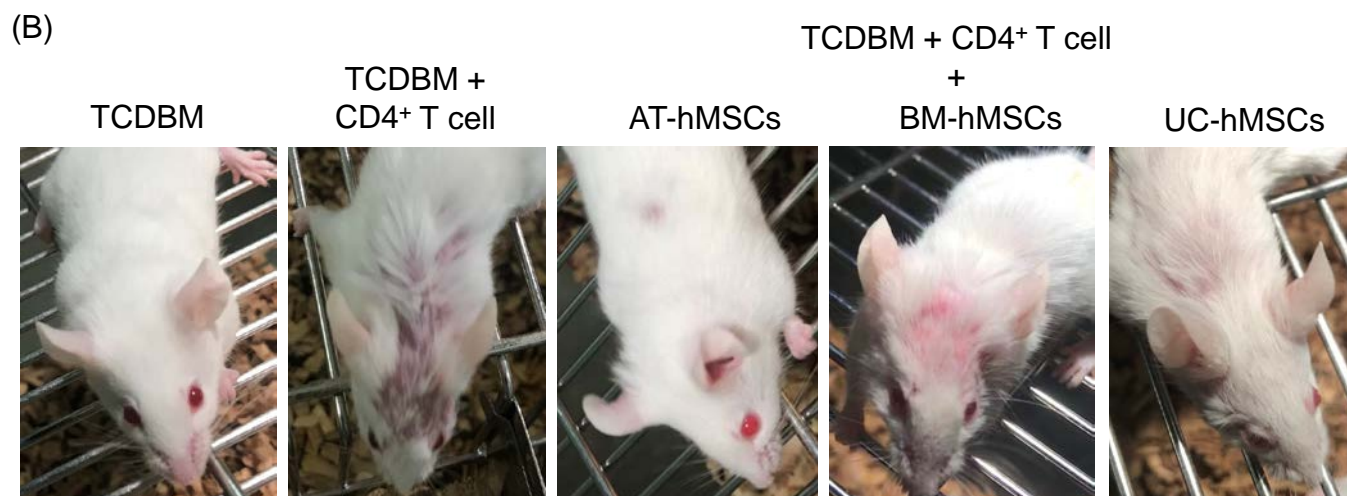

Supplement: Supplementary file 6 — Additional file 6: Fig. S2. A Body weightloss in percentage for the 5 cohorts. n = 12, 17, 9, 10, 8 for TCDBM, TCDBM+CD4, TCDBM+CD4+AT-hMSCs, TCDBM+ CD4+UC-hMSCs, TCDBM+ CD4+BM-hMSCs. B aGvHD clinical manifestations and phenotype of aGvHD mice model. Negative control animalsshowed no abnormality in fur and skin, whereas for positive control animals, loss of fur and scurf was noted. Among the hMSCs treatment groups, AT-hMSCs showed minimal abnormality in skin and fur, whereas BM-hMSC group had a severe loss of fur. Representative image of independent experiments as described in. [file 13287_2023_3380_MOESM6_ESM.pdf]

(A)

Lung

Liver

Spleen

Colon

TCDBM TCDBM +CD4 BM-hMSCAT-hMSC UC-hMSC

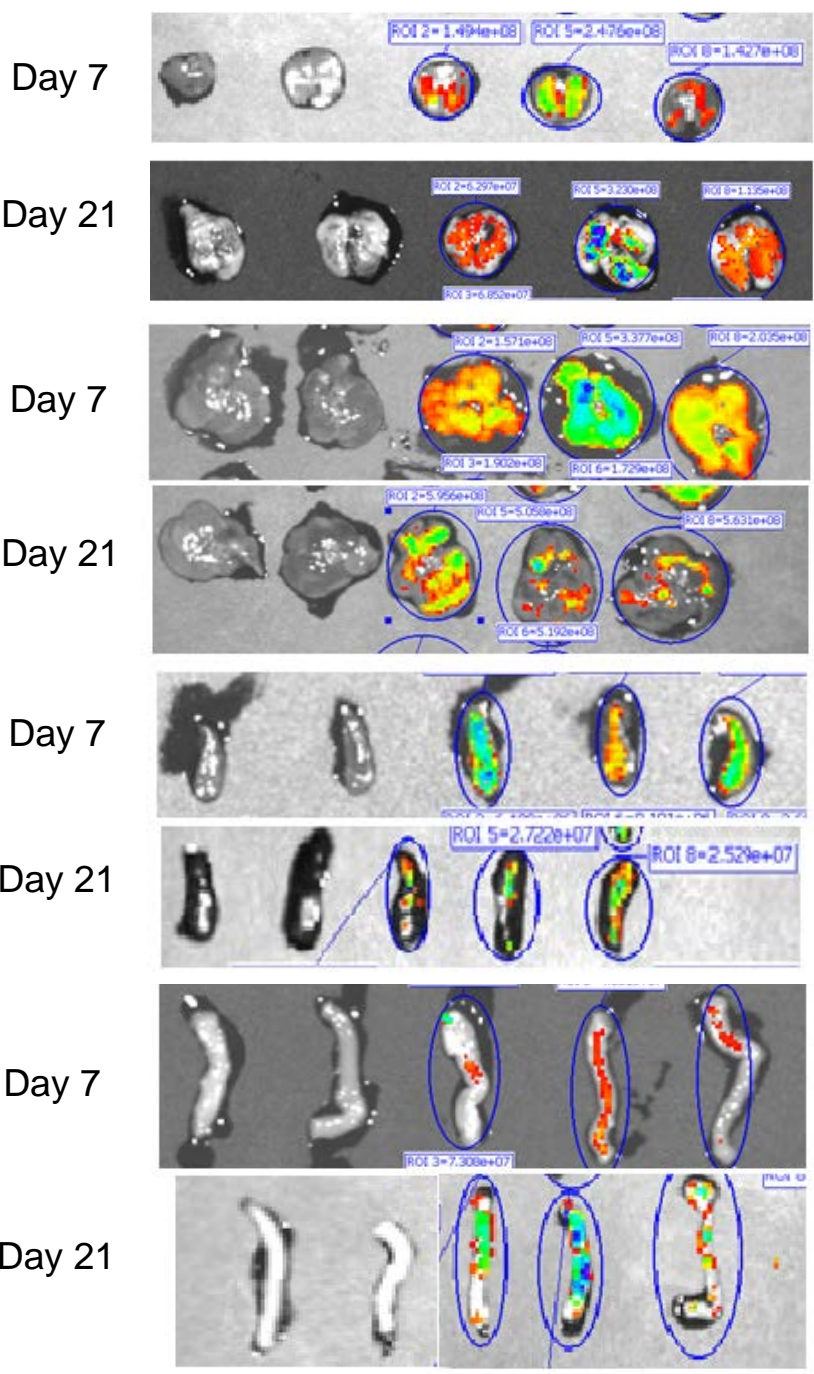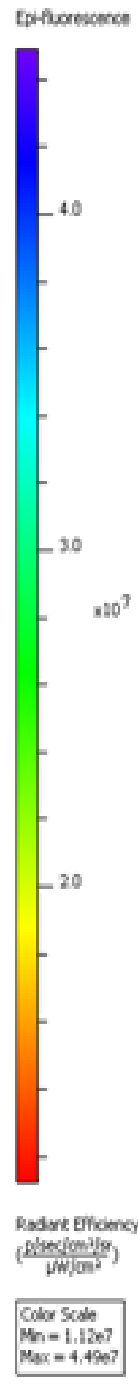

(B)

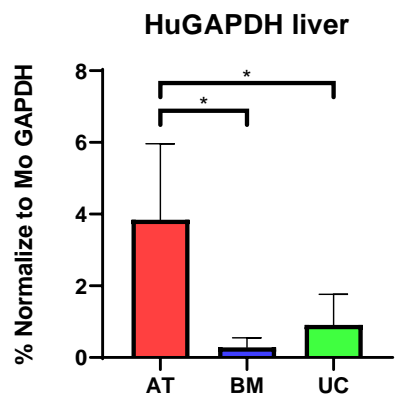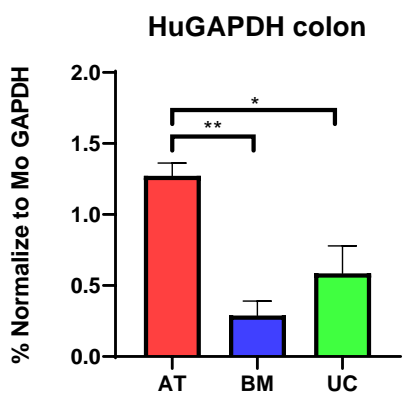

Supplement: Supplementary file 7 — Additional file 7: Fig. S3. A Fluorescence imaging of tissues 21 days after transplantation. Immunofluorescence images of the lung and liver, spleen and colon at 21 days after transplanting CM-Dil labeled hMSCs into mice. B Human specific GAPDH mRNA expression of mice liver and colon at day 21 compared with mouse GAPDH. Graphs show data of three experiments with mean +/− SEM. *p < 0.05, **p < 0.01. [file 13287_2023_3380_MOESM7_ESM.pdf]

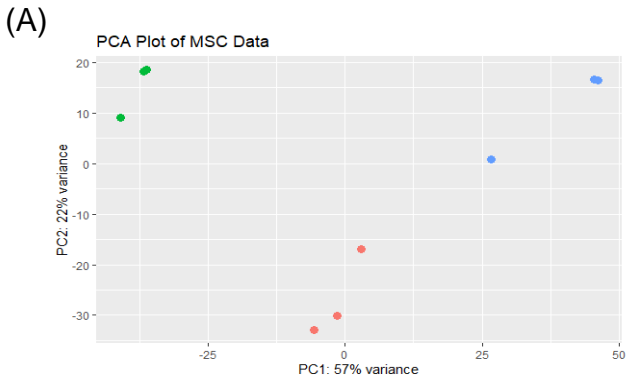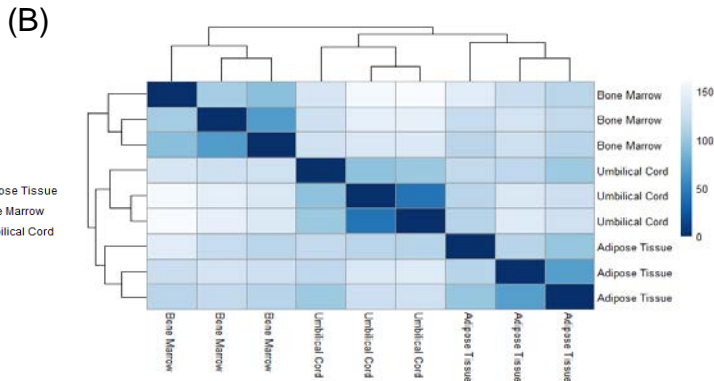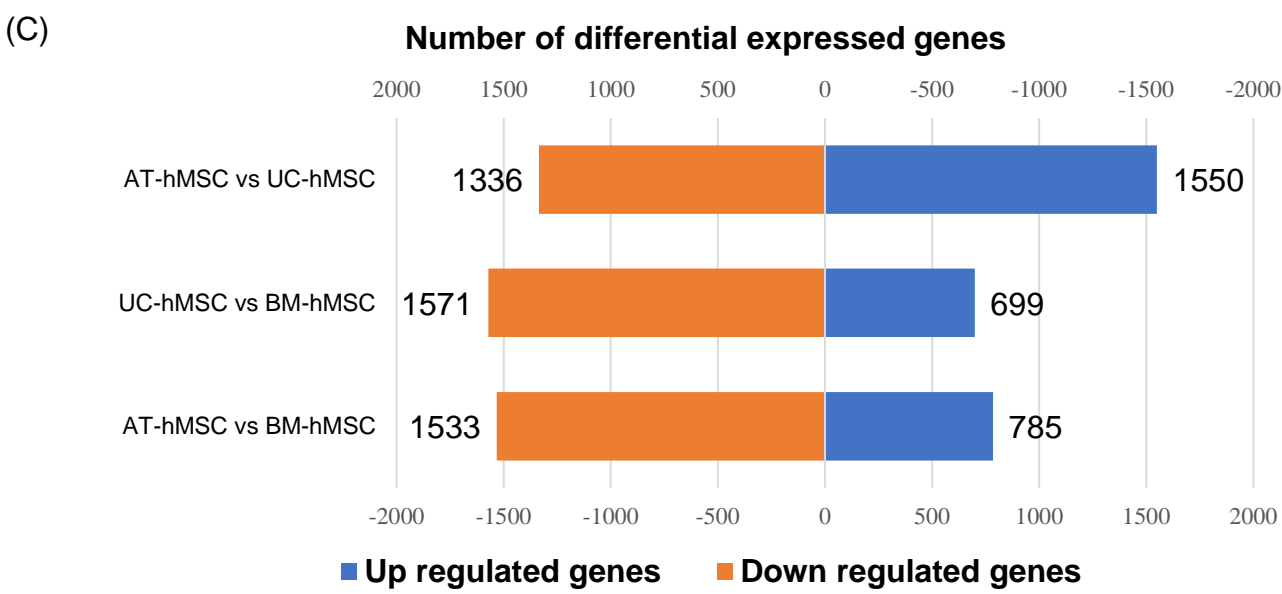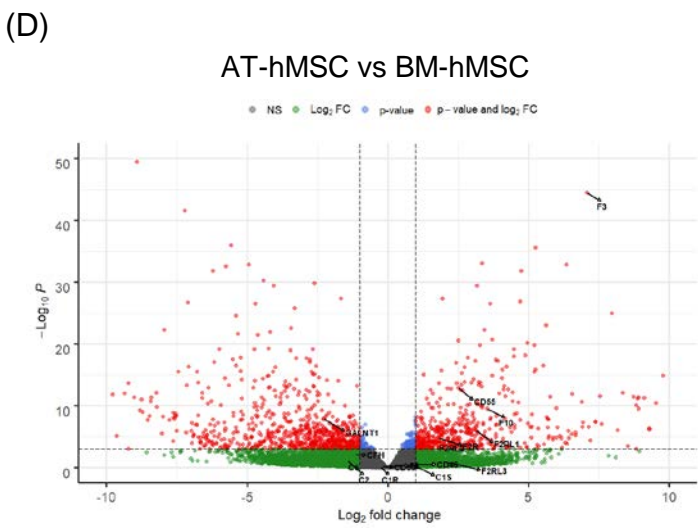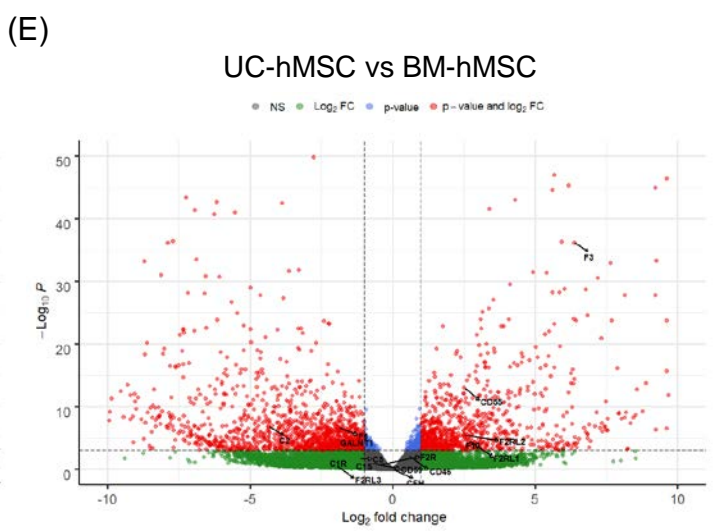

Supplement: Supplementary file 8 — Additional file 8: Fig. S4. A Principal component analysis. B Heatmap of sample-sample distancing using Ward’s method. C Number of up and down regulated genes in each comparison. Blue bars indicated the upregulated genes, whereas the orange bars indicated downregulated genes. D, E Volcano plot of AT-hMSCs versus BM-hMSCs and UC-hMSCs versus BM-hMSCs of our dataset. [file 13287_2023_3380_MOESM8_ESM.pdf]
