# Supplementary material for: Adipose tissue-derived human mesenchymal stromal cells can better suppress complement lysis, engraft and inhibit acute graft-versus-host disease in mice
Source: Stem Cell Res Ther. 2023 Jun 25;14:167. doi: 10.1186/s13287-023-03380-x (PMC10291819; doi:10.1186/s13287-023-03380-x)
Supplement: Supplementary file 5 — Additional file 5: Table S3. Mouse aGvHD clinical scoring system. [file 13287_2023_3380_MOESM5_ESM.pdf]

**Table S3. Mouse aGVHD clinical scoring system.**

| Parameter | Animal ID | Score |
|---|---|---|
| Weight loss | <10 % | 0 |
| | 10-25% | 1 |
| | >25% | 2 |
| Posture | Normal | 0 |
| | Hunching noted only at rest | 1 |
| | Severe hunching | 2 |
| Activity | Normal | 0 |
| | Mild to moderately decreased | 1 |
| | Stationary unless stimulated | 2 |
| Fur texture | Normal | 0 |
| | Normal Mild to moderate ruffling | 1 |
| | Severe ruffling/poor grooming stimulated | 2 |
| Skin integrity | Normal | 0 |
| | Scaling of paws/tail | 1 |
| | Obvious areas of denuded skin | 2 |
| | Total | 0 - 10 |
