# Supplementary material for: Adipose tissue-derived human mesenchymal stromal cells can better suppress complement lysis, engraft and inhibit acute graft-versus-host disease in mice
Source: Stem Cell Res Ther. 2023 Jun 25;14:167. doi: 10.1186/s13287-023-03380-x (PMC10291819; doi:10.1186/s13287-023-03380-x)
Supplement: Supplementary file 9 — Additional file 9: Table S4. List of Differentially expressed genes between AT-hMSCs and BM-hMSCs. Log2 FoldChange > 1 or < − 1, p-value < 0.05. [file 13287_2023_3380_MOESM9_ESM.pdf]

Table S4. List of Differentially expressed genes between AT-hMSCs and BM-hMSCs. Log2 FoldChange >1 or <-1, p-value <0.05

Gene name	log2FoldChange	pvalue	padj
HAND2	10.30741435	9.92E-16	2.49E-13
GSTM1	9.776060963	1.29E-15	3.16E-13
CACNG7	9.537558378	3.63E-11	4.14E-09
MMP3	9.298523034	7.44E-07	3.44E-05
CXCL6	9.287016967	4.24E-07	2.10E-05
TBX5-AS1	9.109096947	4.85E-12	6.16E-10
PAX3	8.926143956	0.002204361	0.027376166
FOXF1	8.919738353	4.38E-12	5.63E-10
CADPS	8.912193488	2.90E-10	2.88E-08
TBX5	8.873036726	5.88E-12	7.38E-10
IL1B	8.829566605	7.44E-11	8.05E-09
HOXB9	8.827517853	0.00099366	0.014712505
HAND2-AS1	8.736817092	3.38E-12	4.53E-10
IL33	8.528792564	0.000304345	0.005681102
CXCL5	8.358609649	7.04E-13	1.05E-10
PI16	8.165661913	7.89E-07	3.58E-05
RP11-92C4.6	8.109705945	0.021313525	0.140178651
VAT1L	7.975404656	1.00E-25	7.41E-23
ADGRF5	7.854804171	0.012246592	0.096509264
EPB41L3	7.679231882	2.97E-53	1.59E-49
TRIM58	7.607782201	7.93E-08	4.92E-06
DAB1	7.53628484	2.90E-12	3.92E-10
MMP1	7.389355128	3.95E-08	2.62E-06
CECR2	7.372351621	3.68E-08	2.47E-06
RNF165	7.200201221	3.77E-05	0.001030334
IL1A	7.149383773	2.40E-07	1.28E-05
F3	7.079783543	3.00E-45	9.13E-42
RP11-495P10.9	6.940776851	4.18E-08	2.75E-06
TATDN2P3	6.917502084	2.67E-07	1.41E-05
ANO4	6.893555158	2.42E-11	2.82E-09
TUNAR	6.685355529	5.25E-06	0.000181384
HIF3A	6.682133824	2.17E-06	8.38E-05
COL15A1	6.547624563	1.12E-12	1.60E-10
CNTN5	6.541681808	3.56E-06	0.000128462
RP11-401O9.4	6.435672353	1.59E-05	0.000478751
LINC01679	6.409450922	1.04E-06	4.54E-05
WNT2	6.375594394	0.000125764	0.002743666
NOMO3	6.363033446	1.42E-33	2.47E-30
LINC02086	6.320592935	9.33E-05	0.002154254
ZFP42	6.190485724	0.000610486	0.009973453
LINC01354	6.172988597	1.74E-05	0.000514621
CTA-384D8.31	6.168760022	0.001147002	0.016391449

FAM162B	6.112777845	6.93E-06	0.000231744
XPNPEP2	6.06276393	0.005706017	0.056128902
DAW1	6.028802158	3.01E-07	1.56E-05
BCHE	5.994543538	2.43E-10	2.42E-08
GDF10	5.990466682	1.26E-05	0.000388019
STXBP5L	5.869942501	2.92E-05	0.000824218
PCSK9	5.86148897	1.47E-05	0.000444357
RP11-255I10.1	5.817390782	0.024267876	0.152798561
RPL9P9	5.804731987	1.20E-72	2.56E-68
LCE2A	5.775528385	0.001782312	0.023244134
DPT	5.770990478	3.67E-12	4.86E-10
RP11-401O9.3	5.766468561	0.001129843	0.016178751
DMRT2	5.738973824	7.93E-06	0.000261373
MYH13	5.730611076	0.001685524	0.02222641
ELMOD1	5.718008243	1.73E-05	0.000512749
CLEC14A	5.699960727	1.46E-08	1.06E-06
GALNT13	5.693471669	1.23E-08	9.19E-07
RP11-109L13.1	5.693287773	3.27E-07	1.68E-05
RP11-514D23.3	5.656311914	0.001304009	0.018110931
LINC01197	5.642641991	0.004686211	0.048325275
FAIM2	5.638973401	6.20E-09	4.92E-07
SGCG	5.618749736	9.15E-24	6.30E-21
SLC13A5	5.564890921	0.037391342	0.202522753
RP11-429E11.2	5.549297535	0.000188945	0.00381033
MESTP3	5.539663821	0.001418353	0.019448024
CARD11	5.534354541	3.11E-17	1.02E-14
RP11-211G23.2	5.37415279	0.000346138	0.006312136
DCLK3	5.354520694	4.41E-07	2.17E-05
MYH2	5.307432743	0.001452272	0.019799149
SLC2A14	5.297857677	0.000192752	0.003872468
RP11-672A2.5	5.2879868	0.000882162	0.013415403
CCL11	5.257328519	0.0164292	0.117826356
CTA-503F6.2	5.254478688	0.002195319	0.027311561
PTGS1	5.250740723	2.52E-36	5.37E-33
SSTR1	5.236235339	1.23E-16	3.63E-14
SV2B	5.230588409	0.036682005	0.20016554
LAMP3	5.226365127	0.007481929	0.06764171
WNK4	5.209479265	1.60E-20	7.95E-18
TNXB	5.207177055	3.05E-16	8.23E-14
C8orf4	5.1991805	0.001141131	0.016318484
KLHDC7B	5.17258724	0.002190495	0.027283361
CACNG8	5.127594683	2.23E-07	1.21E-05
GABRA5	5.120485526	2.19E-09	1.93E-07
RP11-180C1.1	5.104156391	1.22E-05	0.000378055
TRIL	5.05786159	2.55E-07	1.36E-05
CACNA1H	5.039665863	0.002198992	0.027341312

IL1RN	5.024558989	0.01566672	0.114005843
DNER	4.990379157	7.07E-08	4.47E-06
PTPRB	4.960117236	6.67E-21	3.56E-18
SUPT20HL1	4.957500838	0.002923814	0.03390353
CXCL8	4.941991685	0.000454199	0.007840449
RP11-736N17.10	4.937565104	0.00051699	0.008699128
CCDC85A	4.890890712	7.24E-19	3.09E-16
LINC01561	4.862510977	0.000113593	0.002527243
TLX2	4.820111474	0.002694515	0.03183287
ADGRG6	4.737723778	1.33E-32	1.77E-29
PTPRN	4.705676763	1.23E-27	1.14E-24
INHBE	4.701123125	2.03E-08	1.43E-06
TNFRSF9	4.694369257	0.000625319	0.01016131
AC007743.1	4.636606643	2.84E-11	3.29E-09
PRKG2	4.62312715	0.018498229	0.127686255
RP11-324O2.3	4.611875813	0.004340298	0.045555663
HOXB-AS4	4.600048485	0.034509706	0.19244618
NUS1P2	4.59882396	0.014666257	0.109069106
TMEM74	4.597489935	0.005697314	0.05606914
C3orf80	4.57649484	1.51E-09	1.36E-07
RP11-554D15.1	4.567336306	0.012508768	0.097904282
FBXL21	4.559774373	0.005030282	0.050909845
CXCL3	4.557610836	0.007298931	0.066750958
IL32	4.507961081	3.37E-07	1.72E-05
HOXD11	4.507958305	0.001059079	0.015410912
ADAMTS16	4.505357423	1.36E-07	7.89E-06
TRPA1	4.500628321	2.55E-09	2.23E-07
FBLN2	4.481012098	9.74E-19	4.07E-16
CDH4	4.478309832	6.07E-20	2.88E-17
SHC3	4.444906769	6.42E-13	9.72E-11
SEMA3D	4.43763211	1.89E-11	2.27E-09
RN7SL101P	4.426976198	0.018566728	0.127999851
SIK1	4.408954071	0.019704745	0.133339815
PFN1P8	4.402184908	0.00780879	0.069710603
TM6SF1	4.389596581	1.71E-06	6.87E-05
TOX2	4.344810367	2.77E-07	1.46E-05
OR7E12P	4.329186204	0.003768496	0.040980955
FOXS1	4.320976725	0.000451923	0.007813807
CBSL	4.311185263	0.013171245	0.101364253
MAP3K21	4.30014422	0.000259904	0.004960028
IL13RA2	4.29662023	0.00232892	0.028491879
AC005042.2	4.2721429	0.034379179	0.192097385
FAM167A	4.268903989	2.35E-13	3.83E-11
RP11-49I11.3	4.262207206	0.00977915	0.081951275
RP11-123O22.1	4.251518166	0.000449317	0.007799289
ADGRD1	4.227942985	4.59E-17	1.44E-14

CXCL2	4.211121077	0.000293927	0.005530182
RP11-30K9.5	4.191679233	0.000626836	0.010178217
IFI27	4.187571861	2.52E-12	3.42E-10
MMP23B	4.156147268	0.006215692	0.059742307
RP11-184M15.1	4.150803606	1.10E-08	8.26E-07
AC156455.1	4.14555007	5.55E-14	1.03E-11
CH17-360D5.1	4.126627013	0.01788334	0.124937439
ASPN	4.122400585	2.20E-13	3.61E-11
RP4-536B24.4	4.122248887	0.016513861	0.118274502
ZNF280A	4.098358352	0.009105032	0.077674915
HOXB8	4.098052593	2.19E-05	0.000637778
SERINC2	4.084680859	1.38E-16	4.04E-14
AADAC	4.08309798	0.004224474	0.044664711
HTR1D	4.080402373	0.00670649	0.063035101
RPL21P133	4.077703634	0.033685299	0.189833473
MAB21L1	4.071530555	0.012986025	0.100510239
AC008063.2	4.068765484	6.43E-05	0.001583555
CTC-297N7.7	4.065306331	0.002493791	0.03000988
PCDH7	4.051901119	2.80E-15	6.56E-13
P2RX1	4.049939157	0.001283309	0.01791929
PLA2G3	4.035171793	0.026238955	0.160595052
TSSC2	4.034907888	0.048078572	0.237509704
MSC	4.031321692	5.43E-07	2.62E-05
MT1G	3.995934471	0.043512361	0.223382996
RP11-43F13.3	3.981438725	0.024336791	0.153036095
LINC01203	3.978379757	0.039164885	0.208853286
RP11-9M16.2	3.952879682	0.013033788	0.100756845
GPRC5B	3.951353622	9.44E-13	1.36E-10
RP11-308B5.2	3.944637265	0.004184648	0.044358695
KRT18	3.915647478	5.41E-18	1.96E-15
C5orf17	3.9113868	0.01940498	0.131823107
TRIM55	3.901428936	0.017779397	0.124711269
FUT1	3.900413507	0.021070688	0.139267669
FOXL2NB	3.889232061	0.040691183	0.213997311
ZNF467	3.887295588	0.00021614	0.004238566
COL6A6	3.86963687	0.018951222	0.129904091
PLEK2	3.867928907	1.10E-07	6.52E-06
LINC01504	3.847296875	3.95E-09	3.32E-07
ST6GAL2	3.844928932	4.09E-06	0.000145191
HS3ST3A1	3.834942047	3.80E-18	1.40E-15
FYB	3.834215664	1.21E-05	0.000376858
THBD	3.828584148	1.36E-07	7.89E-06
RP11-356C4.5	3.810768549	0.000289316	0.005453051
TDRD1	3.807933559	0.012796418	0.099607581
RP11-290O12.2	3.796278678	0.004584443	0.047484981
RP11-4B16.1	3.778855105	0.042612653	0.220606184

GPR158	3.77826379	0.000725929	0.011489931
NR5A2	3.749478757	0.000967302	0.014402196
KDR	3.738581371	0.036133411	0.198134786
CHRNA9	3.724751113	0.000906991	0.013705072
PMS2P2	3.724517226	0.006943874	0.064611638
SPON2	3.723635777	1.34E-15	3.24E-13
GRIN2D	3.704518478	1.79E-21	1.00E-18
CECR1	3.685036266	0.000231688	0.00449391
SLC6A15	3.674567193	2.96E-05	0.000833401
RP11-473E2.4	3.664602208	0.036526568	0.199674829
GPR4	3.654090604	0.000168649	0.003483337
RP11-566K19.5	3.647116761	0.043145416	0.222661311
S1PR5	3.645461009	8.00E-09	6.16E-07
STXBP2	3.643409725	1.40E-08	1.02E-06
FIBCD1	3.626553041	2.88E-27	2.36E-24
RP11-115N4.1	3.622655236	0.021598831	0.141620365
LINC01415	3.609004063	5.80E-07	2.77E-05
S100A4	3.597564803	0.004799653	0.049304471
LIPG	3.576846969	0.004088128	0.043633971
IRX1	3.570430395	0.000106337	0.002393255
RP11-310H4.6	3.555662707	0.002689525	0.031791532
ADAM19	3.551639681	2.44E-61	1.73E-57
F10	3.530686594	3.06E-10	3.02E-08
TRGV8	3.528250775	0.022978076	0.147668742
AC092168.2	3.525060329	0.03132404	0.180483314
GALNT12	3.523212962	2.31E-05	0.000668446
NETO1	3.521799822	0.031833378	0.182579828
HSPD1P6	3.508754665	0.005385481	0.053769127
GPAA1P1	3.50254267	0.030619494	0.178155858
BCAN	3.493121224	0.03095047	0.179007649
RP11-680G24.4	3.487539474	0.003972219	0.042609992
CH17-195P21.2	3.47361357	0.01199183	0.095137239
MFAP4	3.454859854	1.21E-06	5.17E-05
PTPRD	3.440166094	4.47E-23	2.89E-20
LINC00710	3.427635095	0.048768146	0.239474606
MYOM3	3.424584363	8.85E-06	0.000286957
PCDH1	3.41271407	4.93E-06	0.000170985
PCDHAC2	3.409811485	0.001396418	0.01916011
HSD17B14	3.399352824	6.13E-08	3.88E-06
TBX20	3.384814176	0.024883222	0.155028361
RP11-90C1.1	3.384390607	0.039518372	0.210160517
AC016999.2	3.383984797	9.81E-08	5.93E-06
MBP	3.361818556	8.88E-11	9.38E-09
UNC13A	3.360787951	0.000201815	0.004020475
ATE1-AS1	3.360369321	1.63E-05	0.000488852
RP11-563J2.2	3.356758346	0.040390611	0.212994086

ACVRL1	3.354163397	1.83E-10	1.86E-08
TWIST2	3.353890256	9.54E-34	1.85E-30
CTD-2589M5.5	3.350263601	0.005866608	0.057207473
PAPPA2	3.347061672	0.008272921	0.072608413
TMEM52	3.337988726	0.017855665	0.124907696
FAM196B	3.328094314	6.86E-14	1.24E-11
FAM86GP	3.32617968	0.000655017	0.010563455
MTND4P9	3.31134004	0.039111727	0.208726313
HILS1	3.307320152	0.000136165	0.002912093
EBI3	3.306290125	0.045298697	0.229189707
DPEP1	3.301123393	0.011501171	0.092460058
KANK4	3.300592307	1.98E-05	0.000581962
SYBU	3.296260139	7.11E-11	7.74E-09
PKDCC	3.285389075	1.87E-14	3.80E-12
RBP1	3.283014979	0.000199129	0.003981828
CLDN11	3.277626633	1.51E-20	7.66E-18
PAQR9	3.254744116	0.04633674	0.232394159
PTPRD-AS1	3.246633566	4.56E-05	0.001201422
CBS	3.235463918	3.80E-14	7.37E-12
LINC02057	3.232960326	0.014891014	0.110471029
TENM3	3.229318657	1.45E-14	3.07E-12
TIMM8AP1	3.228088576	0.00025448	0.004860867
SLC8A1-AS1	3.223043413	0.016239797	0.116941806
RAMP2-AS1	3.209923498	0.045006472	0.228035895
TNFSF4	3.209841125	5.47E-08	3.52E-06
RP11-481J13.1	3.20529677	0.000830571	0.012785761
FAM72C	3.203157028	0.037295928	0.202428369
RP11-655C2.3	3.196502584	0.016858946	0.120100994
HHIP	3.19493894	0.000531031	0.008893317
CSMD2	3.192090096	1.59E-06	6.47E-05
KCNG1	3.186481795	5.64E-11	6.24E-09
C4BPB	3.183114663	0.013014893	0.100647247
ATF5	3.174580436	3.18E-30	3.58E-27
NOVA1	3.169596338	0.000399178	0.007056229
ANXA8L1	3.168479166	2.27E-11	2.68E-09
PTPRH	3.1646413	4.05E-06	0.000144279
CHST6	3.149178713	2.29E-07	1.24E-05
F2RL1	3.132673851	9.13E-07	4.04E-05
RP11-1212A22.4	3.132614075	0.012170491	0.096102741
FAT3	3.131441605	1.61E-07	8.98E-06
LPXN	3.110898346	5.67E-18	2.02E-15
CLDN14	3.097714117	9.69E-06	0.000309826
OXTR	3.08060043	7.51E-07	3.45E-05
CDH10	3.078966226	1.30E-08	9.62E-07
EDA	3.075723538	0.00031227	0.005793564
RGS7BP	3.06990921	0.023359419	0.149140564

LY6K	3.064723458	0.005001419	0.050741925
MOV10L1	3.063614643	9.29E-05	0.002147075
RASGRP2	3.062946346	0.04999242	0.243119324
IRAK3	3.058900674	4.53E-07	2.22E-05
MMP8	3.058296564	0.001474655	0.020014788
ADAM33	3.055687774	4.36E-15	9.90E-13
FABP3	3.043400902	0.000135365	0.002902671
TEK	3.042938123	3.88E-08	2.59E-06
CH17-118O6.6	3.042802857	0.049040732	0.240591645
GSTM5	3.04219	4.20E-05	0.001127038
PLPP4	3.031207907	1.15E-09	1.07E-07
WTAPP1	3.026929783	0.02634626	0.160928657
MOXD1	3.015233976	3.29E-09	2.81E-07
SCD	3.014272537	5.63E-05	0.001425191
ADGRF4	3.013466128	0.007017598	0.064985884
RP11-893F2.5	3.010112954	4.45E-06	0.000156362
DLGAP1	3.006736842	0.001809083	0.023507065
TRBC2	2.992077466	0.044966562	0.228035895
CAPNS2	2.988428753	0.038759797	0.207366856
ST6GALNAC3	2.987581943	1.15E-06	4.93E-05
HEY1	2.981793767	0.000489713	0.00838342
SERPINB9P1	2.979923699	0.001477175	0.020036234
HNRNPA1P33	2.970339265	0.004013714	0.043011858
TMEM154	2.969840303	1.76E-14	3.62E-12
IL12A-AS1	2.965649055	0.025159285	0.15623401
TRIML2	2.955223226	1.81E-06	7.20E-05
SORCS2	2.954210926	4.98E-08	3.22E-06
TNFRSF6B	2.94683949	3.02E-05	0.00084606
CRLF1	2.939710441	4.47E-06	0.000156839
ADAP1	2.934960701	6.51E-05	0.001596804
FGF12	2.926143813	0.006241806	0.059840859
FOXL1	2.905552296	5.70E-11	6.27E-09
HTR1B	2.905206757	0.020287389	0.135647678
FAM129A	2.903455493	5.95E-07	2.82E-05
N4BP3	2.896638272	0.001496148	0.020190902
NOS3	2.89657935	2.73E-06	0.000101479
GPR150	2.885191161	0.000301519	0.005653091
NOC2LP1	2.884105827	0.0002016	0.004019952
CDH20	2.884047912	0.030887158	0.178789513
FLT1	2.880946305	0.000129792	0.002811423
ZNF804A	2.877695154	2.53E-06	9.50E-05
LINC00337	2.87736011	0.028754632	0.17069257
RP11-150O12.6	2.876013267	0.004706301	0.048509001
TINAGL1	2.874147784	6.13E-07	2.90E-05
GLI1	2.872488104	4.51E-07	2.21E-05
MYH15	2.869463163	0.000184028	0.003743012

KRT7	2.868137822	0.001178643	0.016787398
LCP1	2.867788029	0.000772679	0.012068724
LINC00517	2.859820847	0.017577239	0.123625903
GPAM	2.857313727	1.71E-18	6.84E-16
ADRA2A	2.855124362	0.003630016	0.0397996
GPR63	2.845044619	5.72E-05	0.001442432
TNFAIP6	2.844953862	0.000205403	0.004057841
TMEM51	2.84405941	2.33E-08	1.63E-06
RP11-262A16.1	2.839244859	0.000131596	0.002841832
USP43	2.836413327	0.002719772	0.032060255
PRR15	2.835706897	9.68E-08	5.88E-06
CDHR1	2.832747196	0.001000801	0.014780182
CDH2	2.829211886	8.21E-11	8.76E-09
GALNT6	2.82550311	1.58E-06	6.46E-05
RP11-353N4.6	2.819300849	0.042763297	0.221031196
RP11-522B15.3	2.813475765	0.000389079	0.006906308
NDUFA4L2	2.806843392	2.42E-06	9.19E-05
CYP2S1	2.801093076	0.015391298	0.112616164
HTR1F	2.799147616	0.020151922	0.135165485
UNC5B	2.798996242	3.14E-07	1.62E-05
PRLR	2.791532014	0.028337405	0.169026244
FERMT3	2.788049107	2.45E-09	2.15E-07
CAMK2A	2.782692522	3.59E-07	1.81E-05
LRRC15	2.779846253	0.034819346	0.193329044
RP11-98J23.2	2.7784958	0.013411474	0.102766191
TRIM14	2.777760374	8.37E-08	5.15E-06
SRGAP2D	2.776676602	4.90E-10	4.78E-08
MAB21L3	2.769342851	0.001294673	0.018042544
IL20RB	2.751341057	1.52E-06	6.25E-05
PRDM1	2.744469638	1.63E-08	1.18E-06
RP11-34P13.13	2.741632302	0.046415441	0.232469447
LINC01684	2.735233832	0.000113819	0.002529636
RP11-495P10.5	2.733473416	0.049129411	0.24069463
PTGIR	2.732022446	5.81E-17	1.75E-14
CTB-60B18.18	2.727341981	0.011814444	0.09423289
CALB2	2.722984154	1.50E-14	3.14E-12
CPNE7	2.701856799	1.33E-07	7.73E-06
RP11-49I11.2	2.698338811	0.022893691	0.147303918
LINC00623	2.694242031	0.028579796	0.169996801
C2orf88	2.683847829	2.75E-09	2.38E-07
BCL2A1	2.676986905	0.020089596	0.135024274
MYLK2	2.670474343	0.013298212	0.102024684
MN1	2.668321808	5.89E-09	4.72E-07
RP11-1430O6.1	2.665732609	0.000938875	0.014057436
NPR1	2.664844981	0.002018847	0.025563277
NTF4	2.662097582	0.000689988	0.011019156

MIR503HG	2.658132105	3.51E-13	5.50E-11
RASL11B	2.654825471	0.000953101	0.014226056
DNM1	2.643154116	1.25E-08	9.26E-07
PRICKLE2-AS1	2.642364009	0.036770939	0.200599528
OAF	2.635403103	4.62E-16	1.22E-13
ANGPTL1	2.630633416	0.000279737	0.005295215
SH3PXD2A	2.623771681	3.92E-08	2.60E-06
RP11-756P10.3	2.616963092	0.000218404	0.004275106
ZNF300P1	2.61563274	1.19E-06	5.09E-05
AOC3	2.615149504	0.00102573	0.015071117
TMEM145	2.60473926	0.021220416	0.139763916
MMP16	2.604104695	4.09E-14	7.85E-12
PTPRR	2.592595464	0.005762647	0.056421054
AC108142.1	2.591436501	1.04E-07	6.22E-06
LINC01969	2.588568246	0.00143858	0.019662738
AFAP1L1	2.588112719	2.42E-13	3.90E-11
SSC5D	2.579833507	1.39E-06	5.77E-05
GUCY1A2	2.576346381	1.93E-10	1.94E-08
CTSC	2.561020771	2.17E-05	0.00063259
FADS2	2.559233388	0.001239728	0.017459301
COL3A1	2.557949332	1.32E-09	1.20E-07
MSC-AS1	2.557006041	0.000203146	0.004031924
SERPINB7	2.555969646	0.003127794	0.03564883
AFAP1L2	2.555714931	0.015037033	0.11109077
RP11-82L18.2	2.554694936	0.000809873	0.012557746
ANXA10	2.550232937	7.60E-05	0.00182025
MAPK13	2.543086403	1.40E-07	8.06E-06
NLRP2	2.539402537	0.0027159	0.032050028
NR2F2	2.527373047	1.31E-08	9.63E-07
CNTN3	2.527044134	3.66E-05	0.001004945
KLHL30	2.523224556	0.009313477	0.078979466
HADHAP2	2.521059042	0.03861643	0.207062105
TYMP	2.518927251	1.20E-05	0.000374084
TMC6	2.518630707	0.023503743	0.149594976
TBX3	2.512557965	2.80E-21	1.53E-18
CD55	2.503082894	1.13E-13	1.94E-11
Mar-03	2.50022917	5.92E-08	3.77E-06
EML5	2.48619682	0.027720038	0.166367012
RP11-627G18.1	2.480562155	0.003781003	0.041019251
NOX4	2.471061523	2.33E-05	0.000675752
KRT8	2.470751609	0.000218715	0.00427728
ITM2A	2.466303917	0.031188791	0.180044387
KCNA7	2.464154751	0.00036889	0.006630705
KIF5A	2.463886116	3.04E-05	0.000850133
RBMS1P1	2.457319463	0.004903309	0.050112043
HAGLROS	2.443561099	0.033793679	0.190092787

CAMK4	2.440966908	2.06E-14	4.14E-12
KIF21B	2.434524965	0.000187818	0.00379478
LRRC8C	2.434102889	1.15E-05	0.000361976
UCP2	2.433359695	0.005289472	0.052959255
TMEM204	2.424507414	1.08E-17	3.65E-15
TBX1	2.42017336	0.007222502	0.066307789
BNC1	2.414539762	1.25E-06	5.30E-05
SFRP2	2.413098784	0.008038861	0.071080459
SLC38A5	2.404603925	2.08E-06	8.12E-05
ANXA3	2.404142248	7.17E-08	4.52E-06
CTB-12A17.2	2.403350859	0.01393883	0.105273936
HOXD10	2.400694407	0.006629466	0.062448691
RP11-20I20.4	2.398677498	0.000119328	0.002624727
MOB3B	2.398617228	0.029752099	0.174825336
GFPT2	2.396325711	1.44E-06	5.97E-05
DPP4	2.392629678	0.000219306	0.004284899
OLR1	2.39013342	0.010881511	0.08844492
HMSD	2.38991224	0.000451516	0.007813807
RP11-1E6.1	2.388962808	0.045864743	0.230958261
CH17-13I23.3	2.388304543	1.68E-06	6.82E-05
FKBP9P1	2.386390398	0.000462579	0.007959335
ERICH2	2.382521038	0.000805294	0.012523139
CCL20	2.377648222	0.004777801	0.049182731
RP11-3L21.2	2.358854019	0.032213816	0.18436534
CGB7	2.335095321	0.001301575	0.018091465
ADAMTS4	2.334716793	4.74E-11	5.32E-09
EDNRB	2.333924699	0.004243693	0.044823482
GS1-57L11.1	2.332309169	0.029734129	0.174816028
LAMA2	2.331174248	6.55E-06	0.00022012
SCRG1	2.330680374	0.000151879	0.003198898
ABCC9	2.330291815	4.67E-05	0.001223043
SYNPO2L	2.329519979	0.014195988	0.106747409
FAM105A	2.328187532	0.013227523	0.101665137
PLPP2	2.327768295	5.87E-05	0.001471111
ITGA2	2.327446555	2.61E-05	0.000745824
IL15RA	2.326689804	2.80E-05	0.000791154
RP11-673E1.3	2.319405942	0.000450299	0.007804688
FBN2	2.317815946	1.52E-07	8.61E-06
RP11-676J12.7	2.309205642	1.17E-06	5.02E-05
GYPE	2.302460558	7.27E-07	3.37E-05
RCOR2	2.301167724	0.00050072	0.008512641
ATP2A3	2.301092705	0.001550631	0.020755493
SCN9A	2.292098662	9.12E-05	0.002114425
U52111.14	2.292086308	5.46E-07	2.63E-05
TRIM7	2.29080927	0.012039698	0.095423107
MAMDC2	2.287050817	0.014685349	0.109135005

FMNL1	2.285845737	0.01437488	0.107577144
AC007405.6	2.285283617	0.002166937	0.027110495
RSPO4	2.279813284	0.006088391	0.058958317
TRIB3	2.277985711	0.007975742	0.070727526
RP11-121A8.1	2.275126661	0.016531065	0.118278604
ARAP3	2.267314377	2.41E-06	9.17E-05
B3GNT8	2.263708009	3.38E-06	0.000122782
STAB1	2.260313056	0.00413068	0.04397814
COL16A1	2.258636316	8.71E-05	0.002027673
TNNT1	2.250747145	0.016624821	0.118750315
MISP	2.24504795	0.02113288	0.139379019
CTD-2650P22.2	2.239414333	0.023575484	0.149831906
KIAA1324L	2.237407916	1.86E-13	3.08E-11
ZBED9	2.229960821	0.003394117	0.037994169
U82695.9	2.226518598	0.031812668	0.182510106
NPAS2	2.222031426	0.000310366	0.005768263
CD70	2.221403527	0.016608601	0.11871394
SDPR	2.217331201	1.05E-06	4.55E-05
E2F7	2.201503715	0.001070913	0.015554121
TSKS	2.192892521	0.041611911	0.217258322
C16orf74	2.192705619	5.73E-05	0.001442432
PPP1R13L	2.182522545	3.34E-08	2.27E-06
RP11-214O1.2	2.182045077	0.004552354	0.047271671
COL13A1	2.178637652	0.00407192	0.043504505
GATA2	2.177153705	0.000108453	0.002428072
ABCC2	2.175286117	7.10E-05	0.001724646
RBMS3-AS3	2.171716392	0.000603988	0.009882431
PCDH9	2.170520493	1.44E-05	0.000436763
INSIG1	2.168625982	0.004573139	0.047434368
MIR503	2.161599398	0.020778687	0.137908627
LRRC17	2.160582633	0.002308756	0.02832641
ANXA8	2.159824145	0.014950053	0.110754975
HTR2A	2.148735598	0.002064257	0.026045524
GAS7	2.147179483	0.000160377	0.00335142
ALDH1A2	2.146525041	0.042019868	0.218618797
UPK3BL	2.146334873	6.27E-11	6.86E-09
NUAK1	2.14550361	1.91E-05	0.000563073
RP11-497E19.1	2.140663557	4.27E-05	0.001144616
GAB3	2.138035576	8.35E-08	5.15E-06
AC144831.3	2.131156859	1.79E-08	1.28E-06
RFLNB	2.125400733	1.35E-13	2.26E-11
OLFM1	2.122321667	7.77E-07	3.55E-05
CH17-472G23.2	2.11591138	0.005658435	0.055866895
NXPH4	2.111419685	0.004532515	0.047127559
FASN	2.109475249	0.001896419	0.024458576
ADAMTS12	2.109060794	0.003007812	0.034614174

POU2F2	2.104453884	0.009586114	0.080777775
NR2F2-AS1	2.102169024	1.89E-05	0.000558766
RPSAP61	2.101478382	0.025933568	0.159365961
LINC00862	2.093519059	0.044717839	0.227545867
RP11-989F5.1	2.088713641	0.047556127	0.235735275
ETV4	2.075825108	1.32E-07	7.70E-06
DOCK11	2.073940876	1.99E-06	7.82E-05
MSMO1	2.06887313	0.003604928	0.039647753
MOCOS	2.062333342	1.41E-07	8.07E-06
GS1-393G12.14	2.056866666	0.042024319	0.218618797
RAB39B	2.045561282	0.004020748	0.043043989
HS3ST3B1	2.041494599	0.000209375	0.004121054
G0S2	2.032881258	0.010339306	0.085173524
HMGCS1	2.031155843	0.004874014	0.049876244
FRMPD4	2.021720711	0.000719397	0.011403463
VWF	2.020166107	5.29E-05	0.001354162
RP4-794I6.4	2.013724594	0.003596613	0.039596151
FAM87A	2.012179249	0.026906491	0.163246608
FLRT2	2.007564928	7.90E-05	0.001870419
AC106786.1	2.007068894	0.000164587	0.003415981
PCK2	1.999690375	1.69E-06	6.84E-05
COLEC11	1.986915967	0.046513135	0.232522083
CHN2	1.981050903	0.007090881	0.06549396
FRMPD1	1.979900238	0.04629039	0.232394159
TRHDE	1.979216901	0.003542558	0.039183004
RAMP2	1.978616599	0.044688609	0.227451376
SC22CB-1E7.1	1.978558412	0.039477887	0.210154737
CCDC8	1.978156273	0.000403246	0.007116334
GATA6	1.97798355	0.000599364	0.009817263
SPHK1	1.973495621	1.12E-07	6.61E-06
CDH13	1.973342199	2.81E-06	0.000104282
STRA6	1.96734961	0.005508118	0.054712661
RP11-13N13.6	1.96596865	0.028450109	0.169388446
CTH	1.959129615	0.000100473	0.002290258
GCH1	1.95834883	0.000233609	0.004521906
GIPC3	1.958265271	0.0495662	0.242111822
ACAT2	1.95760496	0.001517616	0.020403187
VLDLR	1.956239918	6.79E-07	3.18E-05
SLCO5A1	1.951007178	0.014429361	0.107774498
RGAG1	1.946757071	5.04E-07	2.46E-05
FLNC	1.944228212	0.001485407	0.020096795
CH17-472G23.1	1.940162358	0.013760559	0.10437088
TYRP1	1.937006972	0.023180648	0.148352223
BCAT1	1.936248409	4.83E-28	4.68E-25
TSHZ2	1.933248328	0.004632008	0.047905244
AC009505.2	1.932265585	0.019840405	0.133967103

ULBP1	1.930883398	1.15E-05	0.000361842
WBP1LP2	1.930516256	0.015143218	0.111325683
AP003068.23	1.925397731	2.89E-07	1.51E-05
TCF21	1.924970387	0.002567293	0.030652353
FGF2	1.922669715	2.51E-06	9.45E-05
HAGLR	1.921930956	0.013044846	0.100805808
AC015933.2	1.919566356	0.024641455	0.154144313
KIF26B	1.918826049	0.008866875	0.076222262
MYEOV	1.918366341	0.029630523	0.17439913
C9orf47	1.913945235	0.033349186	0.188787008
DHCR7	1.910911437	0.010802277	0.088002059
CNKSR1	1.908978097	0.008893436	0.076416654
COL1A1	1.908832879	5.62E-06	0.000192132
KSR1	1.905048466	2.51E-06	9.46E-05
XRRA1	1.903780607	2.05E-16	5.90E-14
ZC3H12A	1.888565288	0.012249114	0.096509264
SLC7A5	1.88661914	4.47E-08	2.93E-06
SLC22A15	1.886531276	5.51E-06	0.000188814
GFOD1	1.885836362	6.91E-07	3.22E-05
HMGCR	1.884444581	0.001226482	0.017313903
MTUS2	1.881547781	0.037398746	0.202522753
DKK1	1.878793454	0.006875164	0.064196276
STX11	1.874412066	0.001449577	0.019787165
DBH	1.873074354	0.02019831	0.135375493
BEAN1	1.872373218	0.000100192	0.002287061
ABCA9	1.868885926	0.035169427	0.194652392
SGK1	1.866206036	6.15E-12	7.63E-10
SCG5	1.858448851	6.31E-05	0.00156333
ARHGAP26	1.853210664	0.008268546	0.072599874
HPDL	1.852469354	0.006153934	0.059364511
ITGA4	1.850190809	2.04E-07	1.11E-05
RBM24	1.847836005	0.007511491	0.067780102
RAB33A	1.846315318	6.49E-05	0.001592934
SUPT16HP1	1.842569094	0.012923188	0.100228696
HOXA11	1.834781022	1.30E-09	1.18E-07
PTHLH	1.827385564	0.002219052	0.0274627
SLC7A5P1	1.827177977	0.049438939	0.241711547
ERN1	1.825078767	3.76E-05	0.001029127
TCF4	1.823617847	1.11E-10	1.16E-08
SLC1A2	1.820914001	0.000471578	0.008107646
SLC4A11	1.819996116	0.000429072	0.007509986
ERBB3	1.817531497	0.011869412	0.094600589
SEL1L3	1.817307145	2.14E-14	4.27E-12
LRP8	1.812640797	7.46E-07	3.44E-05
SLC35F2	1.81080179	6.68E-06	0.000223733
COL5A1	1.810620004	0.000126326	0.002753106

ETS2	1.808443322	1.19E-06	5.09E-05
F2RL2	1.808326366	0.000886704	0.013457052
MYOCD	1.807991106	0.001909786	0.024590944
CPAMD8	1.807764319	0.030885286	0.178789513
EPHB1	1.807005789	0.0061016	0.059040242
RDH10	1.805189467	0.001851782	0.023988835
MILR1	1.805163803	2.03E-06	7.96E-05
SLC39A8	1.799261753	0.000744576	0.011681085
MFAP3L	1.79655044	1.41E-06	5.87E-05
EMILIN2	1.795049195	0.005535845	0.054908014
ARHGAP20	1.789217858	1.37E-05	0.000418642
HIC1	1.786472553	6.37E-07	3.01E-05
GREM1	1.781419199	0.021040078	0.139197242
F2R	1.781010843	1.42E-05	0.000431431
PLCL2	1.76760559	3.82E-07	1.91E-05
RP11-359E10.1	1.763939724	0.016844432	0.120067683
STXBP6	1.759958259	0.01121739	0.090622578
EIF4A1P10	1.759269446	0.007557884	0.068126326
RP11-758N13.1	1.756616002	0.00609037	0.058958317
TFPI2	1.754122938	0.023116403	0.148156077
B3GNT5	1.752730031	2.46E-07	1.31E-05
EBF2	1.751700752	0.017320951	0.122330288
ABCA1	1.748328292	0.000241119	0.004647252
CHAC1	1.748165029	5.64E-05	0.001425191
TRNP1	1.746709569	0.003023439	0.034719098
KCNJ10	1.743040873	0.049093117	0.240637166
AC144831.1	1.739037119	2.97E-11	3.43E-09
IGF2BP1	1.738992062	0.003160256	0.035903734
MFSD7	1.736209949	2.39E-07	1.28E-05
ADGRA2	1.732398355	9.98E-05	0.002281473
LINC00941	1.73009102	6.30E-05	0.00156333
CARD9	1.728398926	1.05E-06	4.55E-05
LUZP2	1.727208153	0.03303666	0.187785974
DDIT4	1.71999369	0.009280705	0.078736944
CACNA2D4	1.715500019	0.02966277	0.174501422
KCNIP3	1.712509573	8.27E-05	0.001946578
CCIN	1.708083407	0.007348266	0.067020101
CLGN	1.707203796	0.006588386	0.062151644
IL1RAP	1.702173884	1.91E-06	7.56E-05
SOX30	1.693175145	0.02297386	0.147668742
PTGS2	1.690721105	2.46E-05	0.000708965
MVD	1.688010843	0.01166812	0.093344957
FCRLB	1.687907425	0.020398993	0.1360528
SOD3	1.686800432	0.038493401	0.206750785
PCED1B	1.685253299	3.55E-05	0.00097771
CCZ1	1.684203182	2.92E-08	2.00E-06

MICAL2	1.666640487	8.79E-07	3.94E-05
ODF3B	1.661996604	0.022818597	0.146973328
ARSI	1.660722147	7.06E-05	0.001718633
RAET1K	1.65849095	0.030569398	0.178009163
CRABP2	1.655365244	0.044832065	0.227692677
WNT3	1.653682643	0.000166845	0.003457679
TRHDE-AS1	1.651917167	0.003912775	0.042197805
FIBIN	1.651544831	0.022821646	0.146973328
PTPRF	1.644685882	7.70E-07	3.53E-05
TMEM97	1.641801098	0.001110845	0.015960255
MME	1.64061672	0.028508156	0.169617963
DUX4L50	1.638404706	0.000169839	0.003501138
COL7A1	1.637757839	0.012421761	0.097437755
NSFP1	1.637154389	0.002677735	0.031704853
RP11-266K4.9	1.634055494	0.00289034	0.033570108
RP11-177C12.1	1.631849604	0.020563607	0.136765933
PID1	1.623747836	9.09E-06	0.000293806
PAMR1	1.619998352	0.00353712	0.039164895
ABC7-42404400C24.1	1.61360282	7.32E-05	0.001768996
ADM2	1.613170191	0.014411667	0.107774498
FBLN5	1.60526138	4.31E-05	0.00115486
RP11-649A18.12	1.60314995	0.025010332	0.155496749
FADS1	1.602278663	0.038112393	0.205137745
FILIP1L	1.602024638	7.19E-05	0.001743745
BHLHE40	1.600581636	0.002840458	0.033171331
MANBA	1.599606602	0.000751367	0.011778958
GLI2	1.598296916	9.24E-13	1.34E-10
SLC7A1	1.598161215	2.17E-10	2.18E-08
ROBO4	1.59505363	0.000697785	0.011127005
NCR3LG1	1.59464365	0.000606838	0.009921454
RNF157	1.594643482	0.009840354	0.082125024
HSPB6	1.593267168	5.48E-05	0.001398898
AGRN	1.593180823	0.001200009	0.017000926
SDC1	1.591884896	0.001561549	0.020872027
TCEAL7	1.591507427	9.53E-06	0.00030674
HOXA11-AS	1.589957148	0.001594068	0.02121711
BAIAP2L2	1.588320673	0.008072706	0.0713206
RBM47	1.583171037	0.002262463	0.027854536
RHOU	1.582382226	0.000913081	0.013766804
DHCR24	1.581704514	0.008706136	0.07532608
ADGRL4	1.57790966	7.51E-09	5.80E-07
CERKL	1.577649718	0.000984595	0.014608699
GIPC2	1.577198321	0.037790327	0.20386711
SERPINB9	1.568747409	0.006079334	0.058931696
IDI1	1.566354842	0.010652683	0.087182834
RP11-359P5.1	1.565288685	0.023387051	0.149140564

RPL9P7	1.561458349	0.045000621	0.228035895
SLC12A8	1.561210237	0.007296386	0.066750958
GATA6-AS1	1.561185818	0.005603936	0.055457128
MSX1	1.556654827	4.37E-07	2.16E-05
LINC00702	1.552538863	0.006762749	0.063396314
HHIP-AS1	1.55253451	0.016310885	0.117253724
IRS2	1.551954048	0.040550085	0.213413079
RASA4B	1.550867953	5.29E-05	0.001354162
SPRY4	1.541157686	0.000245338	0.004711557
SLC38A1	1.536444335	1.73E-10	1.77E-08
RP11-48O20.5	1.531293995	0.023388842	0.149140564
VEPH1	1.529648458	3.47E-07	1.76E-05
PBX1	1.529064294	0.001725892	0.022702607
ACSS2	1.52332149	0.017961942	0.125278859
MEIS3	1.518767038	2.65E-08	1.84E-06
OSCAR	1.517467269	0.017818904	0.124781814
HOXC8	1.516549783	3.56E-05	0.000980555
LINC01638	1.510718329	0.010111354	0.083813462
PRICKLE2	1.510559867	2.88E-13	4.58E-11
MXRA5	1.507814738	0.000803206	0.012499788
RP11-416N2.4	1.505725625	0.007432663	0.067463029
FJX1	1.505088728	7.73E-05	0.001839087
CORO2A	1.502803319	0.000311143	0.005777672
TNIK	1.490600557	8.70E-05	0.002027673
SEMA7A	1.490583058	0.036883992	0.200908053
PPARGC1B	1.489948041	0.009874829	0.082340749
SLIT2	1.488614958	1.39E-07	8.01E-06
SLC25A23	1.486043243	0.006963686	0.064739521
IQGAP2	1.485344583	0.026674722	0.162238277
FOXP2	1.484961215	0.023816949	0.1509232
AC144652.1	1.484909732	0.004205999	0.044517129
NHLRC4	1.484639803	0.02710916	0.164052291
NID1	1.4838524	5.93E-06	0.000201985
BGN	1.483758145	0.010821397	0.088090549
MKNK2	1.482564308	0.001226969	0.017313903
PRPH2	1.482186709	0.048136106	0.237628866
NGF	1.480895307	0.002789526	0.032701824
AP000695.4	1.478297712	0.000436692	0.007624599
CNIH3	1.478250117	5.43E-05	0.001388445
HIST2H3PS2	1.47667036	0.016494719	0.118177073
FDPS	1.475590688	0.014431258	0.107774498
MBOAT2	1.474282346	5.61E-05	0.001421893
SLC37A2	1.471673785	0.036287117	0.198758827
AKNA	1.4690932	0.002959711	0.034178262
ST3GAL1	1.465779654	3.78E-11	4.29E-09
SMG1P3	1.46301771	5.10E-05	0.001314979

PSAT1	1.462997677	0.000502809	0.008534553
RHOJ	1.46207321	0.006442519	0.061228322
SIGIRR	1.460035915	1.62E-06	6.57E-05
SPOCK1	1.459907128	0.000940204	0.01406745
SCARB1	1.4598028	0.025552671	0.157851601
TCHP	1.4562405	9.93E-06	0.000316689
AHNAK2	1.454769795	0.009281187	0.078736944
WARS	1.454272062	0.002470822	0.029783879
FAM72B	1.453541431	0.047847792	0.236586267
MATN3	1.450016042	0.008330499	0.07297269
RPP25	1.449849331	0.000320428	0.005924303
SH2D5	1.447555755	0.007525056	0.067859089
BEX4	1.446420286	0.039038466	0.208439616
NRP2	1.441241746	0.007495587	0.067736485
CNKSR2	1.440360256	0.008565993	0.074415319
SQLE	1.439815365	0.013864607	0.104973474
LIN9	1.438884297	0.018819882	0.129154392
SIRPA	1.437845097	6.53E-05	0.00159991
RP11-1069G10.1	1.436944665	0.040995735	0.215015977
CEP170	1.436051914	1.47E-05	0.000444641
ASNS	1.435392757	6.30E-06	0.000212371
ARAP2	1.433902728	0.036339984	0.198879577
ZSWIM4	1.432632531	0.028641101	0.170313971
S1PR2	1.432230354	1.54E-05	0.000465387
THBS2	1.431680214	0.036343807	0.198879577
SLC1A4	1.426579243	6.60E-05	0.001614091
LIPA	1.425215363	4.08E-06	0.000145147
RP11-500C11.3	1.418843231	0.001009051	0.014888545
CTD-2012J19.3	1.412298865	0.006204946	0.059699579
ERFE	1.409778247	0.000560995	0.009292997
HIST2H2BB	1.405716545	0.005037059	0.050909845
MFAP5	1.404361914	0.044732366	0.227565514
STARD4	1.404129127	0.000359916	0.006507773
PTER	1.401568179	0.000221065	0.004315318
MFAP2	1.397119371	0.008475964	0.073873841
SLC7A7	1.396747243	0.027142135	0.164052291
SNORD17	1.39547734	0.02199645	0.143258931
MLXIP	1.393914456	0.000483251	0.008288299
PHGDH	1.393818303	0.000416733	0.007324068
LINC01140	1.393319578	0.048334015	0.237945213
CEP170P1	1.393092804	0.0026676	0.031619955
AMZ1	1.389850372	0.01757789	0.123625903
ROBO1	1.38816859	0.000368537	0.006629933
RP11-342D11.2	1.383932876	0.020330203	0.135806266
COL5A2	1.379026757	5.64E-05	0.001425191
DACT3	1.368447816	0.001216348	0.01719814

COLGALT1	1.368352015	5.83E-12	7.36E-10
LRP4	1.366814195	0.001597792	0.021253423
ALDOC	1.364761995	0.013488814	0.103079275
RP11-527N22.2	1.363343558	0.008685873	0.075229848
INHBA	1.362639004	0.000189511	0.003818143
RNF152	1.358809706	0.033206821	0.188330873
TNS1	1.353522185	1.98E-06	7.80E-05
MICALCL	1.353130475	0.033502176	0.189101173
APCDD1L-AS1	1.35210864	0.011414373	0.091900779
SUPT3H	1.351851401	8.29E-06	0.00027008
RASSF5	1.350455017	0.002610999	0.031121941
RNA5SP283	1.344588171	0.021716392	0.142085537
MTHFD2	1.344252384	0.000111999	0.002496985
FZD8	1.342626918	3.92E-07	1.96E-05
RP11-303E16.2	1.341829753	0.000909389	0.013731583
PHACTR1	1.341431392	0.030687344	0.17835608
GDNF	1.340986807	0.00050195	0.008526755
COL4A1	1.340165921	0.014440074	0.107774498
Sep-06	1.334160397	0.001669324	0.022053682
TGFBI	1.331337804	0.010963542	0.088942254
ATP1B1	1.330079841	0.002209111	0.027403248
OSBPL8	1.321827402	5.50E-05	0.001401591
AC004057.1	1.317510408	0.008290035	0.072698801
RAP1GAP	1.317333817	0.029403145	0.173415603
CUBN	1.315519556	0.007448914	0.067526626
DACT1	1.313489003	0.001016896	0.014960156
KLHL29	1.313046053	6.64E-07	3.11E-05
SLC19A2	1.310180145	0.001281324	0.017903291
DEF6	1.309452167	0.031271181	0.180373589
FAP	1.309411914	0.029417014	0.173429515
GPR3	1.305906031	0.005852831	0.057099221
FRG1BP	1.303215539	0.011698573	0.093503899
PRDM6	1.303196902	0.011939645	0.094908057
OTUD1	1.296330613	0.015241171	0.111866418
AMPD3	1.295921183	0.03091723	0.178863886
SYTL3	1.293707182	0.040141673	0.212214849
TPBG	1.293309505	1.04E-07	6.23E-06
MT2A	1.290380818	0.027279614	0.164603461
EBP	1.289916245	0.001838919	0.02383668
WASH3P	1.288201516	0.000564879	0.009342838
NUDCD1	1.286744091	1.68E-05	0.000500991
TMTC2	1.286514965	2.67E-05	0.000756433
BEND3P1	1.284945163	0.041698711	0.217579774
ITPR2	1.281304651	0.013537885	0.103306262
TMEM54	1.27934685	2.41E-06	9.17E-05
ITGB3	1.27919107	0.000176766	0.003629916

RP11-100E13.1	1.275555129	0.011994712	0.095137239
UCK2	1.275449489	9.54E-16	2.42E-13
SGCD	1.273060101	0.000305621	0.005699944
UCHL1	1.271797162	0.000156699	0.003290679
SESN2	1.268119916	0.0121437	0.095962214
GSTT2B	1.26710643	0.035540041	0.195989228
MT1E	1.265419277	0.000915649	0.013777348
MGST1	1.265381763	2.13E-05	0.000621872
SV2A	1.2646374	0.037388873	0.202522753
SPARC	1.263131375	0.000194569	0.00389973
LINC01605	1.262818937	0.003549202	0.039236155
THOC3	1.262380847	7.60E-05	0.00182025
LMO4	1.261977196	1.38E-06	5.76E-05
NRXN3	1.259572957	0.0180987	0.125906051
PSD4	1.259538088	0.020144719	0.13515966
RP11-819C21.1	1.257687392	0.038970526	0.208209173
EIF4EBP1	1.256653819	0.00030945	0.005761276
GTF2H2B	1.25625298	0.00667471	0.062764044
MB21D2	1.255915908	0.00036211	0.006541898
KCTD14	1.251391671	0.020561953	0.136765933
LOXL2	1.249584994	0.000118513	0.002609495
PELI1	1.248975507	0.004773431	0.049177175
C17orf51	1.248266181	3.64E-05	0.001001517
COBLL1	1.24215254	0.000784291	0.01223219
ZNF860	1.236708091	0.003886129	0.041960748
SLC1A5	1.236687978	7.97E-05	0.001883054
RP11-46C24.7	1.236010454	0.032036932	0.183566324
NMNAT2	1.231463586	0.024459929	0.15341153
PTPRJ	1.2269486	5.93E-05	0.001482254
BMPR1B	1.226813513	0.012507597	0.097904282
GPSM3	1.22640514	0.002878296	0.033490649
A1BG	1.221677998	0.005536374	0.054908014
EDA2R	1.220884439	0.014979115	0.110816363
PRRT1	1.216546057	0.028832814	0.171024998
RP11-119F19.5	1.215677289	0.025442608	0.157574307
NCALD	1.213424135	0.046557949	0.232655976
RALA	1.213266241	9.23E-09	7.03E-07
AKT3	1.212337831	4.71E-08	3.08E-06
MBNL2	1.212008347	7.14E-06	0.000237626
CGNL1	1.209151304	0.023683527	0.150256241
UQCRFS1P1	1.206287836	0.006497556	0.061504813
CYP51A1	1.205434529	0.018666718	0.128433758
CNTF	1.205130532	0.019762252	0.133644185
XPOT	1.204563086	0.000115783	0.002560586
SARDH	1.203355197	0.01426598	0.107062592
MARS	1.199897069	1.43E-06	5.93E-05

COL1A2	1.19757111	0.000184993	0.003751911
TMEM205	1.196663511	0.008097083	0.071450966
ADAM12	1.190794983	0.006850283	0.064048042
ARHGEF26	1.18915962	0.002400302	0.029147892
HOXD9	1.188379314	0.000150431	0.003171545
KCNJ8	1.188057206	0.043475831	0.223356691
TMPO	1.187047476	0.02259609	0.146049732
FAM102B	1.1862483	0.000983013	0.014595389
LOXL3	1.18527533	0.001075586	0.015579561
RALGAPA1P1	1.184599457	0.026506861	0.161539673
CTTNBP2	1.184257883	0.046329871	0.232394159
UBL3	1.181385526	7.17E-09	5.58E-07
MED12L	1.181249982	0.017294298	0.122182497
SLC16A1	1.179995239	1.70E-08	1.22E-06
DOK6	1.17889207	0.000349626	0.006359441
UPP1	1.178863058	0.003620648	0.039728136
DNM3	1.178278974	0.024445438	0.153402312
AMMECR1	1.17760058	8.90E-07	3.98E-05
AP001189.4	1.174753672	0.02580028	0.158837704
TENM4	1.174317923	0.016408313	0.117755727
MEDAG	1.172629067	0.007575151	0.068192908
C4orf32	1.170614706	0.000178552	0.003645546
SIRPB1	1.169602913	0.039619981	0.210454387
LIMS1	1.165757611	8.88E-10	8.36E-08
RRN3P2	1.163572934	0.006393447	0.060900295
SPEN	1.161764906	9.56E-05	0.00220187
FSTL3	1.160647643	0.000326245	0.006016208
GPAT2	1.160203383	0.044214936	0.226119336
CCL26	1.158839038	0.04993721	0.243119324
RMND5A	1.15704786	0.000641529	0.010377298
GOT1	1.156359526	1.42E-05	0.000431431
ISLR	1.155817479	2.49E-05	0.000715335
SKIL	1.155085343	0.002683783	0.031758848
LINC01023	1.153901315	0.018461741	0.127558198
FLVCR1-AS1	1.152612497	0.012125088	0.095921721
GK	1.152553035	4.45E-05	0.001183483
RPS28P7	1.150233988	0.035437827	0.195577206
NDRG4	1.148113183	0.00569482	0.05606914
CDC42EP2	1.145698553	0.003699891	0.040358318
CAMKK1	1.144681733	0.003140016	0.035749942
DAAM1	1.137999709	0.002960322	0.034178262
KHDRBS3	1.137279373	0.000412597	0.007269334
PDGFA	1.13637294	0.004785597	0.049207466
NAB1	1.135712127	0.000599545	0.009817263
PIK3CD	1.134066496	0.004039947	0.043212894
GAPDHP1	1.133481856	0.005736911	0.056277123

SLC2A6	1.132199474	0.007463855	0.067526626
COL4A2	1.131520763	0.037540205	0.203185647
H2AFZ	1.129007531	0.008047027	0.071123189
SLC22A4	1.128975851	0.002644457	0.031397963
FCER1G	1.124435626	0.022418864	0.145212171
METRNL	1.123298187	3.13E-06	0.00011494
IKBKE	1.123129239	0.004519968	0.047065901
RPSAP47	1.12220835	0.000215042	0.00422092
EXOSC8	1.122134463	0.000504943	0.008560714
TAF4B	1.121290665	0.000508476	0.008596553
PROB1	1.119543862	0.004178672	0.044334231
FAM83G	1.118869385	1.41E-06	5.85E-05
KIAA1549L	1.118857017	0.008823825	0.07594398
SLC16A7	1.116492849	5.85E-05	0.00146747
USP31	1.11604661	1.18E-05	0.000369686
SLC2A3	1.114494003	0.000381676	0.006803213
SLC6A9	1.113211788	0.04578978	0.230689668
HSD3B7	1.111365129	0.003528243	0.039144549
SREBF1	1.111287465	0.036562821	0.19977064
NCOA7	1.110735868	0.021847586	0.142594094
PLAUR	1.109732812	0.034851129	0.193389775
MYH3	1.107769461	0.000498272	0.008484536
LIMK1	1.10746013	0.002457363	0.029686127
CENPN	1.107022682	0.003426002	0.03825075
INPP4B	1.106859833	0.014317722	0.107299937
HOXD8	1.106655631	0.000199362	0.003982656
FAM60A	1.102552022	0.001891571	0.024415342
AMIGO2	1.100289476	0.033384008	0.18880497
PRKG1	1.099955266	2.24E-05	0.000650216
VAC14	1.098356448	0.035981448	0.197555374
VCAN	1.096512553	0.000991895	0.014696583
ITGA1	1.095823908	0.001461995	0.019885082
PTPN13	1.095537144	8.55E-05	0.002001635
NHSL1	1.094862914	0.001043609	0.01527191
IL17RA	1.093380971	0.000129776	0.002811423
FRMD4A	1.092714328	7.98E-06	0.000261924
IER3	1.08840334	0.039565274	0.210284971
CPNE2	1.088313425	0.000144087	0.003052867
GLIPR2	1.088279603	7.52E-05	0.001806396
MYOSLID	1.087964273	0.027707577	0.166339017
RP11-423H2.1	1.087726901	0.036811465	0.200660182
CDC42EP4	1.085795114	0.000155483	0.00326837
CASC10	1.082932464	0.030757763	0.178522204
DNMBP	1.082799715	0.008997247	0.076939983
JDP2	1.08175191	0.001447344	0.019769861
FHOD3	1.081450661	0.006421277	0.061108106

MSX2	1.079444498	0.034822062	0.193329044
EFR3A	1.07905898	0.000415958	0.007316475
EDEM1	1.076865818	0.000210214	0.004130599
TBC1D31	1.076657453	5.91E-05	0.001478944
AP1AR	1.076448324	6.23E-05	0.001549978
PLK3	1.073858016	0.004802917	0.049314259
DBF4	1.072560073	0.035692679	0.196311746
PMAIP1	1.07197531	0.018410527	0.127246481
TCEA1	1.070613376	0.000526842	0.008837022
ACSL3	1.069826049	0.008113127	0.071529621
RRN3P1	1.069674927	0.000763563	0.011935078
SLC45A3	1.069333289	0.004343841	0.045565486
SH3KBP1	1.068971957	4.76E-05	0.001243098
ACACA	1.068585584	0.008983832	0.076886896
KIFC3	1.067390865	1.47E-07	8.39E-06
GPX7	1.066012821	0.025920203	0.159329718
PGM2	1.065526871	0.002354905	0.028729612
MID2	1.065352055	0.010310774	0.084971293
CHN1	1.064728901	0.004382662	0.045815032
ECI1	1.059128204	0.000394543	0.00699167
GGT1	1.059045916	0.040349371	0.212934498
L3MBTL3	1.057902641	7.37E-09	5.72E-07
ADCY7	1.057561966	0.000328818	0.006053194
IDH1	1.054332881	0.020106508	0.135025949
ME1	1.05028669	0.02464315	0.154144313
AASS	1.049709424	0.010221252	0.084363883
VLDLR-AS1	1.045030991	0.037117358	0.201664365
HSPA13	1.044552799	7.37E-05	0.001777885
SHANK3	1.044219206	0.001367304	0.018857664
RAC2	1.043109566	0.043158589	0.222661311
SLF1	1.043051622	0.03682111	0.200660182
LSS	1.042778295	0.044243852	0.226212993
WDR4	1.041581256	8.67E-05	0.002024192
TRIM2	1.041527259	0.00514328	0.05177141
AFAP1	1.041137036	0.000142019	0.003018035
FLRT1	1.039871729	0.029205602	0.172612389
MEIS2	1.039786534	1.63E-05	0.000488076
NOTCH1	1.039353587	0.000674418	0.010802845
AKAP7	1.038885375	0.000449622	0.007799289
SNX24	1.037456145	1.35E-05	0.000413307
ZBTB38	1.034091449	4.66E-06	0.000162822
ENC1	1.033266534	0.044174064	0.225990174
LINC01615	1.033219082	0.005144141	0.05177141
RP11-756P10.6	1.031224324	0.007145093	0.065766915
WNT5A-AS1	1.029959622	0.024223396	0.152732883
RNF187	1.029523624	0.016172596	0.116784636

NFIA	1.027416177	1.15E-05	0.000361842
TNC	1.026748951	0.019460983	0.132103757
RNF168	1.026651334	2.47E-05	0.000710857
HYAL1	1.025985656	0.025019701	0.155496749
KLHL5	1.025933775	0.006835724	0.063967982
RP11-43F13.1	1.024181412	0.008746466	0.07549894
PROCR	1.019028336	0.006658483	0.062660318
PANX2	1.017577073	7.96E-05	0.001882251
KLF11	1.017026082	0.002232746	0.027572716
ATP2B1	1.012889077	2.26E-06	8.66E-05
LINC01133	1.012649914	0.00156225	0.020872027
DDX21	1.012620543	0.000479242	0.008232774
RAB32	1.012107849	0.014082302	0.106132108
BOLA2	1.012007526	0.026356305	0.16094394
IFIT2	1.010632707	0.047082168	0.234105138
LONRF1	1.010161678	0.000331136	0.006085371
CDK14	1.009381877	0.004369291	0.045764941
BEND3	1.008887634	0.000381085	0.006798351
IARS	1.006653453	0.000147156	0.003105564
PGAM1P8	1.006566807	0.017701896	0.124321149
SHROOM4	1.004502986	0.0006644	0.010671291
IL15	1.003554079	0.029908719	0.175407487
PSPH	1.001922189	0.000733014	0.011542133
CCDC71L	1.000837165	2.66E-08	1.84E-06
XKR8	-1.003295567	1.94E-06	7.65E-05
SDC4	-1.00406525	0.002842649	0.033178755
AHR	-1.006734852	0.001733499	0.022736331
PDCD4	-1.007815487	1.36E-05	0.00041585
SRP14-AS1	-1.008166764	0.033302157	0.188570812
ATP2A1	-1.009129148	0.025299801	0.156826427
CEP250	-1.00921048	8.34E-05	0.001959909
NMRK1	-1.010092271	0.000447538	0.007782135
SYNPO2	-1.012482135	0.035398187	0.195510152
ESR1	-1.013207775	0.025973544	0.159565662
TCAF1P1	-1.014662415	0.002000199	0.025417663
ERCC6	-1.015012089	0.046485921	0.232522083
MYL9	-1.017093393	2.89E-07	1.51E-05
NLRC3	-1.017511674	0.023067203	0.147929617
SLC25A45	-1.018062528	0.002097953	0.026392648
MEX3A	-1.018602933	0.012364166	0.097128812
ZNF436	-1.018645593	0.000184412	0.003745417
DIXDC1	-1.021275939	0.005176543	0.052023889
RAET1G	-1.022728691	0.011975781	0.095093141
ZNF667-AS1	-1.023991554	0.013708862	0.104201025
ZNF334	-1.024073644	0.003587331	0.039514349
NT5DC3	-1.024479377	0.007322415	0.066879731

SYNM	-1.025222202	0.011935111	0.094908057
FNDC4	-1.026849985	0.00732141	0.066879731
SYNGR2	-1.027354367	0.0030419	0.034893533
STON1	-1.029547731	0.000134144	0.002880129
PDCD4-AS1	-1.029628343	0.027140353	0.164052291
CTD-3222D19.8	-1.032283153	0.012077619	0.095581633
MPP2	-1.032439657	0.00838522	0.073322564
SMO	-1.033721758	0.017108638	0.121272389
CTD-2017D11.1	-1.036399131	0.03174014	0.182290074
HOXA10	-1.038160192	2.92E-05	0.000824218
FCGRT	-1.038592232	0.030136554	0.176307515
PAPLN	-1.039513838	0.048471287	0.238510927
KIF28P	-1.039748774	0.046386387	0.232469447
PSMD5-AS1	-1.040313908	0.001741794	0.022813335
CNKSR3	-1.042913346	0.002266661	0.027890121
TTC3P1	-1.04679121	0.004930259	0.050282982
PLEKHG3	-1.047829273	0.01327768	0.101903802
ZNF718	-1.048788384	0.001172327	0.016708596
ZNF763	-1.048798768	0.001715696	0.022582407
NIPSNAP3B	-1.048996984	0.03962295	0.210454387
HERC2P2	-1.049036324	0.044562208	0.22710503
CTD-2015H6.3	-1.04948061	0.00103582	0.015178752
MAPKAPK3	-1.050449139	7.96E-06	0.000261666
SPACA6P-AS	-1.052234314	0.023508643	0.149594976
DNASE1L1	-1.053744398	0.000285273	0.005386358
SHC4	-1.054954604	0.003560976	0.039326358
RP1-111C20.4	-1.058506154	0.030084594	0.17610011
SPDYE1	-1.058566067	0.00961853	0.080955008
ANKRD34A	-1.058720103	0.000417503	0.00732659
SSH3	-1.059174951	1.23E-05	0.00038131
CTD-2334D19.1	-1.059409807	0.040173218	0.212214849
CTC-471F3.5	-1.060242944	0.012895099	0.100120024
ZNF493	-1.061331999	0.00387483	0.041859936
PRDM11	-1.062582138	0.000947722	0.014170003
ERICD	-1.063858213	0.015034978	0.11109077
CTD-3138B18.5	-1.065289569	0.00280969	0.032869908
ULK4	-1.065513909	6.36E-05	0.00157303
ECHDC2	-1.06651885	0.026874911	0.16317675
MFSD6	-1.066571709	0.00019806	0.003964176
DRP2	-1.066762363	0.016356113	0.117460121
TMEM173	-1.067033239	0.001483891	0.020089024
DUSP6	-1.067407802	0.003116193	0.035573621
SCN2A	-1.06849372	0.037716819	0.203728115
ZNF571	-1.071283084	0.003083516	0.035276081
ENDOD1	-1.071657035	0.006009195	0.058331296
ARHGEF25	-1.07172822	0.000447159	0.007781878

FAM110A	-1.071776837	0.008936369	0.0766036
ROM1	-1.072927196	0.03591788	0.197358713
FLNB-AS1	-1.073021621	0.038457527	0.206682569
RP11-380L11.4	-1.073628023	0.026427124	0.161191857
AC009501.4	-1.073819956	0.046682535	0.233041313
RP11-331F4.5	-1.075985048	0.024256222	0.152798561
ATP8B1	-1.077356986	0.008769138	0.075625844
MKX	-1.078040346	0.037570122	0.203192932
PHC1P1	-1.080740894	0.047070672	0.234105138
NPIPB14P	-1.082104743	0.020938447	0.138653852
GOLM1	-1.083558564	0.013238439	0.101703862
CAMK2D	-1.084052654	0.00204194	0.025779186
BST2	-1.08567891	0.038423204	0.206550135
PSD3	-1.085832299	0.020319779	0.135779142
RP11-400F19.18	-1.086551588	0.033387755	0.18880497
BRSK1	-1.086770489	0.002311507	0.028327572
ME3	-1.087359364	0.000171547	0.003532937
PCDHGA2	-1.088009188	0.005102677	0.051451194
NOXRED1	-1.088414858	0.046752749	0.23327194
DSTN	-1.088687838	0.000726786	0.011494959
GBP1	-1.089298724	0.017850528	0.124907696
AP000662.4	-1.089457307	0.010030196	0.083358277
ADAMTS7	-1.089629892	0.015136925	0.111325683
MAP3K14	-1.09143429	8.03E-05	0.001892823
FAM86HP	-1.092836904	0.005954766	0.057908339
EPHX2	-1.094303588	0.032825215	0.186862005
ZNF846	-1.094593389	0.001158025	0.016531284
MELTF-AS1	-1.095700415	0.01393844	0.105273936
DNAH10OS	-1.097466929	0.003960555	0.042527636
PRKCH	-1.098754292	0.011903627	0.09480246
RABGAP1	-1.099985355	2.40E-05	0.000693554
CDC42EP3	-1.10003551	0.000453874	0.007840449
SLC4A7	-1.100672976	0.000713249	0.011331264
FST	-1.100938289	0.015143028	0.111325683
LURAP1	-1.101093315	0.021581146	0.141547901
TCEAL3	-1.1016878	5.62E-14	1.03E-11
ACSF2	-1.105820718	0.040002071	0.211887829
RP11-876N24.4	-1.106926336	0.015326286	0.112275714
CPEB2	-1.107060733	0.003934848	0.042375816
ANKRD19P	-1.107658761	0.046784563	0.233277735
C11orf45	-1.10834519	0.006128132	0.059189599
RP11-384P7.7	-1.109087564	0.015586282	0.113691936
GOLGA8A	-1.109625454	0.04729766	0.234793593
RP11-96H19.1	-1.109716449	0.02343349	0.149336004
AC009950.2	-1.109916508	0.001016591	0.014960156
PNMA2	-1.112395699	0.009842224	0.082125024

RGN	-1.113026781	0.010591011	0.086777958
TMEM56	-1.113748531	0.036990901	0.201182225
RALY-AS1	-1.115748168	0.018299276	0.126805249
ATP8A2	-1.116179631	0.028720682	0.170644519
CITF22-1A6.3	-1.117238277	0.034203149	0.191437144
ZNF664	-1.11780001	0.000348036	0.006335913
STRADB	-1.118997206	0.00328831	0.036964904
TMEM133	-1.119259309	0.031519828	0.181415443
ADGRL1	-1.120295027	0.022227419	0.14423486
NAP1L3	-1.120936413	0.001381832	0.019033415
ALDH4A1	-1.121247721	7.95E-08	4.92E-06
PAPSS1	-1.121630797	5.55E-05	0.001410895
ZNF177	-1.121781764	0.044452569	0.226682605
PAXIP1-AS1	-1.122503219	0.001639716	0.021756825
RP11-195F19.9	-1.124653867	0.002457801	0.029686127
WDR63	-1.12512298	0.028234449	0.16850635
CDHR2	-1.126441473	0.045700604	0.230456566
PCDHB7	-1.12768025	0.018771472	0.128932951
CMYA5	-1.12867937	0.011687578	0.093465577
ZNF418	-1.1297349	0.013928268	0.105268693
SYP	-1.129776661	0.020748363	0.137853484
CD27	-1.134665467	0.030279246	0.176802953
PDE3B	-1.134847546	0.036020651	0.197668882
ZNF385D	-1.135226901	0.021225258	0.139763916
ADARB1	-1.136417085	0.003447168	0.038426734
PLEKHG1	-1.137686743	0.048092886	0.237525422
LINC01341	-1.138607349	0.009031939	0.077143892
RP11-541N10.3	-1.139494278	0.003097956	0.035403313
SPATA1	-1.141859687	0.014999814	0.110892598
SLC29A2	-1.146530761	0.030303191	0.176894361
ZNF273	-1.14794338	0.031299345	0.180438482
RNF144B	-1.149948728	0.005401148	0.053875126
RP11-229O3.1	-1.150047303	0.006949732	0.064637956
RP11-632K20.7	-1.150867151	0.037641364	0.203526645
SPCS3	-1.152928034	0.003446766	0.038426734
LINC00665	-1.154138732	4.28E-06	0.000151293
CMAHP	-1.154230211	0.006114955	0.05914265
TP53TG5	-1.155612152	0.033362843	0.18880497
SEMA6A	-1.156657105	0.019472549	0.132103757
MIR4458HG	-1.157509402	0.007511661	0.067780102
ADAMTS1	-1.157673444	0.02944394	0.173540306
PKIG	-1.161953533	0.004261581	0.0449679
B3GALNT1	-1.163523171	1.03E-05	0.000326342
AK9	-1.163872914	0.008422087	0.073494331
CDKL2	-1.164437656	0.024893474	0.155028361
RP3-405J10.3	-1.164943105	0.002340417	0.028616125

CTD-2368P22.1	-1.166013379	0.020442877	0.136217748
ZFP2	-1.16617645	0.035699201	0.196311746
TRAM1L1	-1.16809786	0.000363726	0.00656264
CTB-13F3.1	-1.170155294	0.02096577	0.138791709
GUSBP9	-1.17247582	0.039318357	0.209514604
RP11-867G23.8	-1.172962742	0.014650044	0.10898652
FHL2	-1.173553657	3.01E-08	2.05E-06
C5	-1.17365354	0.020476318	0.136323484
PLEKHH2	-1.174348283	0.006219245	0.059745074
AC005682.6	-1.179198946	0.026188238	0.160376652
CFH	-1.180690603	0.008862211	0.076212868
GNAO1	-1.180877794	0.024872908	0.155028361
RP11-861E21.2	-1.181455967	0.019008176	0.1300851
RP11-15H20.6	-1.181902335	0.011809068	0.094225234
CCDC92	-1.186149261	0.00013152	0.002841832
SOCS1	-1.187172881	0.012302376	0.096821652
LRRC37A4P	-1.192827391	3.05E-05	0.000850708
AP000648.5	-1.194430829	0.001391587	0.019130738
NR4A2	-1.196000073	0.002848097	0.033216075
PLEKHH1	-1.196901061	0.02633808	0.160928657
HLA-F-AS1	-1.197747627	0.003063299	0.035082417
RP11-989E6.13	-1.198176346	0.027523687	0.165587071
NEB	-1.199225518	0.023510272	0.149594976
PLXDC1	-1.199669598	0.000589186	0.00968128
SUGCT	-1.200028472	0.03358371	0.189432774
MYEF2	-1.201532285	0.003221856	0.036409701
BAMBI	-1.202798175	0.017817331	0.124781814
COLQ	-1.203967551	0.022036853	0.143390758
PLK2	-1.205269377	0.000349955	0.006360004
GPC3	-1.207267107	0.011948024	0.094908057
DOCK5	-1.207521643	0.044594766	0.227190526
CSPG4	-1.207711241	0.003217902	0.036398105
ARHGEF4	-1.209936868	0.006522664	0.061660413
ZFHX4-AS1	-1.21233105	0.006312396	0.060233128
EPHB2	-1.212525005	0.0216155	0.141686114
REEP2	-1.213552574	0.017822745	0.124781814
NACAD	-1.213790495	0.000505571	0.008561003
VAMP5	-1.214680526	0.000432961	0.007565645
ASMTL-AS1	-1.214684238	0.033377591	0.18880497
PCDHB5	-1.215608372	0.007937386	0.070523052
LINC00607	-1.217659336	0.01815685	0.126167467
ALDH3A2	-1.218008303	0.001653675	0.021881488
ANKRD35	-1.218105207	0.004808871	0.049327923
CDKN2A	-1.218862556	0.040156293	0.212214849
LDLRAP1	-1.219424516	4.67E-07	2.28E-05
FBXO41	-1.219680079	0.002024099	0.025614578

SLC25A29	-1.222349091	0.002878789	0.033490649
TOM1L2	-1.223769772	9.95E-05	0.002276879
CTC-308K20.1	-1.223956604	0.025553966	0.157851601
SPTBN2	-1.224128825	0.003850964	0.041623187
KIAA1683	-1.22415263	0.012231003	0.0964378
PLCD4	-1.224475617	9.63E-06	0.000308856
RP11-539I5.1	-1.225086265	0.001450465	0.019787165
PPFIA3	-1.225291888	0.013731529	0.104261884
FER1L4	-1.225529844	0.044438898	0.226667065
RP5-1112D6.8	-1.225787236	0.034396078	0.192113801
SNHG5	-1.22603593	0.007964871	0.070660491
CTC-471J1.2	-1.226431873	0.014089026	0.106145291
TGFB3	-1.227068871	0.002454689	0.029686127
LINC00663	-1.227313038	0.017203492	0.121742534
LXN	-1.228387308	0.014544157	0.108463525
APBA2	-1.228481755	1.76E-07	9.72E-06
BCL2	-1.230015463	0.030766806	0.178526128
SPINT2	-1.23019803	0.006122631	0.059189599
LINC00886	-1.232604911	0.003251567	0.036609731
PRRT3	-1.234140983	0.000106882	0.002402995
IL11RA	-1.234959241	3.32E-09	2.82E-07
CRELD1	-1.238564189	6.45E-05	0.001586378
TJP2	-1.239412516	0.000133298	0.002872778
ITGB8	-1.239558377	9.26E-05	0.002143057
TSPAN1	-1.239604595	0.034943843	0.19369788
CDIP1	-1.243939149	0.000235181	0.004545125
CTD-2017F17.2	-1.244470938	0.031793507	0.182449239
BPGM	-1.244908428	0.00192029	0.024679804
EDIL3	-1.245555364	0.000121699	0.002669948
ABCA11P	-1.245701536	0.010366328	0.08526445
MARCKSL1	-1.247141808	0.000714991	0.011350488
RP1-122P22.2	-1.249487761	0.006284356	0.060116345
IKZF2	-1.249927347	8.29E-06	0.00027008
DPYSL4	-1.24994204	0.000172223	0.003543441
RP1-56K13.2	-1.250871088	0.033157102	0.188148915
ARHGEF3	-1.250898904	0.012135959	0.095936579
SLC9A9	-1.251148106	0.000862436	0.013200099
CTD-2514C3.1	-1.251356748	0.022583434	0.146012166
MTSS1L	-1.251703121	8.45E-05	0.001980801
HOXA10-AS	-1.252416201	0.000649289	0.010486931
ZNF665	-1.25329139	0.006476182	0.061385663
ACCS	-1.253379391	0.009702866	0.081600451
ZNF432	-1.254267041	0.003336031	0.037422486
CFAP70	-1.254356793	0.012631507	0.098647817
TUBB2B	-1.254557524	3.25E-05	0.000903151
CTSB	-1.25516376	0.008688339	0.075229848

C3AR1	-1.256259589	0.024672086	0.154273484
TAF1A-AS1	-1.256548088	0.023085726	0.148003918
RP11-84A19.3	-1.259232591	0.006495781	0.061504813
LRRC2	-1.260195213	0.022718791	0.146665088
TBC1D10C	-1.26073836	0.027400352	0.165051924
GMDS-AS1	-1.261058576	0.000914303	0.013766804
FAM212B	-1.262615286	0.03866912	0.207069604
GOLGA8N	-1.264397537	0.001038954	0.015214225
AF131215.2	-1.265909937	0.001677825	0.02215228
RP11-774O3.3	-1.266151921	0.000204103	0.004039652
NBL1	-1.266909925	5.54E-05	0.001408957
RP4-548D19.3	-1.267360253	0.025581988	0.157978958
TRO	-1.267468082	0.001193243	0.016916304
PSEN2	-1.26973395	1.12E-07	6.60E-06
ZNF883	-1.272570877	0.000635898	0.010301841
GTF2A1L	-1.273081044	0.004899457	0.050112043
ABAT	-1.276300149	0.001506529	0.020299881
DANT2	-1.277264041	0.025680936	0.158406605
RP11-15A1.4	-1.277286059	0.027395536	0.165051924
LLNLR-246C6.1	-1.277321616	0.036317671	0.19883855
MEGF9	-1.277552433	0.000829173	0.012778783
PPP1R12B	-1.282899978	0.000918849	0.013815759
GGN	-1.284404415	0.002655193	0.0315079
ADD3	-1.284900226	0.002735166	0.032165852
LZTS1	-1.285175726	0.000386357	0.006866613
TMEM158	-1.285568439	0.001051937	0.015341174
CDS1	-1.286212424	0.044759606	0.227594692
LINC01547	-1.28629915	5.54E-05	0.001408957
CARD10	-1.289614459	0.013708332	0.104201025
ISG20	-1.290398668	0.002561038	0.0305948
ADSSL1	-1.291849191	0.000297959	0.005596179
GCNT1	-1.291958539	0.016989273	0.120747211
ASPHD1	-1.29387887	0.000222476	0.004338751
PLXNA4	-1.29569558	0.00124415	0.017502625
RP1-283E3.4	-1.296791932	0.016143144	0.116716405
CORO6	-1.297114549	0.000896902	0.013571837
TNFRSF1B	-1.297341168	0.007257189	0.066493782
SCX	-1.297528749	0.00228611	0.028080854
DMTN	-1.298078509	0.026552174	0.161723433
FBXO16	-1.301340697	0.026018022	0.159655023
FAM69A	-1.302390547	0.000161859	0.003375772
PLCD1	-1.302400506	4.01E-05	0.001082393
C6orf163	-1.303513911	0.017712575	0.124324202
ACADS	-1.303551247	0.000829519	0.012778783
CLCN4	-1.303712274	0.013610484	0.10374894
SIM2	-1.303836901	0.016304136	0.117253724

FSIP2	-1.304505353	0.035392403	0.195510152
SPOCD1	-1.304959612	0.000102228	0.002317885
ZNF528-AS1	-1.305238948	0.000133717	0.002876002
RP11-798M19.6	-1.305408969	0.02108336	0.139267669
LINC01004	-1.30574471	0.004424867	0.046188339
PCDH10	-1.310227456	0.043369615	0.223133376
ATF7IP2	-1.310476381	4.41E-05	0.001176744
RBFADN	-1.310922463	0.000661589	0.010661374
SATB1	-1.318948732	0.000342411	0.006265591
TECTA	-1.319254276	0.00128121	0.017903291
DMPK	-1.319640154	4.06E-05	0.001094702
SYNGR1	-1.319921974	0.003617747	0.039728136
RNF217-AS1	-1.320598711	0.01520341	0.111662633
NIPAL1	-1.321257513	0.007133758	0.065708607
CTD-3138B18.6	-1.321650707	0.043284285	0.222965564
MX2	-1.322034738	0.034554544	0.192494974
COL4A5	-1.323699213	0.023982605	0.151547067
AC007228.11	-1.323982756	0.014441181	0.107774498
NXNL2	-1.324431689	0.017424469	0.122817467
RP11-182J1.14	-1.324445669	0.036829081	0.200660182
RAB23	-1.324935505	0.001134038	0.01622793
CEMIP	-1.326834675	0.000413821	0.007284881
GNRH1	-1.327290633	0.002215342	0.027432699
HAPLN3	-1.327369379	0.014974002	0.110816363
CSPG4P11	-1.327808054	0.017339199	0.122381114
AZIN1-AS1	-1.328269142	0.002823737	0.032994115
MACROD2	-1.328567399	0.011576171	0.092783317
RP11-203B9.4	-1.331171293	0.041716558	0.217619679
RAB20	-1.332369056	0.007089659	0.06549396
RP11-876N24.3	-1.332793355	0.04763954	0.235777597
FAM3C2	-1.337097447	0.000730273	0.011524485
HAPLN1	-1.337398814	0.024340356	0.153036095
RILPL2	-1.339220122	4.75E-08	3.09E-06
YAP1P1	-1.340260419	0.037315753	0.202461027
PCDHB9	-1.343793788	0.017476017	0.123116326
MMD	-1.345615021	0.002114186	0.026549897
DISC1	-1.3459654	0.000218239	0.004275106
BAIAP2L1	-1.346352999	0.000318383	0.005891612
VSIR	-1.350217311	8.64E-05	0.002018784
DECR2	-1.350478875	6.55E-09	5.18E-07
CTB-31O20.2	-1.351672194	0.005629346	0.055656959
IRX3	-1.353503518	7.02E-06	0.000234395
VWA5A	-1.355766176	0.033885759	0.190409946
CRISPLD2	-1.357979932	4.89E-05	0.001269691
LINC01588	-1.359757478	0.027336201	0.164851663
AF131215.9	-1.360252787	0.000371651	0.006663486

RP11-715J22.6	-1.36165449	0.046507396	0.232522083
RP11-425A6.5	-1.363006959	0.036785465	0.200627476
CSRNP3	-1.365390693	0.007036696	0.065109279
FGF1	-1.365594791	3.68E-06	0.000132092
ACOT4	-1.365761335	0.002731931	0.032150293
ZNF382	-1.365989404	6.03E-09	4.80E-07
CTD-2349P21.9	-1.368756685	0.007100082	0.065550558
PDZD7	-1.368897622	0.003297863	0.037052772
RP11-173B14.4	-1.369825772	0.024876629	0.155028361
RP11-334C17.5	-1.369943124	0.033792372	0.190092787
RBPJ	-1.370256486	0.000740156	0.011628843
CCDC110	-1.370633362	0.007569923	0.068192908
CRIP2	-1.371037295	0.005393437	0.053823371
RP11-141C7.2	-1.371052645	0.018717537	0.128617513
MINDY1	-1.371418008	0.000164543	0.003415981
CHST15	-1.371636364	4.37E-09	3.65E-07
RP11-394B2.1	-1.372815048	0.004979682	0.050695684
FLJ22447	-1.374613037	0.00039359	0.006980585
RP11-381K20.2	-1.376552037	0.020099226	0.135024274
CSAD	-1.378117098	0.000481349	0.008262315
FSCN2	-1.378226355	0.010100793	0.083758458
NTNG2	-1.378540387	0.013529344	0.103278029
ADAMTS3	-1.380783386	0.001982777	0.02530175
EPSTI1	-1.381742464	0.012639402	0.098673354
TAGLN	-1.382117503	1.04E-06	4.54E-05
CTA-217C2.2	-1.382156122	0.018031192	0.125559242
RP11-298I3.4	-1.38313979	0.012394715	0.097332956
TSPOAP1	-1.385084375	0.025982052	0.159571984
SLC25A27	-1.385822332	0.043289687	0.222965564
CTD-2269F5.1	-1.390947764	0.001494968	0.020187739
ITPKB	-1.391913141	0.00677546	0.063487576
RP11-221N13.3	-1.392777326	0.007183936	0.066038973
PROSER2	-1.395842226	0.003422415	0.038230706
GUCY1B3	-1.396136188	0.032325782	0.184659405
MAST4	-1.396699344	8.17E-05	0.001923746
IL6R	-1.397814413	0.002234962	0.027579612
PMEL	-1.398151272	0.030888801	0.178789513
FLRT3	-1.398424915	0.000890474	0.013503312
MDH1B	-1.402948626	0.033994807	0.190721324
ABCA3	-1.403080497	0.02876077	0.17069257
RP11-131M11.3	-1.4051133	0.00616646	0.059425332
FAM117A	-1.405492557	0.021102216	0.139318165
RP11-121A14.3	-1.406491774	0.020540856	0.136699842
RP11-1072C15.6	-1.40875463	0.021230542	0.139763916
RP3-468K18.6	-1.411960194	0.034936305	0.19369788
FLJ21408	-1.412098282	0.04762108	0.235777597

NETO2	-1.412987677	0.021686407	0.141976426
ECHDC3	-1.414238731	0.029230393	0.172711068
HERC6	-1.416072953	0.003945687	0.042432046
ZNF429	-1.416326867	5.92E-07	2.82E-05
DNAH12	-1.417078031	0.041297668	0.216172444
NPIPB15	-1.417251511	0.003212355	0.036379412
SATB2-AS1	-1.417466484	0.000345545	0.006306705
N4BP2L1	-1.419036927	0.025119739	0.156073018
DGKG	-1.420084852	0.033458346	0.18905383
DYNC1I1	-1.420945534	0.000386521	0.006866613
UPF3AP3	-1.421704822	0.042024847	0.218618797
HOXA1	-1.422470819	4.97E-06	0.000172278
GYG2	-1.4263006	0.008281636	0.072655007
TIAM2	-1.428479591	0.006901582	0.06435846
SMAD3	-1.429602235	2.33E-07	1.25E-05
TCEA3	-1.431790284	0.00136915	0.018870916
RP11-159N11.4	-1.431828459	0.032746942	0.186565758
RANBP17	-1.432661783	3.94E-07	1.96E-05
AC068580.6	-1.433752688	0.025018087	0.155496749
KIAA1549	-1.433892304	0.000739414	0.01162575
SH3RF3	-1.434616076	0.002194528	0.027311561
C4orf47	-1.434868666	0.006294749	0.060118511
PDLIM5	-1.436730031	0.000292903	0.005515772
CALD1	-1.436812714	6.70E-05	0.001636878
AURKC	-1.439592408	0.048709931	0.239354007
PCSK1	-1.444270284	0.031364233	0.18061735
PARD6G	-1.446292058	0.001317652	0.018267334
MAP3K14-AS1	-1.446541164	0.027640944	0.16603243
SCD5	-1.447346939	0.024332849	0.153036095
CRACR2B	-1.448638798	0.014877087	0.110406098
ZNF454	-1.451533351	0.003607979	0.039659888
AK4	-1.457491894	0.003963317	0.042535881
ZNF571-AS1	-1.457601726	0.000169136	0.003490032
CTA-984G1.5	-1.460015351	0.048738548	0.239410529
FSIP1	-1.460493051	0.000826925	0.012757242
SOCS3	-1.460579004	5.41E-06	0.000186046
IFI44L	-1.46253448	0.023386596	0.149140564
USP18	-1.466179353	0.000373119	0.006684186
AC091132.1	-1.467642714	0.032814667	0.186862005
IL1R1	-1.468163687	0.00434077	0.045555663
RP11-333I13.1	-1.469334595	0.044657033	0.227386805
LINC02102	-1.470643326	0.02620681	0.160444332
ANKRD23	-1.473016451	0.046960175	0.233770949
ADAMTSL4	-1.473350915	5.61E-05	0.001421893
EGR3	-1.476235006	0.002992125	0.034489456
CTD-2528L19.6	-1.476318514	0.006976957	0.064806419

HIST1H2AC	-1.477620831	0.023678756	0.150256241
RASGRP3	-1.478170074	0.009815687	0.082116036
OMD	-1.478353588	0.037173093	0.201915763
GVQW2	-1.479441	0.011832378	0.094340668
USP32P3	-1.480513318	0.004090268	0.043634977
LDB2	-1.481561826	0.013414128	0.102766191
RP11-75C10.6	-1.482471484	0.003754101	0.040866072
STK17B	-1.482662556	1.04E-07	6.22E-06
BFSP1	-1.482802581	0.006911603	0.064405903
HIST1H1C	-1.482841814	0.046009661	0.231414927
NR6A1	-1.486982107	0.000574625	0.009489317
MCF2	-1.487133644	0.045671276	0.230399673
SVIL	-1.488553846	0.002247853	0.027706633
OCIAD2	-1.488635031	6.64E-06	0.000222805
FLJ37035	-1.488864682	0.013459682	0.102893505
RP11-87F15.2	-1.490943254	0.008648608	0.074949918
PLEKHA4	-1.490972471	0.00010792	0.002421232
CAPS	-1.49240601	5.83E-05	0.001465674
SNHG25	-1.492830717	0.022059073	0.143491577
CRYAB	-1.493156969	0.030481364	0.177642826
GPR162	-1.494306194	0.003818246	0.041353351
ZNF667	-1.494679777	0.007986939	0.070738617
ARHGEF28	-1.501179233	0.007513121	0.067780102
PRSS23	-1.501421906	1.51E-07	8.57E-06
AQP1	-1.503254285	0.037912736	0.204269227
CEBPA-AS1	-1.503422907	0.030685029	0.17835608
KCND1	-1.503599468	0.004662009	0.048122215
PCDHGA1	-1.503678865	0.005000824	0.050741925
TRABD2B	-1.5041206	0.028665278	0.170362777
RP11-133N21.7	-1.505723314	0.038535089	0.206838908
FAM71F1	-1.509355063	0.026043964	0.159676455
GOLGA2P5	-1.510613469	0.02979555	0.174984271
GMDS	-1.510865389	0.000268124	0.005103199
RP11-266K4.14	-1.514074067	0.011056811	0.089556944
CLDN23	-1.515722797	0.007011817	0.064985884
RP11-1055B8.4	-1.516376313	0.022194564	0.14419708
ANKRD33B	-1.51678097	0.001571698	0.020945508
RP11-356I2.4	-1.518830243	0.008992038	0.076926276
RP1-261D10.2	-1.51957845	0.003537249	0.039164895
DIRAS3	-1.520510356	0.006992946	0.06489843
APLP1	-1.522327784	0.001213553	0.01717725
HPCAL1	-1.522810584	8.88E-11	9.38E-09
STC2	-1.523390569	0.001214065	0.01717725
RNF212	-1.524357889	0.001269588	0.017797594
CTD-2026D20.2	-1.524360379	0.043774869	0.224352776
ALS2CL	-1.527317797	0.025804447	0.158837704

CCNJL	-1.529720074	0.000516356	0.008695329
LINC00933	-1.530022446	0.00953558	0.080447268
ENAH	-1.530735053	6.80E-07	3.18E-05
SCARA3	-1.532339505	3.78E-05	0.001030438
HOTAIR	-1.534690808	3.19E-05	0.000889505
YPEL4	-1.535575578	0.01009463	0.083739905
MDK	-1.536266113	0.001998766	0.025417663
ANGPT1	-1.537587315	5.15E-05	0.001324331
COLGALT2	-1.538596145	0.004493236	0.046810392
LINC00638	-1.538919058	0.005004575	0.050749816
PTCH2	-1.541940761	0.012874784	0.100035099
KCTD12	-1.542568631	0.009789632	0.082006906
ID4	-1.545916934	0.033255655	0.18850761
RGS5	-1.546540831	0.00499315	0.05070626
ZNF781	-1.547585832	0.014992807	0.110879215
RP11-25K19.1	-1.550989889	0.004554673	0.047271671
C1QTNF3	-1.551568133	0.027201912	0.164318324
RP11-707P17.2	-1.554953902	0.017158527	0.121538094
MIR600HG	-1.556818008	0.001160196	0.016546754
FBLN7	-1.560057972	7.34E-06	0.000243188
WBSCR27	-1.561906824	0.000121836	0.002669948
RP11-399B17.1	-1.56218825	0.004783255	0.049207099
PCDHGA5	-1.564159994	4.11E-05	0.001108345
FHL1	-1.566408559	0.00573608	0.056277123
MROH7	-1.566609007	0.020870007	0.138338587
RP11-346C20.3	-1.566636325	0.026743849	0.162566027
CTD-2165H16.4	-1.567267713	0.047583834	0.235775357
BGLAP	-1.567424052	0.0025694	0.030660362
ZNF671	-1.57082747	4.15E-08	2.74E-06
AC007191.4	-1.571142535	0.003241273	0.036546443
CTD-2349P21.5	-1.573593623	0.040665707	0.213916055
FBXL2	-1.574073056	3.24E-06	0.000118207
IFI44	-1.574827108	0.003017006	0.034682563
SLC16A4	-1.576226137	4.14E-05	0.001111496
MARK1	-1.577229069	0.005078784	0.051258717
CLU	-1.577240694	0.006722003	0.063097521
FAM46C	-1.579215355	0.002936936	0.034000252
RP11-799B12.4	-1.579585472	0.008533251	0.074236638
SLC45A1	-1.580529938	0.009529381	0.080442634
AKAP3	-1.582905983	0.001984635	0.02531032
ELL3	-1.584013819	0.001049511	0.015326733
RP11-585P4.5	-1.584283607	0.047858377	0.236586267
RP11-550I24.2	-1.586677829	0.004468285	0.046573194
STARD8	-1.586888654	2.52E-05	0.000720781
CTB-113I20.2	-1.588561049	0.045434796	0.229606066
ASGR1	-1.588729054	0.020072741	0.135015397

RP11-138I17.1	-1.591708498	0.024344043	0.153036095
NIM1K	-1.591809621	0.002454977	0.029686127
RP11-622C24.2	-1.592976898	0.001082367	0.015634157
MEIS3P2	-1.59745911	0.000557618	0.009251429
LYPD1	-1.598066276	0.038376167	0.206401288
IL7	-1.599345395	0.003019505	0.034692598
RP11-1109F11.3	-1.600370322	0.022818038	0.146973328
STAC2	-1.600585784	0.044820216	0.227692677
FILIP1	-1.602166534	0.000400644	0.007076277
COL25A1	-1.604974328	0.018159887	0.126167467
EPCAM	-1.605747696	0.038635865	0.207062105
C1GALT1	-1.607424401	7.46E-08	4.68E-06
SH3RF3-AS1	-1.610617036	0.003307884	0.037145793
EMBP1	-1.611744056	0.025520788	0.157783696
RP11-399K21.14	-1.61475851	0.046468721	0.232517971
GPLD1	-1.615768929	0.033748653	0.190063105
CHAD	-1.617216208	0.043441085	0.223339516
LRIG3	-1.62089165	6.22E-06	0.000210433
EPN3	-1.620963225	0.049645197	0.242225314
CTD-2054N24.2	-1.621390823	0.000926014	0.013894124
JUP	-1.622437408	0.000840684	0.012931428
SCAMP5	-1.623703994	0.014853985	0.110273008
PARP15	-1.623977656	0.000728029	0.011506098
FAM3C	-1.625229474	1.61E-06	6.56E-05
ZNF483	-1.625299781	0.00201047	0.025486016
AC002472.16	-1.626785463	0.037995729	0.204613042
HHEX	-1.629063029	0.04641368	0.232469447
LYN	-1.629182205	1.56E-06	6.38E-05
CTD-2623N2.3	-1.629239812	0.000817513	0.012630316
RP11-13N12.1	-1.632708687	0.039759615	0.210812908
CTXN1	-1.632886699	0.010208925	0.08432738
GHR	-1.634809706	1.72E-05	0.000512
LL21NC02-1C16.1	-1.636960115	0.0464993	0.232522083
TSGA10	-1.638027731	0.000202168	0.004021818
RP11-474P2.2	-1.638430998	0.018521237	0.127803726
PRRT2	-1.641613619	2.16E-06	8.36E-05
NPIPB13	-1.643167753	0.019283514	0.131364318
C1orf54	-1.643482693	1.52E-07	8.61E-06
ALX1	-1.647532233	0.002509043	0.030109274
CTD-2012K14.8	-1.649517695	0.000681421	0.010898651
ZNF540	-1.650680085	0.014640462	0.108953223
OCLM	-1.651128481	0.03375277	0.190063105
CTD-3064H18.4	-1.652846082	0.007038826	0.065109279
CELSR2	-1.653660483	0.007997907	0.070806367
ROS1	-1.659091039	0.011450472	0.092121893
OBSCN	-1.660282565	0.016158771	0.116781883

TCAF2	-1.661405241	5.29E-05	0.001354162
PRDM16	-1.662350555	0.033501229	0.189101173
PLPPR4	-1.662459257	0.00976242	0.081875392
RGS3	-1.663631437	4.23E-28	4.29E-25
RPS20P22	-1.664616541	0.015661295	0.114005843
KRT18P34	-1.665398566	0.013068516	0.100844617
GPR35	-1.666880264	0.021417809	0.140649545
RP1-244F24.1	-1.669956222	0.007848496	0.069949402
MAP3K9	-1.67100887	0.007357135	0.067053329
FAM66A	-1.673978202	0.005520227	0.054806685
GAS6-AS1	-1.674475833	0.000119676	0.002629662
AC025165.8	-1.676421105	0.006576272	0.062084667
GCA	-1.680098068	0.000238999	0.004610565
MCAM	-1.680442635	0.004987357	0.050695684
LA16c-360H6.3	-1.681132042	0.006457292	0.061286827
TTC41P	-1.682780973	0.01343197	0.102829032
RP11-1114A5.4	-1.686516853	0.011920113	0.094898336
CHRNE	-1.686579485	0.016421056	0.117807548
SH3RF2	-1.689241828	0.001886841	0.024369032
CTD-2561J22.2	-1.689459449	0.012694973	0.098998516
FAXDC2	-1.689812695	0.004981282	0.050695684
TBC1D8	-1.691545082	1.15E-06	4.95E-05
ZFYVE28	-1.693541277	2.07E-05	0.000605453
LIF	-1.695222994	0.04430493	0.226416763
POPDC2	-1.695237482	0.030406875	0.177354039
RP11-752L20.3	-1.696505755	0.002457092	0.029686127
C10orf11	-1.696621265	0.039744985	0.210787719
RP3-399L15.3	-1.698144941	0.011359637	0.091658984
CFAP206	-1.700071963	0.005968307	0.058013579
RAB6B	-1.704074767	0.000363873	0.00656264
C9orf3	-1.7045472	4.60E-05	0.001207838
SULT1B1	-1.708199759	0.03406966	0.190940443
GSN-AS1	-1.708867046	0.000375143	0.006714815
RP11-47I22.2	-1.70927434	0.002075166	0.026136802
AC007319.1	-1.709848253	0.048269309	0.237945213
TPD52L1	-1.711561651	0.003568833	0.039357056
ZNF117	-1.713009526	0.000382863	0.006818673
MAOA	-1.713881125	0.045010252	0.228035895
IL17D	-1.716298765	4.45E-05	0.001183483
KCNMB4	-1.720548242	2.62E-05	0.000747883
PROS1	-1.721298705	0.000209049	0.004118439
RP5-1120P11.1	-1.723629746	0.035718594	0.196364833
MICE	-1.72385685	0.000651995	0.010522674
CAPG	-1.724165942	7.22E-05	0.001747777
SNX10	-1.730944964	0.019314886	0.131452124
HIST1H2BD	-1.731642264	0.000313386	0.005809212

CCDC169	-1.732320002	0.001271739	0.017804352
CREB5	-1.73247865	3.05E-06	0.000112569
PCDHB12	-1.735577892	0.018037864	0.125564723
ST3GAL5	-1.738280222	1.91E-06	7.56E-05
TDRD6	-1.738921592	0.005094201	0.05139001
AC005776.1	-1.740910354	0.023835887	0.150998362
RP11-214K3.21	-1.744371911	0.015241642	0.111866418
SMCO3	-1.748681711	0.018679077	0.128477367
FGF7	-1.750186823	5.50E-06	0.000188627
OR7E7P	-1.751881036	0.026958933	0.163407895
FAM155A	-1.752483583	0.000301804	0.005653457
FMO4	-1.753395804	0.002424631	0.029409852
MORN3	-1.755284839	0.006605629	0.062273486
AC034220.3	-1.75787047	0.003219124	0.036398105
LPAR4	-1.758279713	0.036814893	0.200660182
ZNF704	-1.758977109	0.000457398	0.007882918
CSPG4P12	-1.760475477	0.000555838	0.009229066
SPTBN4	-1.760572487	7.72E-07	3.53E-05
GLIS1	-1.762827926	3.41E-07	1.74E-05
TMEM151A	-1.763629099	0.028048947	0.167760394
SH3GL1P2	-1.764121395	0.002325437	0.028465586
AP000892.6	-1.764397578	8.64E-15	1.87E-12
RAPGEF4	-1.768397881	0.03384438	0.190295371
SMIM10L2A	-1.769752597	0.049319406	0.241293017
RP11-397E7.4	-1.77107955	0.040472911	0.213300139
PLPP3	-1.774147356	1.35E-05	0.000415079
OLFML2B	-1.774631565	3.73E-05	0.001022592
MPV17L	-1.775223432	0.003109423	0.035515341
AC007292.3	-1.775420344	0.016026504	0.11614534
FAM169A	-1.776194304	0.009134223	0.077861683
CACNA2D3	-1.776655316	0.043343121	0.223050853
UNC5C	-1.77814844	0.006452477	0.061268377
FAM89A	-1.778985615	1.89E-08	1.34E-06
HIST2H2BF	-1.779681187	0.043042802	0.222363494
RP4-633O19__A.1	-1.780929489	0.026376248	0.161019634
AGBL2	-1.780997196	0.039555716	0.210284971
TNFSF13B	-1.782027911	0.000708772	0.011268524
CFAP53	-1.783074379	0.010802121	0.088002059
ANXA9	-1.785251645	3.45E-06	0.000124892
RAB17	-1.785378869	0.022862487	0.147191917
CTD-2267D19.1	-1.78805336	0.00118308	0.016816914
SLC9A5	-1.788236826	2.00E-07	1.09E-05
TLL1	-1.78842958	0.022216788	0.14421364
NTN4	-1.792983099	0.005059897	0.051116461
CTD-2066L21.3	-1.794473757	0.019538243	0.132465192
EPHX1	-1.796028055	1.38E-06	5.76E-05

CYP1B1	-1.797952649	0.006431931	0.0611822
CDON	-1.798322135	0.000278152	0.005270567
PRG4	-1.798746303	0.016957213	0.120604356
MYCBPAP	-1.799015717	0.021786695	0.142370882
LYPD5	-1.801013654	0.029344078	0.173190939
CTD-2353F22.2	-1.801092894	0.023958429	0.151504756
ST6GAL1	-1.803445361	0.02111611	0.139318165
AC017060.1	-1.803471951	0.013920775	0.105268693
ACTG2	-1.803841968	0.026071271	0.159797943
DIRAS1	-1.80407576	1.71E-05	0.000509158
RP5-1159O4.1	-1.806575845	0.018260591	0.126587979
RP11-671M22.4	-1.806940751	0.007441881	0.067508494
LINC01285	-1.807031189	0.036902491	0.200957517
GPD1	-1.809851096	0.038812792	0.207546296
CTA-243E7.1	-1.810660676	0.027598622	0.165871605
SEMA3E	-1.812894898	0.035424986	0.195577206
CORIN	-1.813755777	0.000342973	0.006270492
NTN5	-1.816597469	0.041887185	0.218242976
A2M	-1.81697073	0.028973222	0.171686943
CAMK1D	-1.817870114	2.30E-06	8.80E-05
RP11-392P7.6	-1.818078547	7.40E-07	3.43E-05
C10orf35	-1.818299853	0.003210534	0.036379412
RP11-90O23.1	-1.818562946	0.046870888	0.233380926
CCBE1	-1.820585869	0.000921831	0.013841092
LRFN1	-1.820606333	0.015111636	0.11125668
SLC2A5	-1.82116099	0.000145124	0.003068741
DTNA	-1.821722569	0.000734313	0.01155405
PPP1R36	-1.823429165	0.004904103	0.050112043
METTL7A	-1.824132433	0.000498951	0.008489321
PRR7	-1.826183592	8.09E-07	3.66E-05
PCDHB8	-1.829306126	0.011172723	0.090295919
ZMYND12	-1.831271911	0.02072876	0.137778447
CITED4	-1.833435288	0.000162084	0.003377162
GAP43	-1.833494813	0.019028665	0.1300851
JCHAIN	-1.833819396	0.008316685	0.072902542
CAND2	-1.834386543	0.000525564	0.008822528
GPC4	-1.835296585	0.009827199	0.082116036
FAM205A	-1.836639179	0.037552919	0.203192932
ZNF528	-1.836739713	2.89E-08	1.98E-06
COL14A1	-1.837193515	0.009986793	0.083071425
ZNF491	-1.837359455	3.16E-07	1.63E-05
MYOM1	-1.83744482	0.000111442	0.002487156
CYGB	-1.838121589	2.39E-07	1.28E-05
AC020951.1	-1.838240231	0.001000782	0.014780182
ARL9	-1.839283426	0.007107854	0.065593937
GAS1RR	-1.841878501	4.82E-05	0.001256155

CYP2G1P	-1.842929024	0.002644391	0.031397963
Sep-03	-1.845731788	0.016182791	0.116805151
MRVI1	-1.847152252	0.000448057	0.007784804
HLA-DPB1	-1.847988343	4.59E-05	0.001206356
SPESP1	-1.848837084	0.046997036	0.23385308
CCDC148	-1.849640496	0.008969824	0.076859506
RP11-173P15.9	-1.851280704	0.030224619	0.176580632
ANKRD6	-1.853431248	0.000138709	0.00295653
RP11-147I3.1	-1.854088172	0.002206783	0.027390295
ACBD7	-1.854255469	0.030724189	0.178424416
PTGER4	-1.858686122	0.003436214	0.038344693
RP11-762B21.5	-1.859047693	0.023009518	0.147781778
CYP4A22-AS1	-1.860771446	0.040100689	0.212213907
ROCK1P1	-1.860958244	0.02356568	0.149820423
NYNRIN	-1.864917491	0.00091407	0.013766804
ERV3-1	-1.868790202	3.47E-05	0.000958196
RP11-61I13.3	-1.87048138	0.002851635	0.033229099
PIK3R3	-1.873049389	3.72E-07	1.87E-05
ZFHX2	-1.876070748	0.009464642	0.080006978
ALDH5A1	-1.877013776	0.002132657	0.026750361
GBP2	-1.877830216	0.020096193	0.135024274
RP11-726G1.1	-1.883398674	0.009270318	0.078707321
PTPRN2	-1.884117861	0.004358895	0.045681914
MISP3	-1.887751844	0.001454012	0.019810211
ADAM21	-1.890254533	0.015142625	0.111325683
RP11-46H11.3	-1.8930667	0.013166121	0.101364253
CYP1B1-AS1	-1.8936153	0.013005864	0.100613893
RP11-493E12.1	-1.893938073	0.012002426	0.095163049
ARHGEF37	-1.894162369	3.74E-05	0.001024772
C7orf61	-1.894172418	0.047217119	0.234531888
NHS	-1.894282328	0.000185871	0.003762557
SLC26A8	-1.896716172	0.021513256	0.141193156
OLFML2A	-1.899340977	0.004530069	0.047125085
MAATS1	-1.90215655	0.006985979	0.064861985
RP5-1125A11.6	-1.902508069	0.034646218	0.192754032
TMEM38A	-1.904602168	0.01190173	0.09480246
TSPAN10	-1.905732283	3.98E-06	0.000142086
HBEGF	-1.906205997	0.000252617	0.004838278
RP11-458I7.1	-1.908891248	0.045385155	0.2294639
CATSPER2	-1.911931386	6.80E-05	0.0016606
MSR1	-1.912418989	0.000114003	0.002531075
RP11-762L8.6	-1.91248543	0.045638072	0.230360518
OSR2	-1.912665337	0.001390332	0.01912581
ANKRD53	-1.915043577	0.000808976	0.012552948
PKD1L1	-1.920593828	0.001528324	0.020508375
GPR39	-1.920684066	0.001510044	0.02031418

CTD-2035E11.5	-1.921479364	0.010044551	0.083389312
CCDC62	-1.923566167	0.015057229	0.111112262
FAAHP1	-1.923825817	0.017117122	0.121292232
SRGAP3	-1.923850841	7.55E-05	0.001812386
CDRT15P1	-1.924608923	0.040089312	0.212213907
TP53I11	-1.92572351	3.59E-07	1.81E-05
TRBV26OR9-2	-1.926116225	0.048825833	0.239647566
RHCE	-1.926197437	0.009024722	0.077113121
CERS4	-1.927000825	0.026155111	0.160219766
AC022007.5	-1.92723269	1.90E-06	7.54E-05
FOXC2-AS1	-1.927473715	0.037867476	0.204128464
FAM87B	-1.928659197	3.92E-05	0.001063839
RP11-91J19.3	-1.928907011	0.033486036	0.189101173
MIR5047	-1.92974397	0.000874311	0.013324494
PHKA1	-1.929991518	1.66E-07	9.24E-06
RAVER2	-1.933718459	2.69E-07	1.42E-05
PTPN20	-1.933772381	0.045600403	0.230305549
SMAD6	-1.934141349	0.000123374	0.00269704
AC074141.3	-1.937399527	0.001105724	0.015908112
ENTPD1	-1.938963019	5.30E-06	0.000182751
TLR1	-1.938980395	0.00375771	0.040884503
RP11-75C10.9	-1.939504962	0.04824747	0.237945213
TMC7	-1.939624082	1.22E-05	0.000377642
HIST2H2BE	-1.939987073	0.006206115	0.059699579
MYO15B	-1.940799333	0.001498284	0.02020694
LIN7A	-1.941250482	8.05E-06	0.000263966
EEPD1	-1.943141794	2.37E-11	2.78E-09
FAM84B	-1.943847409	0.009724724	0.081719854
XXyac-YR38GF2.1	-1.946115236	0.017882012	0.124937439
RP11-2E11.9	-1.946370594	0.001992657	0.025382288
MBNL1-AS1	-1.946430214	5.95E-05	0.001485947
EFCAB1	-1.947194201	0.004921317	0.050239813
RP3-329A5.8	-1.951074845	0.002228316	0.027545399
ITGA3	-1.951137442	3.60E-07	1.81E-05
CLDN4	-1.952337171	0.006607948	0.062273486
TUB	-1.952752595	1.66E-07	9.24E-06
RP11-347P5.1	-1.955900011	0.013715526	0.104214555
EGF	-1.958893849	0.000588886	0.00968128
OR7E2P	-1.958983511	0.009752318	0.08184708
MID1	-1.96119539	1.18E-05	0.000368055
LDB3	-1.961285375	0.00566529	0.055882857
SLC30A3	-1.963254438	0.024970161	0.155415213
RP1-253P7.4	-1.963976606	4.51E-05	0.00119515
MAP7	-1.964058892	5.01E-05	0.001297792
PHEX	-1.964139409	0.000326229	0.006016208
RASEF	-1.966245747	0.005332919	0.053344192

HIST1H4H	-1.968438057	0.00039635	0.007017855
CTD-3234P18.6	-1.968703207	0.009825472	0.082116036
CYP2B7P	-1.968786594	0.003830266	0.041462486
DKK2	-1.973880934	0.041785734	0.217831485
LINC01018	-1.974870866	0.011571245	0.092778684
DEUP1	-1.974887992	0.046332353	0.232394159
SEMA3B	-1.977040667	4.91E-09	3.99E-07
RFPL1S	-1.978497168	0.000299756	0.005624979
RP11-1348G14.5	-1.979116306	0.04258229	0.220528251
LRRC4	-1.981954952	0.027451582	0.165267201
ZSCAN18	-1.982011714	1.04E-05	0.000328701
RP11-96C23.11	-1.982709958	0.010615696	0.08693918
CCDC102B	-1.989503113	0.006754964	0.063351166
RP11-624L4.1	-1.991228918	0.002866682	0.033386209
CACNA1D	-1.99414401	0.01859969	0.128137868
WBP2NL	-1.995266439	0.000892644	0.013526606
TMEM86A	-1.996928099	0.001507078	0.020299881
NOTCH3	-1.997759299	0.008378967	0.073297929
RP5-1063M23.3	-2.004643229	0.002392911	0.029074689
RP11-13P5.2	-2.005138155	0.013341186	0.102317592
RP11-834C11.11	-2.005260992	0.017928704	0.125172389
RP11-20B24.7	-2.008451404	0.007061027	0.065274731
SORBS2	-2.008983856	0.000411556	0.007256998
A2M-AS1	-2.010220772	0.022012175	0.143317596
RAB7B	-2.011919217	0.003313077	0.037184545
PCDHB11	-2.012872719	0.003125718	0.03564883
DUSP4	-2.018737317	0.00142702	0.019542302
CYP46A1	-2.021153814	0.002762034	0.032415153
ASPDH	-2.022232978	0.046862632	0.233380926
PREX1	-2.023172755	6.22E-06	0.000210433
TMEM37	-2.023648148	0.0190574	0.13015643
EFNA5	-2.025175666	3.05E-05	0.000851024
RP11-769O8.1	-2.025366447	0.036657417	0.200082538
RP11-2N1.2	-2.026175606	0.01998709	0.134652526
RP11-16P20.4	-2.026548038	0.006231967	0.059786533
ZNF436-AS1	-2.026809772	2.64E-05	0.000752057
TBC1D8-AS1	-2.027311288	0.01492374	0.110598446
MYOZ1	-2.027411945	0.030331048	0.177008546
PDZD4	-2.029938891	0.018261989	0.126587979
PRCD	-2.030244437	0.000136214	0.002912093
GRIN3A	-2.031607183	0.001923751	0.02469136
PLCE1	-2.033799629	1.81E-06	7.22E-05
GOLGA2P7	-2.036784533	0.019322279	0.131455865
TFP1	-2.041129351	0.032822592	0.186862005
GRIK2	-2.041449573	4.00E-05	0.001080549
ANKS1B	-2.042528644	0.008127247	0.071624507

SEC1P	-2.042878726	0.015703948	0.11419885
TTC23L	-2.042938127	0.02602855	0.159673704
RP11-281O15.4	-2.043901678	0.041567368	0.21720588
HOGA1	-2.04394983	0.00012201	0.002669948
C1orf226	-2.046434557	0.013068774	0.100844617
CHRM2	-2.049460918	0.010733775	0.087711917
ZDHHC15	-2.055584467	0.029311348	0.173045633
RP4-569M23.4	-2.061011159	0.004778581	0.049182731
GALNT16	-2.061638322	0.002362428	0.028769841
GPX3	-2.064246119	0.00716572	0.065899914
RP11-800A3.4	-2.065992012	0.009796153	0.082029329
RGS9	-2.068656242	0.022774118	0.146844542
EDN1	-2.07042966	0.007373668	0.067146637
PCDHB13	-2.070507366	0.00086349	0.01320675
RP5-940J5.8	-2.070836476	0.015334244	0.112275714
CTC-301O7.4	-2.07095897	0.005844205	0.057041152
KRT17	-2.071634422	7.49E-08	4.69E-06
RP11-214K3.24	-2.072737002	0.016212329	0.116899714
RP11-131L23.2	-2.073318324	0.014557432	0.108504686
SLC12A5	-2.074248779	0.022750945	0.146795594
RP11-196B3.2	-2.074867847	0.037330512	0.202461027
GAS2	-2.076054655	0.024259792	0.152798561
DIO2	-2.07734469	0.001982755	0.02530175
GPM6B	-2.083550557	6.11E-06	0.00020807
CA12	-2.083938058	1.47E-06	6.07E-05
ATP1A3	-2.085877089	0.002233111	0.027572716
FAM221A	-2.08627077	0.003953777	0.042497628
DOCK3	-2.088394545	0.000663839	0.010671291
LPAR6	-2.088661486	1.29E-09	1.18E-07
PYGM	-2.090356612	0.024622858	0.154107747
SRGN	-2.095551643	7.77E-05	0.001845645
AC069513.4	-2.096697947	0.039887041	0.211383484
HLA-J	-2.098956525	0.036293794	0.198758827
CDCP1	-2.100492866	3.40E-05	0.000939778
ZNF208	-2.101352675	0.033595437	0.189432774
FAM182B	-2.10180836	0.017043415	0.120999088
HEXDC-IT1	-2.107912331	0.031713525	0.18218626
ARSJ	-2.108100916	2.10E-06	8.16E-05
COL8A1	-2.11032821	4.14E-05	0.001111496
KAZALD1	-2.11356204	0.00015447	0.003250266
GLIPR1L1	-2.115965039	0.025639506	0.158242552
CNN2P4	-2.120431814	0.041510454	0.217075257
HSPA6	-2.121395767	0.033065683	0.187785974
DNM1P35	-2.123086968	0.011306341	0.091272077
AC009005.2	-2.126075978	0.013727276	0.104261884
AC006460.2	-2.134635413	0.045678225	0.230399673

RP11-161M6.6	-2.137804097	0.011787756	0.094125583
GAS6	-2.138992011	1.36E-12	1.93E-10
KIAA1671	-2.14078272	0.00054365	0.009047827
GLIPR1	-2.141739913	0.000100921	0.002296668
LRRN3	-2.142907412	0.010815548	0.088076538
RP11-286E11.2	-2.146353816	0.010267621	0.084681087
PTPRQ	-2.146384217	4.34E-05	0.001160855
STYK1	-2.147246124	0.033950341	0.19062223
C1orf115	-2.150057464	0.001962127	0.025083243
FLVCR2	-2.151771982	3.97E-05	0.001076033
SLC2A1-AS1	-2.155187378	0.001563814	0.020879555
CTD-2231E14.8	-2.156991848	0.020417749	0.136135338
IGFBP4	-2.160037749	3.08E-09	2.65E-07
RP11-50I19.2	-2.160044076	0.048651169	0.239230548
MAGI1-IT1	-2.161802473	0.013455562	0.102893505
TMIE	-2.162895746	0.042443395	0.220226718
AL163953.3	-2.163223761	0.028661035	0.170362777
HTR7	-2.165043	1.02E-06	4.50E-05
PLAU	-2.168797907	6.80E-15	1.50E-12
CTC-325H20.8	-2.169345072	0.006083732	0.058947552
TRAF1	-2.169676414	0.000673947	0.010802845
SLC6A6	-2.170375467	0.000519774	0.008732206
TMEM236	-2.171421516	0.019581203	0.132630018
LINC00511	-2.172080565	0.000183774	0.003741422
CTD-2353F22.1	-2.17504174	0.036417703	0.199181774
FRZB	-2.176623235	7.22E-06	0.000239472
RIMS2	-2.176790051	0.041991478	0.218618797
ZNF204P	-2.177923353	0.010753615	0.087797549
P3H2	-2.178384598	0.000558317	0.009255833
VAMP8	-2.180109854	0.003165268	0.035941544
ADAMTS7P3	-2.181199515	0.024617338	0.154107747
ZNF154	-2.183298814	1.14E-06	4.93E-05
HLA-DPA1	-2.185873235	6.39E-07	3.01E-05
RP11-27G24.3	-2.186397636	0.031852929	0.182642864
SHANK2	-2.191909471	0.004455146	0.046481665
PRDX1P1	-2.194870507	0.012133627	0.095936579
KB-68A7.1	-2.196628702	0.007258344	0.066493782
AC012360.6	-2.196706873	1.25E-06	5.30E-05
FAM124A	-2.197349179	0.007929498	0.07052262
RP11-271K11.5	-2.197547953	0.024224226	0.152732883
NPAS1	-2.197987528	0.001908124	0.024584375
PCDHB14	-2.200605728	0.000346865	0.006319996
FBXW11P1	-2.201019485	0.034025122	0.190740936
RP3-331H24.7	-2.215170355	0.000229234	0.004450359
TMEM255B	-2.21775994	6.01E-09	4.80E-07
TNK2-AS1	-2.219571977	0.001990474	0.025369631

RP3-510O8.4	-2.221069174	0.020359373	0.135900479
LINC00310	-2.224283758	0.003941836	0.042412013
OASL	-2.227442558	0.011412039	0.091900779
SCIMP	-2.227508828	0.000700633	0.011155744
S100A1	-2.22765467	4.32E-06	0.000152272
FCGR2A	-2.228553976	0.030388585	0.177295827
RP11-351M8.1	-2.229628174	5.30E-07	2.57E-05
RASGEF1B	-2.230757894	0.03563707	0.196311746
JAG1	-2.234797866	0.000176069	0.003619081
ELN	-2.23776338	0.012291363	0.09677067
PRRG4	-2.243922098	0.043634189	0.223846853
PEG13	-2.24418033	0.004185136	0.044358695
PIANP	-2.246131345	0.002818472	0.032950636
ARMC4	-2.247059211	0.000222672	0.004338751
C8orf34	-2.251154027	4.48E-05	0.001188268
RP1-122P22.4	-2.257975687	0.02169771	0.142006854
EFHD1	-2.25884953	0.002908599	0.033745437
MAFB	-2.258935301	0.013076581	0.100868379
PLEKHA7	-2.259058785	0.000664705	0.010671291
PLCXD2	-2.259177435	0.003658335	0.040007298
HLA-V	-2.259849038	0.046607251	0.232821101
ELOVL3	-2.260536602	0.000336053	0.006154529
PLEKHG4	-2.261355128	2.63E-06	9.83E-05
GALNT9	-2.266108036	0.01003913	0.083376752
OAS2	-2.266983961	0.000351363	0.006374725
GPRC5C	-2.268599637	0.007983378	0.070736445
CDH15	-2.268924476	0.008005476	0.070843979
GNAZ	-2.269388864	1.39E-08	1.02E-06
ATP8B3	-2.270823798	3.45E-07	1.75E-05
MALRD1	-2.276425321	0.021005847	0.139013884
COL21A1	-2.277205323	0.014327934	0.107338763
RBM20	-2.278123354	0.048332075	0.237945213
SLC2A12	-2.279047465	0.001344671	0.018593578
YPEL1	-2.279610168	0.000126899	0.002762769
CDH5	-2.282226614	0.034016219	0.190740936
RP11-596C23.2	-2.282819401	0.047157567	0.2343708
BMS1P3	-2.283078788	0.049646199	0.242225314
RP11-163F15.1	-2.284395437	0.022383298	0.145069879
GALNT1	-2.285052658	1.27E-08	9.40E-07
NPAS4	-2.285091424	0.02427045	0.152798561
P2RX6	-2.288273209	0.002640142	0.031381655
ZNF559-ZNF177	-2.289829875	0.044985772	0.228035895
AC083949.1	-2.290719369	0.026788159	0.162742641
MARVELD3	-2.293802545	0.037088966	0.201599213
CP	-2.296465409	0.031036898	0.179410259
RP11-337N6.2	-2.297429154	0.032502794	0.185422358

AC009495.3	-2.299126216	0.028054178	0.167760394
AP000654.4	-2.299811541	0.000495856	0.008463659
LINC00472	-2.301709919	1.90E-06	7.54E-05
RP11-175K6.2	-2.303843111	3.89E-06	0.000139633
C1orf145	-2.304014688	0.031164212	0.180044387
LINC02202	-2.305660217	9.83E-05	0.002254559
RP11-96D1.3	-2.309940056	0.037856415	0.20412041
RP11-981G7.6	-2.310343388	0.007196839	0.06612909
ARL4C	-2.3116252	9.51E-06	0.000306534
MGAT4A	-2.315528154	0.043307707	0.222976168
SLC17A7	-2.318567687	0.000528868	0.008864049
TOX	-2.321359928	7.14E-07	3.32E-05
EXTL1	-2.324739284	0.007940145	0.070523052
FAM133A	-2.326967248	0.001032147	0.015135326
C5orf49	-2.330555155	0.001487237	0.020108797
LYPD6	-2.331087418	1.53E-07	8.63E-06
MPP7	-2.333493687	0.000671611	0.010774056
EFHB	-2.344614884	0.032149366	0.184095242
NMNAT3	-2.344861674	0.014230973	0.106837451
TMEM253	-2.349255904	0.021346429	0.140267139
SCIN	-2.351121164	0.004581787	0.047484981
ARHGAP6	-2.351234742	9.00E-07	4.00E-05
PPARGC1A	-2.352329082	0.018530208	0.127824288
DNALI1	-2.35511996	5.47E-10	5.26E-08
FAM20A	-2.356190632	0.005670395	0.055907369
CCR7	-2.358069078	0.007454273	0.067526626
RP11-446H18.5	-2.358510127	0.006536737	0.061762888
ETV7	-2.363313589	0.017579724	0.123625903
LINC00211	-2.364870977	0.004303123	0.045271908
ODF3L1	-2.365068713	0.021186304	0.139601909
HCG4	-2.366283287	0.049637475	0.242225314
CACNA1A	-2.36641563	2.66E-05	0.000755721
ENPP5	-2.367076646	0.014308403	0.107267775
AC098824.6	-2.371590732	0.043133473	0.222661311
TMEM26	-2.37498433	0.000799678	0.012453963
FOXC1	-2.375487103	0.000156946	0.003292635
PTGES3L	-2.383466273	0.000841246	0.012931428
CD37	-2.386470407	0.000167917	0.003474962
FGD4	-2.386737897	0.002503777	0.030099283
HGF	-2.387969558	0.000507802	0.008591962
TSPAN32	-2.388555861	0.006197489	0.059697348
CCDC170	-2.392632549	1.23E-06	5.21E-05
RBM44	-2.396712758	0.001105193	0.015908112
MIR4697HG	-2.39736871	0.030174835	0.176386379
SLC24A3	-2.400550337	3.36E-05	0.000932777
ATP1A2	-2.401760718	0.017979184	0.125278859

ACSBG2	-2.402491473	0.014208973	0.106747409
ZSWIM5	-2.406571611	0.022619248	0.146155143
ATP8B4	-2.409983834	2.18E-06	8.39E-05
REM2	-2.416065244	0.033847532	0.190295371
RP11-588H23.3	-2.417237054	7.65E-05	0.001827136
ARVCF	-2.41751057	8.44E-07	3.79E-05
DNAJC9-AS1	-2.420935558	0.031430093	0.18094778
HECW1	-2.425168077	0.001045774	0.01528262
PURG	-2.426067805	0.01312447	0.101091582
DLX1	-2.426522343	7.82E-08	4.86E-06
C1orf228	-2.429913713	0.006002121	0.058290555
CD14	-2.432530557	0.002802834	0.032823449
AQP3	-2.433744498	1.05E-05	0.000333876
ECM2	-2.434431189	1.18E-05	0.000368055
RP11-494K3.2	-2.43450739	0.028453702	0.169388446
HCG22	-2.437181795	0.047745254	0.236191221
ZNF439	-2.43759016	1.44E-12	2.04E-10
PRR36	-2.440790029	0.012534921	0.09803705
AKAP6	-2.443081479	2.30E-05	0.000666237
AC002480.4	-2.44704673	0.000880698	0.013402686
HIST1H1PS1	-2.450702891	0.024440184	0.153402312
BRSK2	-2.451212766	0.001182977	0.016816914
CXXC5-AS1	-2.452239923	0.028049127	0.167760394
EIF4HP2	-2.453010993	0.012064637	0.095549778
SLC12A7	-2.454053812	0.000732094	0.011542133
ZNF608	-2.455855559	2.10E-06	8.16E-05
HCP5	-2.460550731	0.000330108	0.006071719
DOCK4	-2.461470501	2.41E-15	5.71E-13
RP11-339B21.8	-2.463751634	0.037564456	0.203192932
NYAP1	-2.46490159	5.31E-07	2.57E-05
RP11-381O6.1	-2.467906323	0.032224604	0.184377621
ABCG4	-2.469156093	0.024409285	0.15337873
LINC01589	-2.470386568	0.047172144	0.23438865
ZNF610	-2.472090263	4.78E-05	0.001247009
RP3-495K2.2	-2.473141137	0.003605017	0.039647753
HCAR1	-2.483573632	0.010988772	0.089079193
GAD1	-2.491795894	0.016035798	0.11614534
LINC00202-1	-2.491925554	0.000343655	0.006277591
COL9A3	-2.492415956	0.034740043	0.193056063
PSCA	-2.492926125	1.23E-06	5.24E-05
CTC-471J1.10	-2.49744065	0.019016326	0.1300851
KALRN	-2.499141506	1.67E-11	2.02E-09
ZNF663P	-2.500147768	0.015419624	0.112707464
KIAA0895	-2.508257214	1.68E-07	9.31E-06
SSTR2	-2.508535611	0.021117123	0.139318165
VAV3	-2.508839219	0.02698193	0.163500839

CTD-3099C6.5	-2.513544549	0.018410662	0.127246481
ADORA1	-2.513941741	4.55E-13	6.98E-11
SPRY1	-2.514409972	7.81E-07	3.56E-05
NPNT	-2.516479987	0.046426809	0.232471811
LMOD1	-2.521667707	2.03E-12	2.79E-10
MYO5B	-2.522345255	7.71E-08	4.81E-06
RP4-733B9.1	-2.522501713	0.026317988	0.16089415
LINC01021	-2.523867323	0.018251879	0.126587979
TPRG1	-2.526141577	0.010711297	0.08759534
LINC02015	-2.530373698	0.036279707	0.198758827
ARSE	-2.5320451	6.91E-09	5.42E-07
P2RY2	-2.537101912	0.003047349	0.034937257
AC011524.1	-2.537690956	0.033730923	0.190040395
BMP4	-2.542764657	3.98E-17	1.27E-14
DOC2A	-2.550236959	0.044397237	0.226610388
MUSK	-2.553266972	0.003494444	0.038872501
KIAA0040	-2.554658526	0.015638303	0.113915614
C5AR2	-2.556395866	5.48E-05	0.001398898
GALNT5	-2.559030359	0.000143943	0.003052858
RP11-346C4.3	-2.559274869	0.015312997	0.112235695
GCNT3	-2.55972244	0.013437658	0.102835678
CXCL16	-2.561625356	4.57E-05	0.001204757
ACVR1C	-2.562752707	0.000454894	0.007846096
SUSD2	-2.56549341	8.61E-05	0.002013787
RCAN2	-2.565582397	3.20E-06	0.000117108
ST6GALNAC4P1	-2.566653141	0.026640712	0.162123854
TAS1R1	-2.56725813	0.027544883	0.165641946
HNF4G	-2.569238272	0.000203618	0.004037532
KRT18P52	-2.56930486	0.041094685	0.215481987
SELENBP1	-2.570890177	2.71E-09	2.36E-07
IFITM10	-2.573047002	9.85E-06	0.000314484
PROSER2-AS1	-2.57311262	0.013991027	0.105593404
RGL3	-2.577305917	0.04011122	0.212213907
TMEM221	-2.577876223	0.041914093	0.218329857
OAS1	-2.579246265	0.001786301	0.023252203
ZIC1	-2.58068898	4.92E-06	0.000170865
C10orf55	-2.581250891	0.00011655	0.002574243
HAS3	-2.581443575	2.43E-06	9.21E-05
PCDHB6	-2.582532344	0.001395459	0.01915927
CYP2A7	-2.583871822	1.26E-06	5.33E-05
TMEM229B	-2.584324695	0.001247159	0.017529245
RP11-798K3.2	-2.587383161	0.009985793	0.083071425
RP11-343B18.2	-2.590053663	0.004399387	0.045967349
SPATA20P1	-2.591247309	0.005749526	0.056349057
CAMK2N1	-2.600862549	9.27E-06	0.000299089
ADAMTS14	-2.602319204	0.000135831	0.00290972

ZNF702P	-2.602381102	0.002185859	0.027254189
KLHL3	-2.606752951	3.30E-07	1.69E-05
JAM2	-2.607456783	7.86E-11	8.42E-09
SORT1	-2.608012986	1.47E-30	1.74E-27
LAMC2	-2.611085157	4.68E-05	0.001225867
CCDC114	-2.611472541	0.00284896	0.033216075
COL28A1	-2.612228221	0.008896968	0.076416654
LYVE1	-2.614482475	0.032274082	0.184462851
MTSS1	-2.614999842	9.69E-05	0.002225167
CTD-2349P21.6	-2.618075592	0.010723315	0.087660016
AC104653.1	-2.620791641	0.003958883	0.042527636
NAT8L	-2.62087531	0.013926815	0.105268693
CXCL12	-2.621942654	6.42E-16	1.67E-13
TF	-2.624754181	0.000225791	0.004391502
SCNN1A	-2.627377724	0.000107236	0.002408415
GALNT3	-2.627623392	0.001292408	0.018022757
LSAMP	-2.631648581	7.96E-14	1.43E-11
KCTD19	-2.631741528	0.030497383	0.177666033
TNFRSF19	-2.636666058	2.24E-05	0.000650216
PCLO	-2.638566811	2.77E-07	1.46E-05
RP1-78O14.1	-2.638718381	0.000867101	0.013243003
PPP4R4	-2.638725344	0.013982218	0.105564259
FRMD7	-2.642850899	0.00688091	0.064216384
RP11-1134I14.8	-2.646914613	0.011581884	0.092794246
GJA1	-2.655543061	5.67E-14	1.03E-11
LAPTM5	-2.657452854	0.002200914	0.027349272
OGDHL	-2.658481828	0.007336191	0.066976884
MIR646HG	-2.659668634	0.046772467	0.23327194
TMEM35A	-2.659851562	4.99E-08	3.22E-06
PRR7-AS1	-2.662898952	0.000617044	0.010049806
SLC6A16	-2.66502975	9.67E-05	0.00222214
VSNL1	-2.666016195	7.40E-05	0.001780827
IGSF9B	-2.668818083	0.038673508	0.207069604
FUCA1	-2.67059275	6.60E-16	1.70E-13
PPL	-2.672671765	0.006861294	0.064122898
AP001434.2	-2.680187222	0.000871138	0.013295135
GALNT18	-2.680521639	6.12E-05	0.001524566
CCRL2	-2.68120582	0.008560126	0.074394641
SPTLC3	-2.682885114	6.71E-20	3.08E-17
WNK3	-2.686699135	1.88E-10	1.90E-08
BX842568.2	-2.688515616	0.00762189	0.068443037
RIPK3	-2.6897586	0.000100225	0.002287061
ST8SIA5	-2.690447917	0.006738502	0.063224575
HIST1H2BC	-2.690652008	0.00118675	0.016835433
RP11-553A10.1	-2.696714257	0.005119033	0.051567369
PARD6B	-2.697933613	0.021303852	0.140160034

RP4-758J24.6	-2.698102467	0.00859211	0.074611828
RP3-406P24.5	-2.70145934	0.016450636	0.117940446
VWCE	-2.704833833	0.000279949	0.005295215
P2RX7	-2.706403845	0.001354034	0.01869869
RP11-162D16.2	-2.715664815	0.039490867	0.210160517
PACSIN3	-2.716149482	3.12E-16	8.32E-14
AC091878.1	-2.717615763	0.000194657	0.00389973
AC073410.1	-2.719676361	0.001321276	0.018305674
NUP210	-2.72219774	0.037770171	0.203832426
RP11-93K22.6	-2.725234823	0.013684923	0.104130355
NLRP10	-2.725788427	4.91E-06	0.000170827
TMEM71	-2.726952903	0.001569032	0.020923039
TP63	-2.731380438	0.001051854	0.015341174
RP11-430K21.2	-2.732190849	0.034276569	0.191697218
OSBP2	-2.734901916	3.42E-10	3.35E-08
FAM13C	-2.739165964	0.000110983	0.002479516
RP11-277A4.4	-2.74317312	0.028041025	0.167760394
RP11-806H10.4	-2.745182258	0.022458327	0.145423631
MCOLN3	-2.747470228	0.000162651	0.00338567
TSPAN11	-2.749393627	1.75E-06	6.98E-05
RP11-301L8.2	-2.752969942	0.00164238	0.021765102
BEX5	-2.753040854	0.017780825	0.124711269
RP11-395N3.2	-2.756321106	0.000144811	0.003065159
ICA1	-2.756899826	0.0038324	0.041464547
VMO1	-2.759060068	0.000309297	0.005761276
IL1RAPL1	-2.761705664	0.008520858	0.074204501
SAPCD1-AS1	-2.763624339	0.042584195	0.220528251
DUSP8	-2.766516428	3.39E-05	0.000939327
CRB1	-2.769446021	0.020367325	0.135900479
SYTL2	-2.7703712	9.16E-18	3.15E-15
CDO1	-2.773849761	3.43E-18	1.28E-15
SUSD5	-2.776475711	1.43E-05	0.000434374
NRG2	-2.777366954	0.001061353	0.015425772
LL21NC02-1C16.2	-2.778581444	8.24E-07	3.72E-05
PCDHB16	-2.779217893	2.12E-06	8.23E-05
KIAA1217	-2.784628112	4.92E-13	7.50E-11
SDR42E1	-2.785127572	0.000377185	0.006743032
DNAJC6	-2.7888818	1.05E-08	7.95E-07
LINC00504	-2.790125925	0.040508306	0.213300139
RASD1	-2.793851875	5.66E-07	2.72E-05
SYT14	-2.796274727	2.49E-06	9.41E-05
RNU4ATAC18P	-2.804682502	0.020340628	0.135833378
KRTAP1-5	-2.805986154	0.03136404	0.18061735
KRT18P4	-2.809040767	0.038136956	0.205218184
ZNF793	-2.811786052	2.76E-09	2.39E-07
CLCA2	-2.813334923	1.79E-05	0.000530365

RP11-177H2.1	-2.81632501	0.017877383	0.124937439
TEC	-2.81789208	2.28E-06	8.70E-05
AC073115.6	-2.819618563	0.0166931	0.119165059
SMILR	-2.822318865	0.009805934	0.082079013
PIFO	-2.822685308	0.005661763	0.055873898
CTC-559E9.12	-2.825862853	0.047074737	0.234105138
EPGN	-2.82623479	0.003653742	0.039977554
CYP27A1	-2.828040097	2.53E-05	0.000723004
PDE5A	-2.839396599	1.44E-07	8.22E-06
GOLGA6L3	-2.841471437	0.020873276	0.138338587
TSHR	-2.847579067	0.004555238	0.047271671
PITPNM3	-2.849538625	1.07E-07	6.34E-06
ABLIM1	-2.853539869	3.65E-08	2.45E-06
GIMAP2	-2.85652323	0.004985025	0.050695684
RDH5	-2.857999717	5.72E-10	5.47E-08
COL4A3	-2.86589979	0.000913675	0.013766804
RP11-552F3.9	-2.866821538	0.000581848	0.009593755
RPL36P4	-2.871268127	5.49E-06	0.000188627
CDKL4	-2.87285281	0.035559819	0.19604762
IGSF5	-2.87419298	0.005033107	0.050909845
RPS15AP38	-2.877276607	0.043457112	0.223356691
HSD17B6	-2.880222022	2.94E-07	1.53E-05
EVA1C	-2.885739759	2.57E-13	4.12E-11
CDK18	-2.892407821	0.003561507	0.039326358
RASL10B	-2.892954103	4.40E-07	2.17E-05
PTCHD4	-2.894147363	0.006660743	0.062660318
B3GALT5	-2.89586357	0.002167721	0.027110495
ZNF135	-2.8962553	1.83E-18	7.09E-16
EPHX4	-2.899408941	0.003524955	0.039144549
CKM	-2.901866604	0.010939691	0.088782518
PNCK	-2.902406726	0.00613479	0.059227091
AFF2	-2.902463139	6.99E-12	8.62E-10
SLC47A2	-2.902663408	0.011363464	0.091658984
OLFML1	-2.904447697	5.45E-09	4.39E-07
RP4-569M23.2	-2.905073244	3.00E-05	0.000841422
RP11-693N9.2	-2.905524671	0.002119427	0.026600059
RP11-423H2.3	-2.905801465	0.002356841	0.028729612
PEG3	-2.906213292	0.000115812	0.002560586
HTRA3	-2.912899267	1.03E-07	6.18E-06
ADCY2	-2.918527796	0.000139934	0.002976702
PPM1H	-2.919865567	2.88E-07	1.51E-05
ADCY4	-2.922590747	0.002725843	0.032114067
MKRN7P	-2.925174166	0.001586029	0.021123291
RTN4RL2	-2.925463647	0.024907467	0.155070242
AMH	-2.926918514	0.009750821	0.08184708
GALNT15	-2.931158507	1.94E-07	1.06E-05

MESP1	-2.933718584	0.03489538	0.193584977
KIT	-2.934136812	0.000816543	0.01262447
LINC01535	-2.937363842	4.60E-06	0.000161124
EVI2A	-2.943456407	0.000901003	0.013624243
TNS4	-2.945286532	0.0070156	0.064985884
MYOZ2	-2.945983511	0.002212684	0.027415696
NCAM1	-2.948830112	1.96E-12	2.72E-10
INSRR	-2.95629029	0.006201583	0.059699579
BMP8B	-2.957179425	0.002882756	0.03351852
KLKB1	-2.965858866	1.32E-05	0.000406791
SMKR1	-2.972321759	0.009423361	0.079752809
RP11-823P9.3	-2.976907339	0.044384786	0.226607753
KCNA1	-2.977891711	0.007432997	0.067463029
RN7SL192P	-2.984142645	0.031054808	0.179465163
PDCL3P4	-2.988315263	0.020314746	0.135779142
RPL10P9	-2.988543826	8.95E-07	4.00E-05
CPA5	-2.996117868	0.032355633	0.184780454
ZNF423	-2.997068415	1.99E-08	1.41E-06
GRM6	-2.997744391	0.001271563	0.017804352
ITIH5	-3.000229868	0.02713451	0.164052291
TRAC	-3.003406125	0.003579415	0.039447518
KCTD4	-3.012673743	0.002243522	0.027669238
EPHA5	-3.014518978	0.000854452	0.013087281
RSPO3	-3.01833705	0.000806642	0.012534975
C19orf81	-3.025723692	0.033026861	0.187785974
LINC01111	-3.028094284	0.000193899	0.003891841
PAK3	-3.031453128	8.97E-05	0.002085307
LINC01152	-3.038826743	0.000129729	0.002811423
TSPAN8	-3.039625082	0.025636831	0.158242552
CCDC158	-3.050653914	0.001009736	0.014888545
SIX2	-3.057983362	7.96E-07	3.61E-05
ALX4	-3.070271802	3.01E-06	0.000111155
AC104534.2	-3.070718076	0.027785026	0.166569629
RFLNA	-3.079522527	1.60E-07	8.96E-06
CCL28	-3.085885411	7.06E-05	0.001718633
MIR3179-1	-3.087736214	0.023346606	0.149140564
SLC16A9	-3.087882608	9.04E-07	4.01E-05
ASB2	-3.088808069	0.004138367	0.044038006
CYYR1	-3.091806681	0.001116379	0.016018204
NEK10	-3.094565348	1.58E-07	8.88E-06
RP11-1007J8.1	-3.094707554	0.034162413	0.191359738
SECTM1	-3.102261658	2.13E-08	1.49E-06
HIST1H2AI	-3.105554376	0.016220848	0.116921627
MCHR1	-3.106051438	0.010188535	0.084191548
RP11-9G1.3	-3.106448297	0.015097408	0.111228694
GOLGA6L4	-3.107656147	0.00672069	0.063097521

IQCA1	-3.108903794	0.00017715	0.003631657
EPB41L4A-AS2	-3.112935706	0.024596806	0.154035064
RMDN2-AS1	-3.117987569	5.47E-07	2.63E-05
AMDHD1	-3.118695761	7.89E-05	0.001869542
GJB5	-3.123230343	0.013456733	0.102893505
ACP7	-3.123391468	0.004145077	0.044079831
IGFBP7-AS1	-3.127693808	0.000227811	0.004426762
DCC	-3.136840839	0.000160941	0.003359916
RP5-875H18.9	-3.138686463	0.004928357	0.050282982
SNED1	-3.144045751	3.07E-10	3.02E-08
CACNA1G	-3.152088559	0.000538815	0.008988391
NXPH3	-3.153906506	2.50E-06	9.45E-05
FZD5	-3.154658212	4.46E-13	6.89E-11
ZNF835	-3.162371436	0.02020773	0.135375493
ADD3-AS1	-3.168906477	0.002349567	0.028695109
MYO5C	-3.16909719	0.009590016	0.08077875
SCUBE3	-3.170183784	0.029855122	0.175237657
ERRFI1	-3.186004557	1.29E-10	1.34E-08
TPT1P4	-3.187327285	0.010032079	0.083358277
STEAP1B	-3.188137173	1.66E-12	2.32E-10
RP11-873E20.1	-3.189306908	9.81E-07	4.33E-05
LINC02021	-3.190720434	2.98E-05	0.000837909
DAPK2	-3.199869965	1.01E-05	0.000320797
AC068491.2	-3.211295543	0.015860157	0.115216996
LINC00982	-3.215484975	1.71E-06	6.87E-05
IRF6	-3.215786604	6.96E-05	0.001696478
SMIM1	-3.216391333	0.019332516	0.131455865
CPA1	-3.232556843	0.036544858	0.199723639
PDGFRL	-3.239320289	7.70E-05	0.001833225
RAPGEF5	-3.24056449	0.000969852	0.014430095
COLEC12	-3.243273167	0.020912803	0.138527032
RP11-26P13.2	-3.248080115	0.000309953	0.005765617
RP11-430C7.5	-3.251273993	0.048554563	0.23881055
RP11-17A4.2	-3.252101425	9.68E-06	0.000309826
HIST1H2AD	-3.254036176	0.019294885	0.131399827
SEMA3B-AS1	-3.257928212	0.008726341	0.075409159
RP11-94C24.13	-3.259518457	0.002162811	0.027080826
LINC01619	-3.260141887	0.030598374	0.178081537
SERPINI1	-3.261469757	8.89E-10	8.36E-08
AC005532.5	-3.262828722	2.64E-05	0.000752057
GARNL3	-3.265513834	4.35E-06	0.000153284
NEDD4L	-3.266159078	5.41E-15	1.22E-12
HIST1H3D	-3.266556224	0.010054846	0.083442316
KIF6	-3.26707182	1.70E-06	6.84E-05
ANKEF1	-3.267317213	1.73E-18	6.84E-16
SYT3	-3.280657281	0.031634513	0.181830269

CHRDL1	-3.286556459	0.026762926	0.162635654
LINC01435	-3.289040162	0.000756149	0.011836538
RP11-70D24.2	-3.28947696	0.00324662	0.036573326
HEY2	-3.29072261	0.000284952	0.005385055
CELSR1	-3.292368154	2.71E-06	0.000100989
EGFEM1P	-3.295795552	0.040964282	0.214903842
AC073115.7	-3.297971801	0.045605594	0.230305549
RNU4-78P	-3.29942122	0.042558883	0.220528251
SAMD12-AS1	-3.300065369	0.046998594	0.23385308
DSC2	-3.301307183	0.013423684	0.102802483
ROR1-AS1	-3.308627905	3.14E-06	0.00011494
WDR93	-3.310841639	0.023488567	0.149594976
EPB41L4B	-3.311283895	6.40E-07	3.01E-05
EFNB2	-3.312329305	1.47E-26	1.16E-23
TACR1	-3.316135461	0.000233767	0.004521906
CTD-3162L10.1	-3.320035709	0.037326378	0.202461027
PGR	-3.321237901	0.030577596	0.178009163
DUSP15	-3.324044104	0.003155482	0.035868603
TRIM9	-3.327674713	0.001523053	0.020463387
ADAP2	-3.3288416	0.000515249	0.008683526
ALS2CR11	-3.331694625	1.69E-13	2.82E-11
DOK7	-3.337743166	0.020208661	0.135375493
FAM19A5	-3.341783047	0.018336957	0.126991568
LINC01778	-3.344472849	0.010366005	0.08526445
RP11-478C1.7	-3.344498553	0.024678262	0.154273484
KCNMB2-AS1	-3.345235745	0.020068545	0.135015397
PLCE1-AS1	-3.349674143	0.000274161	0.005204184
FAM47E	-3.354816665	0.000664684	0.010671291
RP11-164P12.5	-3.360614007	0.007848653	0.069949402
RHOT1P1	-3.362853475	0.016163147	0.116781883
CTD-2554C21.2	-3.366841789	5.80E-07	2.77E-05
RUBCNL	-3.372822449	8.01E-05	0.001891595
LTF	-3.375509478	0.017076704	0.121166799
REM1	-3.380789211	0.019719197	0.133395301
HMGB3P4	-3.382303095	0.016571808	0.118490648
FAM160A1	-3.385494556	3.38E-06	0.000122782
MMP13	-3.38623365	0.008916924	0.076529161
MYBPH	-3.386550905	0.003562891	0.039326358
SLC6A13	-3.387679207	0.018969515	0.129945365
MTATP6P16	-3.395828504	0.027014766	0.163653332
CNTNAP3	-3.399512132	0.00648169	0.06140912
LINC00598	-3.403922337	0.046407925	0.232469447
TRH	-3.41747077	0.002586473	0.030846837
INSC	-3.418310601	0.012863648	0.100021423
LINC00520	-3.426746629	0.02224841	0.144327173
RIPK4	-3.42974258	0.00018522	0.003752953

CFI	-3.431173948	5.02E-05	0.001299258
RDH12	-3.431806676	0.02335194	0.149140564
TTC9	-3.432420447	7.47E-06	0.000247041
RP11-61L23.2	-3.43360009	0.000332883	0.006106952
SEMA3F	-3.436208342	2.74E-08	1.88E-06
OTOGL	-3.438646185	0.000108191	0.002424749
HLA-DMA	-3.447961237	2.51E-23	1.68E-20
PITX1	-3.450049333	9.51E-20	4.14E-17
ASIP	-3.45073457	0.028815528	0.170969998
RHOV	-3.454559326	0.002458539	0.029686127
C7	-3.455807418	0.027443088	0.165262695
LINC00890	-3.455892375	0.041546053	0.217154968
CYP2C8	-3.466449016	0.023861755	0.151117366
RP5-961K14.1	-3.470430896	0.03629261	0.198758827
NXPE2	-3.472755783	0.001703306	0.022447021
SLC16A6	-3.475149434	0.00134669	0.018609448
ZNF415	-3.481385821	3.45E-08	2.33E-06
EPB41L4A	-3.486073112	0.000223493	0.004350767
KCNJ6	-3.487351084	2.04E-06	7.99E-05
RP11-93B14.10	-3.503643087	0.046298809	0.232394159
LRRTM3	-3.50501761	0.011132711	0.09000664
CTD-3116E22.7	-3.506333137	0.000378771	0.006762731
CTC-360J11.4	-3.506571049	0.030851169	0.178772551
PRELP	-3.508438613	3.65E-14	7.14E-12
ENTPD3	-3.526621214	7.96E-06	0.000261666
GPR27	-3.536569236	0.00227958	0.028032925
LINC00968	-3.536825465	3.78E-08	2.53E-06
ZPLD1	-3.537615564	0.038675121	0.207069604
MARVELD2	-3.541599185	1.94E-09	1.73E-07
RNU6-323P	-3.544831694	0.028337381	0.169026244
RP11-17A4.3	-3.548651075	0.0001391	0.002961921
RP11-386M24.4	-3.552426008	0.038975789	0.208209173
NLRP3	-3.553832163	0.004191716	0.044406385
GSDMC	-3.566665701	0.019075767	0.130240179
RN7SL526P	-3.569952682	0.017991146	0.125321282
ITGA10	-3.574486947	3.64E-12	4.86E-10
MEOX2	-3.575767025	0.011611459	0.092982298
THSD7A	-3.57724143	6.15E-06	0.00020899
AC093702.1	-3.577832655	0.003230628	0.036470205
SOX6	-3.583352883	8.82E-14	1.54E-11
BTC	-3.58366256	0.044370849	0.226590819
C1QL3	-3.588443687	0.032313752	0.18464012
RASGRP4	-3.592926141	0.046159803	0.231910909
ANK3	-3.593302176	3.16E-08	2.15E-06
C1QL4	-3.595802713	0.030752009	0.178522204
NKX6-1	-3.596421921	0.003234714	0.036497013

RP11-325F22.2	-3.602616833	0.017964822	0.125278859
TMEM255A	-3.610452068	0.002636771	0.031359061
CCDC68	-3.613126669	8.82E-14	1.54E-11
RN7SL589P	-3.618786956	0.003539177	0.039165918
NTRK1	-3.623316402	0.002935988	0.034000252
RPL10P6	-3.624051363	9.96E-07	4.38E-05
RIMS1	-3.635742346	1.55E-06	6.37E-05
LINC00184	-3.636369851	0.006166464	0.059425332
MAN1C1	-3.641375087	0.004834886	0.049547131
LINC01449	-3.644452266	0.020780664	0.137908627
TSPAN7	-3.646610139	8.83E-05	0.002055
DBNDD1	-3.648277875	1.09E-08	8.21E-07
CIITA	-3.649534351	1.21E-14	2.59E-12
CNTN1	-3.661025083	3.93E-13	6.12E-11
B3GALT1	-3.66313339	0.001253127	0.017601523
LINC00840	-3.670503002	0.012490609	0.09787055
PLCB4	-3.670936306	1.90E-09	1.71E-07
FAM134B	-3.675146843	1.42E-05	0.000431431
CNGA1	-3.675271132	0.017220613	0.121782895
AC011738.4	-3.6781313	0.022361348	0.144971658
TUBA4A	-3.684573636	5.10E-14	9.54E-12
RASL11A	-3.690183828	6.88E-09	5.42E-07
APCDD1	-3.691167791	7.66E-05	0.001827177
SOX11	-3.693840787	4.01E-06	0.000143223
LINC00664	-3.711485827	0.00201131	0.025486016
LINC00552	-3.712055834	0.012783476	0.099544255
FMO3	-3.720242273	0.007614951	0.068443037
EXPH5	-3.741823029	2.32E-07	1.25E-05
UBE2U	-3.745812053	0.00817515	0.071957511
TM4SF18	-3.74775079	0.003910709	0.042197805
KRT23	-3.755622643	0.002068806	0.026087501
AC011747.6	-3.756288545	0.012326897	0.096978863
RP11-210M15.2	-3.774390015	5.03E-05	0.001299707
ZMAT1	-3.774792615	8.53E-13	1.25E-10
SOHLH2	-3.779940468	0.000115009	0.002550756
MAOB	-3.783472335	0.000427451	0.00748776
BARX1	-3.7841675	0.001304673	0.018110931
RP11-168K11.3	-3.786953248	2.67E-05	0.000756433
RP11-114H23.1	-3.787481153	0.022886482	0.147301955
RP5-837J1.6	-3.789118776	0.01270964	0.099076681
AP001271.3	-3.789903881	0.046336152	0.232394159
MAPK15	-3.798468281	0.014559848	0.108504686
SCN11A	-3.800008174	0.019841397	0.133967103
RP5-1074L1.1	-3.802136459	0.009919426	0.082639934
ANO2	-3.803480911	0.004066248	0.043465664
SULT4A1	-3.80590785	0.004586571	0.047484981

MCTP2	-3.807219288	2.04E-11	2.42E-09
CYP39A1	-3.812119351	0.000315062	0.00583521
RP11-641J8.1	-3.812866663	0.020369785	0.135900479
CYP26B1	-3.817721353	0.000398182	0.007044452
RAMP1	-3.826035205	4.15E-12	5.39E-10
ABCA13	-3.827873664	1.88E-07	1.03E-05
LINC00882	-3.832947328	5.17E-11	5.77E-09
MAST4-AS1	-3.840602028	0.016404902	0.117755727
MKRN3	-3.845996755	0.000536158	0.008965108
MPPED2	-3.848627484	0.000354896	0.006427904
ANKRD30B	-3.850753715	0.005440081	0.054162192
KCNA4	-3.852438365	0.006216157	0.059742307
ZBED2	-3.854513355	0.029034281	0.171933229
HHIPL2	-3.856008177	2.48E-05	0.000713095
GPRC5D	-3.862899097	0.008359641	0.073188881
GRID2	-3.873120682	0.008267431	0.072599874
ANKRD29	-3.874359926	3.86E-07	1.93E-05
ZIC4	-3.886770728	1.35E-06	5.64E-05
A4GNT	-3.887406889	0.008600226	0.074651922
EEF1A2	-3.895893004	0.006386373	0.060884562
EPYC	-3.896335671	0.001341025	0.018555197
WNT16	-3.903328442	0.00123917	0.017459301
IGSF1	-3.909553069	0.018885494	0.129562987
FRMD4B	-3.912209532	1.27E-06	5.35E-05
RP11-420K14.6	-3.91420487	0.010162751	0.084162044
FAM216B	-3.935411103	0.042673059	0.220727106
TM4SF1	-3.93780657	0.002002452	0.025431137
PADI1	-3.93882176	7.88E-07	3.58E-05
RP11-727F15.14	-3.940591893	0.014374211	0.107577144
CNTNAP3P2	-3.959302459	0.00014357	0.003047964
CEBPA	-3.959406053	1.75E-06	6.98E-05
NPTX2	-3.964448172	0.000492787	0.008418012
RP11-551L14.4	-3.968157425	0.008978648	0.076873365
MEGF10	-3.971718388	0.010183099	0.084179232
SDK1	-3.978229315	2.44E-12	3.34E-10
PRKCB	-3.980727929	0.020044951	0.13495711
BX842568.4	-3.987680542	0.012520045	0.09795661
AC012531.2	-3.991699873	0.008538466	0.074236638
KLHL13	-3.993699098	5.58E-17	1.70E-14
CTD-2561J22.5	-3.995552334	0.001026356	0.015071117
RP11-336N8.4	-4.001226599	0.007721629	0.069105988
RP11-1191J2.4	-4.008302838	0.01652366	0.118278604
MIR27B	-4.014765557	0.000104708	0.00236158
ANKFN1	-4.020049972	0.009239475	0.078539218
CD4	-4.020404104	2.97E-13	4.69E-11
LRRTM2	-4.022130528	1.17E-05	0.000368055

NRXN2	-4.033335799	9.29E-08	5.68E-06
RP5-1028K7.2	-4.036303291	0.001615574	0.021476567
RP11-168F9.2	-4.05707228	0.005233067	0.052468387
CNTNAP2	-4.057956956	2.29E-09	2.02E-07
LINC01614	-4.068352109	3.75E-30	4.00E-27
LINC01515	-4.075466243	2.24E-06	8.61E-05
ANK1	-4.085846901	2.74E-16	7.67E-14
PPP2R2B	-4.086670726	0.000853074	0.01307557
RP11-58A18.1	-4.087501348	0.011380158	0.091729149
MAF	-4.097929227	1.30E-07	7.57E-06
WISP2	-4.102854095	0.001419224	0.019448024
HPN	-4.105106548	0.012490697	0.09787055
ACAN	-4.118376989	2.20E-06	8.46E-05
INTS4P1	-4.120887226	0.005786453	0.056555093
ZNF560	-4.121847952	0.000180505	0.003681884
ITGB2-AS1	-4.131313572	1.98E-09	1.76E-07
FOXQ1	-4.132310987	8.28E-06	0.00027008
ZNF793-AS1	-4.148710893	5.07E-05	0.001309008
RP11-431K24.1	-4.152877403	0.006645239	0.062569645
NKX3-2	-4.153457484	5.85E-05	0.00146747
LRRC4C	-4.163271258	1.67E-05	0.000498668
HID1	-4.165339067	0.003287654	0.036964904
AC016582.2	-4.16728667	0.000990439	0.0146852
BMS1P7	-4.171301336	0.002543531	0.030419724
PTN	-4.182172233	1.22E-22	7.41E-20
CCDC54	-4.188208478	0.00823421	0.072387768
LA16c-329F2.2	-4.188772762	0.016942231	0.120573533
GNA14	-4.190970815	8.67E-09	6.63E-07
PPP1R14C	-4.192433771	2.89E-06	0.000106916
RGS18	-4.197575607	0.028054419	0.167760394
SLC2A8	-4.197692486	8.80E-14	1.54E-11
B3GALT5-AS1	-4.202758196	0.000512673	0.008653795
PADI2	-4.22529415	6.36E-10	6.06E-08
RP11-164P12.4	-4.226974548	0.024881102	0.155028361
CHDH	-4.229081587	2.12E-05	0.000621475
PRSS51	-4.231516051	0.006709739	0.063037867
ITGB2	-4.241177932	4.72E-20	2.29E-17
CPZ	-4.245824134	6.22E-06	0.000210433
LEPR	-4.247910799	1.22E-13	2.07E-11
CNTNAP3B	-4.247987261	0.002357778	0.028729612
GABRB2	-4.24869437	0.005508193	0.054712661
GGTLC4P	-4.250361094	8.80E-06	0.000285792
RRM2P3	-4.25125124	0.010547248	0.086485814
PRUNE2	-4.253921949	1.21E-15	2.99E-13
PHOSPHO1	-4.259380724	0.003035506	0.034838925
LONRF2	-4.260970576	3.47E-06	0.000125142

RP11-100G15.7	-4.262460213	0.004252258	0.044891723
LY75	-4.266501122	8.37E-07	3.77E-05
ANKRD20A5P	-4.273240578	2.13E-06	8.26E-05
CRTAM	-4.276314604	0.002534928	0.030384956
SLC28A2	-4.278813871	0.008858737	0.076212868
EYA1	-4.283239674	1.71E-15	4.10E-13
PPFIA4	-4.286357584	1.20E-09	1.11E-07
ZBTB7C	-4.289752287	0.011367158	0.091658984
RP11-259O2.1	-4.324784513	0.020252134	0.135469604
MYH14	-4.328187371	1.65E-05	0.000494249
CFAP221	-4.342336055	0.014046175	0.105897244
NGFR	-4.347168282	4.40E-09	3.65E-07
RP3-439F8.1	-4.37647993	0.00063643	0.010302639
LRRC25	-4.384381268	0.046820181	0.233380926
CXADRP3	-4.403463748	0.008748188	0.07549894
TFAP2A-AS1	-4.404016952	0.002040655	0.025778223
EYA4	-4.407137391	1.08E-11	1.31E-09
RIMS4	-4.410751876	4.83E-05	0.001256211
KCNMB2	-4.41176399	6.37E-05	0.00157303
DAPK1	-4.422177904	4.96E-31	6.22E-28
OR7E22P	-4.432365576	0.027957982	0.167418331
BTLA	-4.432595495	0.00572107	0.056251033
RP11-388P9.2	-4.432863324	0.007724923	0.069106479
TREM2	-4.438248492	0.013576456	0.103531851
SFTA1P	-4.442805079	4.24E-15	9.73E-13
SMC2-AS1	-4.445208394	6.48E-05	0.001592934
METTL7B	-4.4475896	5.24E-11	5.82E-09
ABCC12	-4.455258495	0.012062257	0.095549778
CPB1	-4.461753553	0.001220599	0.017246818
NOS1AP	-4.467214951	0.001112994	0.015980379
RPL23AP87	-4.483693433	5.66E-08	3.62E-06
TCEAL2	-4.488751746	0.003529401	0.039144549
SPINT1	-4.48917561	0.001354901	0.01869869
TEX41	-4.489869466	3.81E-12	5.02E-10
NPR3	-4.493402372	1.69E-10	1.74E-08
ZNF727	-4.505643353	0.00016692	0.003457679
RP11-379F4.6	-4.507173645	0.004848213	0.049636024
EPHA5-AS1	-4.515838048	0.00199952	0.025417663
RP11-384F7.2	-4.516590553	6.24E-06	0.000210722
LINC01139	-4.522142301	0.0001783	0.003643884
COL6A4P2	-4.524279541	0.005320096	0.053240886
HNRNPA1P68	-4.524561281	0.014108278	0.106252815
AC073046.25	-4.543703916	0.00628179	0.060116345
CATSPER1	-4.554828859	0.001456357	0.019829509
RAI2	-4.555227827	6.30E-05	0.00156333
CCNO	-4.559987979	9.90E-05	0.002269849

LINC01579	-4.56232688	0.011937244	0.094908057
SERPINA9	-4.567107437	0.001962072	0.025083243
PLA1A	-4.573704252	2.55E-06	9.57E-05
HNRNPA1P35	-4.574676648	0.002617206	0.031161104
CDH3	-4.575816642	0.002469535	0.029783879
PALD1	-4.589815386	0.002771574	0.03250924
NFE2	-4.611079266	0.000332513	0.006105426
IPCEF1	-4.618900651	0.000430334	0.007525902
FAM84A	-4.627767373	3.22E-22	1.86E-19
RSPO2	-4.630334554	0.000254328	0.004860867
RANBP3L	-4.633516014	4.52E-09	3.73E-07
CFAP99	-4.637677312	0.02654933	0.161723433
RTN4RL1	-4.642006217	6.96E-09	5.44E-07
PALMD	-4.64482798	5.07E-10	4.90E-08
DLX3	-4.654536359	1.10E-10	1.15E-08
PCA3	-4.672213688	0.001031882	0.015135326
RAB37	-4.67829454	0.001244445	0.017502625
RUNX3	-4.686553133	3.42E-09	2.89E-07
KCNB1	-4.689946962	1.59E-07	8.92E-06
ATP6V0A4	-4.694988102	0.000352239	0.006385187
RP11-454P21.1	-4.698591682	0.000359237	0.006501001
L1CAM	-4.702602481	0.000159475	0.003335842
GRAMD4P7	-4.705760031	0.009201329	0.078277336
EYA2	-4.707893957	2.85E-27	2.36E-24
AC116609.2	-4.719510965	0.001727994	0.022716258
TMEM150C	-4.726088588	8.67E-15	1.87E-12
SERPINA10	-4.728140056	0.019450397	0.132079464
CLEC3B	-4.729875012	3.63E-11	4.14E-09
SHROOM2	-4.75205367	7.18E-20	3.19E-17
SERPINA1	-4.755045944	1.64E-10	1.69E-08
RNU6-403P	-4.774397158	0.018775529	0.128932951
SLC8A3	-4.777091119	0.001055265	0.015368692
RP11-81H14.2	-4.780594165	0.001120467	0.016055257
FOXE1	-4.788722863	9.36E-07	4.13E-05
CPN2	-4.791398151	5.37E-07	2.60E-05
CYP27C1	-4.791706653	1.08E-13	1.87E-11
SYT12	-4.804188732	5.66E-08	3.62E-06
RP11-111E14.1	-4.814338789	0.009251028	0.078574814
RP11-494M8.4	-4.818197218	0.000664354	0.010671291
RNU6-42P	-4.819836185	0.000562398	0.009309011
PRR33	-4.831057108	1.14E-05	0.000361323
POM121L9P	-4.83389897	1.07E-08	8.07E-07
PLCH2	-4.849721419	8.09E-06	0.000264667
RP11-368L12.1	-4.85356581	0.000813267	0.01258829
B4GALNT3	-4.857444634	1.40E-05	0.000428037
AC010890.1	-4.871855499	0.001059609	0.015410912

LINC00173	-4.873237592	0.001526742	0.020500047
CSPG4P13	-4.890343825	0.004586921	0.047484981
MKRN3-AS1	-4.891178693	0.00210323	0.026435586
CHL1	-4.922163724	0.000103736	0.00234462
PREX2	-4.931809799	5.94E-07	2.82E-05
AP001092.4	-4.945537595	1.02E-07	6.14E-06
RP11-18B16.2	-4.948023823	0.000210247	0.004130599
FGFR2	-4.959451613	1.50E-33	2.47E-30
LINC01505	-4.97285222	0.005758195	0.056408103
RP11-757O6.6	-4.979169811	0.004163325	0.044193386
AC011893.3	-4.989698846	0.004283121	0.045150525
MIR30C2	-4.991742804	0.002810026	0.032869908
LINC01444	-4.996287034	1.94E-05	0.000569756
KYNU	-5.008930031	0.000628584	0.01019109
NAT2	-5.034212455	0.001438528	0.019662738
ATP6V0D2	-5.038449056	0.031249385	0.180296614
CTC-535M15.2	-5.040441525	0.002659624	0.031542934
GPR183	-5.06135777	0.001044407	0.015273113
SCN1A	-5.06407611	1.56E-05	0.000469555
RP11-144I2.1	-5.072904684	5.23E-06	0.000180718
TMEM176B	-5.135734674	0.022908574	0.147355238
BUD13P1	-5.142715384	0.001924528	0.02469136
LRMP	-5.14285916	0.00018693	0.003780416
LSP1	-5.150067474	3.16E-09	2.71E-07
LINC01114	-5.154944491	0.000743837	0.011678074
EFHC2	-5.16533635	1.43E-08	1.04E-06
RP11-486O13.5	-5.166229114	0.006912707	0.064405903
TRIM63	-5.177423719	0.013089214	0.100929333
FEM1AP2	-5.184671488	0.001079901	0.01563146
ISM1	-5.192173718	6.14E-12	7.63E-10
GDF5	-5.201686333	5.93E-15	1.32E-12
ITGA7	-5.209768659	7.33E-10	6.95E-08
FAM178B	-5.212883702	0.011084468	0.089675245
FAM181B	-5.222726782	0.015804851	0.114854324
TFAP2A	-5.229664495	2.59E-16	7.35E-14
FGF10	-5.230663003	0.001864552	0.024122521
LINC01268	-5.231525288	1.46E-18	6.01E-16
MRAP2	-5.232253983	1.86E-08	1.33E-06
TBC1D3F	-5.234717549	0.011946007	0.094908057
OR7E28P	-5.241905271	0.007641048	0.068586203
RP11-90C4.1	-5.252588897	0.000233518	0.004521906
XK	-5.296398253	0.000978314	0.014545188
RN7SKP281	-5.297709132	0.01148174	0.092338636
RP11-429A20.3	-5.322733678	0.001433192	0.019614227
CCDC190	-5.324922687	1.70E-06	6.84E-05
IGF2-AS	-5.336161961	0.012961603	0.100380675

PTPN3	-5.337996015	1.99E-22	1.18E-19
OVCH2	-5.354910153	0.004308203	0.045303017
DSG3	-5.354950489	0.000102441	0.002320245
RP4-671O14.7	-5.357225558	0.002307332	0.028325216
LINC01266	-5.376350389	0.002898029	0.033641103
ANGPT4	-5.383713871	3.05E-07	1.58E-05
KRTAP2-2	-5.394373384	0.001733784	0.022736331
INHBB	-5.395228464	1.31E-06	5.49E-05
SAMD5	-5.397709059	2.76E-17	9.21E-15
NTRK2	-5.401926939	2.28E-25	1.62E-22
TMEM108	-5.484103215	4.71E-09	3.86E-07
FLG	-5.486487319	1.22E-09	1.12E-07
PDK4	-5.490353239	2.57E-06	9.64E-05
ITGA8	-5.492491613	1.93E-05	0.000569756
RASL12	-5.502811975	0.043472689	0.223356691
TSPAN18	-5.521512341	2.71E-18	1.03E-15
LRRTM4	-5.541973777	0.008160014	0.071883592
GCNT2	-5.564187776	7.34E-05	0.001770997
KIAA1644	-5.56663563	1.14E-36	2.70E-33
NPY2R	-5.594420591	0.007621528	0.068443037
CD80	-5.627324407	3.95E-05	0.001069798
ZSCAN1	-5.639820983	0.000252058	0.004831913
C20orf197	-5.640547438	0.003708834	0.040435196
BRINP1	-5.662174952	4.66E-14	8.81E-12
RP11-662G23.1	-5.680285965	0.028370626	0.169177103
PENK	-5.689085391	0.000366082	0.006596889
VANGL2	-5.699463447	0.000485811	0.008325514
SYT13	-5.711937904	0.002319406	0.028408066
ALDH3A1	-5.713284958	0.003621528	0.039728136
COL26A1	-5.714570521	0.000852947	0.01307557
OLFM4	-5.736033313	0.001562272	0.020872027
RP11-525K10.3	-5.74466905	0.00133926	0.018542799
CRISPLD1	-5.757951016	2.83E-33	4.31E-30
RP11-535M15.1	-5.767684834	0.000188003	0.003794917
NKAIN2	-5.769560944	4.12E-07	2.05E-05
BEND5	-5.770205467	0.00093603	0.014024675
CSF2RA	-5.77784491	0.000531845	0.008899959
TM4SF4	-5.793191983	0.000250786	0.004811853
ALPL	-5.798145633	3.04E-15	7.06E-13
LINC01443	-5.80379227	1.11E-06	4.81E-05
LRRC37A7P	-5.807349531	2.38E-05	0.000686631
WISP3	-5.815078829	4.72E-08	3.08E-06
CYTIP	-5.870504574	0.003776254	0.041019251
RP11-121G22.3	-5.885189072	0.001298047	0.01807776
AC064875.2	-5.890105876	0.000417563	0.00732659
OR7D2	-5.918764969	2.89E-05	0.000816503

SPAG17	-5.944150856	7.47E-11	8.05E-09
CTLA4	-5.974506449	0.000589426	0.00968128
LAMA3	-5.991701419	6.79E-20	3.08E-17
KIF5C	-6.007340437	3.91E-06	0.000139932
CX3CL1	-6.011791722	0.002070127	0.026088734
RP11-735G4.1	-6.014255894	0.001509344	0.02031418
ERMN	-6.035955204	6.09E-08	3.87E-06
MSLN	-6.038240248	1.07E-05	0.000338764
ACSM5	-6.062486354	0.002310558	0.028327572
RP11-2L8.1	-6.101667012	0.000115441	0.002557689
RP11-145A3.2	-6.111491539	0.000133653	0.002876002
TLR2	-6.133339918	0.002455674	0.029686127
RHOXF1-AS1	-6.156651458	1.71E-07	9.43E-06
GRIK4	-6.177442029	7.17E-06	0.000238236
LEP	-6.178253725	1.13E-06	4.89E-05
DLX5	-6.185685134	8.57E-18	3.00E-15
DCDC1	-6.215786403	1.54E-08	1.11E-06
ST8SIA1	-6.226893962	1.27E-32	1.77E-29
HCK	-6.227318694	0.000242559	0.004666578
GPR78	-6.242151596	0.000164058	0.003411635
NDP	-6.247310712	6.07E-05	0.00151222
FRRS1L	-6.274188993	1.91E-11	2.28E-09
HLA-DOA	-6.289530277	1.51E-06	6.21E-05
HLA-DRB6	-6.309995542	7.61E-05	0.00182025
SYT6	-6.315162306	0.002284033	0.028071506
CTC-340A15.2	-6.32766219	9.41E-08	5.74E-06
CYP4X1	-6.347771518	0.000886793	0.013457052
CRLF2	-6.349435701	4.51E-05	0.00119515
BLNK	-6.364958631	0.002670013	0.031630986
SELENOP	-6.373424916	1.20E-13	2.04E-11
LINC01876	-6.393520036	3.47E-05	0.000958557
RBP4	-6.397472846	4.63E-11	5.23E-09
FAM189A2	-6.427706758	4.84E-06	0.000168848
GPR88	-6.431443517	0.000102087	0.002317885
RP11-679B19.1	-6.434032037	1.47E-05	0.000444641
ADAMTS19	-6.437172406	6.40E-06	0.000215387
INA	-6.473618799	9.00E-07	4.00E-05
FSHR	-6.479898323	1.62E-05	0.000485361
RPTN	-6.491539315	0.000286454	0.005403876
CPXM1	-6.539953755	0.000304255	0.005681102
RP11-168K11.5	-6.566471434	8.45E-06	0.00027474
ANGPTL5	-6.570257282	2.69E-06	0.000100262
RP11-145A3.1	-6.604881994	8.02E-13	1.19E-10
NLGN4X	-6.610582459	4.67E-12	5.97E-10
PPFIA2	-6.64727881	0.000121972	0.002669948
RP11-553K8.5	-6.669552641	5.00E-05	0.001296591

WFDC1	-6.671423963	1.32E-05	0.000406791
IRX6	-6.682080235	0.000952312	0.014226056
SLPI	-6.686270935	7.12E-05	0.001727033
ASB5	-6.733630788	1.59E-14	3.30E-12
GDF5OS	-6.735719061	1.19E-05	0.000371069
LINC01013	-6.743946545	3.42E-09	2.89E-07
LYPD6B	-6.781136154	2.77E-16	7.67E-14
RP11-706O15.3	-6.788138117	1.26E-07	7.36E-06
C11orf87	-6.82647973	9.73E-08	5.90E-06
LRRN1	-6.868806594	0.00167895	0.02215342
RP11-326A19.4	-6.894844895	3.63E-06	0.000130603
TXK	-6.904803031	5.73E-06	0.00019577
LINC01550	-6.917663417	9.56E-06	0.00030719
ATRNL1	-6.955108316	4.98E-08	3.22E-06
SCUBE1	-6.958076422	0.000103048	0.00233154
CST6	-6.975491571	1.05E-06	4.55E-05
RP11-761N21.1	-6.998505946	0.032130518	0.184036707
LINC01423	-7.090064599	4.95E-17	1.53E-14
SLC14A1	-7.101094458	2.05E-27	1.82E-24
RP11-116O18.3	-7.139248471	4.66E-14	8.81E-12
HLA-DRB5	-7.16355776	2.00E-06	7.85E-05
CADM1	-7.225615674	2.67E-42	7.11E-39
CSF2RB	-7.351909	0.002154856	0.026997062
THEMIS	-7.384605172	0.001714631	0.022582325
STON2	-7.394545502	0.002988662	0.034468159
STEAP4	-7.441136011	2.99E-16	8.17E-14
SNAP25	-7.5000819	1.04E-06	4.54E-05
ANO5	-7.520112219	4.84E-09	3.96E-07
CHI3L1	-7.554403303	1.76E-09	1.58E-07
HLA-DMB	-7.57379132	4.45E-09	3.68E-07
RORB	-7.591440312	1.21E-08	9.08E-07
LAMP5	-7.62647509	0.018077964	0.125802819
APOB	-7.62835439	4.53E-05	0.00119719
RP11-1102P16.1	-7.629945722	8.42E-09	6.46E-07
LINC00900	-7.631980194	1.17E-09	1.09E-07
CYP4F35P	-7.695836226	0.002951162	0.034127913
GPR37	-7.762115292	1.99E-08	1.41E-06
VCAM1	-7.947638071	4.99E-23	3.13E-20
LINC00547	-7.948113034	0.009171514	0.07811362
RP11-314N14.1	-7.996566197	4.91E-09	3.99E-07
SLITRK1	-8.033291358	2.95E-06	0.000109224
PLEKHS1	-8.221928262	1.60E-10	1.66E-08
TM4SF20	-8.243702168	6.80E-13	1.02E-10
LANCL3	-8.425175601	4.29E-12	5.55E-10
CHRM3	-8.441650534	1.72E-11	2.07E-09
MLC1	-8.482901967	4.02E-09	3.37E-07

KCNMB1	-8.536959327	2.10E-08	1.48E-06
CTNND2	-8.573289554	0.003083458	0.035276081
SHOX	-8.726450662	1.03E-09	9.62E-08
DLX6	-8.758592262	9.00E-12	1.10E-09
IBSP	-8.810351861	5.06E-10	4.90E-08
CD74	-8.909567817	3.54E-50	1.26E-46
DLX6-AS1	-9.012626021	4.00E-12	5.24E-10
HS3ST1	-9.213807271	0.000876521	0.01334865
RP11-706O15.5	-9.220970947	2.17E-14	4.29E-12
S100B	-9.362531165	8.91E-13	1.30E-10
CCKAR	-9.637057163	7.03E-06	0.000234395
SMOC2	-9.794390661	1.54E-12	2.16E-10
IGF2	-10.07562412	8.89E-66	9.49E-62
LGR5	-10.12442073	3.33E-05	0.000924233
IGFBP2	-10.17394942	1.71E-52	7.28E-49
HLA-DRB1	-10.46894756	3.90E-17	1.26E-14
LBP	-10.92529578	2.08E-07	1.13E-05
HLA-DRA	-11.54216323	9.47E-21	4.93E-18
SMOC1	-13.32734178	1.01E-25	7.41E-23
