# Supplementary material for: Adipose tissue-derived human mesenchymal stromal cells can better suppress complement lysis, engraft and inhibit acute graft-versus-host disease in mice
Source: Stem Cell Res Ther. 2023 Jun 25;14:167. doi: 10.1186/s13287-023-03380-x (PMC10291819; doi:10.1186/s13287-023-03380-x)
Supplement: Supplementary file 10 — Additional file 10: Table S5. List of Differentially expressed genes between UC-hMSCsand BM-hMSCs. Log2 FoldChange > 1 or < − 1, p-value < 0.05. [file 13287_2023_3380_MOESM10_ESM.pdf]

Table S5. List of Differentially expressed genes between UC-hMSCs and BM-hMSCs. Log2 FoldChange >1 or <-1, p-value <0.05

Gene name	log2FoldChange	pvalue	padj
HAND2	13.72039409	1.06E-26	4.60E-24
IL1B	13.62036373	8.52E-24	2.78E-21
HAND2-AS1	11.76498925	6.14E-21	1.47E-18
CNTN5	11.3519125	4.09E-16	5.56E-14
FOXF1	11.31213515	1.35E-18	2.46E-16
DSC3	11.23987021	8.48E-59	4.78E-55
TBX20	11.22703627	2.32E-16	3.29E-14
HOXD11	10.79547766	1.53E-16	2.20E-14
KDR	10.75104554	6.10E-10	3.34E-08
SULT1E1	10.57493337	9.80E-09	4.16E-07
IL1A	10.36845331	5.52E-14	5.81E-12
FENDRR	9.676761654	1.50E-12	1.23E-10
DAW1	9.617649232	2.00E-16	2.86E-14
MMP3	9.616464735	3.08E-07	9.14E-06
BCHE	9.615886269	1.59E-24	5.59E-22
KRT8	9.614076972	3.60E-47	8.11E-44
VAT1L	9.252116819	4.28E-34	3.57E-31
CXCL6	9.230061391	4.98E-07	1.39E-05
HOXD10	9.221016924	1.55E-28	7.42E-26
CARD11	9.215082421	1.07E-45	2.00E-42
CXADR	8.896073301	1.57E-14	1.79E-12
ALDH1A1	8.764476872	5.34E-10	2.97E-08
CXCL5	8.578085116	1.73E-13	1.69E-11
DPP10	8.538113158	1.82E-09	8.99E-08
HOXB9	8.51519925	0.001494996	0.012755002
CACNG7	8.442259364	4.88E-09	2.21E-07
MYRF	8.375748401	6.89E-77	7.76E-73
NETO1	8.343637069	4.67E-08	1.69E-06
IL33	8.240471356	0.000484061	0.00504915
RP11-438B23.2	8.230609688	0.000646113	0.006458552
TATDN2P3	8.18665564	8.89E-10	4.70E-08
HOXA13	8.156511137	1.69E-28	7.91E-26
ZSWIM5P3	8.147113059	5.91E-09	2.63E-07
STXBP5L	8.023608859	8.55E-09	3.68E-07
LINC01679	7.943623266	9.58E-10	5.02E-08
TRBC2	7.935371757	1.32E-09	6.73E-08
NPPB	7.883795878	7.27E-17	1.09E-14
FLT1	7.677065538	1.51E-24	5.49E-22
SSTR1	7.642638733	1.09E-33	8.48E-31
KCNK13	7.54871237	6.06E-07	1.66E-05
SLC35F3	7.545984196	9.11E-09	3.90E-07
BRINP3	7.502620752	6.26E-07	1.71E-05
RP11-109L13.1	7.478010438	1.59E-11	1.11E-09

HTR1D	7.447257938	1.78E-07	5.66E-06
HIF3A	7.434654549	1.18E-07	3.87E-06
FDCSP	7.370233417	8.70E-07	2.27E-05
TNFRSF9	7.348309224	4.73E-08	1.71E-06
PARM1	7.314210587	1.27E-21	3.17E-19
CXCL8	7.274845908	2.42E-07	7.43E-06
ARAP2	7.203453807	2.90E-31	1.77E-28
KRT18	7.195999206	5.46E-57	2.46E-53
HOTTIP	7.163974382	8.48E-10	4.50E-08
MMP1	7.09700644	1.32E-07	4.33E-06
SOX17	6.945180919	3.40E-05	0.000560057
LINC01501	6.898551216	0.000352325	0.003904499
DMRT2	6.892671602	7.14E-08	2.47E-06
AC068490.1	6.845076788	1.07E-05	0.000204704
SEMA3D	6.84306616	2.70E-25	1.07E-22
ASXL3	6.842414637	3.26E-06	7.20E-05
GPR143	6.841399168	1.97E-06	4.63E-05
MDGA2	6.784312964	1.13E-06	2.81E-05
ST6GALNAC3	6.772718881	1.76E-29	9.69E-27
KRT8P36	6.715427785	2.44E-07	7.46E-06
PAQR9	6.52626374	1.44E-05	0.000267496
FUT1	6.471632492	6.27E-05	0.000940893
RP11-38L15.2	6.456951283	0.000314261	0.003563255
DSC2	6.444248767	1.58E-24	5.59E-22
ARSH	6.421854414	8.22E-05	0.001192094
NIPAL4	6.398156363	7.20E-06	0.000143922
PLD5	6.391229648	0.001545169	0.013079657
RP11-706O15.7	6.374785425	6.06E-05	0.000913171
F3	6.368407794	6.30E-37	5.92E-34
EEF1A2	6.367303768	3.75E-15	4.65E-13
SHC3	6.347587989	8.73E-25	3.39E-22
PI16	6.327238687	0.000130241	0.001747474
ST6GAL2	6.326884461	1.98E-14	2.24E-12
SPOCK3	6.308265934	1.05E-16	1.54E-14
NDST3	6.303656539	6.08E-08	2.13E-06
KRT8P3	6.297068756	1.21E-10	7.37E-09
HOXB-AS4	6.294282481	0.003354102	0.023940726
KCNH5	6.278428939	0.000541556	0.005553778
MOV10L1	6.258130265	4.35E-16	5.87E-14
ANXA8	6.253044355	1.69E-14	1.92E-12
RP11-567M16.2	6.174361462	0.000130472	0.001749538
KCNN2	6.167193314	5.04E-13	4.60E-11
ANXA3	6.166584115	4.31E-46	8.82E-43
OR51E2	6.153093163	0.000117703	0.001606014
GSDMA	6.136779183	5.82E-05	0.000885561
GALNT13	6.118635264	8.97E-10	4.72E-08

CAMK2A	6.036770472	1.45E-29	8.19E-27
RP11-672A2.5	6.035970813	0.000124824	0.001686848
HTR1B	6.010264735	4.79E-07	1.35E-05
DKFZP434K028	6.009418923	0.001031422	0.009447088
ECEL1P2	5.99762962	0.000457776	0.004821839
AC008063.2	5.995073054	2.82E-09	1.34E-07
ASTN1	5.979095963	0.006381733	0.039239127
LMNTD1	5.978030888	0.003380837	0.02405538
ODAM	5.975356477	0.000633902	0.006351946
PRR9	5.971165459	0.000450039	0.004755894
CADPS	5.952698722	2.88E-05	0.000486601
ANXA8L1	5.924477275	4.43E-37	4.34E-34
FGF9	5.921851855	0.001176128	0.010541192
NPY4R	5.898846652	6.90E-06	0.000138954
IQGAP2	5.871210153	2.35E-20	5.40E-18
CDH10	5.850313284	5.80E-29	3.04E-26
LINC01354	5.842483367	4.89E-05	0.00077232
PKHD1L1	5.841730529	0.001079254	0.009817379
RP11-566K19.5	5.827218737	0.000952027	0.008856644
CXCL3	5.789763523	0.000640927	0.006412404
PKP2	5.784538118	9.59E-05	0.001350767
IL1RN	5.758361204	0.005435773	0.034818914
RP1-170O19.14	5.743184453	4.11E-06	8.86E-05
ADGRF4	5.741896554	1.05E-07	3.49E-06
LIX1	5.727779382	8.43E-05	0.001213411
TRIM55	5.722836082	0.000290525	0.003348804
LINGO2	5.716185278	0.000634039	0.006351946
RP5-1011O1.2	5.694692689	0.000373609	0.004091946
TMEM88	5.677493833	5.33E-17	8.17E-15
ST6GALNAC5	5.671364172	1.01E-47	2.52E-44
WNK4	5.662537055	5.58E-24	1.88E-21
LINC02057	5.621749854	1.83E-05	0.000329139
ADGRG6	5.6164869	2.55E-45	4.42E-42
RP11-713C5.1	5.610821586	0.002261799	0.017671827
EPB41L3	5.599402716	4.97E-29	2.67E-26
ADAM19	5.580350323	4.91E-149	1.11E-144
SLITRK5	5.569467349	0.000240642	0.002865457
AC007743.1	5.551652763	1.41E-15	1.80E-13
MT1G	5.535466615	0.004151569	0.028254986
RP6-65G23.3	5.522568608	0.000117596	0.001606014
SGCG	5.487230418	9.52E-23	2.59E-20
MMP23B	5.467510111	0.000248594	0.002944587
S1PR5	5.465499044	8.91E-19	1.69E-16
MAP3K21	5.44890778	2.73E-06	6.15E-05
RP4-564M11.2	5.438935084	9.92E-05	0.001387347
RP11-4B16.1	5.438142695	0.002547765	0.019388644

RP11-429E11.2	5.422886171	0.00026025	0.003060145
RP5-1011O1.3	5.419614466	0.004558863	0.030348889
ADAMTS16	5.407521953	2.08E-10	1.22E-08
Mar-03	5.404878358	3.76E-32	2.57E-29
RP11-736N17.10	5.399052107	0.00013275	0.001775327
RP11-644A7.2	5.380977637	0.006209422	0.038526448
LRRTM3	5.367540266	1.35E-13	1.35E-11
CDH20	5.364547701	2.98E-05	0.000499829
ANXA10	5.359746839	2.65E-17	4.27E-15
CCDC85A	5.355418038	2.50E-22	6.55E-20
TIMM8AP1	5.347040477	5.85E-10	3.22E-08
RPL9P9	5.33658942	1.25E-61	9.39E-58
CDH7	5.293893038	0.000312457	0.003554009
RP11-80H5.7	5.283296084	0.002265963	0.017698112
NELL2	5.266770149	0.003418526	0.024262281
RP11-82L18.2	5.265565957	2.24E-12	1.78E-10
RP11-90C1.1	5.261825575	0.000668847	0.006626375
FAIM2	5.261024455	5.93E-08	2.08E-06
CACNG8	5.259905701	1.06E-07	3.49E-06
C4BPB	5.224032299	2.59E-05	0.00044339
PLEK2	5.214565305	6.83E-13	6.06E-11
HOXD13	5.213723835	0.001148828	0.010354147
RP11-95G6.1	5.208593761	0.007909617	0.046333335
RP11-148L24.1	5.207253135	0.000369005	0.004053486
OXTR	5.191872273	7.04E-17	1.06E-14
HNRNPA1P33	5.136082403	2.98E-07	8.93E-06
ACTG2	5.133513343	1.88E-10	1.10E-08
AADAC	5.129233557	0.000250626	0.00296399
HAPLN4	5.09817904	3.22E-05	0.000534479
MYOCD	5.084416287	1.28E-18	2.35E-16
NPFFR2	5.065675652	0.001265413	0.011159496
AC156455.1	5.033894313	2.76E-20	6.28E-18
ZNF280A	5.003680679	0.001240005	0.010978375
RPL21P133	4.996753557	0.007994429	0.046732994
STRA6	4.992099987	8.67E-13	7.49E-11
TYRP1	4.967496585	2.74E-10	1.57E-08
AC106801.1	4.953415696	0.004407569	0.029603997
LRRN4	4.94784774	0.000105324	0.001460089
STXBP2	4.926572964	1.18E-14	1.36E-12
ARSI	4.913919833	3.09E-32	2.18E-29
ADD2	4.888818887	1.93E-05	0.000344732
PAK6	4.877110821	0.00297694	0.021802353
CH17-360D5.1	4.874896241	0.004326473	0.029163582
SYNPO2L	4.860749562	2.10E-07	6.54E-06
LINC01561	4.852450682	0.000114497	0.0015737
GPRC5B	4.812490563	3.34E-18	5.93E-16

CACNG6	4.801093365	0.003900931	0.026923114
SUPT20HL1	4.799006602	0.003945898	0.027125492
DNAJC22	4.750337846	5.76E-14	6.01E-12
ZDHHC8P1	4.749744143	6.16E-06	0.000125789
LINC01504	4.738186322	2.40E-13	2.29E-11
TM6SF1	4.724085122	2.45E-07	7.48E-06
CSMD3	4.655011331	0.003171216	0.022933073
SLIT2-IT1	4.618354714	0.004799551	0.031596576
IP6K3	4.596152404	1.24E-05	0.000232561
PFN1P8	4.59362355	0.005104186	0.03324284
TRIM14	4.561516654	9.41E-19	1.77E-16
GSTT2	4.55563482	0.000391167	0.004255449
GPAA1P1	4.545578237	0.004136234	0.028184658
RP11-563J2.2	4.529931641	0.004560631	0.030351695
PDLIM3	4.528485259	7.59E-18	1.32E-15
TRGV8	4.527537505	0.00283625	0.021006137
RTN1	4.52690858	2.65E-19	5.29E-17
CTNNA3	4.512682399	0.001471326	0.012602754
PDIA2	4.494306563	0.003980232	0.027328177
ELMOD1	4.491982428	0.000852282	0.00806181
ZNF804A	4.478033413	1.51E-13	1.50E-11
GBP4	4.466151667	8.38E-08	2.85E-06
DPY19L2P1	4.456895333	8.59E-13	7.45E-11
TMEM156	4.455511682	0.008074421	0.047029851
NOS3	4.45295528	3.03E-13	2.85E-11
ZP1	4.451496525	0.0058284	0.036667026
TMEM74	4.448825827	0.007404006	0.043919397
RAMP2-AS1	4.445739115	0.004225708	0.028633798
EBI3	4.444151021	0.006372471	0.039212394
PTPRH	4.423255053	1.06E-10	6.54E-09
SP140	4.414493458	0.008056153	0.046947709
MBP	4.382828155	2.62E-17	4.24E-15
GRID2	4.361248694	7.19E-07	1.93E-05
USP43	4.343509405	2.10E-06	4.91E-05
SC22CB-1E7.1	4.338090162	2.90E-06	6.47E-05
LINC02086	4.337082004	0.007756696	0.045598747
TRHDE	4.331973313	1.58E-10	9.38E-09
SLC8A1-AS1	4.327968496	0.001102993	0.009988978
CYP4F26P	4.326685552	2.99E-05	0.000501864
TOR4A	4.291423774	9.61E-44	1.44E-40
Dec-01	4.290948054	4.28E-05	0.00068579
SORL1	4.290065169	1.81E-12	1.46E-10
RP11-1L9.1	4.283265013	0.007775494	0.04567824
BNC1	4.258646402	1.09E-17	1.83E-15
RASAL3	4.256245052	0.000337861	0.003783523
AP1M2	4.242760595	0.003479686	0.024620039

GABRA5	4.240954332	8.29E-07	2.18E-05
BEST3	4.223690125	6.01E-08	2.11E-06
SERPINB7	4.223431069	1.02E-06	2.61E-05
F11R	4.181461177	8.31E-08	2.83E-06
TINAGL1	4.157073428	5.28E-13	4.80E-11
RP11-180C1.1	4.145058044	0.000396767	0.004297706
HRASLS	4.122127888	1.68E-08	6.72E-07
LPXN	4.114278263	2.66E-30	1.58E-27
CDH4	4.108331339	5.03E-17	7.82E-15
CD200	4.098325377	1.74E-08	6.96E-07
RP11-30K9.5	4.093652468	0.000824305	0.007855012
MYZAP	4.093187765	5.44E-11	3.51E-09
RP11-481J13.1	4.087632195	1.51E-05	0.000278198
PTHLH	4.084993804	4.46E-12	3.34E-10
UCP2	4.077505893	2.68E-06	6.07E-05
GPRC5A	4.068764129	9.89E-25	3.78E-22
PANCR	4.060526848	0.000437063	0.004653677
TEK	4.049978802	2.47E-13	2.35E-11
RAMP2	4.036171647	1.63E-05	0.00029792
JPH1	4.030831914	1.55E-08	6.28E-07
TRHDE-AS1	4.029921339	8.59E-13	7.45E-11
PCDH10	4.004296462	5.57E-10	3.09E-08
RP11-655C2.3	3.994595385	0.002506251	0.01913733
LINC00920	3.978901369	0.000120991	0.001643911
TNNT1	3.971554046	1.18E-05	0.000222536
ACTBL2	3.967080812	1.10E-06	2.76E-05
TRPM2	3.966408129	2.33E-06	5.41E-05
LINC00670	3.960586119	7.41E-07	1.98E-05
TSTD1	3.9541022	0.008546325	0.049118488
DPP4	3.938873268	1.17E-09	5.99E-08
S100A4	3.934985515	0.002037233	0.016261072
FAM167A	3.921505945	1.71E-11	1.18E-09
CNTN6	3.911173301	0.004820617	0.031698227
CCL20	3.910490452	1.79E-06	4.27E-05
CPA4	3.899344591	1.26E-06	3.09E-05
ADAM33	3.88860403	1.48E-23	4.57E-21
CH17-360D5.2	3.883900087	0.000384728	0.004195522
BTBD11	3.881570494	2.39E-11	1.63E-09
ICAM5	3.881479721	8.37E-07	2.19E-05
RP11-308B5.2	3.875864387	0.004817888	0.031689528
RDH10-AS1	3.871399108	1.85E-09	9.12E-08
ARHGEF26	3.824350764	1.40E-23	4.45E-21
DAB1	3.812576052	0.000527889	0.005433398
CHMP4C	3.797892783	0.000356672	0.003944913
GPR4	3.786512642	9.16E-05	0.001301733
FAM84B	3.781979066	1.80E-07	5.72E-06

VWDE	3.78188013	0.001175616	0.010540799
LUZP2	3.774281027	2.48E-06	5.68E-05
LINC01629	3.773603795	0.006240266	0.038632746
MOB3B	3.766911606	0.000536563	0.005510094
STAB1	3.763859507	1.05E-06	2.65E-05
ANO4	3.736727627	0.000365775	0.004027817
KCNQ3	3.720827495	5.37E-13	4.86E-11
OLR1	3.72013455	6.20E-05	0.000930915
SYT9	3.719194041	0.005369676	0.034515169
GALNT6	3.716604411	2.60E-10	1.50E-08
RP11-501C14.7	3.715420593	0.005602526	0.035603762
MAP3K9	3.699002756	3.39E-12	2.61E-10
RP11-627G18.1	3.694955211	1.02E-05	0.000196413
RBM47	3.693630783	4.00E-13	3.71E-11
CXCL2	3.691397472	0.001517011	0.012894021
NDRG2	3.688014755	3.86E-07	1.12E-05
CTB-109A12.1	3.687041411	0.000159953	0.002057933
CLEC14A	3.680730901	0.000329071	0.003703237
PRICKLE2-AS1	3.677565999	0.002934299	0.021600929
CPNE5	3.672562065	0.000158352	0.002039661
RP11-893F2.5	3.670067187	1.57E-08	6.37E-07
GUCY1A3	3.654525205	0.005053006	0.032957121
MGAT5B	3.632233663	3.87E-08	1.43E-06
CYP2S1	3.61586648	0.001611558	0.013534322
CTB-47B11.3	3.60787267	9.64E-06	0.000186636
LYPD1	3.600590175	2.44E-06	5.61E-05
C9orf47	3.59849409	3.35E-05	0.000553598
CPNE7	3.592363678	2.22E-12	1.78E-10
GRIP1	3.584968895	1.50E-08	6.12E-07
PTGS2	3.580520153	1.95E-19	3.95E-17
GRIN2D	3.560975575	5.69E-20	1.25E-17
SYTL5	3.547052939	8.06E-06	0.000159376
BDKRB1	3.533080746	1.56E-10	9.31E-09
FSTL3	3.525009563	8.34E-28	3.76E-25
RP11-150O12.6	3.516840628	0.000435434	0.004640719
IGF2BP1	3.511861099	2.41E-09	1.16E-07
FCHO1	3.504176952	8.97E-05	0.001278786
CTC-296K1.3	3.502391713	2.24E-05	0.000392476
TMEM51	3.500462224	4.86E-12	3.61E-10
MGARP	3.497611647	1.07E-09	5.52E-08
SYT1	3.477704115	1.56E-09	7.80E-08
RP11-495P10.9	3.46182477	0.007198554	0.043001803
CTSC	3.46047151	9.27E-09	3.96E-07
LINC00337	3.456101738	0.007389056	0.043853797
RHOU	3.432900402	4.29E-13	3.94E-11
DOK2	3.428504622	0.000671179	0.006641369

ATP6V0A4	3.410886855	8.41E-09	3.63E-07
RP11-650L12.2	3.397341932	0.004214564	0.028580174
OGFRL1	3.397050031	2.45E-42	3.07E-39
IGSF3	3.386751424	1.01E-09	5.24E-08
FILIP1L	3.373769742	5.50E-17	8.38E-15
CAMK4	3.369033568	1.82E-26	7.73E-24
RP11-404P21.3	3.368368147	0.001364852	0.011855542
ZNF467	3.359406738	0.001430242	0.012314397
HSD17B14	3.345164753	9.85E-08	3.29E-06
ATE1-AS1	3.343646834	1.67E-05	0.000304231
GATA6	3.342836125	6.48E-09	2.83E-07
MATN2	3.340739286	3.03E-19	5.99E-17
CDH8	3.340732753	0.001629421	0.013643678
GPR63	3.335755615	1.72E-06	4.10E-05
PKDCC	3.33085532	7.74E-15	9.23E-13
AC015933.2	3.324027291	5.40E-05	0.000833297
CTC-296K1.4	3.320348014	0.000137341	0.001824276
ALDH1A3	3.311128252	4.34E-14	4.72E-12
IL32	3.30994985	0.000181329	0.002278341
LCP1	3.297455327	0.000108271	0.001494512
CSRP2	3.297096179	1.06E-11	7.50E-10
CLDN11	3.293847906	9.75E-21	2.29E-18
ITGA2	3.289132731	2.78E-09	1.32E-07
SPON2	3.268086524	2.33E-12	1.83E-10
CCND2	3.265231009	0.00039179	0.00426017
RDH10	3.262768599	1.80E-08	7.19E-07
GAB3	3.253022722	5.25E-17	8.10E-15
EML5	3.250561279	0.003309986	0.023753707
TMEM154	3.248877128	4.59E-17	7.18E-15
RASGRF2	3.237188123	2.12E-07	6.58E-06
GATA3	3.236083365	0.002678648	0.020096618
PIK3C2B	3.234893211	1.18E-06	2.92E-05
NIPAL1	3.233358911	1.05E-17	1.81E-15
HILS1	3.22870672	0.000190632	0.002375361
FRMPD1	3.224871066	0.000738422	0.007180658
AP1S3	3.22385939	6.53E-18	1.15E-15
PTPRB	3.221134462	1.21E-09	6.22E-08
RP11-4B16.4	3.206691812	5.08E-05	0.00079281
ZNF185	3.203501199	1.57E-13	1.55E-11
DEF6	3.17579169	8.81E-08	2.99E-06
RP5-1023B21.1	3.173822944	0.002078155	0.016511704
MYOM3	3.170073469	3.94E-05	0.000637479
C16orf74	3.168060603	5.15E-09	2.31E-07
AC007362.3	3.163789887	6.39E-07	1.74E-05
DNER	3.159152285	0.000743889	0.007225349
PRSS35	3.148359457	3.69E-08	1.37E-06

HMSD	3.146726493	2.60E-06	5.92E-05
HOXA11	3.14441313	6.90E-26	2.83E-23
AMIGO2	3.139125498	1.24E-09	6.34E-08
RP11-9G1.3	3.138634016	5.87E-05	0.000890882
SH3GL3	3.121068021	3.11E-06	6.92E-05
AC124789.1	3.115339471	3.47E-23	1.02E-20
STX11	3.104086974	5.86E-08	2.06E-06
SERPINB9	3.101184248	5.11E-08	1.83E-06
TNFRSF6B	3.100805711	1.12E-05	0.000212463
MB21D2	3.099228069	5.67E-19	1.08E-16
LINC01969	3.097954348	0.000115954	0.001588892
ISYNA1	3.090977215	1.92E-07	6.05E-06
STOX2	3.090298198	2.44E-08	9.48E-07
SH3TC1	3.088029323	1.06E-06	2.67E-05
KIAA1324L	3.087301346	1.10E-24	4.14E-22
EDARADD	3.084168066	0.000479378	0.005011901
DLGAP1	3.083076089	0.001278248	0.011246909
LLGL2	3.081188717	1.95E-06	4.60E-05
RP11-568J23.8	3.077626204	0.002866036	0.021160604
NPTX1	3.074687045	0.002825301	0.020962843
CDH2	3.070753614	1.77E-12	1.44E-10
ZBED9	3.066743713	3.75E-05	0.000609563
ATF4P4	3.064674647	6.83E-05	0.001008887
RGS2	3.058392449	2.22E-09	1.08E-07
MAL2	3.053680732	0.007315363	0.043533963
SLCO4A1	3.050490838	0.00031235	0.003554009
FBXO2	3.046909642	9.61E-08	3.22E-06
RP11-121A8.1	3.032230423	0.001246354	0.011019814
ELOVL2	3.03157651	1.81E-05	0.00032653
LYL1	3.025229095	1.49E-12	1.23E-10
GNAZ	3.020000208	1.56E-20	3.63E-18
LINC01828	3.019553158	0.004681534	0.030976466
GPRC5D	3.009288253	0.000690189	0.006796383
NFE2L3	2.99573201	3.88E-10	2.19E-08
TRIM36	2.994324678	6.87E-08	2.38E-06
ADGRD1	2.993933488	3.36E-09	1.57E-07
TFAP2A	2.992847942	1.00E-14	1.17E-12
DUSP4	2.974846091	7.83E-07	2.08E-05
SLC24A3	2.964847293	6.31E-10	3.44E-08
TPD52	2.962292414	0.001743871	0.014419885
HOXD9	2.956024715	3.44E-22	8.81E-20
RNA5SP37	2.955800275	0.005494468	0.035085215
LINC01583	2.954409363	0.001544727	0.013079657
HOXA11-AS	2.953009059	2.37E-09	1.14E-07
GNG4	2.94973344	0.000818882	0.007814739
RP11-184M15.1	2.947545093	5.63E-05	0.000862118

LPAR3	2.945422205	0.000179363	0.00225993
PTPRD	2.94426963	2.68E-17	4.28E-15
SLC7A5P1	2.941722944	0.001002994	0.009250609
PTPRR	2.940189957	0.001587185	0.013359496
RP11-277P12.20	2.933788087	6.20E-07	1.69E-05
DEPDC1B	2.924545364	0.005475885	0.035016149
ETS2	2.922130317	3.57E-15	4.47E-13
ITGA4	2.916748057	2.45E-16	3.43E-14
C5orf46	2.913853077	0.000712568	0.006983476
GSTT2B	2.910829125	9.67E-07	2.48E-05
RP11-465B22.3	2.897794365	0.002063478	0.016421382
RP11-756P10.3	2.896067223	3.54E-05	0.000577974
PLPP2	2.894345815	5.63E-07	1.55E-05
HTRA4	2.889710999	0.000302152	0.003455507
CORO1A	2.8884984	0.000239915	0.002858746
TMEM151A	2.886975515	4.04E-05	0.000651965
ARAP3	2.873595035	2.18E-09	1.06E-07
NTF3	2.871121415	0.000295818	0.003397199
GATA6-AS1	2.853631014	2.59E-07	7.87E-06
KCNA4	2.844806449	0.000326794	0.003683126
IL11	2.829431605	2.61E-08	1.01E-06
GCH1	2.826169077	9.58E-08	3.21E-06
RP11-627G18.2	2.816811103	2.95E-05	0.000496052
EPPK1	2.813008456	0.000498567	0.005176509
KSR1	2.800020727	4.18E-12	3.15E-10
ICOSLG	2.790648153	0.000351082	0.003896473
RGS4	2.789780286	2.60E-07	7.88E-06
HIST2H3PS2	2.783977404	2.41E-06	5.56E-05
DKK1	2.779581178	6.36E-05	0.000950328
TNFSF4	2.774497069	2.68E-06	6.07E-05
RP11-149I23.3	2.757618973	0.005066123	0.033033115
COL4A6	2.752689347	9.90E-06	0.000191359
NRP2	2.737674499	3.78E-07	1.10E-05
RP11-14D22.1	2.735301659	0.000118424	0.001614873
RP11-566K19.6	2.729426107	9.00E-05	0.001280727
GFPT2	2.727048674	4.13E-08	1.52E-06
ASRGL1	2.722585878	0.000118881	0.001620135
SNX18P7	2.721650511	0.00136112	0.011827681
NOMO3	2.721633704	6.55E-07	1.78E-05
TFPI2	2.712817096	0.00044176	0.004688227
CDCA7	2.711089025	0.002861252	0.021132204
CERKL	2.697629537	1.60E-08	6.44E-07
B3GNT5	2.683620666	1.16E-15	1.51E-13
CNKSR2	2.681100422	7.11E-07	1.91E-05
CH17-13I23.3	2.680077063	6.82E-08	2.37E-06
B4GALNT4	2.67928039	4.42E-06	9.42E-05

F2RL1	2.677568618	2.77E-05	0.000469377
HIST2H2BB	2.668916555	6.31E-08	2.21E-06
FBLN2	2.667906292	1.43E-07	4.64E-06
NAP1L4P1	2.667429293	0.004583311	0.030466653
ABC7-42404400C24.1	2.667386168	3.26E-11	2.17E-09
C15orf48	2.65547851	3.38E-05	0.000556694
RTN4R	2.645284608	0.000842474	0.00798579
STXBP6	2.633263985	0.000140718	0.001861462
FAT3	2.631875795	1.07E-05	0.000205526
PCDH7	2.627035307	3.07E-07	9.13E-06
HES4	2.626554664	0.000201219	0.002482584
C1orf226	2.625243633	0.000170962	0.002173522
PTPRD-AS1	2.604602079	0.001225762	0.010869358
ERICH5	2.60431872	2.63E-06	5.97E-05
PTPRF	2.601166343	5.29E-15	6.44E-13
ADAP1	2.60095481	0.000410429	0.004424432
C3orf52	2.599998539	1.03E-05	0.000198401
STK32A	2.587870951	0.00099348	0.009174118
LRRC8C	2.578023054	3.35E-06	7.36E-05
RP11-1069G10.1	2.575013537	0.000224133	0.00270315
SERINC2	2.571579273	2.02E-07	6.33E-06
COBLL1	2.565407458	3.72E-12	2.84E-10
TOX2	2.565391113	0.002437164	0.018730351
MELTF	2.563292136	1.38E-08	5.69E-07
RHPN2	2.559179105	2.25E-05	0.000394244
TNIK	2.550916039	1.72E-11	1.19E-09
PLPP4	2.544811068	3.26E-07	9.61E-06
IL27RA	2.540896235	5.25E-09	2.35E-07
SCG5	2.530262523	4.47E-08	1.63E-06
F2RL2	2.522092363	3.52E-06	7.70E-05
ERBB3	2.518578944	0.000430184	0.004602176
CFAP45	2.518047745	0.000959444	0.008907261
F10	2.517258609	8.17E-06	0.000161057
HSPA2	2.515896737	7.44E-06	0.000148443
AFP	2.515038647	9.29E-07	2.40E-05
CD55	2.514187593	8.70E-14	8.88E-12
EPHA4	2.50923257	7.66E-13	6.72E-11
CNKSR1	2.507551374	0.000437314	0.004654149
NRXN3	2.50613781	2.48E-06	5.67E-05
RP11-266K4.9	2.500848761	2.64E-06	5.98E-05
WFDC21P	2.489653744	5.23E-07	1.45E-05
NIPA1	2.47583297	7.42E-13	6.56E-11
CHRNA5	2.467645749	0.000291683	0.003357925
XDH	2.459573306	4.96E-06	0.000103724
RP11-359E10.1	2.45865724	0.000796525	0.007636946
RP11-229P13.25	2.453711451	0.008624939	0.049456817

SPTBN2	2.453207971	9.90E-10	5.18E-08
RP11-185E8.2	2.449760531	3.35E-05	0.000552613
NOS1AP	2.449159114	0.000560852	0.005733405
COL25A1	2.434803895	0.000206319	0.002524773
RP4-794I6.4	2.434035245	0.000388486	0.004232415
RBMS3-AS3	2.424095208	0.000110561	0.001523326
SDPR	2.422422485	8.86E-08	3.00E-06
N4BP3	2.422171593	0.008444252	0.048713958
KRT8P46	2.41466963	0.000252461	0.002982559
ATG9B	2.410168349	0.003138484	0.022754767
AFAP1L1	2.408304952	9.36E-12	6.74E-10
LINC00941	2.407703614	2.21E-08	8.63E-07
NDRG4	2.40446771	5.08E-09	2.29E-07
FAM19A3	2.399036721	0.001757167	0.014497915
RP11-497E19.1	2.392211033	4.14E-06	8.93E-05
SGK1	2.392195571	1.17E-18	2.18E-16
FGF2	2.389860414	4.86E-09	2.21E-07
AKR1B1	2.388015216	1.54E-10	9.22E-09
MYH15	2.383847813	0.001898813	0.015396095
FAM60A	2.368253437	1.76E-11	1.21E-09
Sep-06	2.367699956	2.34E-08	9.09E-07
RP11-73O6.3	2.362326143	0.001647534	0.013759562
CDC25A	2.359976722	0.00241712	0.018614383
ARHGAP4	2.353475577	6.66E-05	0.00098688
TRIM6	2.351402342	2.13E-08	8.38E-07
IFI27	2.347337396	8.97E-05	0.001278786
TESMIN	2.344228512	0.003430844	0.024326723
TCF21	2.338367611	0.000223051	0.002694424
DTX4	2.334531177	0.002388084	0.018449994
FAM43A	2.332179521	3.59E-05	0.000585352
CDK15	2.328603394	1.55E-11	1.09E-09
ETV4	2.322895312	2.89E-09	1.36E-07
MMP16	2.301308652	2.41E-11	1.64E-09
SYBU	2.299955015	5.76E-06	0.000118621
SLC7A7	2.299813186	0.000268723	0.003138488
FOXL1	2.298100022	2.30E-07	7.09E-06
EZR	2.296167529	1.62E-06	3.88E-05
RP11-527N22.2	2.295487354	6.30E-06	0.000128057
TM6SF2	2.28621396	5.96E-09	2.64E-07
FABP4	2.284805813	0.00301412	0.021994584
TMEM132A	2.28189654	0.001677506	0.013962113
EGF	2.268969726	2.00E-05	0.00035458
SLC7A5	2.263071555	5.24E-11	3.40E-09
PANX2	2.260704735	1.22E-19	2.53E-17
ADGRL3	2.258003581	3.67E-05	0.000597562
MDFI	2.255331611	0.005800364	0.03655186

CHST6	2.254140011	0.000224445	0.00270539
GATA2	2.246565073	6.33E-05	0.000946766
PLS1	2.244815211	1.14E-05	0.000216893
SLC12A8	2.232227991	0.000123037	0.001667694
FIBCD1	2.228985273	7.22E-11	4.56E-09
LRRC1	2.225637872	3.18E-19	6.23E-17
HOXD-AS2	2.223538073	3.58E-12	2.74E-10
RP11-757F18.5	2.222112006	7.56E-05	0.001102975
HMGA2	2.215896565	1.85E-05	0.000332647
GSG2	2.215622818	0.003787685	0.026310694
RPS6KA1	2.211575707	7.35E-12	5.38E-10
CYB5R2	2.208485566	2.41E-10	1.40E-08
TRPA1	2.198049259	0.00453453	0.0302226
CD274	2.196486815	2.39E-06	5.52E-05
PEG10	2.196207323	9.60E-06	0.000186274
CHN2	2.195685789	0.002647423	0.019985122
ANKRD18B	2.191987167	1.49E-05	0.000275125
PRPH2	2.189404937	0.003322906	0.023816096
TSPAN13	2.187511787	0.0080943	0.047133456
SCN9A	2.187119115	0.000188599	0.002353939
COL4A5	2.183916442	0.000170467	0.002168456
TLL1	2.182010724	0.000153639	0.002000208
ADAMTS3	2.176063743	2.72E-07	8.24E-06
IL31RA	2.170723594	7.01E-05	0.001032425
COL3A1	2.166200115	2.80E-07	8.45E-06
UCHL1	2.162588982	1.28E-10	7.80E-09
PABPC4L	2.160182004	1.71E-09	8.51E-08
ABCG2	2.155207013	7.42E-05	0.001086224
KLHL23	2.155101352	7.02E-06	0.00014103
HS3ST3A1	2.144740981	1.34E-06	3.28E-05
ARHGEF3	2.140217361	1.58E-05	0.000288964
SNORD22	2.134400873	0.002063874	0.016421382
DNM1	2.124222346	4.81E-06	0.000101381
RPS6KL1	2.118079394	0.000729626	0.007110461
BIRC3	2.113836128	0.000322751	0.00364851
BOLA2	2.111952922	1.14E-06	2.83E-05
RP11-214O1.2	2.107576185	0.006100898	0.037999544
FRMD3	2.102729925	0.001316944	0.011519296
RP11-673E1.3	2.09837093	0.001511575	0.012852661
GBGT1	2.094545871	0.000181162	0.002277508
DUX4L50	2.093555963	9.35E-07	2.41E-05
RAB33A	2.091381306	5.50E-06	0.000113636
FBN2	2.090779347	2.18E-06	5.09E-05
PKP3	2.08792743	0.001297774	0.011386972
NR2F2-AS1	2.084336866	2.12E-05	0.000373568
SAMD12	2.082834792	6.06E-05	0.000913171

GLIS1	2.082087348	5.11E-12	3.76E-10
RP11-177C12.1	2.081211854	0.00266977	0.020081327
PLEKHN1	2.067712885	0.002968845	0.021755762
ERBB4	2.067562493	0.001067184	0.009735085
ATP2A3	2.066375551	0.004485001	0.029998931
CSMD2	2.064524659	0.001929709	0.01557953
GCSAM	2.064416039	0.00048668	0.005071777
FLRT3	2.064042373	4.34E-07	1.24E-05
CARD9	2.063753404	3.28E-09	1.53E-07
ZNF300P1	2.061529395	0.000135304	0.001804666
SLC4A11	2.056874385	6.34E-05	0.000948809
DFNA5	2.055072678	3.83E-06	8.32E-05
SLC8A1	2.054342119	0.00509057	0.033182858
TSPAN15	2.053396993	2.71E-05	0.000461216
CCDC85C	2.051819339	1.43E-12	1.19E-10
EAF2	2.047758945	2.81E-05	0.000475178
CDH18	2.046703017	0.004449809	0.029807773
BCO2	2.045788056	0.000167378	0.002135794
RAD18	2.044234465	2.05E-05	0.000363442
NOX4	2.03639811	0.000497507	0.005167883
ATP11A	2.036366553	9.17E-16	1.20E-13
VEPH1	2.033636044	1.15E-11	8.10E-10
U52111.14	2.033593992	9.08E-06	0.000177358
LINC01605	2.033526886	2.34E-06	5.42E-05
RFLNB	2.029140604	1.58E-12	1.30E-10
DSG3	2.024309466	0.005538719	0.035287809
PHLDA2	2.020507078	4.27E-11	2.79E-09
CGNL1	2.01462665	0.000158075	0.00203929
ARHGEF19	2.014048637	2.63E-05	0.000449695
RP11-20I20.4	2.012396868	0.001260752	0.011127097
FILIP1	2.008346616	4.72E-07	1.33E-05
LINC01615	2.006398377	1.96E-08	7.76E-07
RGS5	1.99777734	0.000121497	0.001649794
CKS2	1.991796915	0.002111997	0.016704045
SNORD17	1.986989783	0.000802544	0.007678334
SIM2	1.985361932	0.000174321	0.002206267
STARD4	1.982425517	4.63E-07	1.31E-05
KLHL29	1.981630053	5.12E-14	5.50E-12
MT1L	1.976560174	0.002382762	0.018431653
MIR31HG	1.97141786	0.000932533	0.008707632
RP11-1148L6.9	1.969830857	0.005745261	0.036275608
MT-TR	1.967144694	0.005630555	0.035731514
PRSS3	1.965109335	0.002589639	0.019634394
RP11-649A18.12	1.964298059	0.00536329	0.034506635
KRT80	1.957436864	0.001694175	0.014081269
TCEAL7	1.95479307	4.47E-08	1.63E-06

SH2D5	1.954611936	0.000300599	0.003441239
TRPV3	1.952439027	1.39E-09	7.06E-08
PAPSS2	1.948733636	2.49E-14	2.78E-12
SLC25A23	1.948415505	0.000401403	0.004337496
RPP25	1.94641028	1.31E-06	3.22E-05
RASSF7	1.944289631	6.33E-10	3.44E-08
COL4A1	1.940684958	0.000396727	0.004297706
IFNWP19	1.936768599	0.003342111	0.023895967
KIF5A	1.935362793	0.001125162	0.010157099
CBS	1.930404547	7.04E-06	0.000141385
RGAG1	1.923600931	6.20E-07	1.69E-05
CCDC138	1.922061884	0.001301488	0.011406235
KCNG1	1.918337582	8.27E-05	0.001197356
SDC1	1.915831753	0.000140486	0.00185948
LINC01638	1.91561746	0.001050283	0.009604233
CENPV	1.910709876	6.75E-10	3.65E-08
TBX3	1.903829178	7.47E-13	6.58E-11
SEMA6A	1.901886813	5.01E-05	0.000783904
SRGAP2D	1.900551442	2.46E-05	0.000425738
FLNC	1.896628903	0.001936941	0.015626552
CNTN1	1.895010155	8.42E-06	0.000165681
PPM1J	1.892362661	0.000466694	0.004902026
STK26	1.889128206	6.95E-12	5.10E-10
CCDC8	1.887931431	0.000732923	0.007136418
MYCL	1.884725407	8.55E-05	0.001227664
PLEKHA7	1.884166959	0.001060939	0.009685953
FANCB	1.883542343	0.003353429	0.023940726
CCNF	1.878434523	0.00520023	0.033731912
DMD	1.878000089	0.00058	0.005906841
AC068499.10	1.876656528	0.006696851	0.040710278
VLDLR	1.874999549	1.92E-06	4.53E-05
RARB	1.873632063	3.34E-06	7.35E-05
CEP128	1.871589111	0.003555094	0.025018846
NR2F2	1.870579476	2.59E-05	0.00044339
PRDM1	1.862923388	0.000136219	0.001812475
ANKLE1	1.856787555	0.001409244	0.012170795
ONECUT2	1.854960143	0.00373265	0.02602195
ODC1	1.852932289	4.94E-05	0.000777493
PFKFB3	1.845665003	3.43E-05	0.000564989
EIF4A1P10	1.845487589	0.004611358	0.030598949
PBX1	1.844306914	0.000156373	0.002026913
PID1	1.841835679	4.72E-07	1.33E-05
AMPD3	1.840501897	0.002151603	0.016940025
MBNL2	1.838971248	9.24E-12	6.68E-10
RP11-346C4.3	1.837959219	0.004930378	0.032297485
KLHL4	1.837462065	0.005541572	0.03529601

SHROOM2	1.836675246	2.35E-07	7.22E-06
ALG1L13P	1.833866919	0.007462979	0.04420355
TRIM29	1.828376748	0.004010572	0.027511355
RBM24	1.825460061	0.008236465	0.047776163
AP003068.23	1.823231439	1.12E-06	2.79E-05
RP11-380J14.1	1.819910149	0.000669075	0.006626375
LRRC37A15P	1.814448117	0.000190125	0.002370357
SEL1L3	1.809530394	2.72E-14	3.01E-12
FSD1	1.801591879	5.68E-07	1.56E-05
CBX2	1.800917769	0.000595076	0.006031295
NUAK1	1.798548696	0.000338562	0.003787361
CH507-9B2.5	1.796647308	0.000100576	0.001403771
ISG15	1.79042669	0.000668396	0.006625471
EFNB2	1.787541366	1.52E-09	7.67E-08
RP11-283G6.3	1.787117538	0.000134663	0.001797187
NSFP1	1.779246646	0.000976098	0.00903953
MT1E	1.772822905	3.29E-06	7.26E-05
DNMBP	1.771645735	1.90E-05	0.000340273
BCAT1	1.764142598	1.47E-23	4.57E-21
RAC1P2	1.75713918	0.006790526	0.041168708
PITX2	1.756450201	6.59E-11	4.21E-09
FAM129A	1.75600473	0.002534152	0.019311131
ENC1	1.753869772	0.000634774	0.00635648
BDNF	1.750889595	0.002119646	0.016745498
BHLHE41	1.748900017	0.000200075	0.002471111
MIR137HG	1.74606472	0.000325439	0.003673367
GPSM3	1.73858943	1.98E-05	0.000352806
FLRT2	1.737948655	0.000632513	0.006342288
GFOD1	1.735911323	4.86E-06	0.000102175
MLLT11	1.729716789	3.46E-06	7.59E-05
AFAP1-AS1	1.729241541	5.35E-05	0.000826451
SLC22A4	1.727241367	3.59E-06	7.85E-05
PTPRN	1.724890465	8.36E-05	0.0012073
KPNA2	1.724815007	0.001585569	0.013350884
THOC3	1.724241687	5.72E-08	2.02E-06
JUP	1.720412316	0.00034283	0.0038275
TYW1B	1.717633959	0.00291481	0.021478503
MARS2	1.715994106	4.05E-12	3.07E-10
CELF2	1.715412354	1.37E-06	3.35E-05
ASPHD1	1.710202052	1.60E-07	5.13E-06
RNF207	1.709822427	0.000728607	0.007106096
TSPAN9	1.708376937	5.13E-05	0.000798351
MT-TH	1.706835749	0.007457487	0.044201737
RP11-549B18.1	1.706572206	0.000509277	0.005270709
RP11-557N21.1	1.701936139	0.000984548	0.009102826
NCAM1	1.697239314	1.14E-05	0.000217427

FAM86B3P	1.692801532	0.000139922	0.00185528
TPPP	1.692211392	0.006219037	0.038554424
FAM86FP	1.69160218	0.000691103	0.006799716
LINC00702	1.690242055	0.003141157	0.022759508
PPIL1	1.688276232	9.25E-06	0.000180032
PGM2	1.68816907	1.40E-06	3.41E-05
PTK2B	1.686262627	0.00181503	0.014844921
TYMP	1.68280174	0.003481028	0.024620039
SKP2	1.680889347	1.36E-05	0.000253381
EPHA2	1.680091104	7.30E-14	7.59E-12
SLC45A3	1.677622265	6.17E-06	0.000125927
NEK5	1.676130296	0.000101704	0.00141688
MFSD2A	1.676097628	3.57E-11	2.37E-09
ADAMTSL5	1.675331179	5.33E-07	1.48E-05
ADORA2B	1.671947547	2.97E-08	1.13E-06
KIF17	1.668207045	0.000209454	0.002556199
RPL7AP28	1.661481386	0.002228999	0.017454043
AGRN	1.66009744	0.000738003	0.00717968
HSPB8	1.656917116	1.59E-06	3.82E-05
NUDCD1	1.653855578	2.98E-08	1.13E-06
GPR3	1.650777266	0.000417311	0.004487886
DNAH11	1.650620657	0.000434174	0.00463054
NRIP1	1.650484099	2.55E-14	2.84E-12
PSD4	1.648804296	0.002284395	0.01781749
TWIST2	1.6473241	3.35E-09	1.56E-07
PCDH9	1.645845058	0.001010219	0.009286847
ZNF711	1.645680583	0.003749066	0.026114864
ARHGAP20	1.640601013	6.64E-05	0.000986518
CTPS1	1.638024987	0.000156696	0.002028766
DPYSL4	1.633819625	9.06E-08	3.07E-06
DCBLD1	1.628414353	4.33E-07	1.24E-05
SCRG1	1.62149538	0.008455518	0.048745736
SARDH	1.62138383	0.000907865	0.008519646
TMEM25	1.621080088	4.61E-08	1.67E-06
CORO2A	1.620380976	9.27E-05	0.001315725
RP4-584D14.5	1.619654778	0.00612145	0.038085453
PLS3	1.616094463	0.000187618	0.00234429
CHN1	1.613234449	1.52E-05	0.000280073
PROCR	1.609871036	1.78E-05	0.000320842
TAGLN2	1.607422058	9.32E-05	0.001321662
STK33	1.606114497	0.000206095	0.002523406
LINC01094	1.604488431	0.007933428	0.046448675
RP11-154D6.1	1.60302054	0.00296181	0.021711272
GK	1.602454635	1.05E-08	4.42E-07
DPF1	1.601475807	0.002353308	0.018247648
ELOVL5	1.601046411	1.99E-08	7.85E-07

ARMC4	1.595299666	0.000277251	0.003218079
MT2A	1.594370273	0.006378115	0.03922758
RHOJ	1.591507029	0.003008935	0.021978095
KCTD16	1.59038007	0.008269349	0.047942229
ISOC1	1.589331107	2.94E-11	1.98E-09
CLIC3	1.588490002	0.007273808	0.043363903
GAPDHP1	1.587159816	7.45E-05	0.00108955
PIK3AP1	1.58646948	0.004115799	0.028096376
RP13-516M14.8	1.584671634	0.007137023	0.042706565
LIPA	1.581402912	3.17E-07	9.37E-06
GCA	1.576313263	0.000171685	0.002181477
SFXN2	1.574668248	0.001518514	0.012901928
AC004057.1	1.573273713	0.001368089	0.011874508
CCDC50	1.569826584	3.95E-08	1.45E-06
COL4A2	1.568547058	0.003937085	0.027089217
ITSN1	1.564241204	3.55E-12	2.73E-10
PAPPA	1.560874982	0.00012507	0.001689129
APCDD1L-AS1	1.560420324	0.003430575	0.024326723
MECOM	1.559960135	6.35E-09	2.79E-07
NDUFA12	1.558692649	2.08E-07	6.48E-06
CHSY1	1.555895185	1.91E-08	7.59E-07
CCNJL	1.553251172	0.000156501	0.002027405
HIST1H3E	1.546656047	0.004572486	0.030412633
NDC1	1.543795913	0.000715944	0.00701047
HSPB6	1.541928959	9.45E-05	0.001332332
TRIM24	1.541717616	4.89E-20	1.08E-17
HOXC11	1.53783791	2.71E-05	0.000460747
ST3GAL1	1.53382038	4.45E-12	3.34E-10
SMG1P3	1.53078745	2.14E-05	0.000376309
ULBP1	1.530475941	0.000510367	0.005279567
TLE1	1.529364583	3.12E-07	9.25E-06
DBF4	1.527144646	0.002732567	0.020422677
CCDC81	1.526200495	0.002835691	0.021006137
PLA2G4C	1.523483076	0.000668242	0.006625471
GRK5	1.522570026	6.76E-06	0.00013656
CSRP1	1.517109269	0.000149825	0.001957795
HOTAIR	1.516840018	6.99E-06	0.000140624
KCNAB3	1.515991979	0.007010808	0.0421865
BMP4	1.515408003	3.70E-08	1.37E-06
LNCSRLR	1.51331974	0.001778716	0.014615074
PGAM1P8	1.509917073	0.000250794	0.002964415
SLC17A9	1.509855761	0.002547699	0.019388644
SUPT3H	1.509476817	5.59E-07	1.54E-05
B4GALT4	1.508474384	0.000414301	0.004457638
SLC38A5	1.507076889	0.002958162	0.021698646
TNFRSF21	1.505669236	0.004410989	0.029618144

CALB2	1.505499329	2.64E-05	0.000449892
MEIS2	1.504957666	4.02E-10	2.26E-08
CALM2	1.504490823	0.000285268	0.003300933
MICAL2	1.504418591	9.07E-06	0.00017723
PDE10A	1.503969634	0.003031097	0.022089868
SYT15	1.502599711	1.15E-05	0.000218224
BZW2	1.502113495	1.96E-06	4.61E-05
TXNRD1	1.498414205	2.75E-08	1.06E-06
TENM3	1.497318071	0.000363987	0.004010084
IKBKE	1.492657197	0.000156912	0.002029882
PRICKLE2	1.490375989	5.56E-13	5.00E-11
XRRA1	1.484853508	1.54E-10	9.22E-09
ATP2C1	1.484005436	1.50E-15	1.90E-13
MEIS1	1.483890186	3.62E-07	1.06E-05
SH3RF2	1.482913363	0.00580222	0.036553334
GGH	1.482850126	0.003318892	0.023794895
SLC35F2	1.481887475	0.000229919	0.002754522
KIFC3	1.480415598	2.79E-13	2.64E-11
FABP5	1.480198632	0.001723292	0.014270652
POLR3G	1.478944975	1.21E-05	0.000226817
GTF2A1L	1.477127524	3.95E-05	0.000637785
GDNF	1.475758444	0.00012663	0.001706091
CHD7	1.473229036	0.000176673	0.002233533
CCDC89	1.472673025	0.000432145	0.004618764
WDR4	1.46916668	2.61E-08	1.01E-06
CDC42EP2	1.466609286	0.00020018	0.002471111
SIGIRR	1.464959125	1.40E-06	3.41E-05
PRR15	1.464904083	0.006266651	0.038740612
TUBA1C	1.464220116	0.008155309	0.047410625
ECE2	1.46356932	4.61E-08	1.67E-06
SLC9A3R1	1.463121927	0.000218993	0.002652507
SIRPA	1.456292275	5.25E-05	0.000812731
FZD8	1.455050381	3.74E-08	1.38E-06
TNFRSF10A	1.453668057	3.54E-07	1.04E-05
NCR3LG1	1.452919241	0.001783871	0.01464854
H2AFZ	1.452578256	0.000648497	0.006471609
NT5DC2	1.450899654	4.00E-16	5.46E-14
HIVEP3	1.449604968	1.03E-06	2.62E-05
PSORS1C1	1.4485952	0.000172876	0.00219168
ULBP2	1.446894778	0.000508918	0.005269417
RP11-423H2.1	1.444793714	0.005416713	0.034726432
C8orf88	1.443158927	9.38E-05	0.001323735
B3GNT8	1.442773374	0.003482041	0.024620039
PTPRJ	1.442338147	2.31E-06	5.35E-05
MOCOS	1.44183649	0.000235931	0.002815307
EBNA1BP2	1.441427826	7.62E-05	0.001109574

CD99P1	1.440930516	0.000106264	0.001471316
RALGAPA1P1	1.439839697	0.006080135	0.037891158
SENCR	1.433131233	0.001305066	0.011428706
TMTC2	1.432705742	2.68E-06	6.07E-05
FAM196B	1.4309883	0.001357929	0.011809055
RP11-756P10.6	1.430335055	0.000149813	0.001957795
CPNE2	1.429976324	5.61E-07	1.55E-05
PKIA	1.429520616	0.003915869	0.026993154
CCDC86	1.42735914	5.03E-09	2.27E-07
CUX1	1.427297308	2.58E-06	5.89E-05
ARHGAP5-AS1	1.425124191	1.41E-06	3.42E-05
TNFAIP3	1.424024019	0.001250541	0.011049955
ENTPD7	1.421982175	2.97E-08	1.13E-06
MYH10	1.420590232	7.27E-07	1.95E-05
SAMD3	1.41906317	0.006242343	0.038634998
BMPER	1.41870971	0.002667117	0.020081327
COL5A2	1.41831824	3.44E-05	0.000565249
VPS9D1-AS1	1.417615026	0.000170319	0.002167795
AP000695.4	1.417078774	0.000708135	0.006949094
NDUFV2P1	1.41634292	0.002407281	0.018557631
SORBS1	1.414085185	0.001191597	0.010641758
INTS6L	1.41237263	0.000367826	0.0040425
SLC29A1	1.407718173	3.99E-06	8.62E-05
TMEM200A	1.407032305	0.002607514	0.019743356
LRP8	1.40612499	0.000124299	0.001681771
FAR2	1.403557899	1.46E-05	0.000270279
GALE	1.402401704	1.12E-06	2.80E-05
LSM11	1.399487763	6.11E-09	2.70E-07
FSCN1	1.398995419	0.001082429	0.009838323
TCHP	1.397734993	2.20E-05	0.000385896
ARL5B	1.396561905	8.70E-11	5.44E-09
IL1RAP	1.386702072	0.000105091	0.00145776
ERN1	1.382223277	0.00180473	0.014771396
ANP32E	1.379608079	0.002880447	0.021248402
ARHGDIB	1.377994462	0.002305821	0.017928282
GADD45A	1.377925046	0.000203912	0.002507576
XRCC4	1.377126223	3.22E-05	0.00053432
RAB3IP	1.371978185	0.000338292	0.003786214
RP11-676J12.7	1.371905679	0.004935449	0.032314111
GLS	1.371582191	2.00E-05	0.000354655
SLFNL1	1.370821081	0.008030344	0.046833631
SHB	1.370419566	4.90E-07	1.37E-05
ST7L	1.368587669	5.46E-08	1.94E-06
ARL4A	1.36789869	9.34E-05	0.001322319
SLC38A1	1.367824508	1.33E-08	5.47E-07
UBE2S	1.367085862	0.003618625	0.025370896

TMEM164	1.366994528	1.06E-08	4.47E-07
MLKL	1.366165077	0.000159941	0.002057933
CCZ1	1.364964286	7.11E-06	0.000142383
NUAK2	1.359844854	0.000259386	0.003053175
TDRKH	1.356366421	3.74E-05	0.000607688
PRSS12	1.355080407	0.005302296	0.034265787
OPHN1	1.351705516	1.13E-06	2.82E-05
SAMD10	1.350641466	0.001500479	0.012782429
NUP35	1.347615997	0.004174899	0.028353839
CEP170P1	1.34701909	0.003564639	0.025070365
SLC25A24	1.34478482	1.82E-07	5.75E-06
HPRT1	1.344058057	0.001092365	0.009912641
NHSL1	1.343735095	5.61E-05	0.00086006
CNN1	1.34281652	0.003823763	0.026504133
SMG1P6	1.342146071	0.001715836	0.014229822
AMMECR1	1.341377372	1.99E-08	7.85E-07
ZNF703	1.339748143	0.004971289	0.032508797
C17orf97	1.338137544	3.37E-06	7.41E-05
PRKCZ-AS1	1.337868408	0.00267068	0.020081327
PCYOX1L	1.336776818	0.001897104	0.015393313
SOGA3	1.336318034	1.32E-05	0.000245372
RNFT2	1.33485183	0.005002962	0.032668533
SARM1	1.332465324	2.76E-06	6.20E-05
VWF	1.331081124	0.008432166	0.048686594
RP11-66B24.4	1.329680702	8.93E-05	0.001275323
PSPHP1	1.327979486	8.10E-07	2.14E-05
PROSC	1.326631556	5.55E-07	1.53E-05
TET1	1.325197583	0.000673245	0.006658893
ATP7B	1.323810281	0.000129423	0.00173753
ATP1B1	1.319651332	0.002383643	0.018432145
NOLC1	1.317451225	6.60E-07	1.79E-05
FAM50B	1.317185861	5.25E-08	1.87E-06
LPCAT2	1.317007871	4.25E-08	1.56E-06
KDELC1	1.314247935	1.03E-06	2.62E-05
FKBP1B	1.313903619	0.001508562	0.012836018
HEG1	1.313461707	0.003336175	0.023875227
TMPPE	1.31064421	0.002081115	0.016529393
PPAT	1.308513858	2.58E-05	0.000442249
CSRNP1	1.306063066	0.000449634	0.004754352
MPV17L2	1.301433101	6.93E-05	0.00102289
RRP7A	1.297334637	3.00E-05	0.000502033
SYNGR2	1.296296388	0.000136274	0.001812475
CHAC2	1.29465362	0.003568867	0.025092275
DDX21	1.294082634	8.05E-06	0.000159271
USPL1	1.293539225	1.49E-06	3.59E-05
NRAS	1.292011479	8.67E-05	0.001242224

PLEKHG3	1.290011639	0.001761526	0.014517936
TOMM40	1.288721214	0.000156158	0.002026454
SLC16A1	1.288676042	7.12E-10	3.83E-08
ITGA1	1.288210085	0.000182412	0.002289388
BCYRN1	1.287856912	0.005357569	0.034494422
CKAP4	1.286665758	6.78E-05	0.00100161
LYAR	1.285791617	0.001277247	0.011246257
SPRY4	1.284052376	0.00224942	0.017585556
PDCD6IPP2	1.281147272	0.006581836	0.040184497
LYSMD2	1.27866461	0.000914186	0.008564704
SNX24	1.276309749	7.73E-08	2.66E-06
RAI14	1.273895419	7.46E-15	8.94E-13
NAA15	1.268070561	0.000233248	0.002786242
SEMA4B	1.258058265	1.26E-05	0.000234843
PPP1R13L	1.257865223	0.00147674	0.01263751
PUM3	1.254820224	8.18E-06	0.000161154
ANKRD50	1.250946776	1.12E-05	0.000212828
LOXL2	1.250714789	0.000116822	0.00159787
LMO4	1.241992765	2.00E-06	4.69E-05
CEND1	1.241938678	0.004603053	0.030570838
RRN3P1	1.23963875	8.56E-05	0.001227664
KIAA1324	1.238018401	0.000566964	0.005793266
SLC19A1	1.236795757	0.000830534	0.007896304
SSFA2	1.232425738	0.000151287	0.001972839
PLXNB3	1.232388438	0.004953321	0.032403267
KLHL5	1.231730184	0.001161488	0.01044685
ADARB1	1.230493948	0.001504735	0.012813836
TUBB6	1.228429114	0.002585581	0.019610276
NOL4L	1.220095987	6.87E-06	0.000138576
QTRT2	1.218631987	2.48E-05	0.000427544
DYRK4	1.218062634	0.000769911	0.007438732
RAC3	1.217232947	0.002946858	0.021668589
LYPLA1	1.216713815	3.94E-06	8.53E-05
ZFPM1	1.2162113	0.005684774	0.035994523
POLR3B	1.212319636	2.90E-05	0.000489052
PLK2	1.212202636	0.00030959	0.003529825
ZNF850	1.209636725	0.000363785	0.004009813
CCT5	1.208521777	0.000204029	0.002507637
YRDC	1.207866198	1.54E-06	3.71E-05
TRIM46	1.207621072	0.000167579	0.002136312
LINC00857	1.206971697	1.23E-06	3.04E-05
RHOF	1.202326861	0.00220029	0.017257045
MAN1B1-AS1	1.201665463	0.00620858	0.038526448
SLC25A15	1.200974301	0.000212386	0.002589177
CD58	1.199337728	8.61E-05	0.001234154
ARID3A	1.193237589	3.04E-11	2.04E-09

KCNIP3	1.192510686	0.00651859	0.039906449
SMS	1.191722136	0.000377772	0.004129473
ABCE1	1.191187892	0.000114727	0.001573987
FLI1	1.190441638	2.52E-08	9.76E-07
ASAP1	1.189994477	3.07E-06	6.83E-05
KIF21A	1.189309601	0.000101018	0.001409064
RPP40	1.188353047	0.000463932	0.004875283
GRK3	1.186726991	5.53E-06	0.000114038
L3MBTL3	1.186444079	6.87E-11	4.35E-09
DNAJC9	1.185862121	0.00492926	0.032297485
SPATS2	1.185684215	1.54E-08	6.26E-07
TPMT	1.185607569	0.008024132	0.046809514
RUVBL1	1.185322791	6.62E-05	0.000983429
PPME1	1.185312579	0.000140415	0.00185948
MIR503HG	1.185211745	0.001260659	0.011127097
WEE1	1.184996576	0.000992419	0.009168077
SPRYD7	1.181879229	0.000931373	0.008700408
SNX25	1.181289123	1.84E-05	0.000330694
TTC27	1.178668639	9.47E-05	0.001333957
UTP20	1.178054395	2.63E-05	0.000449551
PXYLP1	1.176030415	2.59E-06	5.89E-05
MEIS3	1.175834509	1.67E-05	0.000304102
AACS	1.173347228	0.000120559	0.001640018
POP1	1.168976972	0.00053908	0.005530909
LRRC8D	1.167443862	2.30E-05	0.000401497
NECTIN2	1.165606666	1.77E-09	8.79E-08
WASH3P	1.165224939	0.001798867	0.014728755
USB1	1.164499368	3.30E-12	2.55E-10
RP11-500C11.3	1.164106095	0.00710518	0.042549911
QPCT	1.164073219	0.002038272	0.016263604
ATG7	1.164014541	2.28E-12	1.80E-10
AASS	1.161924389	0.004448348	0.029806839
CYCS	1.155434221	0.000151326	0.001972839
YOD1	1.154234008	2.75E-06	6.18E-05
GPN3	1.15219285	0.005140783	0.033432891
UCK2	1.150525707	4.11E-13	3.79E-11
OPN1SW	1.149754429	0.00484011	0.031790816
MCC	1.148714745	0.000458063	0.004822585
INPP5F	1.145201406	0.000223764	0.002701588
PPP1R15B	1.143079832	1.62E-07	5.17E-06
GBP3	1.141896647	0.000598401	0.00605955
PRELID2	1.140842522	0.001779147	0.014615074
SLC2A6	1.138190124	0.007135543	0.042706565
SAMD14	1.137871238	0.004084557	0.027898642
DDX18	1.137660355	0.000298328	0.003420459
DCHS1	1.134580426	0.008620823	0.049445791

GULP1	1.133680851	0.000717532	0.007016871
ACTL10	1.13103076	0.0013106	0.011472712
POLR3K	1.130882545	0.00160197	0.013473881
DOK4	1.129134025	0.000189082	0.002358658
PLEKHA2	1.128956914	3.09E-06	6.88E-05
TFRC	1.127710063	1.04E-11	7.39E-10
TAX1BP1	1.127366789	1.53E-08	6.25E-07
APAF1	1.125246608	0.00137705	0.011933907
TTLL12	1.122645519	8.89E-07	2.31E-05
HSPD1	1.121999005	8.78E-07	2.28E-05
SLC10A7	1.121781881	0.000784591	0.007548211
AC002398.13	1.119051892	0.005250362	0.034007767
IQCB1	1.118692866	0.001912632	0.015469193
MPP1	1.117965427	0.000434274	0.00463054
SLC39A10	1.117789844	0.003586862	0.025187372
ECE1	1.117707264	0.000393986	0.004279918
NOL6	1.116310739	0.000152851	0.001991573
TDG	1.116251088	5.94E-06	0.000121929
COPS6	1.116066509	0.000372862	0.00408788
PTGR1	1.115890562	7.45E-07	1.99E-05
TUBB2A	1.115815693	0.006844511	0.041429138
ZBTB18	1.112646394	2.14E-08	8.39E-07
LMNB2	1.112055268	0.002764889	0.020609578
B4GALT5	1.112045805	0.000316308	0.003581067
NECAB3	1.111044926	0.000449682	0.004754352
HOXC8	1.109221217	0.002503498	0.019122792
RRS1	1.106615442	5.06E-07	1.41E-05
GSTO1	1.105309446	0.000205119	0.00251419
NOP16	1.103114384	0.000330992	0.003721142
NBPF1	1.102166878	4.15E-06	8.93E-05
ALYREF	1.101268809	0.007208694	0.043021234
RP11-755F10.1	1.101184528	0.002750571	0.020536804
KIAA0408	1.099921166	8.08E-05	0.001172078
CASP10	1.098885008	0.003806757	0.026410619
CEP170	1.097999493	0.000924024	0.0086425
FKBP4	1.097926993	2.25E-09	1.08E-07
MTAP	1.09636793	2.01E-07	6.30E-06
GOT2	1.094230031	3.61E-06	7.88E-05
AEN	1.093795532	3.95E-09	1.81E-07
FBXL6	1.091803623	0.002111375	0.016704045
SLC19A2	1.091295294	0.007317899	0.043534641
TOM1L1	1.09122527	0.008690622	0.049719624
LRRC37A2	1.085960994	8.61E-05	0.001234154
CLOCK	1.085698158	0.002983834	0.021836529
PLEKHA8P1	1.085192743	0.000976792	0.00904225
NOP58	1.085077027	0.001027126	0.009419221

MT1X	1.0837847	0.005131407	0.03338154
NDFIP2	1.081905969	0.001170669	0.010513164
FAM216A	1.08184246	0.000119317	0.001624109
MICA	1.081354296	0.003157523	0.022841373
TMED5	1.080931424	1.24E-06	3.06E-05
RAPGEF6	1.080406888	6.94E-05	0.001023702
SERPINB8	1.080390362	6.41E-05	0.000956106
ATF5	1.079475159	0.000106068	0.001469506
MYEF2	1.077814459	0.007056329	0.042358645
FGD6	1.077522518	0.00200547	0.016075854
ABCA5	1.077173192	2.67E-05	0.000455173
TMEM2	1.076220705	0.000227179	0.00272966
SHROOM4	1.076153776	0.000255015	0.003008002
PITX1	1.075923063	0.00257387	0.019550348
HMBS	1.075749008	0.003887189	0.026861169
FAM49B	1.074167304	2.17E-05	0.000381926
RRP9	1.073543473	1.59E-05	0.000290786
STAG3L4	1.070080716	5.36E-05	0.000828032
PAK1IP1	1.069568663	0.000194731	0.00241443
NOP2	1.068639717	3.70E-07	1.08E-05
RPS26	1.067888106	3.33E-06	7.34E-05
NUP88	1.066505884	0.007714877	0.045404971
NBEA	1.064930184	0.000649485	0.006477894
FARSB	1.064796037	0.00080103	0.007670356
PIM3	1.064593757	0.002059733	0.016400066
ACSL4	1.063280724	4.17E-06	8.96E-05
KATNAL1	1.062339274	0.007476789	0.044269582
LRP2BP	1.058995652	0.002666164	0.020081327
PRPSAP2	1.058099224	3.34E-08	1.26E-06
CCT2	1.058084412	0.000194175	0.00240905
ITGB3	1.05674215	0.001949029	0.015701595
ATP10D	1.05577076	0.000513764	0.005304968
OAF	1.053422825	0.001188561	0.010623055
DNAJB11	1.052908411	0.000422076	0.004530483
ECI2	1.050539592	0.004085596	0.027898642
PSMA5	1.049545149	3.10E-05	0.000515842
ELOVL6	1.047220356	7.74E-07	2.06E-05
PDCD11	1.047033824	6.23E-06	0.000127033
PDPK2P	1.046852924	0.008132049	0.047304392
SLC25A4	1.04593443	9.38E-05	0.001323709
GCAT	1.045821473	2.64E-05	0.000450786
DKC1	1.045646497	0.000263286	0.003084638
DAAM1	1.043942463	0.006393518	0.039290153
MPP5	1.04076587	0.002452682	0.018804731
PAQR3	1.039873292	3.47E-08	1.30E-06
KCNK6	1.039363849	1.47E-05	0.000271744

NOC2L	1.03922582	0.00019199	0.002387005
ATG4D	1.037860065	5.74E-05	0.000875451
SLC4A2	1.036712775	1.14E-06	2.83E-05
TBL1X	1.035304167	0.000529208	0.005444493
SEC23B	1.035056939	0.000172574	0.002189074
CCDC134	1.031706858	0.006056477	0.037764608
ATP6V1C2	1.031396009	0.007846985	0.04605022
EEF2KMT	1.030533834	0.000899278	0.008453138
SEC61G	1.028047347	0.002393037	0.018473102
NME1	1.02779908	0.00130001	0.011402146
WDR75	1.026956351	4.91E-06	0.000102953
POLR1B	1.025504389	4.84E-06	0.000101743
NOC3L	1.023704833	7.77E-08	2.67E-06
PRKAR1B	1.02347076	6.87E-09	3.00E-07
THY1	1.019534945	7.68E-06	0.000152946
CSTF2	1.019037978	0.000245049	0.002910245
KNOP1	1.018686812	1.03E-06	2.62E-05
DPY19L1	1.01852203	0.001190283	0.010634236
TCOF1	1.014762651	0.001028924	0.009431873
CRLS1	1.010741201	0.000960476	0.008913174
AKAP7	1.010019475	0.000606575	0.006128559
PM20D2	1.009488181	5.13E-05	0.000798351
MRPL39	1.007267425	2.07E-05	0.000366605
UST	1.005431277	3.42E-06	7.49E-05
GSTO2	1.005081983	0.000612992	0.006176775
REEP4	1.002003996	0.005305695	0.034268103
SNRPD1	1.001822221	0.008117485	0.047231859
NUP188	1.001067665	4.26E-08	1.56E-06
TCTN1	-1.000481793	0.002461281	0.018851423
RNF130	-1.001957272	0.005843957	0.036726713
TTC12	-1.00213627	4.33E-06	9.27E-05
ANKEF1	-1.002313559	0.002582405	0.019599324
IQSEC1	-1.004250701	0.000536267	0.005509571
SERPINB1	-1.005068153	0.001216095	0.010821953
ITPR3	-1.005167371	2.58E-05	0.00044211
PITPNC1	-1.005222926	0.005592083	0.035547415
ZNF439	-1.006055326	0.00192973	0.01557953
NLRX1	-1.006270996	1.07E-07	3.55E-06
TMEM91	-1.006530935	0.006821942	0.041325825
TPCN2	-1.00978577	5.66E-05	0.000867129
RP11-774O3.3	-1.010162803	0.002673531	0.020081327
CYP2F2P	-1.011602652	0.006835846	0.041398929
MXRA8	-1.011973941	0.000213704	0.002603837
LMOD1	-1.012293632	0.004620963	0.030653656
OR2A1-AS1	-1.013924342	0.002565968	0.019504319
SLC29A3	-1.014006094	0.001763865	0.014531901

GATS	-1.01413531	0.001896927	0.015393313
PINK1	-1.014394355	0.000912999	0.008560691
FBXO6	-1.014786953	0.00175297	0.014484493
RMDN2	-1.015123829	0.001088874	0.00989291
SSBP2	-1.016728908	0.000242983	0.002888761
C11orf95	-1.016877854	0.00023227	0.00277603
RP3-428L16.2	-1.021067482	0.00323286	0.023283932
STIM1	-1.022313744	0.001461094	0.012532157
EZH1	-1.022324056	1.25E-06	3.07E-05
KIAA1958	-1.024417845	0.00150042	0.012782429
NDRG3	-1.025421064	0.000202761	0.002498867
CYTH3	-1.027296595	0.000266421	0.00311807
C8orf58	-1.027646972	2.99E-06	6.67E-05
HDAC5	-1.028177849	2.16E-08	8.46E-07
PPP1R3E	-1.029639759	5.59E-05	0.000858023
DDR2	-1.031677844	0.005130665	0.03338154
PRKD1	-1.034526001	0.000166682	0.002128719
CEMIP	-1.036397001	0.005811293	0.036600269
MAP3K5	-1.037869607	5.88E-06	0.000120776
LZTS1	-1.038797847	0.003863644	0.026731246
APBB3	-1.040992485	0.002162938	0.017011443
RADIL	-1.042997893	0.004882791	0.032032305
HK2	-1.043849175	6.60E-05	0.000981632
KIAA1614	-1.044358974	1.17E-05	0.00022123
HCG11	-1.045083791	0.00149984	0.012782429
ATP6V1G2	-1.04537333	0.005943314	0.03724444
OFD1	-1.04539624	3.09E-07	9.17E-06
FAM89A	-1.047525748	0.000651583	0.006495943
GEMIN8	-1.048646382	0.000648568	0.006471609
SERINC5	-1.051512515	0.002055981	0.016387506
KIAA0513	-1.051953224	0.006995491	0.042116817
CD302	-1.052602562	0.000690463	0.006796383
MTURN	-1.05300382	0.00019419	0.00240905
ZNF660	-1.054679373	0.006292796	0.038861678
ZNF491	-1.055654414	0.002036409	0.016260251
RP11-290D2.6	-1.059227559	0.003903648	0.026925374
TECTA	-1.060477807	0.007609161	0.044888276
ZNF575	-1.061950673	0.004383396	0.02947677
PLCD1	-1.061987254	0.000790851	0.007598709
PHF11	-1.063421401	1.50E-09	7.57E-08
PABPC1P4	-1.065536078	0.003466767	0.02454284
STAG3	-1.067208102	0.002743316	0.020496215
CTGF	-1.067258681	0.003006851	0.021969995
HEXA	-1.068054832	0.008171204	0.04748314
C8orf44	-1.070340973	0.006625746	0.040386947
CMTM4	-1.071619505	4.92E-05	0.000775383

SNN	-1.071856676	8.96E-05	0.001277622
CD109	-1.071994294	0.00010275	0.001430573
RGAG4	-1.073457179	0.001657655	0.013823606
FAM3C2	-1.073685103	0.00625885	0.038715897
FNDC10	-1.075283886	0.004531046	0.030208312
GLB1L	-1.076878747	0.007305324	0.043505764
IKBKB	-1.077530624	0.000848166	0.008026244
DMPK	-1.079872419	0.000773864	0.007470517
CABLES1	-1.080472959	0.003010076	0.02197931
ALOX12-AS1	-1.080679018	0.005989173	0.037438145
TBC1D12	-1.083699197	9.84E-07	2.52E-05
CPNE8	-1.083841406	0.000139112	0.001845629
CTHRC1	-1.084771686	3.96E-06	8.57E-05
CEP162	-1.087340514	0.000411793	0.004434887
ZNF677	-1.088179105	0.000126702	0.001706091
TRPV4	-1.088344009	0.001261726	0.011131339
STS	-1.088537189	0.000705287	0.006924162
RP11-517P14.2	-1.08915867	0.003533359	0.024889202
MICALL2	-1.089980568	0.003633721	0.025445076
SCPEP1	-1.090174608	0.000205908	0.002522484
TIMP1	-1.092461579	2.29E-06	5.31E-05
KLF12	-1.094636243	0.000166307	0.002125125
SMPD1	-1.096377718	0.000400095	0.004325437
DNAH10OS	-1.100889099	0.003756778	0.026148482
TFDP2	-1.101016977	0.00013564	0.001808082
ZNF846	-1.102297874	0.001035627	0.009481746
LHPP	-1.102551821	0.000122468	0.00166198
CPQ	-1.103806708	0.002638135	0.019928353
ABTB1	-1.105286813	1.75E-05	0.00031577
NR6A1	-1.112199107	0.006800988	0.041221046
GOLGA8N	-1.112516613	0.003342531	0.023895967
COQ8A	-1.11414224	0.000170211	0.002167647
ISG20	-1.114752986	0.007756721	0.045598747
CPNE1	-1.115688486	5.96E-10	3.28E-08
TIMP2	-1.116174465	0.004807509	0.031635203
TFPI	-1.117600383	0.000267056	0.003122251
CALHM2	-1.118205431	1.93E-05	0.000343974
ZNF432	-1.119533135	0.008672783	0.049646281
PDK2	-1.121911651	5.72E-05	0.000874074
TRAK2	-1.123913074	1.74E-05	0.000315377
PTP4A3	-1.124127105	0.00645528	0.039576188
C1orf54	-1.125530474	0.000263291	0.003084638
FAM3C	-1.127021435	0.000871357	0.008225775
PDLIM2	-1.128161332	1.06E-06	2.68E-05
PRRT3	-1.130680436	0.000359506	0.003972364
MOK	-1.131561104	0.000807267	0.007720242

RP11-339B21.10	-1.132514093	0.005945901	0.037250292
C17orf100	-1.132523151	0.003725603	0.025991588
HIP1R	-1.137805925	0.000116302	0.001592693
RP11-333E1.1	-1.138061297	0.007091907	0.0425043
STAT2	-1.14243246	0.002985001	0.021836529
CARF	-1.144185253	0.000410886	0.004427242
BAALC-AS1	-1.145760563	0.00154332	0.013078183
SLCO3A1	-1.146222106	0.001436904	0.012359545
ZNF763	-1.147586696	0.000558382	0.005710748
ANXA9	-1.147851926	0.001639138	0.013704675
GMDS	-1.148484251	0.005459444	0.034950677
ZNF571	-1.148617555	0.001435472	0.012354703
CEP250	-1.150279791	7.28E-06	0.000145253
PTN	-1.151794223	0.005465496	0.034969556
ST6GALNAC6	-1.153157163	2.83E-12	2.21E-10
WWTR1	-1.153750432	0.00556318	0.035413626
HSD17B11	-1.153819788	0.000228841	0.002746696
EHBP1	-1.15453603	1.92E-05	0.000343799
ATP6AP1L	-1.154625589	0.008106482	0.047180021
ZNF585B	-1.154730158	2.26E-06	5.26E-05
ADGRE5	-1.155521519	4.79E-06	0.000101063
ZFP14	-1.156032231	0.003515606	0.024802901
ME3	-1.156283249	6.18E-05	0.000927917
SPACA9	-1.158805249	0.000136291	0.001812475
CAT	-1.160265356	4.28E-06	9.17E-05
PML	-1.160977924	0.000472971	0.004958716
MMP19	-1.163304383	0.002387609	0.018449994
FHOD1	-1.163956862	0.002475657	0.01894865
CHST11	-1.166970659	0.001538466	0.013046862
MTSS1L	-1.167115177	0.000242297	0.002882124
ACOX2	-1.167870202	6.26E-06	0.000127422
ASPHD2	-1.169188277	0.005291239	0.034213946
FGFR1	-1.170679675	0.000145749	0.001914528
S100A13	-1.170969406	1.88E-05	0.000337173
CSAD	-1.170976859	0.002947336	0.021668589
KALRN	-1.171283819	0.00121057	0.010781291
RGS3	-1.171956667	6.67E-15	8.04E-13
DNM3OS	-1.172269104	0.000619771	0.006228383
PDCD4	-1.174980115	3.92E-07	1.13E-05
TLR3	-1.176349652	0.002679133	0.020096618
IRX5	-1.1825767	2.24E-05	0.000392368
RILP	-1.183066515	0.001020132	0.009358894
AHR	-1.184163447	0.000229018	0.00274736
TCEANC	-1.185082183	0.000312499	0.003554009
RP11-33B1.1	-1.186990533	0.000608525	0.006145502
VMAC	-1.187069046	4.06E-06	8.76E-05

ZNF345	-1.18762743	0.001174592	0.010535801
AVIL	-1.188296189	0.001400986	0.012113399
ULK4	-1.188448738	7.74E-06	0.000154022
RP11-539I5.1	-1.188458203	0.001853877	0.015113287
NMRK1	-1.189204051	3.56E-05	0.000581723
ZNF503-AS2	-1.190438104	0.002595807	0.019667927
CREBRF	-1.19559053	0.002805963	0.02085368
HECA	-1.197143466	0.000315371	0.003574041
TTC3P1	-1.197146653	0.001293436	0.01135775
SRPX2	-1.199368391	0.002454973	0.018815892
CASC2	-1.199791568	2.92E-05	0.000491612
RFTN1	-1.200656972	7.77E-06	0.000154376
ZNF836	-1.200729689	0.000661088	0.006561682
AC005682.5	-1.203747734	0.001321873	0.011542644
GGN	-1.205671359	0.004254992	0.028793687
KIAA0895	-1.205991279	0.006363571	0.039180893
MXD4	-1.206132685	0.00017907	0.0022575
CTC-471F3.5	-1.20777555	0.004522269	0.03016739
TBCK	-1.208178454	0.000167426	0.002135794
ZNF493	-1.211203573	0.000967083	0.008967101
SPATA20	-1.211663845	1.88E-09	9.24E-08
SPACA6P-AS	-1.215488442	0.008747607	0.049906306
RP11-195F19.9	-1.21901278	0.000954476	0.0088721
VSIR	-1.219999054	0.000384659	0.004195522
ARHGAP31	-1.221595869	1.82E-05	0.000327537
PTPN3	-1.2258251	0.004595293	0.03052829
LTBP1	-1.226030499	1.72E-05	0.000312185
ITGA9-AS1	-1.2263447	0.00030561	0.003489746
CERCAM	-1.228761942	6.10E-05	0.000917999
LINC01547	-1.230169798	0.000104043	0.001444995
SGCD	-1.231502891	0.000503797	0.005223595
MMD	-1.234408813	0.004660435	0.030861007
TMEM8B	-1.234805373	0.00095924	0.008907261
ARHGAP22	-1.234857819	0.002797662	0.020812571
MTMR11	-1.240482289	4.45E-07	1.27E-05
LINC00959	-1.243420841	0.000101666	0.00141688
JAM2	-1.246124204	0.001636201	0.013695361
LINC01116	-1.246977564	0.000109994	0.001516432
ZNF577	-1.247562723	0.003358004	0.023958258
VAX2	-1.249219774	0.000818159	0.007811145
ANKH	-1.251032369	2.12E-05	0.000373848
DPP7	-1.252511651	0.00360812	0.02532087
KCNK1	-1.252850341	0.00121741	0.010828777
RABGAP1	-1.256115826	1.41E-06	3.42E-05
BAIAP2L1	-1.257008033	0.000762358	0.007381579
EEPD1	-1.257312523	8.56E-06	0.000168218

CSPG4P12	-1.258135705	0.00870739	0.04977765
ZDHHC1	-1.260243407	0.002150556	0.016940025
RAB23	-1.260651004	0.001946261	0.015690509
ABHD5	-1.261530407	0.001200194	0.010705804
GSN	-1.265516326	9.99E-07	2.56E-05
BOC	-1.270579348	0.005179811	0.033638179
ZEB2	-1.271209914	3.85E-07	1.12E-05
CUEDC1	-1.271651222	0.000836598	0.007940111
SAYSD1	-1.273044267	5.21E-08	1.86E-06
CPEB2	-1.273831123	0.000906785	0.008513057
CCDC125	-1.274757576	3.83E-05	0.00062105
SH3BP4	-1.275115623	4.64E-05	0.000737088
BAHCC1	-1.275236917	2.27E-07	6.99E-06
TAPBPL	-1.277850171	0.004431755	0.029722162
ANKRD33B	-1.280457558	0.007510102	0.044420155
WDFY3-AS2	-1.282509613	0.00020823	0.002544023
TTC39A	-1.285638922	0.006261287	0.038720347
LOXL1	-1.285802506	4.60E-06	9.77E-05
AC113189.5	-1.28631169	0.001785701	0.01465289
CTD-2530N21.4	-1.286717427	0.008099125	0.047149371
FTH1P8	-1.287342006	0.004635764	0.030733752
IL11RA	-1.289183118	5.98E-10	3.28E-08
ACACB	-1.289274339	5.72E-05	0.000874862
DENND6B	-1.289842064	0.000575898	0.005871243
NFIX	-1.290773051	4.21E-05	0.000676535
DOCK4	-1.2915237	2.39E-05	0.000415176
C1orf132	-1.296534188	0.002617318	0.019807035
PAM	-1.297437974	2.84E-05	0.000481068
CAPG	-1.298113115	0.002782259	0.020708573
LURAP1	-1.29867582	0.006732776	0.040884566
HOXA3	-1.300339033	9.26E-06	0.000180032
WWOX	-1.304596779	0.004349891	0.029277665
PRRX1	-1.307125578	0.002306349	0.017928282
GOLGA6L5P	-1.308861352	0.002827757	0.020974157
BTG1	-1.31331332	0.00120254	0.010717049
PCDHGA6	-1.314422572	0.000583359	0.005928541
SEMA6C	-1.316530438	4.19E-07	1.21E-05
AK1	-1.316889126	5.52E-05	0.000850598
FLVCR2	-1.31728398	0.008743928	0.049899875
RP11-134L10.1	-1.317534761	0.00800118	0.04674821
RBFADN	-1.318258355	0.000548207	0.005614328
ZFYVE28	-1.3200197	0.00083257	0.007905208
CAMK2D	-1.320574492	0.000172069	0.002185135
FAM110B	-1.323125609	0.001157769	0.010426397
SLC9A5	-1.326725554	9.66E-05	0.001357424
TNFAIP8L3	-1.328541761	0.000283756	0.003285129

MXRA5	-1.329823919	0.00321085	0.023182504
NIPAL2	-1.330302183	8.16E-08	2.79E-06
CHST3	-1.331449206	1.67E-05	0.000303177
RORA	-1.332025179	0.000933976	0.008713884
FTH1P23	-1.332047483	0.006880585	0.041570834
RILPL2	-1.33327623	5.05E-08	1.81E-06
CD83	-1.334049526	0.000867799	0.008201705
CTA-963H5.5	-1.337102845	0.002088533	0.016570794
ZNF503	-1.337669172	2.19E-07	6.76E-06
MIR600HG	-1.338269473	0.004157074	0.028266279
ITGB5	-1.343825325	9.11E-11	5.69E-09
ZNF436	-1.344378816	8.19E-07	2.15E-05
AC012360.6	-1.345516582	0.002677499	0.020096618
C14orf37	-1.346593378	0.000182753	0.00229025
GRAMD4	-1.350241546	0.003686019	0.025755285
SLC9A9	-1.352865203	0.000307793	0.003511118
APOL6	-1.353310264	0.001425484	0.012278119
PLXND1	-1.353534401	2.45E-09	1.18E-07
CRNDE	-1.354106069	3.42E-09	1.59E-07
RP11-81A22.4	-1.354786117	0.000696799	0.006849784
LINC00910	-1.358683665	0.00109781	0.009954041
RASA4B	-1.359220223	0.001270785	0.011198113
ANKRD35	-1.360667617	0.001613681	0.013542067
TRPS1	-1.363441958	0.000114691	0.001573987
ATP9A	-1.363618334	3.20E-06	7.10E-05
SMIM3	-1.363880353	0.000666298	0.006610482
CBX7	-1.364076862	5.59E-05	0.000858023
FAAH	-1.366147443	0.005903995	0.037047417
ANGPT1	-1.366692939	0.000311945	0.003553086
CARD6	-1.367219452	0.001558094	0.013168731
RP11-54A4.2	-1.371508783	0.007667109	0.045159213
PRRT2	-1.372019935	5.92E-05	0.000898052
RUNX2	-1.372483929	0.001359163	0.011815232
CTC-203F4.2	-1.374149822	0.005949746	0.037264025
SLC16A4	-1.375184778	0.000330478	0.003717214
PPP1R12B	-1.375555603	0.000377938	0.004129473
FUCA1	-1.377065558	2.37E-05	0.000413019
IL1R1	-1.377111113	0.00745649	0.044201737
CCDC113	-1.379442766	6.42E-08	2.24E-06
EML1	-1.383376874	5.17E-05	0.000803692
ARVCF	-1.38622713	0.004267399	0.028843008
POLR2J3	-1.38738504	0.000128563	0.001728044
PLCL1	-1.387397343	0.004523445	0.03016739
PRX	-1.392773403	0.004555709	0.030336851
ADGRG1	-1.39308369	0.007349905	0.043661307
RP3-508I15.9	-1.393232327	3.06E-05	0.000509729

LMO7-AS1	-1.394343017	0.001492739	0.01274056
MYLK4	-1.397811991	0.000763602	0.007390444
LL21NC02-1C16.2	-1.400163552	0.006873189	0.041546944
RP1-253P7.4	-1.402832907	0.003119583	0.022654161
USP32P3	-1.403101501	0.006102882	0.038001396
CD27-AS1	-1.404404851	8.00E-06	0.000158466
PCGF5	-1.404644305	1.87E-05	0.000334884
TMEM140	-1.405717389	0.000448232	0.004746241
MIR4458HG	-1.406173037	0.001179994	0.010567441
SGMS1-AS1	-1.409612989	0.000214186	0.002608302
HIP1	-1.413100241	4.65E-05	0.000738688
PLEKHG4	-1.415436962	0.003188204	0.023033749
VGLL3	-1.41588731	1.24E-07	4.08E-06
CCDC146	-1.421632732	0.00198591	0.015949937
LAMB3	-1.424056608	0.000301471	0.003449469
RP11-120M18.2	-1.424876456	0.004928205	0.032297485
ADGRL1	-1.42493602	0.003653939	0.025554898
VDR	-1.427885674	0.00415826	0.028266279
JHDM1D-AS1	-1.428255725	0.003388374	0.024093796
FAM149A	-1.4285268	0.002090775	0.016576908
ZNF793	-1.429435239	0.001851018	0.015100905
TMEM56	-1.429865554	0.007757916	0.045598747
CEP126	-1.43149923	1.66E-05	0.000302528
CTSA	-1.432126906	0.002658275	0.020046901
RXRA	-1.433414734	9.37E-10	4.92E-08
FN1	-1.433638131	0.005022367	0.032785743
LRP3	-1.43366993	2.45E-12	1.92E-10
FSCN2	-1.433910916	0.007053232	0.042358645
SNX18	-1.436200329	0.000296566	0.003403719
LINC00339	-1.439671501	1.21E-05	0.000227749
STXBP5	-1.440312799	1.34E-09	6.80E-08
PLEKHA4	-1.440554689	0.00018144	0.002278458
TSC22D3	-1.441241188	0.003312951	0.023759856
ZNF664	-1.441453592	4.00E-06	8.64E-05
PDCD4-AS1	-1.441615648	0.002301601	0.017919085
CALCOCO1	-1.441960249	5.54E-05	0.00085247
CTD-2623N2.3	-1.442074811	0.00236069	0.018286022
MINDY1	-1.442282379	7.23E-05	0.001062826
PPFIA3	-1.442498843	0.003732949	0.02602195
KLF3-AS1	-1.443257284	0.000593623	0.006019278
ATP5F1P5	-1.443746458	0.004120317	0.028118701
STK17B	-1.445296738	2.08E-07	6.49E-06
RP11-228B15.4	-1.447618288	0.002707672	0.020270224
ZCWPW1	-1.451221364	0.000731612	0.007126736
ENG	-1.451663748	0.001444743	0.012406079
ZCCHC24	-1.452235616	5.55E-05	0.000853453

C5orf56	-1.455806988	0.001073239	0.009778446
ECM1	-1.456874988	5.94E-05	0.000899676
MACROD2	-1.45825105	0.005504041	0.035136387
AK9	-1.459696092	0.00097295	0.009014085
FAM65B	-1.459862406	0.007567782	0.044679247
AKAP12	-1.460236217	1.61E-05	0.000293914
GPC3	-1.461442333	0.002389226	0.018449994
ABHD2	-1.463503317	1.36E-07	4.43E-06
NPIPA1	-1.46389776	0.003022208	0.022039337
MXI1	-1.463903869	9.84E-05	0.001378547
CAPS	-1.466483184	7.36E-05	0.001079019
MYLK	-1.46696254	0.00032676	0.003683126
SRGN	-1.472526389	0.005472201	0.035002522
PMP22	-1.472706289	1.66E-06	3.98E-05
RP11-861E21.2	-1.475228621	0.003512266	0.024797154
ARRDC2	-1.476648118	1.09E-10	6.73E-09
ZNF528	-1.477416533	7.24E-06	0.000144756
LINC01376	-1.477993267	0.006861837	0.04148945
CCPG1	-1.478979939	0.00157659	0.013290167
DHX58	-1.480214319	0.002340465	0.018166809
ETV1	-1.4807159	0.00042242	0.004532013
CRACR2A	-1.484951805	9.89E-06	0.000191359
RBPJ	-1.485732237	0.000253393	0.002990441
RP11-622C24.2	-1.486119171	0.002098294	0.016618988
SIDT2	-1.486785538	2.55E-09	1.22E-07
SPINT2	-1.487163974	0.000956969	0.008891602
ESR1	-1.48937205	0.00114292	0.01030915
RP11-541N10.3	-1.490420526	0.000138513	0.001838762
MAGEE1	-1.490557162	9.77E-05	0.001369515
GAS6	-1.491457436	7.67E-07	2.04E-05
POLM	-1.492020924	0.008526737	0.049060502
MIR210HG	-1.492283563	0.001898279	0.015396095
ZCCHC14	-1.492932159	0.002403583	0.018535462
PTPRM	-1.49305852	0.001677981	0.013962113
WDR31	-1.493547648	1.18E-08	4.90E-07
ATP8B4	-1.495087839	0.0013987	0.012098271
FAM171B	-1.497334774	8.87E-05	0.001268036
ACCS	-1.497380994	0.002008001	0.016086444
RAVER2	-1.497678546	6.06E-05	0.000913171
ZFP36	-1.497938873	4.61E-05	0.000734718
LGR4	-1.497981386	3.14E-08	1.19E-06
LINC00663	-1.497986057	0.003713145	0.025912696
CYP4V2	-1.501507187	2.58E-07	7.84E-06
GMDS-AS1	-1.505182326	7.74E-05	0.001127049
WASF3	-1.505515822	0.000122933	0.001667282
DECR2	-1.505582461	9.48E-11	5.90E-09

STAT1	-1.506783013	0.000755343	0.007319946
NUP50-AS1	-1.509704486	9.54E-07	2.46E-05
GOLGA8A	-1.510419419	0.006952269	0.041912571
FMO4	-1.511019216	0.008515059	0.049026295
MRVI1	-1.512629459	0.004019392	0.027563467
CTD-2541J13.1	-1.512837558	0.001081445	0.00983334
AOX1	-1.521901134	0.001714515	0.014224101
AC004623.3	-1.524916103	0.000409204	0.004415446
MITF	-1.526503697	0.001974384	0.015883149
TMEM198B	-1.530443782	2.60E-05	0.000445571
RP11-394B2.1	-1.533624951	0.001668743	0.013900641
ZC3H12B	-1.535090745	3.03E-05	0.000506328
FOXD2-AS1	-1.535252966	0.004285295	0.028930044
RCBTB2	-1.536420651	9.50E-06	0.000184383
RP11-632K20.7	-1.538407	0.005674271	0.035948214
PRR16	-1.538476405	2.00E-05	0.00035458
CCDC92	-1.540171058	6.93E-07	1.87E-05
RP11-102G14.1	-1.541705474	0.008194613	0.047582372
TSPAN10	-1.541812243	0.000167656	0.002136312
MIR99AHG	-1.541886573	9.37E-05	0.001323709
SLC27A1	-1.54193268	1.09E-06	2.74E-05
ZNF671	-1.543189228	5.66E-08	2.00E-06
RP4-800G7.2	-1.544053522	0.003414975	0.024252347
CSPG4P11	-1.547333645	0.005648903	0.035837863
NUTM2G	-1.548154197	5.24E-05	0.000812731
EDNRA	-1.555389117	2.61E-10	1.50E-08
INSR	-1.557865324	0.001006145	0.009271904
NFATC1	-1.559229995	1.49E-08	6.09E-07
RARA	-1.55990205	0.000352839	0.003908276
CTD-2528L19.6	-1.560011386	0.004051731	0.027734612
ECHDC2	-1.561745016	0.001202881	0.010717049
ITGB8	-1.563228601	8.67E-07	2.26E-05
SPDYE6	-1.563520129	0.005845697	0.036726713
PSMG3-AS1	-1.563533016	9.26E-06	0.000180032
CPED1	-1.564632641	0.001765139	0.014537085
NLRC3	-1.565828691	0.000713317	0.006987782
CA12	-1.568388544	0.000288856	0.003333902
GLRB	-1.569282827	0.000885767	0.008336564
ARHGEF10L	-1.572707294	8.34E-10	4.44E-08
ADAM32	-1.573323211	0.005715309	0.036147246
LINC00565	-1.574120462	0.000884885	0.008331739
SNAI1	-1.577384088	0.006037683	0.037657833
COL6A1	-1.577963305	3.43E-07	1.01E-05
FYN	-1.578282353	1.53E-06	3.70E-05
RP11-395L14.18	-1.581320199	0.000933855	0.008713884
STXBP5-AS1	-1.583025804	0.000271672	0.003168343

ARHGEF4	-1.583357184	0.000383629	0.00418759
B4GALT1-AS1	-1.585636415	0.007636325	0.045013182
TEX22	-1.58947513	0.006024665	0.037597448
PCDHGB2	-1.593969016	0.002672738	0.020081327
EFCAB6	-1.594640544	0.000447212	0.004739353
SLC25A34	-1.598719539	0.001620994	0.013583207
CBLB	-1.599424237	5.01E-05	0.000783904
LGALS3	-1.601006371	5.60E-10	3.10E-08
LINC00899	-1.601553231	0.00692248	0.041766496
KBTBD11	-1.603171371	0.000441408	0.004688227
NDRG1	-1.604783729	6.04E-06	0.000123621
ROBO4	-1.608042952	0.002440292	0.018739427
FLT3LG	-1.60889399	0.000758134	0.007343837
SRPX	-1.614416336	0.002427016	0.018677831
TMEM35A	-1.615548434	0.000629083	0.006313517
CTC-490E21.10	-1.617160707	9.17E-06	0.000178651
ZFP36L2	-1.619605308	9.13E-07	2.36E-05
GAA	-1.624673061	0.007892346	0.046280271
CTC-490E21.11	-1.625685553	0.001222953	0.010857263
OSER1-AS1	-1.630284067	0.004131507	0.028169482
LINC01341	-1.63070374	0.000229751	0.002754522
TSC22D1	-1.631538451	0.000177283	0.002238734
SNX9	-1.63192186	1.63E-10	9.70E-09
RFPL1S	-1.632246498	0.001840637	0.015032535
LDHD	-1.63363033	0.001421368	0.012247343
RP11-522I20.3	-1.635940569	0.000191388	0.002383462
LRIG3	-1.636415759	4.93E-06	0.000103237
GAS1	-1.636568458	0.000479837	0.005013059
PSMB9	-1.637975784	0.000267941	0.003130981
Z97634.3	-1.643173678	0.00730191	0.043505764
ADAMTS13	-1.645290534	0.001288449	0.011318369
AKAP3	-1.649911099	0.001161894	0.01044685
ACSF2	-1.653291032	0.002150909	0.016940025
VAMP5	-1.654014141	1.72E-06	4.10E-05
ANPEP	-1.654256353	0.000263697	0.003087789
CFAP206	-1.654915701	0.006616215	0.040359838
SOCS3	-1.654958697	2.55E-07	7.77E-06
LINC00174	-1.655033719	8.50E-05	0.001221314
PDGFRA	-1.655275036	1.69E-05	0.000306652
RP11-284E5.1	-1.65556232	0.00144058	0.012379764
MAP7	-1.657215583	0.000458275	0.004822585
CFAP69	-1.658858021	0.000317074	0.003587938
LFNG	-1.65895715	2.54E-05	0.000436869
PARP10	-1.659311081	5.15E-06	0.000107173
ARNT2	-1.661248017	1.23E-06	3.03E-05
CTD-2331H12.5	-1.661531005	0.006351348	0.03912701

LAYN	-1.663065648	0.00112459	0.010156002
SYCP2	-1.664980275	0.004947155	0.032379157
DNM1P46	-1.671071244	0.000587969	0.005972695
CTSB	-1.676867865	0.000455451	0.004804081
KANK2	-1.679086079	1.78E-12	1.45E-10
RALGPS1	-1.681692531	0.000148362	0.00194508
JMY	-1.682323643	0.003237521	0.023307877
RP11-214K3.19	-1.682618588	0.006431509	0.039459074
HAS3	-1.682948084	0.001832497	0.014971484
EMP1	-1.684660274	4.46E-05	0.000712633
LA16c-360H6.3	-1.687056487	0.005406862	0.034692874
MIR497HG	-1.688479224	0.001164621	0.01046719
AMACR	-1.689258341	1.05E-05	0.000200758
FAM20C	-1.691296405	0.000448283	0.004746241
PTCH2	-1.691375118	0.006292518	0.038861678
AFF2	-1.692091758	4.87E-05	0.000769276
HPCAL1	-1.693348697	5.46E-13	4.92E-11
EVA1C	-1.695613399	3.76E-06	8.19E-05
PPIL6	-1.696487631	0.00863127	0.049467952
PSD3	-1.69779638	0.000286567	0.003312565
HLA-B	-1.697979356	0.002034303	0.016249192
CYP11A1	-1.700727167	0.001117608	0.010101047
CLU	-1.705621879	0.00337888	0.024054594
C1GALT1	-1.708737094	1.06E-08	4.48E-07
A4GALT	-1.712497697	2.10E-11	1.43E-09
LYRM9	-1.713208971	9.30E-05	0.001319859
RP11-752L20.3	-1.713358791	0.001988427	0.015961945
ARHGEF6	-1.715681832	4.20E-06	9.03E-05
FBXL22	-1.725377119	0.003922072	0.027019387
ADORA1	-1.731751593	3.36E-07	9.90E-06
MYO5B	-1.733598329	7.79E-05	0.001132649
SOBP	-1.736852509	7.58E-08	2.61E-06
MISP3	-1.737196682	0.00271319	0.020304785
PCDHGA5	-1.738057287	5.08E-06	0.000105791
HOXA2	-1.739762026	0.00058174	0.005917421
LINC00968	-1.744103622	0.006216341	0.038554424
FAM21FP	-1.747963065	0.000595742	0.006035339
CAPN3	-1.748945303	0.00171064	0.01419718
IL7	-1.749746331	0.001155684	0.010411783
PAXIP1-AS1	-1.751253851	1.46E-06	3.53E-05
CSF1R	-1.754880151	2.62E-05	0.000448368
SYNE3	-1.76012286	2.79E-14	3.06E-12
PLBD1	-1.761378766	1.28E-07	4.20E-06
ATP10A	-1.762579613	5.27E-05	0.000816042
CTD-2516F10.2	-1.763294625	0.002023291	0.016172696
IL17D	-1.770511163	2.31E-05	0.000404156

RP1-122P22.2	-1.771498988	0.000141179	0.001866464
GJA1	-1.772029874	5.25E-07	1.46E-05
CLEC2D	-1.773318157	8.22E-05	0.001192094
CYP2E1	-1.776579762	0.00014612	0.001918288
ZNF528-AS1	-1.780090869	3.02E-07	9.05E-06
LARGE1	-1.783194066	8.34E-07	2.19E-05
BCL2L11	-1.784420224	0.007831102	0.045980953
CLDN23	-1.789378428	0.001473996	0.012618817
AP000347.4	-1.790672763	0.007043482	0.042337901
PRSS23	-1.791051699	3.74E-10	2.12E-08
ABCA7	-1.792779376	0.002830447	0.020987207
RP4-665J23.1	-1.79538299	0.001440234	0.012379764
CAPS2	-1.796305301	7.87E-05	0.001144179
BICC1	-1.797149415	2.17E-09	1.05E-07
CLCA2	-1.798092434	0.005586589	0.035522513
IL3RA	-1.799305998	0.003222442	0.023236426
RNF125	-1.802527031	0.006343983	0.03911371
ADAMTS2	-1.803817853	3.74E-09	1.73E-07
ACE	-1.812968625	0.000728217	0.007105949
TMEM86A	-1.821706137	0.003528923	0.024865723
HOXB3	-1.822033531	3.95E-05	0.000637785
LINC00607	-1.823854735	0.000424485	0.004548244
RP5-1198O20.4	-1.824986979	0.000816574	0.007799324
LDB2	-1.82502925	0.002326916	0.018074095
PTPRG	-1.826606597	2.78E-08	1.07E-06
IKZF2	-1.82673035	1.64E-10	9.70E-09
SH3GL1P2	-1.827614617	0.001420817	0.012247276
RAB3IL1	-1.828853153	0.002782001	0.020708573
SPATA13	-1.831873075	0.000808424	0.00772803
UNC79	-1.834455247	0.006926403	0.041778979
MFAP5	-1.835638429	0.008744266	0.049899875
RP11-848P1.2	-1.835665976	0.008156621	0.047410625
PLA2G6	-1.837646478	0.000111506	0.001535408
C14orf159	-1.838051707	1.45E-08	5.97E-07
RP11-848P1.9	-1.840175011	3.72E-07	1.08E-05
HOXD3	-1.840799442	9.62E-06	0.000186411
RP11-434E6.4	-1.842542058	4.09E-05	0.000658861
LINC01535	-1.842580072	0.001979274	0.015905459
AC074141.3	-1.842737648	0.00148465	0.012690747
TRANK1	-1.846858941	1.04E-05	0.000200603
CMYA5	-1.851695043	4.22E-05	0.000677632
RPS20P22	-1.854585135	0.007079735	0.042465224
BEAN1	-1.857992156	0.004238946	0.028710965
CYP7B1	-1.858491065	0.001622563	0.013591298
CRISPLD1	-1.858691781	4.76E-06	0.000100371
DAPK1	-1.858943385	5.49E-07	1.52E-05

DSP	-1.860023929	0.000259713	0.003055428
ATP6V1B1	-1.861710115	0.002945946	0.021668589
LBH	-1.8631252	7.71E-05	0.001122482
CFAP43	-1.864555008	0.003583388	0.025170815
TP53I11	-1.868835177	7.80E-07	2.07E-05
F8	-1.870392186	2.12E-07	6.58E-06
WBP2NL	-1.872631071	0.001300758	0.011404274
LINC02102	-1.876254515	0.005521466	0.035217704
RP11-517I3.2	-1.877188999	2.12E-08	8.36E-07
ZNF365	-1.877582101	0.002498092	0.019091971
YPEL3	-1.877715938	3.51E-10	1.99E-08
TLR4	-1.878572365	5.85E-11	3.76E-09
RIBC1	-1.878824543	0.006637865	0.040436239
MSR1	-1.878930977	0.000132151	0.001768891
EPHX1	-1.87956601	4.34E-07	1.24E-05
IQCH-AS1	-1.880937617	0.000397385	0.004302337
ANKRD6	-1.881150653	0.000107542	0.00148618
WNT9A	-1.881664918	0.000782689	0.007533128
DNAJC6	-1.885000432	8.48E-05	0.001220134
PALM2	-1.885857204	0.002526356	0.019264748
FNDC4	-1.886725372	9.32E-07	2.41E-05
NPL	-1.89024601	0.001650732	0.013779081
GALNT1	-1.89234107	2.44E-06	5.61E-05
GPM6B	-1.893692482	3.14E-05	0.00052143
FSIP1	-1.896635326	2.35E-05	0.000409176
FPR1	-1.89894496	0.000684388	0.00675133
LY96	-1.902493548	0.000165452	0.002116598
FAT2	-1.902741627	5.71E-05	0.000873334
TESK2	-1.906610197	0.000149708	0.001957795
RP11-631N16.4	-1.906761475	0.000500849	0.005195418
CAMK1D	-1.90690129	7.03E-07	1.90E-05
AC009501.4	-1.906963103	0.000590224	0.005992901
HECW2	-1.908638671	0.000829879	0.007896283
CORO6	-1.91044978	1.16E-06	2.87E-05
RP11-456K23.1	-1.911068228	0.003547219	0.024979029
DCN	-1.914677276	7.11E-09	3.10E-07
MEIS3P2	-1.914772152	3.99E-05	0.00064462
SATB1	-1.914982267	2.15E-07	6.65E-06
GSN-AS1	-1.916225005	6.65E-05	0.000986518
TNS2	-1.922470922	1.51E-07	4.85E-06
NR4A2	-1.923114594	3.28E-06	7.25E-05
KCNK2	-1.925506636	4.72E-07	1.33E-05
FAM83H	-1.925691582	4.19E-06	9.00E-05
TMEM169	-1.927671897	0.001009037	0.009283549
ERRFI1	-1.92963346	9.67E-05	0.00135785
ADAM22	-1.930628087	0.000144603	0.00190391

ITGA11	-1.930957338	0.000841686	0.00798168
RP11-34F20.7	-1.934813119	0.006439982	0.039500307
LHFP	-1.935929996	2.41E-16	3.39E-14
LRRC6	-1.937331995	2.40E-05	0.00041628
ROS1	-1.940439759	0.003153916	0.022822606
PIWIL4	-1.941195198	9.00E-05	0.001280727
COL8A2	-1.941222894	0.002508923	0.019144765
LINC01119	-1.942941116	0.000225525	0.002712677
CTB-174D11.1	-1.944077155	0.004061152	0.027773794
TEKT4P2	-1.948291804	0.004505193	0.03010719
RP11-17A4.2	-1.951995841	0.005572167	0.035450811
DOK1	-1.953256883	8.04E-08	2.75E-06
ZNF429	-1.959399	1.33E-11	9.33E-10
TSPOAP1	-1.961882741	0.001787206	0.014659898
RP5-1180D12.1	-1.96315192	1.06E-06	2.68E-05
RP11-1055B8.4	-1.963523789	0.003624314	0.025402879
MYO15B	-1.965801666	0.001235623	0.010948181
MEGF9	-1.966499276	2.80E-07	8.45E-06
ENTPD3	-1.96966185	0.003734573	0.02602195
NFATC4	-1.971433326	1.13E-11	8.02E-10
HLA-A	-1.974094833	0.001184729	0.010601415
CD68	-1.97428748	3.22E-08	1.21E-06
CSDC2	-1.977639896	3.47E-05	0.000569124
PLEKHF1	-1.979494999	5.23E-05	0.000812346
PABPC5	-1.981020016	2.22E-05	0.000389844
CRLF1	-1.981865269	0.002233404	0.017480267
RHBDL2	-1.987173045	0.007303574	0.043505764
LNX1	-1.988978615	1.70E-07	5.41E-06
HOXB-AS1	-1.990806227	1.19E-05	0.000223242
DLG2	-1.991817046	0.000741799	0.007210385
RP5-827C21.4	-1.994705804	0.008726783	0.049863221
KCND1	-1.994792856	0.000193507	0.002403215
EMP2	-1.996211951	0.000307418	0.003508608
SNX10	-1.996583571	0.007210641	0.043021453
DGKG	-1.996846919	0.003284329	0.023614689
SRRM3	-2.001532618	0.000335327	0.003760502
ATP1A3	-2.00370368	0.002880756	0.021248402
PCDHB9	-2.004574837	0.000569335	0.005814856
LINC01301	-2.005110611	0.0003389	0.003789259
GHR	-2.005473062	1.44E-07	4.65E-06
RP3-512B11.3	-2.006920442	8.08E-07	2.14E-05
AGAP9	-2.007153666	0.001078356	0.009816382
MEGF6	-2.009430111	0.00707027	0.042431034
SAXO2	-2.010172281	0.000117649	0.001606014
ROR1-AS1	-2.012347615	0.002193251	0.017213814
NTM	-2.01843261	0.000149025	0.001950737

SCARA3	-2.019223529	5.68E-08	2.01E-06
COL5A3	-2.022247871	1.00E-09	5.23E-08
RAB6B	-2.025411761	2.34E-05	0.000407806
AC027612.6	-2.029774325	0.000352154	0.003904499
MAP6	-2.031836528	4.48E-07	1.27E-05
PORCN	-2.033424604	0.000353186	0.003910204
FLG-AS1	-2.034112584	0.000855841	0.008092082
SH3BGR	-2.038082306	0.000313963	0.003561676
NECAB2	-2.038609429	7.34E-05	0.001076121
SMAD6	-2.04019544	5.10E-05	0.000795854
MMP2	-2.042156034	1.51E-05	0.000277819
SSC5D	-2.042972572	0.00014133	0.001867364
RFX3-AS1	-2.045266035	0.003183829	0.023009516
LINC01703	-2.046470245	0.00153496	0.01302203
STAT4	-2.05145856	0.00710169	0.042540313
IFI30	-2.053021948	0.00359238	0.025218261
PODNL1	-2.054542014	5.00E-06	0.000104437
AP000654.4	-2.055543407	0.001015653	0.009325393
FZD5	-2.058573604	1.16E-06	2.87E-05
CTD-2026K11.5	-2.062624955	0.004782277	0.031510464
BVES-AS1	-2.063188774	0.006715657	0.040802593
PCDH18	-2.064506532	0.000334363	0.003751551
CFAP53	-2.067754353	0.003282363	0.023608087
ASAP3	-2.071056097	5.00E-08	1.80E-06
NTNG2	-2.072485915	0.000214476	0.002610417
FANK1	-2.077097658	0.000114629	0.001573987
CHRNE	-2.079224882	0.003577472	0.025144932
RP11-631N16.2	-2.079986126	0.000624603	0.006271349
GALNT5	-2.086132082	0.001939534	0.015641873
NIM1K	-2.087903291	9.33E-05	0.001322191
GNA14	-2.090336083	0.001219743	0.010840086
SLIT3	-2.092086692	0.001302874	0.011413948
HCP5	-2.095429287	0.001675211	0.013949363
PDGFRB	-2.100672447	6.11E-05	0.000918859
KATNAL2	-2.10530982	1.25E-14	1.44E-12
RP11-212I21.3	-2.107312061	1.01E-05	0.000195284
ZSCAN18	-2.107875655	2.69E-06	6.08E-05
LINC01118	-2.108722418	0.005412228	0.034717427
N4BP2L1	-2.110156322	0.00093954	0.008758549
PAK3	-2.112808836	0.005585646	0.035522513
RP11-671M22.4	-2.113229248	0.001975852	0.015889288
GCKR	-2.116121064	0.0083758	0.048434814
RP11-672A2.4	-2.117827985	0.00261856	0.019807035
FLJ37035	-2.121043052	0.000829468	0.007895711
RP11-357H14.16	-2.125273686	0.004795541	0.031587667
ZNF667-AS1	-2.125583696	4.57E-07	1.30E-05

LINC00933	-2.126514179	0.00042971	0.004599287
SLC6A16	-2.126585866	0.001352087	0.011763844
CACNA1A	-2.127894924	0.00015462	0.002008808
CTSF	-2.129433376	0.00055113	0.005641699
AC012462.2	-2.130678289	0.002020441	0.016170202
JAG1	-2.131729988	0.000344336	0.003840514
PTGIS	-2.131905277	0.000728863	0.007106096
AC091878.1	-2.133804317	0.002443704	0.018748649
POSTN	-2.135496787	0.000656682	0.006535222
LPAR6	-2.136087207	3.98E-10	2.24E-08
CEBPA-AS1	-2.13624426	0.003233162	0.023283932
VWA1	-2.136974215	0.00134929	0.011752067
ANGPTL4	-2.137499851	0.006294485	0.038861678
HOMER2	-2.140303975	1.16E-06	2.88E-05
ZNF521	-2.140319269	1.06E-05	0.000202903
TPTE2P6	-2.140665612	0.004746084	0.031317766
CRISPLD2	-2.145735009	1.46E-10	8.81E-09
SERPINI1	-2.148321776	4.09E-05	0.000659448
DNALI1	-2.149077837	9.68E-09	4.12E-07
RASSF2	-2.152935501	2.40E-05	0.00041628
KIAA1683	-2.154294045	2.06E-05	0.00036487
AIM1	-2.156092901	1.10E-08	4.60E-07
RNF144A	-2.15796594	0.005369979	0.034515169
PCDHGB3	-2.158103288	6.02E-05	0.000910284
PLCE1	-2.158886142	4.02E-07	1.16E-05
BACE2	-2.166025949	1.68E-05	0.000304379
SDHAP2	-2.166526366	1.26E-06	3.08E-05
RP11-46H11.3	-2.168565571	0.004872964	0.031977141
LINC00202-1	-2.169624119	0.001213548	0.010803548
NYNRIN	-2.17117161	0.00011354	0.001561511
ZFP2	-2.171455006	0.000206682	0.002527846
PTPRN2	-2.17485521	0.001064777	0.009717059
SPTBN4	-2.175657733	1.54E-09	7.72E-08
GSC	-2.180267277	0.000821845	0.007836384
SCD5	-2.189797949	0.000673594	0.006659419
FAAHP1	-2.190899536	0.00715942	0.042795141
NDST1-AS1	-2.191264136	0.004808225	0.031635203
AC092614.2	-2.1957	0.005797563	0.036544425
PTPN22	-2.195899259	1.18E-05	0.000222152
ZNF154	-2.196054274	9.37E-07	2.41E-05
SLC2A12	-2.200654696	0.001910806	0.015466185
CTA-217C2.2	-2.201385384	0.000386718	0.004215193
SETBP1	-2.203252783	2.53E-08	9.77E-07
PLAC4	-2.204460137	0.005151708	0.033494278
CD37	-2.20601303	0.000408753	0.004412687
PREX1	-2.206219086	8.31E-07	2.18E-05

SYP	-2.208533658	2.88E-05	0.000486664
RP11-134G8.5	-2.211602014	0.000507198	0.005256434
IL6R	-2.213268731	1.84E-06	4.36E-05
RP11-540A21.2	-2.215838607	1.24E-06	3.05E-05
NCF2	-2.217443893	0.001752685	0.014484493
RP11-351M8.1	-2.222021978	4.82E-07	1.35E-05
RAMP1	-2.223205998	2.42E-05	0.00041889
GPNMB	-2.223848364	0.00051198	0.005291401
SORBS2	-2.227074143	9.01E-05	0.001280727
CTD-2033D15.2	-2.227935567	0.003928432	0.027046665
PLEKHH2	-2.230760625	2.43E-07	7.45E-06
FZD1	-2.231985778	5.72E-24	1.90E-21
B3GALT2	-2.232692087	0.00092077	0.00861564
CCDC188	-2.233126298	0.000127017	0.001709312
FRMD4B	-2.233872872	0.003977205	0.027315715
PSCA	-2.237074774	5.83E-06	0.000119907
AC116366.6	-2.239870481	7.49E-05	0.001093756
PLPPR4	-2.240730125	0.000522799	0.00538768
FGL2	-2.244837292	0.006191065	0.038440723
ZNF667	-2.246229107	7.33E-05	0.001075018
SUSD5	-2.246316213	0.000426794	0.004570244
C16orf45	-2.2490208	4.63E-24	1.58E-21
CCDC152	-2.257109679	0.001845782	0.015069098
VSTM2L	-2.257496382	1.03E-06	2.62E-05
C1R	-2.25837286	0.004836409	0.031790816
RP3-399L15.3	-2.260434246	0.001015387	0.009325393
CXXC5	-2.260866604	1.58E-17	2.63E-15
CDON	-2.261438278	5.11E-06	0.000106355
AC068580.6	-2.262106448	0.000522969	0.00538768
FTCDNL1	-2.262132406	4.67E-06	9.90E-05
SUGCT	-2.263466173	6.50E-05	0.000968272
TNFRSF19	-2.266155238	0.000263255	0.003084638
P3H2	-2.267819408	0.000326544	0.003683126
CELF6	-2.270422534	0.003046292	0.022164774
SLC22A23	-2.274315238	0.001732253	0.014339595
ROCK1P1	-2.277174357	0.006346112	0.039116139
RASEF	-2.284001578	0.0012565	0.011098265
RP11-295M18.6	-2.28491661	0.007771877	0.045668896
STEAP1	-2.285331145	1.16E-05	0.000219729
SESN3	-2.285422705	0.000507496	0.005257108
RP11-43N5.1	-2.289506795	0.005827055	0.036667026
LSAMP	-2.289606284	5.52E-11	3.55E-09
ACSS3	-2.29338365	1.55E-08	6.29E-07
LINC01232	-2.294996781	4.47E-07	1.27E-05
RP11-726G1.1	-2.294997928	0.00175923	0.014504315
CTC-301O7.4	-2.296390706	0.002336381	0.018141359

HCG4P5	-2.296517668	0.000216343	0.002627474
CYP2G1P	-2.297113438	0.000228414	0.002743043
HCG4P7	-2.297899332	0.002555588	0.019435056
CCDC68	-2.298557354	1.94E-07	6.11E-06
TBX18	-2.298732721	2.41E-07	7.39E-06
FBXO16	-2.299895336	0.000173011	0.002192151
ZNF436-AS1	-2.300223274	1.97E-06	4.63E-05
CTD-2054N24.2	-2.305542433	4.82E-06	0.000101407
SERPINE2	-2.305873145	0.000529813	0.005448232
MARCKSL1P1	-2.30721866	0.008401401	0.048557946
RPL23AP49	-2.307489762	0.004138183	0.028189414
ADD3	-2.309115545	7.35E-08	2.54E-06
SPTLC3	-2.313574685	6.64E-16	8.75E-14
LTBP2	-2.314370237	7.43E-07	1.99E-05
RP11-111F16.2	-2.315182406	0.000196247	0.002429213
SRGAP3	-2.317563455	2.17E-06	5.07E-05
CTSD	-2.319294949	1.10E-06	2.76E-05
S100A1	-2.323611987	1.37E-06	3.34E-05
BHMT2	-2.329235566	0.000217404	0.002637516
RP11-417L19.5	-2.331106844	0.000148688	0.001947464
NME5	-2.342692784	9.15E-07	2.37E-05
C8orf46	-2.344415015	2.14E-05	0.000377543
CDHR3	-2.347096044	8.11E-06	0.000160277
AC090587.5	-2.349047702	0.00061586	0.006197371
USP32P2	-2.350038821	0.000143399	0.001889159
MYH11	-2.351149948	0.000824922	0.007855749
A2M-AS1	-2.351572938	0.008225736	0.047726206
ITGA9	-2.351770364	7.21E-05	0.001060681
FRRS1L	-2.353636788	0.000573776	0.005852256
TMEM130	-2.353867344	0.003336351	0.023875227
ADAMTS7	-2.354171192	1.60E-07	5.13E-06
GAS1RR	-2.354352408	3.53E-07	1.04E-05
GFRA1	-2.354747223	8.51E-06	0.000167374
MPZ	-2.356935219	0.002851596	0.021088522
PLAC9	-2.360878912	2.50E-11	1.69E-09
RP11-25K19.1	-2.362100253	2.57E-05	0.000441464
TBX6	-2.370634706	0.00560713	0.035622975
ACP5	-2.373961385	0.005268359	0.034085557
ZNF702P	-2.378023653	0.00464507	0.03078639
COLGALT2	-2.382751509	1.19E-05	0.000224568
DOCK3	-2.385035708	0.000104681	0.001452967
AK4	-2.387472704	2.45E-06	5.62E-05
COL6A3	-2.392695364	1.47E-10	8.82E-09
RASL11A	-2.392841326	3.90E-05	0.000631221
HLA-DPB1	-2.394020342	1.69E-07	5.38E-06
EPOP	-2.394990375	1.53E-05	0.000281569

RPL21P44	-2.397188324	0.00714959	0.042761109
SCAMP5	-2.397531941	0.0003326	0.003733638
GPR68	-2.40096596	0.00140717	0.01215754
CHST15	-2.403638472	2.01E-24	6.98E-22
ADAMTS5	-2.404024482	0.002774687	0.020668946
TMEM229B	-2.404770297	0.00250809	0.019144765
MPV17L	-2.408655893	9.92E-05	0.001387347
NPC1	-2.411498149	0.000895441	0.008420584
RP11-999E24.3	-2.411813246	7.54E-08	2.60E-06
EPHB3	-2.412912105	4.70E-06	9.96E-05
CTD-2012K14.8	-2.419482617	2.22E-06	5.16E-05
C15orf59	-2.419801259	0.000454534	0.004796652
HAGLR	-2.422828875	0.002859035	0.021129674
RP11-46F15.2	-2.42668067	0.00600621	0.037505691
RP11-121P12.1	-2.428207705	0.00200382	0.016068339
COL6A2	-2.429103072	9.20E-14	9.34E-12
ITGB5-AS1	-2.432636165	0.007624386	0.044954559
TUB	-2.435576407	7.31E-11	4.60E-09
ZNF454	-2.438291792	2.39E-06	5.52E-05
CYP46A1	-2.43986896	0.000340176	0.003801634
LINC01140	-2.440433552	0.003138424	0.022754767
PLCE1-AS1	-2.452373522	0.007308675	0.043514222
PNPLA3	-2.453516375	0.000618863	0.006222393
ZNF793-AS1	-2.45508927	0.000927015	0.008666883
TRAF1	-2.459461446	0.00011731	0.001603567
SLC44A3	-2.462098423	0.001478681	0.012649319
TMEM108	-2.467999681	0.000831613	0.007902778
SELENBP1	-2.468433452	1.01E-08	4.29E-07
ABCC6	-2.47009706	0.000695938	0.0068443
FAM106A	-2.470547576	0.001644035	0.013740527
OTUD7A	-2.470646341	0.000571077	0.005830005
CCDC102B	-2.471671746	0.000816428	0.007799324
AC000403.4	-2.471757806	3.86E-09	1.77E-07
MORN3	-2.476239857	0.000178095	0.002247721
GDF5	-2.479398273	0.000156305	0.002026913
HLA-H	-2.480312218	0.000215838	0.002622753
SVIL	-2.480487936	3.61E-07	1.06E-05
THBS1	-2.484514682	4.90E-08	1.76E-06
MYOM1	-2.487802511	2.07E-07	6.45E-06
KIAA1671	-2.488953578	5.82E-05	0.000885561
CRYM	-2.491636674	0.001344427	0.011714232
CD80	-2.492037651	0.002516316	0.019194679
TRIM47	-2.492789239	1.99E-05	0.000353538
CTTNBP2	-2.495768683	0.000132824	0.001775327
PBX4	-2.497126979	0.000203586	0.002504928
ZNF608	-2.498357488	1.34E-06	3.27E-05

KCND2	-2.500480057	0.000474858	0.004973872
RP11-61I13.3	-2.500930519	8.37E-05	0.0012073
LINC00950	-2.503269477	0.000613319	0.006177312
FAM124A	-2.503464191	0.002542378	0.019367268
CTB-51J22.1	-2.504030634	0.000548174	0.005614328
CCDC144B	-2.50852233	0.000604365	0.006111704
SYT7	-2.509424391	0.004681994	0.030976466
FRG1BP	-2.509869318	6.17E-06	0.000125927
ACSL5	-2.512537876	6.15E-05	0.000923801
ADAMTS14	-2.513257064	0.000221445	0.002677894
RP11-552F3.9	-2.51865076	0.001174009	0.010534772
NXPH3	-2.521156208	0.000128084	0.001722636
GDF6	-2.521570638	0.000614217	0.006183592
CCDC170	-2.521716368	2.96E-07	8.89E-06
CTD-2033D15.1	-2.526765932	2.00E-08	7.89E-07
CYP27A1	-2.527101144	0.000158198	0.00203929
SNHG5	-2.533449145	4.51E-08	1.64E-06
RP11-723O4.2	-2.534488754	0.000722371	0.007054843
GNAO1	-2.538201481	1.57E-05	0.000287566
NATD1	-2.545726828	0.000304678	0.003482628
MARVELD2	-2.546551696	5.34E-06	0.000110633
TEC	-2.547345195	1.09E-05	0.000208339
GRIN3A	-2.548469177	0.000158232	0.00203929
LRRN4CL	-2.551391648	0.000423123	0.004537396
STEAP2	-2.558409253	5.30E-06	0.000109879
PATJ	-2.55985322	4.28E-10	2.39E-08
ARHGAP28	-2.559942777	0.000272936	0.003179461
PURG	-2.560144721	0.008631166	0.049467952
TNFRSF14	-2.565814526	0.000247809	0.002939928
KLF8	-2.570527179	1.97E-07	6.18E-06
AMDHD1	-2.57191854	0.000350839	0.003895694
DMGDH	-2.579659871	4.20E-07	1.21E-05
C11orf52	-2.586999421	0.0001068	0.001477824
PTGER4	-2.595603215	5.02E-05	0.000784702
NPAS1	-2.598796884	0.000247285	0.002935256
ELFN1	-2.599067894	0.003418141	0.024262281
LAMC2	-2.600085887	4.98E-05	0.000781371
SPRY1	-2.600621405	3.03E-07	9.07E-06
APOL3	-2.603749165	3.04E-05	0.000507297
TNNC2	-2.605733631	0.00299931	0.021921997
ENTPD1	-2.611800159	1.30E-09	6.64E-08
SYT14	-2.612951771	9.97E-06	0.000192332
SECTM1	-2.613390845	1.88E-06	4.44E-05
LINC01422	-2.618230877	0.000745598	0.007237953
ETV7	-2.619954624	0.008554433	0.049152553
RP11-134G8.7	-2.621923111	0.001199169	0.010700902

SHISA2	-2.623543863	0.005775543	0.036425995
KIF6	-2.625197898	2.48E-05	0.000427773
LINC01285	-2.627494334	0.004510206	0.030131759
RP11-214K3.24	-2.628074735	0.003513691	0.024797154
OSBP2	-2.628864942	1.14E-09	5.87E-08
MGP	-2.629331035	0.001866519	0.015199858
IFI44L	-2.6311788	4.96E-05	0.000779748
RP11-2N1.2	-2.632860707	0.002672066	0.020081327
EGFLAM	-2.639757541	1.67E-09	8.33E-08
GPC4	-2.640783472	0.000204275	0.002507935
RP11-573G6.4	-2.644203697	2.42E-07	7.43E-06
RP11-333I13.1	-2.648545541	0.000457683	0.004821839
CCDC158	-2.660541139	0.003358684	0.023958258
CTD-2334D19.1	-2.661403098	1.47E-06	3.55E-05
IGFBP4	-2.662951581	2.73E-13	2.58E-11
AC007319.1	-2.66752493	0.003911315	0.026970008
FGF7	-2.671600805	4.13E-12	3.13E-10
AC145676.2	-2.672466189	0.002953481	0.021691424
RP11-873E20.1	-2.674904545	1.23E-05	0.000231148
C8orf31	-2.675086449	4.81E-07	1.35E-05
LAMA4	-2.677141068	0.000389154	0.004237641
TSPAN7	-2.678812613	0.002247557	0.017582087
SCNN1A	-2.684339483	6.04E-05	0.000912341
CTD-2033D15.3	-2.689656148	1.33E-05	0.000247058
ZNF395	-2.689820828	5.97E-21	1.45E-18
PLCB4	-2.699930515	9.04E-06	0.000176998
CTD-2207P18.1	-2.704449649	0.006909013	0.041707571
LINC00511	-2.706536134	4.73E-06	9.99E-05
FAM47E	-2.706715338	0.002388593	0.018449994
MYCBPAP	-2.707629054	0.000769356	0.00743656
ZNF560	-2.709165225	0.007844094	0.046045242
ANK1	-2.712718731	8.79E-09	3.77E-07
MIRLET7BHG	-2.717877327	6.96E-08	2.41E-06
KCND3	-2.72116116	3.64E-05	0.00059346
RPSAP70	-2.722367849	0.003975797	0.02731437
KIAA1217	-2.723945369	1.49E-12	1.23E-10
RP11-47I22.2	-2.734504616	6.58E-06	0.0001335
FOXE1	-2.739556293	0.001923172	0.015537715
AK8	-2.743051969	0.00154233	0.013074713
IL16	-2.743160344	0.000299832	0.003435949
FOXD2	-2.751374961	2.84E-06	6.36E-05
RNU7-57P	-2.756197034	0.004839847	0.031790816
RP11-182J1.18	-2.756664069	0.000743979	0.007225349
ALOX12P2	-2.757431527	2.58E-06	5.89E-05
AUTS2	-2.766572267	1.30E-50	3.66E-47
CRYAB	-2.768221699	6.08E-05	0.000915267

RP11-693N9.2	-2.770655526	0.002750273	0.020536804
MUSK	-2.772895843	0.001545234	0.013079657
RDH5	-2.779026225	1.44E-09	7.26E-08
NTN4	-2.780712933	1.39E-05	0.000258472
SERPINA1	-2.784113046	2.74E-06	6.17E-05
ENPP4	-2.784668494	8.13E-07	2.14E-05
OAS2	-2.787030707	1.15E-05	0.000217513
PCSK1	-2.787809276	6.54E-05	0.000973133
B3GALT1	-2.790820945	0.007753546	0.045598747
G0S2	-2.794014396	0.000612451	0.006174086
BBOX1-AS1	-2.794633868	0.006708062	0.040767435
PALMD	-2.797722403	4.46E-05	0.000712633
CTC-340A15.2	-2.799519669	0.003548983	0.024983646
YPEL1	-2.806230346	4.38E-06	9.36E-05
P2RY2	-2.810196482	0.001089648	0.009895951
TUBA4A	-2.81034179	5.00E-09	2.26E-07
LINC01111	-2.812900687	0.000424538	0.004548244
RP11-225H22.4	-2.815599023	0.006405844	0.039348067
RP11-271K11.5	-2.816461181	0.006224727	0.03855773
GPAT2	-2.818187706	1.25E-05	0.000234843
ADAMTSL3	-2.824028017	0.002021544	0.016170202
MKRN7P	-2.831861358	0.001496368	0.012761871
AF131217.1	-2.833734549	9.37E-05	0.001323709
PEG3	-2.839352365	0.000141883	0.001872469
ABLIM1	-2.839736102	4.16E-08	1.53E-06
DBP	-2.852908419	0.000164271	0.002105077
RP11-494M8.4	-2.853422077	0.008363885	0.048390741
HTRA1	-2.854686901	1.43E-07	4.64E-06
CGREF1	-2.85657415	0.000141882	0.001872469
NBL1	-2.857625579	1.14E-19	2.37E-17
KRT86	-2.863806307	0.00155591	0.013160134
FBXO32	-2.865337888	0.000124247	0.001681771
MAF	-2.867940268	0.000191655	0.002385454
XXbac-BPG248L24.12	-2.868748395	1.64E-05	0.00029931
CYP26B1	-2.869717963	0.00597885	0.037421028
CTC-558O2.2	-2.876283777	0.001458659	0.012519239
CTD-2517O10.6	-2.877966087	0.001443332	0.012398683
ZNF883	-2.878815986	4.94E-13	4.53E-11
ZNF658B	-2.8795869	6.78E-05	0.00100161
ADAMTSL4	-2.88336484	6.14E-15	7.44E-13
B4GALNT3	-2.887351337	0.000491498	0.005114894
KRT23	-2.888540967	0.006145611	0.038193606
RP5-1021I20.1	-2.889733328	0.004924439	0.032286734
ZNF391	-2.895405544	1.71E-08	6.84E-07
TRIB2	-2.906427771	8.33E-15	9.88E-13
GNG7	-2.910551251	0.000781683	0.007526659

TNFSF9	-2.912298149	1.55E-06	3.73E-05
MUC20	-2.920944308	2.19E-05	0.000385386
SLC37A1	-2.930587962	9.11E-07	2.36E-05
RP11-42I10.1	-2.93190535	0.000260454	0.003060945
LMO7DN	-2.933907977	0.001060683	0.009685953
THBS4	-2.936650125	0.001197286	0.010688326
AC017060.1	-2.944214069	0.000450598	0.004759574
CSTA	-2.944599777	2.88E-05	0.000487065
KB-1995A5.5	-2.947947646	0.001509048	0.012836018
PDK4	-2.952082096	1.93E-05	0.000344656
RP11-144I2.1	-2.953313951	0.000344088	0.003839649
ZNF663P	-2.955736364	0.005163178	0.033559165
PARP15	-2.957569941	7.64E-09	3.32E-07
CCNO	-2.961364817	0.003176819	0.022966218
FAM13C	-2.964975673	2.97E-05	0.000498492
NRG2	-2.96965401	0.000467315	0.004906261
RHD	-2.974966037	0.003923411	0.027020349
ABCA6	-2.977530006	6.32E-05	0.000946379
PCDHB12	-2.988698924	0.000279397	0.003239658
CCBE1	-2.988844152	5.60E-08	1.98E-06
RPL10P9	-2.992476924	8.04E-07	2.13E-05
NCAPGP1	-2.995858892	0.007939266	0.046458719
RP11-214K3.21	-3.005819047	9.62E-05	0.001353
VWCE	-3.013671505	5.24E-05	0.000812541
LINGO1	-3.016499835	0.007502912	0.044395849
NXPE2	-3.016953232	0.003037198	0.022119265
RP11-446H18.5	-3.019118529	0.001044286	0.009553269
CXCL16	-3.02198697	1.72E-06	4.10E-05
AXIN2	-3.028749271	5.29E-14	5.62E-12
SYT16	-3.029958159	0.008359585	0.048378282
MARVELD3	-3.030379189	0.007959391	0.046564403
LRFN1	-3.032152524	0.000153814	0.002000653
RP11-326C3.11	-3.033498291	6.41E-05	0.000956106
MROH7	-3.036979952	0.000107448	0.001485877
METTL7A	-3.042467646	8.28E-09	3.58E-07
CADPS2	-3.042770786	2.95E-09	1.39E-07
SLC2A5	-3.043687884	8.39E-10	4.46E-08
CASC15	-3.047419698	8.38E-05	0.001208418
CCR7	-3.04790984	0.000717461	0.007016871
TTLL9	-3.05440134	0.005364059	0.034506635
GRB14	-3.056233946	0.000776672	0.007487122
CTD-2547L24.3	-3.058665441	0.001352208	0.011763844
TMC7	-3.060365942	6.71E-11	4.26E-09
SNTB1	-3.060702804	0.000128959	0.001732344
RP11-469A15.2	-3.061679736	0.000660424	0.006557985
FRZB	-3.063730526	1.80E-09	8.93E-08

CCDC151	-3.06714487	3.48E-05	0.000569716
EFHC2	-3.06871455	2.18E-06	5.08E-05
DIRAS1	-3.073456476	3.32E-13	3.09E-11
TSPAN32	-3.096018267	0.000583262	0.005928541
C19orf38	-3.096928678	0.000748295	0.00725788
ARL4C	-3.097481435	3.03E-09	1.42E-07
PCDHB16	-3.097945396	1.50E-07	4.85E-06
GALNT16	-3.108605461	5.27E-06	0.000109228
CTD-3116E22.7	-3.112646311	0.000631835	0.006338316
OLFML2A	-3.116824081	3.31E-06	7.30E-05
ZDHHC15	-3.118088975	0.001856466	0.01512892
LINC00664	-3.122604244	0.007109715	0.042565757
SLC12A5	-3.122628248	0.001965997	0.015821328
LINC01435	-3.126005053	0.000830581	0.007896304
SLC25A27	-3.127183093	7.89E-06	0.000156765
TOX	-3.134440338	3.72E-11	2.45E-09
LINC02021	-3.136483617	2.35E-05	0.000410217
FAM157C	-3.137531226	0.005429663	0.034799558
RP11-723O4.9	-3.139248804	0.000313694	0.003561676
TPTE2P1	-3.140852429	0.002362978	0.018297456
PCDHB15	-3.147545167	2.38E-05	0.000413102
RP11-141C7.2	-3.150778166	7.65E-07	2.04E-05
EPHA5	-3.155430017	0.000479976	0.005013059
HAPLN3	-3.15567869	8.66E-09	3.72E-07
C8orf34	-3.16088039	3.71E-08	1.38E-06
MLC1	-3.161161818	0.000328197	0.003697094
LRP1-AS	-3.164332621	0.004047338	0.027712951
CDO1	-3.164380031	1.61E-22	4.28E-20
FLJ22447	-3.169682175	1.15E-12	9.68E-11
VSTM4	-3.177903175	9.77E-15	1.15E-12
PCDHB3	-3.183633028	0.000113459	0.001561343
THRB	-3.183811691	2.88E-06	6.44E-05
RP11-27G24.3	-3.186830128	0.003794641	0.026350898
ISL2	-3.187297622	0.002985679	0.021836529
PRTG	-3.190527157	9.16E-06	0.000178651
PHEX	-3.193350401	5.80E-08	2.04E-06
CACNA1C	-3.194123734	8.03E-07	2.13E-05
HHIPL2	-3.201083817	0.000255307	0.00300987
FZD3	-3.203916391	5.00E-06	0.000104357
HTRA3	-3.211986782	4.62E-09	2.10E-07
SYTL2	-3.216974898	2.92E-23	8.78E-21
IL34	-3.217752987	0.006125804	0.038102028
CFD	-3.218924917	0.000164623	0.002108386
EPB41L4B	-3.222424726	1.10E-06	2.76E-05
RFX8	-3.230750268	1.02E-09	5.31E-08
SLC5A9	-3.235677657	0.004351335	0.029278642

NEK10	-3.240569097	3.94E-08	1.45E-06
AC010524.2	-3.245902317	0.004935783	0.032314111
RP11-356M20.3	-3.258083248	0.004460144	0.029857629
RP11-301L8.2	-3.259149863	0.000331786	0.003726354
LMO2	-3.261438033	7.04E-05	0.001036952
IPCEF1	-3.26180483	0.00141425	0.0122
RP11-713M15.2	-3.263034856	0.003581713	0.025166895
FGF14	-3.263197723	8.15E-14	8.35E-12
GCNT4	-3.264746265	0.000207022	0.002530633
FBLN7	-3.275121441	7.28E-20	1.58E-17
NMNAT3	-3.275813496	0.001278349	0.011246909
VWA5A	-3.276044662	3.94E-07	1.14E-05
HERC2P10	-3.278605145	0.000225414	0.002712677
ARHGEF16	-3.283641897	0.000313932	0.003561676
TXLNB	-3.283653717	0.005305181	0.034268103
CMAHP	-3.284672409	7.77E-14	8.00E-12
RP11-367G18.1	-3.288203209	0.004302546	0.029028342
ISLR	-3.289666404	1.39E-32	1.05E-29
RP11-861E21.1	-3.290735674	0.002164484	0.017017657
RPL10P6	-3.29228538	3.18E-06	7.05E-05
CELSR1	-3.293572011	2.57E-06	5.88E-05
HEY2	-3.299043363	0.000222815	0.00269301
RP11-423H2.3	-3.302744204	0.000653542	0.006512598
IL20RA	-3.305873411	0.002880271	0.021248402
CTD-2303H24.2	-3.306102434	4.64E-06	9.84E-05
GRIA1	-3.307170346	0.000281853	0.00326477
ZNF135	-3.30791286	3.26E-23	9.68E-21
LYPD6B	-3.310328997	1.11E-05	0.000210928
TMEM100	-3.311030923	0.000178721	0.00225436
RP11-624L4.1	-3.312126122	2.85E-06	6.37E-05
TNFRSF11A	-3.313282652	0.00068569	0.006755688
TBC1D8	-3.315077715	3.99E-20	8.99E-18
AC002480.5	-3.319403129	0.007599979	0.044857589
C12orf56	-3.321556091	0.001320564	0.011542605
PADI2	-3.324873753	2.64E-07	8.01E-06
SATB2	-3.331545789	8.41E-15	9.92E-13
PWP2	-3.343093964	0.000481985	0.005029823
CFI	-3.344594288	7.53E-05	0.001099611
KCTD12	-3.347748224	2.13E-08	8.38E-07
ZNF208	-3.351177569	0.002574217	0.019550348
OTOGL	-3.352340288	0.000123429	0.001672005
PROS1	-3.352899631	6.05E-13	5.42E-11
OASL	-3.360673022	0.000460849	0.004847408
ESM1	-3.36493655	0.000453487	0.004787848
GRM6	-3.365535057	0.000373086	0.004088352
VSNL1	-3.367746123	1.55E-06	3.73E-05

ANK3	-3.371674605	1.96E-07	6.16E-06
RP11-81H14.2	-3.373263536	0.005048738	0.032938812
PCDHB7	-3.376333366	1.07E-09	5.52E-08
APOD	-3.381063123	0.001931505	0.015588273
RP5-1120P11.1	-3.381419759	0.000492024	0.005118002
CNGA3	-3.385136678	0.003988988	0.027379954
LINC00856	-3.390814702	3.24E-05	0.000537303
ALX4	-3.395784726	3.77E-07	1.10E-05
PCDHB10	-3.397725281	2.19E-07	6.77E-06
AC005532.5	-3.398965393	1.07E-05	0.000204261
RP11-68I18.10	-3.40009253	0.003087264	0.022441156
C1QTNF3	-3.403010428	1.88E-06	4.45E-05
AC131056.3	-3.403100734	0.002054193	0.016379049
IGDCC4	-3.407662531	6.73E-07	1.82E-05
AC010878.3	-3.410107083	0.001101564	0.009984058
FAM78B	-3.413513202	0.002441845	0.018740768
RP11-399B17.1	-3.413748385	1.43E-08	5.87E-07
EDNRB	-3.414225837	0.000337884	0.003783523
CIITA	-3.415769644	1.74E-13	1.70E-11
CNTNAP3	-3.420425324	0.005824947	0.036667026
HSPB2	-3.421142943	1.23E-10	7.48E-09
CNTNAP3P2	-3.464996456	0.000218203	0.00264436
RP3-395M20.8	-3.468254384	0.000997068	0.00919972
TLE2	-3.475436953	4.71E-05	0.000747297
CILP2	-3.48041835	3.90E-05	0.00063125
SLC16A6	-3.485100955	0.001078709	0.009816382
AC083949.1	-3.487162576	0.002431792	0.018701819
LRRTM2	-3.496812685	8.69E-05	0.001243981
KIAA1755	-3.497274941	3.05E-05	0.000508434
CTD-2066L21.3	-3.499745654	3.44E-05	0.000565249
RP11-145A3.1	-3.50125601	3.05E-07	9.10E-06
ABCA13	-3.501506791	1.05E-06	2.67E-05
SPAG17	-3.507803652	3.01E-08	1.14E-06
PLA1A	-3.514938675	5.31E-05	0.000820859
ARHGAP6	-3.525485219	1.85E-12	1.49E-10
PCDHB11	-3.530169067	4.42E-06	9.42E-05
FOXQ1	-3.531913988	0.000116388	0.001592895
C11orf88	-3.53428734	0.003151033	0.022814817
PROM2	-3.53668216	0.000916783	0.008581899
ZNF385D	-3.550989734	9.41E-12	6.76E-10
RP11-16P20.4	-3.563770943	4.43E-05	0.000708449
RP11-284M14.1	-3.566388819	0.00382722	0.026519942
PCDHB13	-3.568161218	4.90E-08	1.76E-06
PRCD	-3.570045337	4.18E-10	2.34E-08
CCRL2	-3.570290783	0.001797721	0.014724807
PDE5A	-3.576954905	3.62E-11	2.39E-09

RP4-668J24.2	-3.577626564	0.003769881	0.026227437
PCDHB8	-3.578500757	1.74E-05	0.000314916
SEMA3E	-3.580818092	5.04E-05	0.000787607
FMN1	-3.589191841	3.21E-10	1.83E-08
BCL2	-3.590833366	7.66E-10	4.10E-08
NKAIN2	-3.594283356	9.32E-08	3.14E-06
LINC00882	-3.598532692	3.26E-10	1.86E-08
ZNF415	-3.600709483	1.09E-08	4.57E-07
COL21A1	-3.601243542	0.00013128	0.00175828
MTSS1	-3.605237642	8.13E-08	2.78E-06
RAI2	-3.606764534	0.001168305	0.010496122
SMILR	-3.609051322	0.001173645	0.010534772
VCAM1	-3.60966336	3.25E-06	7.18E-05
PAX9	-3.62273467	1.18E-05	0.000222152
KAZALD1	-3.627613718	1.14E-10	6.99E-09
PCDHB2	-3.639039334	0.000659714	0.006553814
IRX3	-3.641745007	2.12E-32	1.54E-29
ITGBL1	-3.646029263	2.02E-14	2.28E-12
PARK2	-3.648690421	4.57E-07	1.30E-05
RASA4	-3.652903794	1.89E-08	7.53E-07
SUSD2	-3.66075919	2.79E-08	1.07E-06
KRT81	-3.667006666	0.002549903	0.019398369
RIPK3	-3.684185022	2.03E-07	6.35E-06
CD4	-3.689052288	1.57E-11	1.10E-09
RP11-755E23.2	-3.691124751	0.001184426	0.010601415
MYL4	-3.694751436	0.00065445	0.006518761
RP11-800A3.4	-3.694945207	9.67E-05	0.00135785
INMT	-3.695422474	0.000176979	0.002236147
CTD-2554C21.2	-3.697081883	5.38E-08	1.92E-06
PRELP	-3.70373109	1.29E-15	1.66E-13
PPARG	-3.705470578	1.54E-05	0.000283658
FAM87B	-3.712043827	8.81E-12	6.39E-10
EGR2	-3.716837084	4.94E-05	0.000777493
LY75	-3.717004578	4.63E-06	9.83E-05
TNFRSF1B	-3.717235615	9.97E-14	1.01E-11
RP11-400K9.4	-3.724916732	8.12E-07	2.14E-05
KIAA1644	-3.728195351	6.88E-18	1.20E-15
ERG	-3.736473606	4.38E-06	9.36E-05
GLIPR1L2	-3.737705326	0.000774928	0.007477591
bP-2171C21.5	-3.740803662	0.008000174	0.04674821
COL28A1	-3.753069649	0.001120852	0.010126303
IRF6	-3.759573648	4.87E-06	0.000102274
MYO1D	-3.763634375	3.07E-09	1.44E-07
DAAM2	-3.764150658	6.52E-07	1.77E-05
EFEMP1	-3.766004997	1.58E-08	6.37E-07
GLIPR1L1	-3.771120482	0.001055388	0.00964309

KCNJ15	-3.771285629	3.40E-08	1.27E-06
LEF1	-3.779100521	0.002861183	0.021132204
CYP19A1	-3.784677443	0.003308163	0.023748183
CILP	-3.786496924	0.006373906	0.039212394
COL15A1	-3.792905017	5.73E-05	0.000874979
HSD11B1	-3.792924624	0.001370893	0.011889687
RP11-361F15.5	-3.80148753	0.0036494	0.025531075
PIFO	-3.8135708	0.000234367	0.002798126
KCNJ6	-3.826187773	3.45E-07	1.01E-05
PCA3	-3.830131063	0.001651225	0.013779081
EPYC	-3.831032535	0.001287262	0.011312348
PCDHB14	-3.835691999	2.17E-09	1.05E-07
HLA-DMA	-3.838619684	3.96E-28	1.82E-25
KANK4	-3.839930743	0.006222483	0.038554424
RP1-124C6.1	-3.84068671	0.002053182	0.016376778
ANO5	-3.847534451	1.04E-07	3.46E-06
NPR1	-3.857222976	0.007894856	0.046282952
AC116614.1	-3.863554393	0.00177118	0.014573386
GOLGA8O	-3.865198018	0.002644178	0.019967308
PINLYP	-3.866472549	2.47E-06	5.67E-05
YAP1P1	-3.867563665	1.77E-06	4.21E-05
KRTAP1-5	-3.875310498	0.002984206	0.021836529
TRIM9	-3.875728222	0.000350264	0.003891226
PLAU	-3.885912794	3.32E-43	4.40E-40
NDNF	-3.888378546	2.40E-05	0.00041628
ZNF826P	-3.890737713	4.91E-14	5.30E-12
KLKB1	-3.89319195	9.20E-08	3.11E-06
SCUBE3	-3.893221471	0.007646221	0.045059732
BMF	-3.903155295	3.87E-09	1.78E-07
RTP4	-3.904919072	9.22E-05	0.001309804
RP3-439F8.1	-3.905715391	0.00049655	0.005160315
TF	-3.912867592	4.55E-06	9.68E-05
C4B	-3.922726033	0.000793622	0.007613871
ADD3-AS1	-3.923542855	0.000274267	0.003193317
EGR3	-3.936836364	1.19E-12	9.96E-11
RP11-466A19.1	-3.937838489	0.000126381	0.001703794
EMCN	-3.943148677	0.000610916	0.006164124
CACNA1D	-3.950425528	0.000323368	0.00365365
RASSF9	-3.950510409	2.39E-18	4.28E-16
ZSCAN1	-3.954869433	0.005350682	0.034475267
KCNMB2	-3.956657239	9.35E-05	0.00132361
PDE1A	-3.959923136	1.46E-07	4.73E-06
RXFP1	-3.960967491	0.005625151	0.03571733
PLBD1-AS1	-3.972083261	0.005036274	0.03286701
DCC	-3.976958803	8.67E-06	0.000170278
EBF1	-3.979913823	2.03E-07	6.35E-06

GGTLC4P	-3.984029611	1.91E-05	0.000341203
RP11-466A19.8	-3.989867058	0.004326334	0.029163582
NKX3-2	-3.990953167	6.05E-05	0.000913171
C10orf55	-3.992565837	3.99E-07	1.15E-05
CYP2A7	-3.993215279	7.81E-11	4.90E-09
PGF	-3.995833945	3.36E-08	1.26E-06
RASGEF1B	-4.003741754	0.00138687	0.012005161
NPTX2	-4.009364013	0.00039431	0.004281381
ZNF541	-4.009945747	0.003272836	0.0235546
AC112198.1	-4.011268069	0.002607203	0.019743356
SLC17A7	-4.01803835	2.08E-06	4.87E-05
RP11-588H23.3	-4.02711691	1.91E-09	9.37E-08
LINC00900	-4.03059095	1.68E-12	1.37E-10
C11orf96	-4.030745388	0.004226283	0.028633798
PLPP3	-4.034588361	5.24E-23	1.46E-20
COLEC12	-4.03617513	0.004073274	0.027844221
TDRD6	-4.048977889	5.09E-07	1.42E-05
TEX41	-4.049306688	7.59E-12	5.54E-10
LINC01018	-4.052163995	2.38E-06	5.51E-05
FIRRE	-4.052525061	0.008744088	0.049899875
RP11-494K3.2	-4.052967249	0.00041754	0.004488205
CRLF2	-4.059672712	0.001921035	0.015527753
PPP1R14A	-4.066551294	3.66E-08	1.36E-06
PCDHB5	-4.067911057	1.86E-13	1.79E-11
RARRES3	-4.073891076	2.90E-05	0.000489052
RSAD2	-4.074825437	0.000945784	0.008809471
RP11-822E23.6	-4.100269339	0.000140453	0.00185948
SLC12A1	-4.107195172	0.003311386	0.023756193
CDH3	-4.108283903	0.002836728	0.021006137
C9orf106	-4.118041354	0.000355201	0.003930573
CTD-2561J22.5	-4.119015301	0.000635599	0.006361921
RGN	-4.133383753	1.31E-10	7.94E-09
ADGRE2	-4.137813084	0.006640485	0.040436239
EXTL1	-4.1378255	2.73E-06	6.15E-05
PITPNM3	-4.144141998	5.67E-14	5.95E-12
CST6	-4.146482809	1.15E-05	0.00021795
RP11-666A8.7	-4.148973068	0.000382355	0.004175711
TRIML2	-4.15447626	0.001710596	0.01419718
LAMA3	-4.160035625	1.08E-10	6.69E-09
ITGB2-AS1	-4.160596841	6.36E-10	3.45E-08
ANKFN1	-4.188866307	0.006576921	0.040184497
RP11-387A1.5	-4.188931516	0.007055558	0.042358645
LTF	-4.190947125	0.004297084	0.029000169
SCN1A	-4.197739938	8.42E-05	0.001212145
SLC7A8	-4.205969315	8.02E-10	4.28E-08
VEGFD	-4.210562481	0.002465421	0.018876715

LGI2	-4.228975782	0.004084179	0.027898642
NINJ2	-4.229723001	1.03E-07	3.43E-06
GALNT15	-4.250700214	1.12E-13	1.13E-11
RP11-397O8.7	-4.295815309	4.26E-05	0.000683858
CKB	-4.298856693	0.000359863	0.003974363
CYP39A1	-4.299839893	9.96E-05	0.001392007
ANKRD20A5P	-4.301861553	1.42E-06	3.45E-05
AC002480.3	-4.303776069	3.15E-08	1.19E-06
SLC30A3	-4.306019179	0.000153692	0.002000208
NOTCH3	-4.316842646	1.25E-08	5.18E-07
DRAXIN	-4.317985884	0.000175981	0.002226026
PTGDR	-4.322496644	0.006503	0.039821819
NFE2	-4.322575127	0.000349958	0.003889743
NRN1	-4.33348118	0.00370174	0.025849111
KCNH1	-4.333540556	0.004922822	0.032285522
IGFBP5	-4.337305101	4.22E-07	1.21E-05
LRRC4C	-4.340458833	7.06E-06	0.000141526
DLX2	-4.344673097	1.01E-06	2.57E-05
IL21R	-4.353982471	4.72E-06	9.98E-05
RP11-415J8.7	-4.358015871	0.006066615	0.037817359
C2	-4.359024623	1.40E-07	4.55E-06
AC016582.2	-4.360388539	0.00059926	0.00606552
SIM1	-4.362044564	2.48E-05	0.000427502
POM121L9P	-4.362728068	1.85E-07	5.84E-06
PTPN20	-4.385083424	3.01E-05	0.00050316
CYP1B1-AS1	-4.392470745	2.92E-08	1.11E-06
RIMS1	-4.398719964	7.75E-09	3.36E-07
DMKN	-4.401459707	0.000480156	0.005013059
SERPINF1	-4.403668778	6.76E-07	1.83E-05
ACKR3	-4.418872104	3.83E-11	2.51E-09
SMN2	-4.419162012	4.40E-15	5.39E-13
RP11-466A19.4	-4.435517258	0.006776771	0.041096364
SULT1C2	-4.437099866	0.004428284	0.029716566
CYP2C8	-4.445887277	0.006221227	0.038554424
SLC1A7	-4.457490731	1.58E-08	6.37E-07
STEAP1B	-4.460216282	7.51E-22	1.90E-19
RANBP3L	-4.465047281	3.10E-09	1.45E-07
ACP7	-4.466976394	0.00016401	0.002102928
RAPGEF5	-4.481109575	1.06E-05	0.00020246
LANCL3	-4.481302145	2.60E-10	1.50E-08
TMEM255A	-4.481796897	0.000271701	0.003168343
ERMN	-4.482640476	1.46E-05	0.000270279
LL22NC03-N14H11.1	-4.489669735	0.002910166	0.021451292
RNF182	-4.494707992	2.44E-06	5.61E-05
CES1	-4.497272112	6.85E-06	0.000138332
STAC2	-4.51044051	0.000231704	0.002772205

C5orf38	-4.512734935	0.004514598	0.030134305
PRRX2	-4.513417781	6.18E-13	5.51E-11
ACSM3	-4.52427441	0.000605371	0.006119134
BBOX1	-4.52656661	0.004156459	0.028266279
HAS1	-4.531124175	6.34E-10	3.44E-08
EVI2B	-4.536287195	1.85E-06	4.39E-05
TCF7	-4.547533167	3.33E-08	1.25E-06
NTRK1	-4.550131033	0.000309799	0.003530419
SPESP1	-4.551937714	6.89E-06	0.00013894
SFTA1P	-4.562044436	4.69E-16	6.22E-14
WISP1	-4.563836546	9.64E-12	6.90E-10
SMC2-AS1	-4.582294335	3.33E-05	0.000550201
ZNF423	-4.584323914	1.06E-16	1.54E-14
RGL3	-4.59662175	0.003137473	0.022754767
C10orf11	-4.598429703	1.64E-06	3.94E-05
ANKRD65	-4.617501476	5.25E-06	0.000108933
RP11-388P9.2	-4.618946867	0.005508727	0.035156346
AC073621.2	-4.6226829	0.002858056	0.021129365
ALS2CR11	-4.623846773	1.91E-19	3.91E-17
CHMP1B2P	-4.635710403	0.00567373	0.035948214
RP11-395N3.2	-4.642917068	8.36E-07	2.19E-05
PLXNA4	-4.651277193	1.51E-28	7.39E-26
RP11-585P4.6	-4.651621962	0.004609446	0.030595263
RP5-1054A22.4	-4.652771038	0.008321184	0.048193168
PRUNE2	-4.655581517	2.01E-18	3.62E-16
CH25H	-4.675817379	4.72E-05	0.000748207
LEPR	-4.684485279	3.06E-16	4.24E-14
LINC00840	-4.687684257	0.00283799	0.021008582
CCL28	-4.688722757	5.42E-09	2.42E-07
HECW1	-4.688999111	1.13E-08	4.73E-07
ACADL	-4.694716537	0.001608529	0.013513919
RP1-28O10.1	-4.697545575	0.001470376	0.012602754
RP11-321G12.1	-4.702196037	0.007561537	0.044676407
IFITM10	-4.707280581	1.42E-15	1.80E-13
MYH14	-4.710115225	5.52E-06	0.000113886
RP3-425P12.4	-4.710367414	0.004774375	0.03148601
KCNB1	-4.711168509	1.06E-07	3.49E-06
IQCA1	-4.712434987	2.71E-06	6.13E-05
EXOC3L2	-4.733713157	0.003337511	0.023875227
RP11-815M8.1	-4.734910222	0.001505744	0.012817587
NHS	-4.741334147	9.61E-20	2.06E-17
PODN	-4.749143395	1.92E-12	1.54E-10
CECR7	-4.757168835	0.000496131	0.005158339
RP11-39M21.2	-4.757781172	0.005832311	0.036671163
WNT16	-4.761524704	0.000103604	0.001440682
FOXC1	-4.772124032	7.55E-14	7.80E-12

RNF212	-4.776596374	3.49E-16	4.80E-14
ZNF503-AS1	-4.777063479	0.004953714	0.032403267
PPARGC1A	-4.77804838	3.01E-05	0.00050316
RASD1	-4.783041988	3.99E-15	4.91E-13
RP11-679B19.1	-4.787973513	0.000471535	0.004945958
CEBPA	-4.797987598	3.01E-07	9.01E-06
PPL	-4.806717987	1.58E-06	3.80E-05
COL10A1	-4.811319736	2.25E-08	8.76E-07
TBX15	-4.815295108	0.006230735	0.038584338
CPZ	-4.826313135	6.44E-07	1.75E-05
WSCD1	-4.827187258	0.002008785	0.016086444
CACNA1G	-4.827612997	6.67E-06	0.000135015
EMX2	-4.830131538	0.001719051	0.014245676
KCNK15-AS1	-4.835352778	0.000263034	0.003084638
P2RX7	-4.851667325	5.07E-08	1.82E-06
ZNF835	-4.856197343	0.002413746	0.018594756
GREM2	-4.857150982	4.55E-05	0.000725509
KRT222	-4.858666698	0.005722701	0.036183844
ANKRD29	-4.865688687	3.11E-10	1.78E-08
AC002480.4	-4.866284846	5.74E-07	1.58E-05
DUXAP10	-4.87269202	1.59E-05	0.000291436
COL14A1	-4.882100837	8.20E-12	5.96E-10
AC011747.6	-4.882148861	0.002635317	0.019913735
CYYR1	-4.887606008	1.18E-05	0.000222152
CXCL12	-4.893546986	5.71E-51	1.84E-47
CKM	-4.908534393	0.000797056	0.007638794
PPFIA2	-4.911614687	0.003230961	0.023282956
RP11-168K11.5	-4.919865489	0.000290593	0.003348804
AP000473.8	-4.930985487	0.002396104	0.018490443
CTB-139P11.2	-4.933669132	0.004779996	0.031504647
FAM225B	-4.938286919	8.24E-09	3.56E-07
LINC00839	-4.940241891	0.003931652	0.027060572
C16orf47	-4.95526849	0.002266889	0.017698112
PLXDC1	-4.974432266	4.74E-21	1.16E-18
AC099684.1	-4.976077033	0.007962015	0.046567667
PALM	-4.977257058	0.000163725	0.002100476
NGFR	-4.978035707	4.30E-11	2.80E-09
OLFML2B	-4.982227423	1.04E-29	6.01E-27
CTD-2595P9.4	-4.985941981	0.004196472	0.028474618
HLA-DPA1	-4.994204218	4.31E-23	1.23E-20
AC007618.3	-5.004602799	0.008314491	0.048166778
RP11-47G11.2	-5.008569972	0.004423375	0.029692468
HEPH	-5.014834832	9.60E-05	0.001351431
TMED11P	-5.018162881	0.001075117	0.009791601
ALMS1P1	-5.021185741	0.007020835	0.042213026
CD22	-5.029842331	0.006172413	0.038349606

SLITRK1	-5.032234238	0.000349762	0.003889485
RSPH1	-5.034322266	0.000434204	0.00463054
LINC01936	-5.035425302	0.008009823	0.046773933
RP3-488M23.3	-5.037376603	0.003661996	0.025603305
CHRDL1	-5.054670326	0.000710638	0.006967586
GPX3	-5.055975914	6.67E-11	4.25E-09
FNDC1-IT1	-5.059714945	0.001471561	0.012602754
CYTIP	-5.06234717	0.008436235	0.048686594
RMDN2-AS1	-5.065319688	5.21E-09	2.33E-07
TTC9	-5.072463982	2.83E-09	1.34E-07
PCDHB6	-5.072993108	3.06E-07	9.13E-06
MCOLN3	-5.073672461	3.77E-11	2.48E-09
RP11-168F9.2	-5.075064811	0.001102577	0.009988978
CST1	-5.07590799	0.001850005	0.015098102
SFRP1	-5.077627239	5.86E-05	0.000890638
MPPED2	-5.081129605	5.86E-06	0.000120319
CACNA1C-AS2	-5.08867963	0.0009773	0.009043235
PDE9A	-5.089728323	0.006332125	0.03907266
RIPK4	-5.090540482	6.26E-06	0.000127422
CCR1	-5.106607224	0.002491328	0.019055661
VIT	-5.109401088	2.27E-12	1.80E-10
CTD-2012J19.2	-5.11132247	0.00117735	0.010547951
SERPINA5	-5.113507322	0.003132341	0.022739483
AC064875.2	-5.114401774	0.002184027	0.017153361
MUC4	-5.119791118	0.002190441	0.017197749
LINC01655	-5.129108902	0.001758484	0.014503472
ANKRD45	-5.130970026	0.002660633	0.020051261
RP11-475I24.3	-5.137579907	0.004514471	0.030134305
AC069513.4	-5.141830313	0.000469382	0.004925672
C21orf33	-5.145692651	8.76E-07	2.28E-05
INA	-5.149858632	8.37E-05	0.0012073
RARRES2	-5.152069013	0.000275307	0.00320377
LINC01013	-5.160781185	1.22E-08	5.04E-07
RP11-757O6.6	-5.165255292	0.002952952	0.021691424
CTC-239J10.1	-5.167388444	0.004222702	0.028626745
RP11-403F21.4	-5.17484653	0.002629678	0.019877794
FBP1	-5.175199991	0.002073869	0.01648927
TMEM150C	-5.176488341	1.05E-16	1.54E-14
RP11-195E2.1	-5.187522983	0.003518277	0.024813982
LINC00982	-5.188840099	4.49E-10	2.51E-08
ZMYND15	-5.197258321	0.002897019	0.02136137
INHBB	-5.201711216	1.08E-06	2.73E-05
NGEF	-5.204479095	1.71E-09	8.51E-08
SYT8	-5.208622397	0.00328021	0.023600131
PRRG4	-5.21233988	0.000340903	0.003807873
RP11-234G16.5	-5.213177269	0.005169729	0.033583978

FOXF2	-5.214214468	2.10E-09	1.03E-07
RIMS3	-5.223829275	6.90E-05	0.001018437
OLFML1	-5.225964718	1.23E-23	3.96E-21
LINC01268	-5.230110982	1.02E-19	2.16E-17
NLRP10	-5.231864427	5.17E-14	5.52E-12
HRASLS5	-5.234798667	0.003635602	0.025446985
TSHR	-5.236836984	2.77E-05	0.00046969
NKX6-1	-5.247349638	0.000362451	0.003997068
SCARA5	-5.247709635	0.001321145	0.011542605
RP11-403B2.7	-5.25435772	0.000413288	0.004448869
LINC01956	-5.260480028	4.71E-06	9.97E-05
AC093702.1	-5.261255056	0.000367523	0.004041135
RNU6-342P	-5.269100186	0.002303993	0.017926929
ISLR2	-5.272355505	1.28E-05	0.000239826
SVILP1	-5.272891011	6.30E-09	2.77E-07
ANGPTL5	-5.274959006	2.72E-06	6.15E-05
AC003090.1	-5.275527413	0.002446867	0.018766533
ADCY4	-5.286698745	9.24E-08	3.12E-06
EMX2OS	-5.297185505	0.006549107	0.040049761
MAFB	-5.315430884	1.47E-08	6.02E-07
RP11-20I20.1	-5.316536021	0.000537966	0.005521986
RP11-70D24.2	-5.31719736	0.0002421	0.002881295
CTD-2341M24.1	-5.319588656	0.003373729	0.024035151
TMEM119	-5.322715588	1.14E-16	1.64E-14
AC092535.3	-5.322871248	0.00077221	0.00745775
RP11-278A23.2	-5.327228816	0.001241187	0.010984528
LINC01114	-5.341028269	0.000474716	0.004973872
TRABD2B	-5.352852034	6.25E-09	2.76E-07
AC073115.7	-5.354988078	0.002280872	0.01779617
ASB5	-5.355678168	4.62E-16	6.19E-14
MTATP6P16	-5.370218501	0.000996979	0.00919972
FEM1AP2	-5.37075588	0.000708812	0.006952716
VMO1	-5.378820596	4.82E-07	1.35E-05
CX3CR1	-5.380808783	0.000611191	0.006164142
RP11-384F7.2	-5.390184711	4.87E-07	1.36E-05
INSRR	-5.392527122	1.86E-05	0.000334281
RP5-961K14.1	-5.398024699	0.00191231	0.015469193
FAM178B	-5.398975936	0.008521292	0.049049655
RP11-90C4.1	-5.399759588	0.000142767	0.001883042
RP11-445H22.3	-5.407445552	0.00051493	0.005314578
KRT16	-5.409267811	0.000499298	0.00518171
A4GNT	-5.4150636	0.001244183	0.011006725
MYBPH	-5.417549635	0.000193024	0.002398539
ST8SIA5	-5.42207544	6.38E-05	0.0009519
RGMA	-5.426394696	3.07E-07	9.13E-06
RP11-120M18.5	-5.427472286	0.004060696	0.027773794

OR7E28P	-5.42799633	0.005741023	0.036259005
ZBTB7C	-5.437628471	0.00133427	0.011634728
DANT2	-5.441046008	1.80E-06	4.27E-05
ITGA10	-5.450443377	1.04E-25	4.18E-23
RP11-116O18.3	-5.452017966	5.35E-14	5.66E-12
GRIK4	-5.461454204	1.08E-05	0.000206439
RP11-259O2.1	-5.472661791	0.003305113	0.023733851
PARP4P1	-5.478225227	0.000348163	0.003877439
HEYL	-5.498080707	0.000232168	0.00277603
SLC14A1	-5.499811263	2.15E-17	3.53E-15
RP11-138I17.1	-5.501216718	2.89E-07	8.72E-06
UNC5C	-5.514945846	1.01E-12	8.62E-11
RP11-146E13.5	-5.525598017	0.002212211	0.017338476
MUC20P1	-5.527353628	0.000300317	0.003439755
DMBT1	-5.528023099	0.001616251	0.013557194
CACNA1C-AS1	-5.528385679	9.91E-06	0.000191359
LINC01482	-5.531168465	0.004130646	0.028169482
TAT	-5.531358368	0.007650029	0.045070386
LINC01614	-5.531755669	8.54E-42	9.62E-39
ENPP5	-5.533724347	9.55E-07	2.46E-05
CXADRP3	-5.551340092	0.00094925	0.00883445
LAPTM5	-5.559344919	2.69E-08	1.04E-06
LINC00619	-5.578172746	0.002578491	0.019576212
RP11-141C7.3	-5.586634786	0.001735556	0.014361661
LINC01750	-5.589782905	0.000164767	0.002109042
XXyac-YR38GF2.1	-5.597721748	7.76E-07	2.06E-05
RP11-13P5.2	-5.600051859	8.14E-06	0.000160752
ABCC12	-5.603135532	0.0015936	0.013408491
RTN4RL2	-5.607986931	5.82E-05	0.000885561
RP11-262H14.3	-5.618184894	1.47E-13	1.47E-11
GPR27	-5.631476214	0.000155268	0.002016073
SIX1	-5.632331506	1.00E-10	6.24E-09
KLHL13	-5.649308667	2.05E-27	9.04E-25
FRMD7	-5.654311979	3.44E-05	0.000565249
RN7SL526P	-5.658173097	0.000521885	0.005381432
COL6A4P2	-5.672155811	0.000475716	0.004980546
BMS1P22	-5.688108229	0.000579623	0.005906543
TMEM246	-5.697402495	0.000678868	0.006705677
LINC01436	-5.698367897	1.15E-06	2.86E-05
RP11-614F17.2	-5.723409552	0.000608984	0.006147382
PRR33	-5.724458216	1.07E-06	2.70E-05
LRRTM4	-5.728067652	0.006253401	0.038692813
C7	-5.752627299	0.001788454	0.0146648
RP11-343B18.2	-5.759763255	1.17E-05	0.000221661
PTH1R	-5.763315285	0.001548001	0.013098153
NPY2R	-5.78051465	0.005830969	0.036671163

ECEL1	-5.784282031	0.007263049	0.043311216
IGF1	-5.815025932	0.00020339	0.002503891
PPFIA4	-5.822881312	1.11E-15	1.45E-13
CYP1B1	-5.82494253	1.24E-18	2.29E-16
C20orf197	-5.826639288	0.002720751	0.020347862
MAN1C1	-5.830184225	7.05E-06	0.000141422
KCNMB1	-5.832999294	9.53E-08	3.20E-06
IGSF1	-5.835666539	0.000832465	0.007905208
C11orf87	-5.835766028	3.79E-06	8.23E-05
SERPINA9	-5.842642599	0.000373777	0.004091946
EFHD1	-5.844894475	1.07E-12	9.07E-11
CYP27C1	-5.858889189	9.71E-17	1.44E-14
RP11-196B3.2	-5.859506028	4.64E-05	0.000737088
PRDM16	-5.887978766	1.09E-12	9.19E-11
SYT13	-5.898028945	0.001659861	0.013836881
HCG22	-5.908169763	0.000195122	0.002416616
DUXAP9	-5.918151158	8.24E-13	7.20E-11
ALX1	-5.921590473	4.67E-16	6.22E-14
OLFM4	-5.922123541	0.00109288	0.009913322
TSHZ2	-5.93450031	2.84E-11	1.91E-09
CNTNAP3B	-5.942274516	2.52E-05	0.000434418
EVI2A	-5.944261972	4.79E-11	3.11E-09
RP11-18B16.2	-5.951628107	3.76E-05	0.000611409
CYGB	-5.952136482	3.71E-55	1.40E-51
RP11-111E14.1	-5.96221719	0.001267829	0.011176432
CSF2RA	-5.963933277	0.000349239	0.003885585
ANGPT4	-5.969077528	1.21E-07	3.98E-06
RP11-551L14.4	-5.978540594	0.000229981	0.002754522
SERPING1	-5.981480572	0.007690234	0.045271761
ATP1A2	-5.994686161	3.00E-05	0.000501915
RP11-34P13.14	-5.996447907	0.000279253	0.003239646
OSR2	-5.997800362	1.07E-14	1.24E-12
DAPK2	-6.00768588	2.46E-14	2.76E-12
CD24	-6.028912536	0.003219455	0.023229749
MACC1	-6.033205371	5.41E-05	0.000834603
LINC01444	-6.035118309	7.60E-06	0.000151508
METTL7B	-6.03783349	1.73E-10	1.02E-08
MKRN3-AS1	-6.03905551	0.000146487	0.001921985
ZMAT1	-6.047212199	2.06E-19	4.15E-17
RHOT1P1	-6.058541976	0.000215238	0.002618281
EBF3	-6.059604304	7.41E-05	0.001085814
RP11-121G22.3	-6.071279899	0.000906323	0.008512259
ITGB2	-6.073754958	1.91E-31	1.20E-28
SHISA9	-6.076575957	5.12E-05	0.000797945
NUTM2F	-6.115360323	0.000194999	0.002416427
REM1	-6.116224567	0.000287923	0.003326539

GLDN	-6.1232647	0.000215631	0.002621644
ZNF704	-6.151679067	1.23E-24	4.56E-22
CTLA4	-6.160596207	0.000394922	0.004285954
NRXN2	-6.162295278	3.59E-15	4.47E-13
RP11-93K22.1	-6.166986928	0.001470555	0.012602754
FGFR2	-6.186068851	2.28E-43	3.21E-40
CTC-535M15.2	-6.188319229	0.000225325	0.002712677
HR	-6.197332443	3.00E-05	0.000501915
RP11-735G4.1	-6.20034802	0.001071529	0.009770767
TRPC6	-6.200377969	5.07E-05	0.00079138
FAM19A5	-6.201460242	1.64E-05	0.00029931
AKR1C3	-6.21106127	8.93E-13	7.66E-11
ADGRF5P1	-6.221829953	6.40E-06	0.000129979
MAOB	-6.233334493	2.02E-05	0.000358394
ITGA7	-6.245573047	1.87E-13	1.79E-11
ACSM5	-6.248580073	0.001685885	0.014022703
AC011524.1	-6.254064028	0.000120966	0.001643911
CD74	-6.263273212	1.70E-41	1.83E-38
GAD1	-6.273611744	3.48E-05	0.000569735
TMEM176B	-6.283617393	0.005379061	0.034553836
RP5-1028K7.2	-6.303890397	8.36E-05	0.0012073
TRIM63	-6.325304995	0.002433829	0.018711098
UBE2QL1	-6.340672062	6.17E-07	1.69E-05
FGF10	-6.378541143	0.00014842	0.00194508
AGT	-6.387499737	8.28E-05	0.001198092
FAM225A	-6.396298694	1.97E-11	1.35E-09
COMP	-6.399748677	6.34E-08	2.21E-06
HCK	-6.41340845	0.000157052	0.002029882
PHOSPHO1	-6.415651475	4.87E-05	0.000769251
RP11-175K6.2	-6.420286972	4.75E-14	5.15E-12
BMS1P10	-6.42585545	2.28E-06	5.29E-05
GPR78	-6.428240827	0.000103984	0.001444995
NDP	-6.433398656	3.63E-05	0.00059265
AC006946.16	-6.437870297	2.79E-06	6.27E-05
AC073115.6	-6.446985127	3.50E-05	0.000572882
CACNA2D3	-6.447267917	4.91E-06	0.000102953
ZMAT4	-6.45620991	9.74E-05	0.001365522
FAM216B	-6.468854261	0.002072386	0.01648329
WISP3	-6.469087392	6.39E-09	2.80E-07
HLA-DRB6	-6.496084095	4.64E-05	0.000737176
MRAP2	-6.49716101	3.22E-11	2.15E-09
CYP4X1	-6.533864732	0.000622505	0.006253071
TSPAN11	-6.538707541	2.54E-23	7.74E-21
FMO3	-6.547013101	3.78E-05	0.000613244
RUNX3	-6.557945165	5.05E-12	3.74E-10
IGFBP2	-6.566526126	1.46E-31	9.39E-29

LINC01876	-6.579608098	2.04E-05	0.000361459
ECM2	-6.595710938	8.78E-29	4.40E-26
DDO	-6.607093776	6.63E-08	2.31E-06
CTD-2201I18.1	-6.643771166	1.86E-05	0.000333399
SATB2-AS1	-6.656832529	6.91E-10	3.73E-08
FSHR	-6.66598602	9.17E-06	0.000178651
CHI3L1	-6.694148804	9.53E-08	3.20E-06
POU3F3	-6.728099249	8.73E-05	0.001248943
RP11-1134I14.8	-6.731553632	6.64E-06	0.000134667
PRG4	-6.763665022	1.04E-12	8.85E-11
FOLR1	-6.782774165	0.000801737	0.007673869
BEX5	-6.784447567	1.60E-05	0.000292713
SLC8A3	-6.788729654	1.48E-05	0.000274211
CHDH	-6.812979552	3.93E-07	1.14E-05
RORB	-6.815734133	3.13E-07	9.25E-06
AC008991.1	-6.822504989	0.000150439	0.001964691
DUSP15	-6.827194758	6.05E-06	0.000123667
DLX1	-6.833317847	1.77E-13	1.71E-11
INSC	-6.863141842	5.25E-05	0.000812955
RP11-93K22.6	-6.869967957	7.94E-06	0.000157622
ST8SIA1	-6.891164995	3.19E-34	2.76E-31
RBP4	-6.899917303	4.64E-12	3.46E-10
CPB1	-6.907746678	4.76E-05	0.000753511
BEND5	-6.918085285	7.27E-05	0.001067248
SFRP2	-6.93110694	3.12E-06	6.93E-05
RHOXF1-AS1	-6.936246649	1.43E-07	4.64E-06
SNED1	-6.943210914	4.38E-42	5.19E-39
NPR3	-6.970668665	7.85E-23	2.16E-20
AP001092.4	-6.974698937	1.01E-07	3.37E-06
RUBCNL	-6.979102745	3.16E-11	2.12E-09
FCGR2A	-7.061156865	3.15E-06	6.99E-05
OR7D2	-7.066642527	5.94E-07	1.63E-05
BARX1	-7.067063395	3.70E-06	8.05E-05
RP11-326A19.4	-7.080933196	1.97E-06	4.63E-05
SYT12	-7.093824238	1.66E-11	1.15E-09
ZNF727	-7.103053416	1.04E-06	2.64E-05
LINC01550	-7.103752763	5.47E-06	0.000113089
SMOC2	-7.128079624	3.74E-12	2.85E-10
SCUBE1	-7.144169278	6.69E-05	0.000990422
EYA4	-7.149235609	1.06E-17	1.81E-15
TP63	-7.161551353	1.80E-07	5.72E-06
RCAN2	-7.192861347	6.95E-29	3.56E-26
HNF4G	-7.195050603	3.19E-08	1.21E-06
LINC01139	-7.214116529	5.08E-06	0.000105791
IGF2	-7.247210852	3.89E-44	6.27E-41
PREX2	-7.286634932	1.61E-07	5.16E-06

RP11-429A20.3	-7.286927238	3.33E-05	0.000550274
DCDC1	-7.321411407	2.84E-09	1.35E-07
MMP13	-7.32396579	1.81E-05	0.0003266
FNDC1	-7.333535311	1.36E-22	3.66E-20
ISM1	-7.340303071	1.70E-17	2.82E-15
LSP1	-7.347068116	1.99E-15	2.51E-13
HLA-DRB5	-7.349646815	1.08E-06	2.71E-05
ALPL	-7.35066401	4.84E-23	1.36E-20
SEMA3F	-7.354684591	4.31E-23	1.23E-20
CLEC3B	-7.39743588	1.11E-19	2.33E-17
PCDHB4	-7.408399445	8.20E-08	2.79E-06
CHL1	-7.440305729	7.05E-07	1.90E-05
SIX2	-7.470541835	3.40E-22	8.81E-20
ITGA8	-7.479701917	1.11E-08	4.64E-07
CDH5	-7.504725238	1.48E-06	3.58E-05
A2M	-7.516594758	2.61E-17	4.24E-15
STEAP4	-7.613130897	5.77E-17	8.73E-15
SMG1P4	-7.63469509	0.001805501	0.014772338
FLG	-7.63818263	3.31E-17	5.21E-15
RPTN	-7.639421204	1.97E-05	0.000350459
SNAP25	-7.686172001	5.58E-07	1.54E-05
ZIC4	-7.695181323	4.89E-09	2.21E-07
AC226119.4	-7.696646918	0.000335539	0.00376101
FAM84A	-7.719887211	3.73E-37	3.83E-34
MEOX2	-7.738454139	1.35E-07	4.40E-06
HLA-DMB	-7.759876841	1.85E-09	9.11E-08
OMD	-7.767541053	9.84E-09	4.17E-07
NLGN4X	-7.776296495	3.14E-12	2.45E-10
ZNF204P	-7.80494636	4.27E-08	1.56E-06
LINC01443	-7.80621085	3.59E-09	1.66E-07
SELENOP	-7.813506028	3.06E-17	4.86E-15
RP11-1102P16.1	-7.816033177	3.63E-09	1.68E-07
RPL23AP87	-7.849431461	1.08E-09	5.55E-08
MKRN3	-7.876925718	2.90E-07	8.74E-06
NTRK2	-7.891694895	7.52E-37	6.78E-34
SPATA20P1	-7.901771174	1.08E-07	3.55E-06
GIMAP2	-7.927062566	1.66E-07	5.29E-06
ACAN	-8.017830034	4.76E-20	1.06E-17
ELN	-8.048201585	3.60E-19	7.00E-17
DLX3	-8.092762725	5.73E-10	3.17E-08
TSPAN18	-8.129218596	9.25E-32	6.13E-29
HCAR1	-8.161991094	5.53E-08	1.96E-06
RP11-314N14.1	-8.18265481	2.15E-09	1.05E-07
CPN2	-8.186892916	2.73E-09	1.30E-07
LEP	-8.194117182	4.35E-09	1.98E-07
ZIC1	-8.212316754	1.10E-10	6.77E-09

HLA-DOA	-8.258021054	5.96E-09	2.64E-07
KIAA0040	-8.31570082	1.17E-07	3.85E-06
TMEM30B	-8.324618213	1.15E-08	4.76E-07
LINC02202	-8.342117724	1.65E-10	9.76E-09
TM4SF20	-8.377535974	1.63E-13	1.60E-11
PLEKHS1	-8.408016152	6.15E-11	3.94E-09
LGR5	-8.416225216	0.000367125	0.004038731
APCDD1	-8.443529717	5.58E-09	2.49E-07
WFDC1	-8.446495016	1.07E-06	2.69E-05
C5AR2	-8.487135374	1.45E-10	8.74E-09
CSF2RB	-8.499801051	0.000389622	0.004240688
OR7E22P	-8.544298448	0.00017981	0.002264298
AP001434.2	-8.594452431	8.63E-10	4.57E-08
FAM20A	-8.616600049	6.69E-21	1.59E-18
ITIH5	-8.675506007	3.55E-08	1.32E-06
EYA1	-8.689984236	3.91E-19	7.53E-17
EYA2	-8.709779279	5.45E-34	4.39E-31
CCKAR	-8.73440752	1.29E-05	0.000241779
CTNND2	-8.759413507	0.002498617	0.019091971
RIMS4	-8.819281012	9.57E-09	4.08E-07
DLX6	-8.94468096	3.25E-12	2.52E-10
IBSP	-8.996442577	2.17E-10	1.27E-08
WISP2	-9.048995864	1.35E-11	9.50E-10
DLX5	-9.068974918	8.80E-13	7.57E-11
AP000350.5	-9.108064828	4.62E-05	0.000734718
PSG4	-9.125140496	3.82E-09	1.76E-07
DLX6-AS1	-9.198715328	1.44E-12	1.20E-10
TRH	-9.20065526	1.12E-08	4.68E-07
BRINP1	-9.302998817	3.33E-14	3.65E-12
KCNA1	-9.446351681	3.97E-09	1.81E-07
S100B	-9.548621178	3.14E-13	2.93E-11
SHOX	-9.874333192	4.91E-12	3.64E-10
PENK	-9.935094101	7.18E-10	3.85E-08
CMKLR1	-9.949929263	1.65E-08	6.61E-07
LBP	-10.19285743	4.29E-07	1.23E-05
RTN4RL1	-10.26652798	1.75E-13	1.70E-11
HLA-DRB1	-10.65503825	1.08E-17	1.83E-15
LINC01423	-11.09948979	2.58E-16	3.59E-14
HLA-DRA	-11.72825537	2.26E-21	5.59E-19
SMOC1	-13.5134372	2.12E-26	8.84E-24
