# Supplementary material for: Adipose tissue-derived human mesenchymal stromal cells can better suppress complement lysis, engraft and inhibit acute graft-versus-host disease in mice
Source: Stem Cell Res Ther. 2023 Jun 25;14:167. doi: 10.1186/s13287-023-03380-x (PMC10291819; doi:10.1186/s13287-023-03380-x)
Supplement: Supplementary file 11 — Additional file 11: Table S6. List of Differentially expressed genes between AT-hMSCs and UC-hMSCs. Log2 FoldChange > 1 or < − 1, p-value < 0.05. [file 13287_2023_3380_MOESM11_ESM.pdf]

**Table S6. List of Differentially expressed genes between AT-hMSCs and UC-hMSCs.** Log2 FoldChange >1 or <-1, p-value <0.05

| Gene name | log2FoldChange | pvalue | padj |
|---|---|---|---|
| GSTM1 | 10.74898141 | 1.48E-18 | 6.74E-16 |
| COL15A1 | 10.34052958 | 4.35E-28 | 4.15E-25 |
| SFRP2 | 9.344205724 | 2.60E-10 | 2.86E-08 |
| XPNPEP2 | 8.597065478 | 0.000260173 | 0.00532311 |
| CMKLR1 | 8.352828079 | 2.18E-06 | 9.00E-05 |
| DPT | 8.314580448 | 3.82E-16 | 1.23E-13 |
| RP11-92C4.6 | 8.120831027 | 0.02113621 | 0.14814255 |
| AP000350.5 | 8.112884204 | 0.000287935 | 0.005726857 |
| TSHZ2 | 7.867748638 | 9.24E-19 | 4.61E-16 |
| SHISA9 | 7.831164963 | 1.41E-07 | 8.08E-06 |
| PSG4 | 7.793707715 | 4.90E-07 | 2.37E-05 |
| TMEM30B | 7.4584328 | 3.43E-07 | 1.75E-05 |
| RNF165 | 7.211326355 | 3.67E-05 | 0.001023947 |
| AC006946.16 | 7.202265499 | 1.43E-07 | 8.13E-06 |
| KANK4 | 7.14052305 | 1.52E-07 | 8.58E-06 |
| TRIML2 | 7.109699486 | 3.46E-08 | 2.32E-06 |
| GDF10 | 6.927347951 | 3.87E-06 | 0.000149782 |
| PCDHB4 | 6.826577962 | 8.60E-07 | 3.97E-05 |
| SLC2A14 | 6.78980177 | 3.75E-05 | 0.00104181 |
| LCE2A | 6.74844792 | 0.000261628 | 0.005334191 |
| SMG1P4 | 6.737441644 | 0.005981619 | 0.061205573 |
| NPR1 | 6.522067956 | 3.94E-06 | 0.000151801 |
| KCNA1 | 6.46845997 | 6.43E-05 | 0.001653824 |
| COMP | 6.417229908 | 5.84E-08 | 3.64E-06 |
| TBX5 | 6.381578135 | 4.18E-16 | 1.33E-13 |
| PTGDR | 6.315979107 | 5.52E-05 | 0.001463641 |
| OMD | 6.289187464 | 4.33E-06 | 0.000164019 |
| SLCO4C1 | 6.289045792 | 0.010312481 | 0.090711789 |
| FAM20A | 6.260409417 | 9.90E-12 | 1.43E-09 |
| DUXAP10 | 6.236890037 | 2.95E-08 | 2.02E-06 |
| CCL11 | 6.230247389 | 0.004465732 | 0.04893568 |
| ZFP42 | 6.201610857 | 0.000596786 | 0.010204432 |
| CHMP1B2P | 6.107056501 | 0.00021243 | 0.004485355 |
| LINC01750 | 6.078209245 | 4.06E-05 | 0.00111547 |
| FNDC1 | 6.05465374 | 6.85E-16 | 2.06E-13 |
| LINC02202 | 6.036457507 | 4.50E-06 | 0.000169958 |
| UBE2QL1 | 6.036253822 | 2.22E-06 | 9.12E-05 |
| TBX5-AS1 | 6.007148144 | 2.21E-15 | 5.92E-13 |
| LINC01436 | 5.995187705 | 3.07E-07 | 1.60E-05 |
| AR | 5.990282425 | 0.0002879 | 0.005726857 |
| RP11-656G20.1 | 5.946393268 | 0.002133389 | 0.027563189 |
| C5AR2 | 5.930739508 | 8.97E-06 | 0.000310305 |
| TMEM119 | 5.930340028 | 2.56E-20 | 1.48E-17 |

| | | | |
|---|---|---|---|
| CECR7 | 5.914628178 | 1.47E-05 | 0.000474462 |
| AP001434.2 | 5.91426521 | 3.25E-05 | 0.000927009 |
| UNC13A | 5.868563221 | 1.61E-09 | 1.51E-07 |
| RP11-146E13.5 | 5.859171345 | 0.001159363 | 0.017196826 |
| POU3F3 | 5.856411457 | 0.000692538 | 0.011498853 |
| ISLR2 | 5.85276519 | 1.23E-06 | 5.44E-05 |
| RARRES2 | 5.847967054 | 3.64E-05 | 0.001017419 |
| RP11-255I10.1 | 5.828515897 | 0.023997456 | 0.161898262 |
| ELN | 5.810438205 | 1.05E-10 | 1.26E-08 |
| TLX2 | 5.793031434 | 0.000310648 | 0.00607453 |
| TRH | 5.783184489 | 0.000398823 | 0.007377219 |
| RP11-401O9.3 | 5.777593695 | 0.0011051 | 0.016593898 |
| PRKG2 | 5.776672511 | 0.003437174 | 0.039938948 |
| KIAA0040 | 5.761042294 | 0.0002676 | 0.005430714 |
| ADGRF5P1 | 5.756150945 | 3.42E-05 | 0.000964978 |
| AGT | 5.749740391 | 0.00039734 | 0.007355984 |
| EDNRB | 5.748150536 | 1.09E-09 | 1.08E-07 |
| MYH13 | 5.741736209 | 0.00165077 | 0.022577682 |
| A2M | 5.699624028 | 1.45E-10 | 1.71E-08 |
| HCAR1 | 5.678417461 | 0.000197954 | 0.00421623 |
| ITIH5 | 5.675276139 | 0.000322622 | 0.006246856 |
| LINC01197 | 5.65376712 | 0.004605239 | 0.050052091 |
| FAM225A | 5.649570422 | 3.48E-09 | 2.94E-07 |
| ZIC1 | 5.631627774 | 1.51E-05 | 0.000484646 |
| ZNF204P | 5.627023007 | 0.000103041 | 0.002465117 |
| RTN4RL1 | 5.624521764 | 7.79E-05 | 0.001944439 |
| HADHAP2 | 5.607843816 | 0.00056249 | 0.009708818 |
| SIM1 | 5.603636054 | 5.38E-08 | 3.42E-06 |
| SLC13A5 | 5.576016033 | 0.037012475 | 0.213285967 |
| HEYL | 5.56473879 | 0.000197254 | 0.004205401 |
| PTGS1 | 5.513133559 | 8.16E-39 | 1.79E-35 |
| RP11-401O9.4 | 5.48500251 | 0.000235131 | 0.004880129 |
| RP11-34P13.14 | 5.479384357 | 0.000974852 | 0.015113988 |
| CD70 | 5.46596252 | 0.000130599 | 0.0030127 |
| DUXAP9 | 5.428526789 | 6.14E-11 | 7.65E-09 |
| FIRRE | 5.425952366 | 0.000307668 | 0.006026452 |
| LINC01684 | 5.418639867 | 3.55E-06 | 0.000138916 |
| RIMS3 | 5.390017032 | 4.03E-05 | 0.001108671 |
| RP11-195E2.1 | 5.382483511 | 0.002460358 | 0.030650384 |
| HR | 5.367762547 | 0.000327234 | 0.006313854 |
| TNXB | 5.364768137 | 3.92E-17 | 1.46E-14 |
| NUTM2F | 5.35280799 | 0.001236957 | 0.018066825 |
| MMP9 | 5.338580889 | 0.018004729 | 0.133803437 |
| LA16c-60H5.7 | 5.326571286 | 0.001522107 | 0.021271171 |
| MYH2 | 5.318557879 | 0.00141914 | 0.020096673 |
| DDO | 5.316018576 | 1.57E-05 | 0.000502361 |

| | | | |
|---|---|---|---|
| SPATA20P1 | 5.310523864 | 0.00041133 | 0.007563917 |
| IRX1 | 5.303004913 | 1.46E-07 | 8.29E-06 |
| APBB1IP | 5.300275911 | 0.001032896 | 0.01579071 |
| LGI2 | 5.299784514 | 0.000273921 | 0.005523242 |
| TCL6 | 5.287912765 | 0.010292097 | 0.090605146 |
| EGR2 | 5.258515107 | 8.69E-09 | 6.64E-07 |
| DCLK3 | 5.245235784 | 5.42E-07 | 2.59E-05 |
| SATB2-AS1 | 5.239366045 | 1.50E-06 | 6.45E-05 |
| RP11-262H14.3 | 5.237650445 | 6.68E-12 | 9.96E-10 |
| RP11-278A23.2 | 5.235198364 | 0.001569141 | 0.021691121 |
| RP11-3L21.2 | 5.232549356 | 0.000615153 | 0.010493961 |
| L1TD1 | 5.226743263 | 0.026285182 | 0.17070584 |
| CDH5 | 5.222498624 | 0.001015537 | 0.015590525 |
| KCNH1 | 5.186937155 | 0.000761304 | 0.012371475 |
| ANKRD33 | 5.164454858 | 0.039057694 | 0.220798401 |
| COL6A6 | 5.152967798 | 0.001843449 | 0.024674542 |
| XXbac-BPG55C20.7 | 5.149508063 | 0.003792684 | 0.043188346 |
| BMS1P10 | 5.137300248 | 0.000214144 | 0.004512856 |
| UNC93B3 | 5.128832332 | 0.008298589 | 0.077371206 |
| TRIM58 | 5.126365682 | 6.37E-06 | 0.000229727 |
| TBX15 | 5.120270298 | 0.00362951 | 0.041731761 |
| PLA2G5 | 5.107854308 | 0.000224605 | 0.004701711 |
| CH25H | 5.105776949 | 8.77E-06 | 0.000304466 |
| FOXF2 | 5.101120123 | 4.70E-09 | 3.89E-07 |
| LL22NC03-N64E9.1 | 5.097396461 | 0.004965425 | 0.052930757 |
| PSORS1C3 | 5.092058785 | 0.009605402 | NA |
| ADGRE2 | 5.082488209 | 0.000760188 | 0.012362491 |
| IL21R | 5.072566135 | 9.52E-08 | 5.60E-06 |
| GIMAP2 | 5.070539336 | 0.001081582 | 0.016352678 |
| KLHL30 | 5.059335337 | 3.36E-06 | 0.000132144 |
| LINC00908 | 5.045961086 | 0.002629493 | 0.032233286 |
| CACNA1C-AS2 | 5.02791654 | 0.001181121 | 0.017425301 |
| SERPINF1 | 5.025690695 | 1.43E-08 | 1.05E-06 |
| AC008991.1 | 5.020081628 | 0.006018122 | 0.061521698 |
| TAC1 | 5.018695804 | 0.003062801 | 0.036261055 |
| RP11-626H12.2 | 5.009656807 | 0.01518702 | 0.119717154 |
| MAB21L1 | 4.97762209 | 0.002478956 | 0.030794641 |
| C5orf38 | 4.974959421 | 0.001742193 | 0.023578174 |
| PRG4 | 4.964918719 | 1.97E-07 | 1.07E-05 |
| TRPC6 | 4.962120119 | 0.001315817 | 0.018953644 |
| RP11-533E19.5 | 4.953289929 | 0.001215003 | 0.017799267 |
| WISP2 | 4.94614177 | 0.000223044 | 0.004673478 |
| SIX1 | 4.931303878 | 1.54E-08 | 1.13E-06 |
| CRLF1 | 4.92157571 | 2.70E-14 | 6.43E-12 |
| ACADL | 4.918820348 | 0.000945554 | 0.014722189 |
| EBF3 | 4.916174713 | 0.001306766 | 0.01888526 |

| | | | |
|---|---|---|---|
| C3orf80 | 4.894957968 | 1.75E-10 | 2.02E-08 |
| HSFX2 | 4.886325781 | 0.00752227 | 0.072478249 |
| IGF1 | 4.8618882 | 0.002246144 | 0.028516152 |
| TUNAR | 4.85953374 | 0.000316866 | 0.006168523 |
| FCGR2A | 4.832602889 | 0.001887549 | 0.025126851 |
| VIT | 4.829501915 | 3.56E-11 | 4.62E-09 |
| C8B | 4.828473478 | 0.041795355 | NA |
| G0S2 | 4.826895654 | 2.91E-09 | 2.49E-07 |
| GPR65 | 4.821818344 | 0.010926459 | NA |
| DMKN | 4.819181554 | 0.000131765 | 0.003036399 |
| PDE1A | 4.797257378 | 1.68E-10 | 1.96E-08 |
| AC226119.4 | 4.780734644 | 0.026565634 | 0.171916486 |
| RP11-661A12.12 | 4.779942405 | 0.003550382 | NA |
| RP11-141C7.3 | 4.763373168 | 0.008356019 | 0.077642381 |
| RP11-213G6.2 | 4.756597489 | 0.017503133 | 0.131366311 |
| APCDD1 | 4.752361927 | 0.00152514 | 0.021271171 |
| NINJ2 | 4.749969995 | 2.05E-09 | 1.83E-07 |
| CTD-2201I18.1 | 4.730475878 | 0.002955098 | 0.03525228 |
| COL10A1 | 4.723697697 | 4.10E-08 | 2.69E-06 |
| SVILP1 | 4.714320197 | 2.41E-07 | 1.29E-05 |
| RGMA | 4.709925616 | 9.21E-06 | 0.000318017 |
| CACNA2D3 | 4.6706126 | 0.001327932 | 0.019078046 |
| SYT17 | 4.662258555 | 0.008394971 | 0.077905618 |
| CES1 | 4.658734683 | 3.17E-06 | 0.000125592 |
| APOD | 4.650120776 | 1.90E-05 | 0.000590514 |
| AKR1C3 | 4.632706287 | 1.01E-07 | 5.93E-06 |
| ERG | 4.630987041 | 1.12E-08 | 8.42E-07 |
| RCAN2 | 4.62727895 | 9.72E-13 | 1.76E-10 |
| HNF4G | 4.625812331 | 0.000649234 | 0.010930845 |
| RP11-356C4.5 | 4.624793889 | 4.39E-05 | 0.001199004 |
| SSC5D | 4.622806079 | 6.72E-18 | 2.84E-15 |
| RASSF9 | 4.615653261 | 1.24E-24 | 8.77E-22 |
| ACKR3 | 4.603923487 | 5.69E-12 | 8.55E-10 |
| RSPO4 | 4.593263245 | 0.000179402 | 0.00393573 |
| CTB-12A17.2 | 4.576113397 | 0.000558104 | 0.009663524 |
| PODN | 4.571253005 | 1.24E-11 | 1.75E-09 |
| SFRP1 | 4.569169952 | 0.000299593 | 0.005910467 |
| HEPH | 4.558176217 | 0.000392713 | 0.007279608 |
| CTD-2293H3.2 | 4.557954888 | 0.024020481 | 0.16191293 |
| CACNA1C-AS1 | 4.557043151 | 0.000304877 | 0.005987607 |
| SMN2 | 4.548726884 | 7.08E-16 | 2.10E-13 |
| SLCO2A1 | 4.548653172 | 0.007102709 | NA |
| C8orf4 | 4.548331998 | 0.002041939 | 0.026600984 |
| RP11-445H22.3 | 4.545976493 | 0.003848458 | 0.043699521 |
| BMS1P22 | 4.544273027 | 0.007050394 | 0.0695153 |
| C21orf33 | 4.510523184 | 1.72E-05 | 0.000545358 |

| | | | |
|---|---|---|---|
| TNNT3 | 4.489674513 | 0.049504151 | 0.256083616 |
| GLDN | 4.484205896 | 0.008208316 | 0.076750699 |
| MMP8 | 4.461215266 | 1.24E-05 | 0.000407648 |
| ISLR | 4.445483883 | 4.11E-58 | 2.25E-54 |
| ZNF826P | 4.437574737 | 6.46E-18 | 2.78E-15 |
| HOXD1 | 4.436221227 | 0.004418925 | 0.048568322 |
| TP63 | 4.430170915 | 0.002004055 | 0.026185206 |
| FAM157C | 4.413966617 | 6.96E-05 | 0.001774424 |
| CST1 | 4.413141973 | 0.007767737 | 0.073897928 |
| SIX2 | 4.412558473 | 1.42E-08 | 1.05E-06 |
| RIMS4 | 4.408529136 | 0.005836148 | 0.059940734 |
| DLX1 | 4.406795504 | 3.08E-06 | 0.000122405 |
| EYA1 | 4.406744562 | 8.61E-06 | 0.000299191 |
| GPAT2P1 | 4.399393938 | 0.003212355 | 0.037714856 |
| ZNF704 | 4.392701958 | 3.62E-13 | 7.15E-11 |
| RP11-575H3.1 | 4.390902995 | 0.002889492 | 0.034696043 |
| RP11-6E9.4 | 4.383081836 | 0.040927031 | NA |
| FAM87A | 4.382507306 | 0.000707958 | 0.011721646 |
| ITM2A | 4.367357775 | 0.001099026 | 0.016548 |
| HAGLR | 4.34475983 | 7.33E-08 | 4.43E-06 |
| ADAMTSL2 | 4.34101034 | 0.004897279 | 0.052356969 |
| SHOX2 | 4.340220331 | 0.00780274 | 0.074006276 |
| RNF182 | 4.339851416 | 5.42E-06 | 0.000199334 |
| RASA4 | 4.333129262 | 2.14E-11 | 2.92E-09 |
| CTD-2012J19.2 | 4.329026543 | 0.007112801 | 0.069879368 |
| EMX2 | 4.318104373 | 0.005075662 | 0.053795236 |
| ZMAT4 | 4.313501243 | 0.010404141 | 0.091117337 |
| SLC1A7 | 4.307814563 | 5.02E-08 | 3.24E-06 |
| RP11-426C22.1 | 4.299132005 | 0.014765147 | NA |
| ADRA2A | 4.296788352 | 2.39E-05 | 0.000720279 |
| RP11-387A1.5 | 4.291786607 | 0.005839904 | 0.059951244 |
| SCN7A | 4.29175024 | 0.03166569 | 0.192618676 |
| KRT14 | 4.291170734 | 0.012429323 | 0.103875994 |
| MFAP4 | 4.285798775 | 1.80E-09 | 1.64E-07 |
| ALX1 | 4.27405824 | 6.53E-09 | 5.17E-07 |
| SOX18 | 4.270149075 | 0.035334906 | NA |
| PALM | 4.265477452 | 0.001238679 | 0.018067908 |
| INHBE | 4.263126553 | 3.04E-07 | 1.59E-05 |
| AC139099.4 | 4.262592597 | 0.029475345 | NA |
| LINC01655 | 4.261293073 | 0.010162177 | 0.08970688 |
| HAS1 | 4.260996998 | 6.20E-09 | 4.95E-07 |
| RP11-212D19.5 | 4.255575652 | 0.008014453 | 0.075394967 |
| HTR1F | 4.248240225 | 0.001933527 | 0.025537462 |
| PENK | 4.24600871 | 0.008490535 | 0.078592976 |
| AC092535.3 | 4.244269854 | 0.008164721 | 0.076503287 |
| FOXS1 | 4.244159126 | 0.00055013 | 0.009548071 |

| | | | |
|---|---|---|---|
| PCSK9 | 4.242522635 | 0.001646885 | 0.022552663 |
| KRT7 | 4.236069575 | 1.70E-06 | 7.17E-05 |
| RP11-671E7.1 | 4.233504952 | 0.004614086 | 0.050086005 |
| NGEF | 4.230845398 | 1.05E-06 | 4.70E-05 |
| DUXAP7 | 4.228664406 | 0.034327799 | NA |
| PRDM16 | 4.225628211 | 3.47E-07 | 1.76E-05 |
| LINC01956 | 4.221036727 | 0.000290377 | 0.005759749 |
| TLE2 | 4.214884801 | 7.64E-07 | 3.56E-05 |
| STAC | 4.211992113 | 0.002905177 | 0.034808183 |
| RP11-495P10.5 | 4.203200492 | 0.009321226 | 0.08418652 |
| PTH1R | 4.201567005 | 0.024309986 | 0.162889399 |
| IGDCC4 | 4.196516417 | 9.13E-10 | 9.23E-08 |
| RP11-84D1.1 | 4.193895429 | 0.047382411 | NA |
| HSPD1P6 | 4.189429478 | 0.001974513 | 0.025876271 |
| DANT2 | 4.163781967 | 0.000323011 | 0.006248858 |
| MEOX2 | 4.162687113 | 0.004654752 | 0.050477483 |
| ECM2 | 4.161279748 | 2.58E-12 | 4.23E-10 |
| CD22 | 4.160428113 | 0.026147083 | 0.170161586 |
| RP11-346C16.4 | 4.159271874 | 0.041933862 | NA |
| FAM225B | 4.150275651 | 1.48E-06 | 6.38E-05 |
| RP11-93K22.6 | 4.144733134 | 0.009824347 | 0.087505698 |
| AMD1P4 | 4.1421361 | 0.0216996 | NA |
| PINLYP | 4.141904263 | 4.36E-07 | 2.15E-05 |
| WISP1 | 4.137120832 | 6.73E-10 | 6.99E-08 |
| C16orf47 | 4.130459599 | 0.012887515 | 0.106288086 |
| EMX2OS | 4.129385974 | 0.034060203 | 0.201318083 |
| NRN1 | 4.124006037 | 0.005743027 | 0.05920607 |
| RP11-175K6.2 | 4.116443861 | 2.10E-06 | 8.70E-05 |
| MACC1 | 4.115596563 | 0.008204173 | 0.076750699 |
| CYGB | 4.114014893 | 4.49E-27 | 3.64E-24 |
| PTGDS | 4.107015287 | 0.046547142 | 0.247845913 |
| RP11-60A14.1 | 4.100866187 | 0.025512169 | NA |
| RP11-415J8.7 | 4.093690887 | 0.010468009 | 0.091565727 |
| LEF1 | 4.086527065 | 0.001251751 | 0.018222224 |
| OSR2 | 4.085135025 | 1.92E-07 | 1.05E-05 |
| RP11-1134I14.8 | 4.084639019 | 0.009079111 | 0.082447835 |
| OTX1 | 4.079929211 | 0.019249709 | NA |
| MIR4664 | 4.078567981 | 0.009264035 | 0.083790658 |
| TUBBP6 | 4.0677126 | 0.029567364 | 0.183961667 |
| PCDHA11 | 4.064539978 | 0.023724705 | NA |
| ABCA9-AS1 | 4.06387929 | 0.002314582 | 0.029215932 |
| DUXAP8 | 4.055450359 | 0.025895377 | 0.169276751 |
| F2RL3 | 4.050377966 | 0.016974339 | 0.128718648 |
| IL20RB | 4.046861311 | 8.91E-12 | 1.30E-09 |
| TRIL | 4.041618751 | 1.97E-05 | 0.000607169 |
| PARP4P1 | 4.041251696 | 0.010943236 | 0.094890399 |

| | | | |
|---|---|---|---|
| ASPN | 4.033645056 | 5.95E-13 | 1.13E-10 |
| BEX5 | 4.031406714 | 0.014189865 | 0.114063676 |
| MKRN3 | 4.030928963 | 0.012516711 | 0.104478321 |
| AP000473.8 | 4.028384516 | 0.014403192 | 0.115278084 |
| CYP1B1 | 4.026989881 | 1.12E-09 | 1.10E-07 |
| TPTEP1 | 4.024825729 | 0.007937564 | 0.074767831 |
| LINC01060 | 4.020823254 | 0.000272538 | 0.005510537 |
| AC007618.3 | 4.010008381 | 0.038296664 | 0.218164689 |
| LINC01423 | 4.009425195 | 0.005665195 | 0.058623691 |
| ADRA2C | 4.008753028 | 0.0074223 | 0.071758753 |
| RUNDC3B | 4.006447639 | 0.001907104 | 0.025279793 |
| FNDC1-IT1 | 4.005194898 | 0.014528242 | 0.115856263 |
| MYO1D | 4.004885755 | 2.83E-10 | 3.10E-08 |
| EYA2 | 4.001885322 | 4.84E-08 | 3.14E-06 |
| RP6-74O6.6 | 3.995080055 | 0.041665746 | 0.230707503 |
| ANKRD65 | 3.982591553 | 0.000101442 | 0.002433698 |
| SYT7 | 3.982235666 | 7.04E-06 | 0.000251142 |
| GPAT2 | 3.978391088 | 5.19E-10 | 5.53E-08 |
| GS1-57L11.1 | 3.977971402 | 0.001661234 | 0.022706634 |
| BHMT | 3.977200629 | 0.001893341 | 0.025173399 |
| MUC20P1 | 3.977057262 | 0.010839607 | 0.094247623 |
| C11orf96 | 3.971877764 | 0.004821835 | 0.051699536 |
| PDE1C | 3.963847325 | 0.001873744 | 0.025018994 |
| RP11-236F9.2 | 3.9623358 | 0.048881025 | NA |
| C4B | 3.960798634 | 0.000722002 | 0.011891349 |
| CECR2 | 3.960052944 | 5.29E-06 | 0.000195814 |
| AC073621.2 | 3.944717514 | 0.012025402 | 0.101505679 |
| MMP13 | 3.937732141 | 0.024194307 | 0.162577469 |
| RP11-379H8.1 | 3.935902298 | 0.010404272 | 0.091117337 |
| ANKRD45 | 3.935001218 | 0.025197969 | 0.166053786 |
| CDX1 | 3.927017067 | 0.013632572 | 0.110849281 |
| RP11-469A15.2 | 3.92409751 | 1.12E-05 | 0.000374296 |
| SRP68P2 | 3.923783188 | 0.019869639 | 0.142964955 |
| LL22NC03-N14H11.1 | 3.922555384 | 0.009781062 | 0.087219139 |
| SEMA3F | 3.918476249 | 1.87E-07 | 1.03E-05 |
| RP11-138I17.1 | 3.90950822 | 0.000334802 | 0.006422206 |
| ABCA6 | 3.903685186 | 1.53E-07 | 8.65E-06 |
| ACAN | 3.899453045 | 8.28E-06 | 0.000289376 |
| RP11-680G24.4 | 3.898296416 | 0.001750532 | 0.023661844 |
| RP5-1054A22.4 | 3.89706033 | 0.028887051 | 0.181323068 |
| LINC01482 | 3.896281591 | 0.049312631 | 0.255778566 |
| GRID1 | 3.896253219 | 0.000797408 | 0.01279703 |
| RP11-466A19.4 | 3.892282084 | 0.019975638 | 0.143506087 |
| TMEM246 | 3.889861626 | 0.020411048 | 0.1451467 |
| FAM162B | 3.886317521 | 3.13E-05 | 0.000901752 |
| RP11-1430O6.1 | 3.87115496 | 2.46E-05 | 0.000737633 |

| | | | |
|---|---|---|---|
| TCAM1P | 3.86887643 | 0.043368017 | NA |
| RP11-234G16.5 | 3.868845219 | 0.043420199 | 0.236685496 |
| SALRNA1 | 3.867671608 | 0.045351527 | 0.2431977 |
| KRT81 | 3.865344208 | 0.00146974 | 0.020668692 |
| CACNA1H | 3.864441748 | 0.017867292 | 0.133013091 |
| PPARG | 3.864014553 | 6.57E-06 | 0.000236785 |
| RP11-11N5.1 | 3.86243036 | 0.036695218 | NA |
| CSTA | 3.857215953 | 3.53E-08 | 2.35E-06 |
| TRABD2B | 3.848731435 | 3.74E-05 | 0.001040608 |
| PRRX2 | 3.847492191 | 8.76E-10 | 8.93E-08 |
| LINC01140 | 3.83375313 | 2.43E-06 | 9.89E-05 |
| THBD | 3.82111888 | 1.43E-07 | 8.15E-06 |
| FRG1BP | 3.813084857 | 4.67E-12 | 7.17E-10 |
| ZIC4 | 3.808410595 | 0.007313146 | 0.070979581 |
| SNORA3 | 3.806720319 | 0.027961222 | NA |
| SNED1 | 3.799165163 | 1.13E-13 | 2.34E-11 |
| TSPAN11 | 3.789313914 | 1.11E-08 | 8.34E-07 |
| RP11-196B3.2 | 3.784638181 | 0.012868981 | 0.106215085 |
| CLEC2B | 3.78225573 | 0.020569032 | 0.145939015 |
| GAD1 | 3.78181585 | 0.015328516 | 0.120443042 |
| PLXDC1 | 3.774762668 | 2.02E-12 | 3.36E-10 |
| GSTM5 | 3.769901292 | 3.24E-06 | 0.000128113 |
| PARK2 | 3.761268592 | 2.03E-07 | 1.10E-05 |
| RTP4 | 3.751575402 | 0.000177791 | 0.003908189 |
| UNC5C | 3.736797407 | 1.73E-06 | 7.26E-05 |
| KRT7-AS | 3.736591183 | 0.000766917 | 0.012435052 |
| MIR628 | 3.734929472 | 0.031678739 | 0.192618676 |
| CD163 | 3.730738166 | 0.013408309 | 0.109608745 |
| BEAN1 | 3.730365374 | 1.59E-09 | 1.50E-07 |
| RP11-815M8.1 | 3.728880573 | 0.012916877 | 0.106450204 |
| TBX1 | 3.728684083 | 0.000167293 | 0.003725966 |
| RP11-713M15.2 | 3.727685444 | 0.000839148 | 0.01329187 |
| DAB1 | 3.723708788 | 2.44E-07 | 1.30E-05 |
| HAGLROS | 3.721683269 | 0.007125792 | 0.069956746 |
| AC011524.1 | 3.716373072 | 0.026096831 | 0.17002476 |
| AC005301.9 | 3.71199399 | 0.00044628 | 0.008077956 |
| IL12A-AS1 | 3.708438254 | 0.009265937 | 0.083790658 |
| ABCA9 | 3.692561511 | 3.32E-05 | 0.000946856 |
| WSCD1 | 3.686223623 | 0.020872629 | 0.147188599 |
| SOD3 | 3.683050558 | 6.75E-06 | 0.000241401 |
| CTTNBP2 | 3.680026566 | 1.33E-08 | 9.86E-07 |
| RP11-134O21.1 | 3.67562409 | 0.008566531 | 0.079129501 |
| KCND3 | 3.672883433 | 2.40E-08 | 1.68E-06 |
| CDHR1 | 3.667737726 | 5.57E-05 | 0.001473406 |
| CYP19A1 | 3.65817683 | 0.004670112 | 0.050525225 |
| CLUHP10 | 3.652180045 | 0.040288739 | 0.225243211 |

| | | | |
|---|---|---|---|
| XXyac-YR38GF2.1 | 3.651606512 | 0.001547821 | 0.021504815 |
| TCF7 | 3.646845402 | 9.59E-06 | 0.000328357 |
| NOMO3 | 3.641399741 | 5.69E-22 | 3.57E-19 |
| BRINP1 | 3.640823866 | 0.003976959 | 0.044879895 |
| RP11-314C9.1 | 3.631522568 | 0.022097189 | 0.152535487 |
| AC073115.6 | 3.627366563 | 0.028188833 | 0.178166436 |
| RUBCNL | 3.606280296 | 0.000735011 | 0.012035706 |
| RP1-28O10.1 | 3.603251861 | 0.017224645 | 0.130032434 |
| MB | 3.599117695 | 0.034786898 | 0.204544348 |
| RP11-13P5.2 | 3.594913704 | 0.005145724 | 0.054350934 |
| ATP1A2 | 3.592925442 | 0.01930298 | 0.140305928 |
| GPR150 | 3.591852779 | 5.77E-05 | 0.001508647 |
| RP11-585P4.6 | 3.587757131 | 0.03521704 | 0.206354545 |
| EFHD1 | 3.586044945 | 1.41E-05 | 0.000457416 |
| KIAA1755 | 3.569223236 | 2.09E-05 | 0.000639797 |
| MPZ | 3.547237782 | 6.07E-06 | 0.000220573 |
| RP11-403B2.7 | 3.545771688 | 0.020784599 | 0.146851054 |
| LRRC15 | 3.526744199 | 0.007436215 | 0.071832861 |
| RP11-822E23.6 | 3.520310534 | 0.001236624 | 0.018066825 |
| EFEMP1 | 3.507978045 | 1.40E-07 | 8.01E-06 |
| TMED11P | 3.507112685 | 0.026235991 | 0.170537827 |
| DUSP15 | 3.503150654 | 0.030503646 | 0.187761224 |
| RAB39B | 3.501186863 | 2.06E-06 | 8.52E-05 |
| WNT11 | 3.497885053 | 0.000310739 | 0.00607453 |
| PPP2R2C | 3.489656516 | 0.00244194 | 0.030481976 |
| RP11-180P8.1 | 3.479828969 | 0.009682378 | 0.086522207 |
| RP11-495P10.9 | 3.478952081 | 0.00039136 | 0.007269921 |
| RP11-614F17.2 | 3.472012752 | 0.048779527 | 0.254228417 |
| HCG22 | 3.470987968 | 0.039427867 | 0.222299805 |
| RASA4DP | 3.459401787 | 0.001556204 | 0.021560869 |
| THRB | 3.457260192 | 3.72E-07 | 1.87E-05 |
| LINC01415 | 3.454225973 | 1.18E-06 | 5.25E-05 |
| PODXL | 3.454099176 | 0.011002182 | 0.095160739 |
| AC006116.12 | 3.449700137 | 0.027837698 | 0.176908432 |
| EXOC3L2 | 3.449505689 | 0.03687194 | 0.212780402 |
| KRT16 | 3.446113363 | 0.027000598 | 0.173477811 |
| C2orf88 | 3.446090587 | 4.82E-14 | 1.08E-11 |
| DLX3 | 3.438226366 | 0.012634191 | 0.104928203 |
| RSPH1 | 3.429414147 | 0.020565667 | 0.145939015 |
| AP001476.2 | 3.420451956 | 0.010569257 | 0.09219418 |
| CTC-558O2.2 | 3.419257164 | 0.000148139 | 0.003353385 |
| GREM2 | 3.411971458 | 0.004186431 | 0.046695485 |
| KLF15 | 3.405911552 | 0.001046937 | 0.015960883 |
| RPSAP70 | 3.404388853 | 0.000275039 | 0.005535609 |
| CPN2 | 3.395494765 | 0.024786326 | 0.164676688 |
| CTD-2207P18.1 | 3.394532177 | 0.000630591 | 0.010657867 |

| | | | |
|---|---|---|---|
| RP11-357H14.16 | 3.390300829 | 4.95E-06 | 0.000185751 |
| DCAF12L2 | 3.384253585 | 0.048799155 | 0.254228417 |
| ASPA | 3.382559763 | 0.001419162 | 0.020096673 |
| SNTB1 | 3.375874595 | 2.41E-05 | 0.000726076 |
| IL20RA | 3.370160328 | 0.00240655 | 0.030203032 |
| CLIC2 | 3.368514916 | 0.000381165 | 0.007140911 |
| PMS2P2 | 3.365753331 | 0.01087719 | 0.09442968 |
| RPL23AP87 | 3.365738028 | 0.018826217 | 0.137853651 |
| ABCG1 | 3.365640589 | 0.024966046 | 0.165369905 |
| RP11-648L3.2 | 3.360875683 | 0.043079249 | 0.23526825 |
| PLXNA4 | 3.355581612 | 1.61E-15 | 4.53E-13 |
| HCG4B | 3.340292497 | 0.008350307 | 0.077632498 |
| IL1RL1 | 3.329664894 | 0.017408816 | 0.130992272 |
| CECR1 | 3.329143118 | 0.000834079 | 0.013222091 |
| CTD-2303H24.2 | 3.327548643 | 4.13E-06 | 0.000158303 |
| ITGBL1 | 3.318562486 | 3.36E-12 | 5.23E-10 |
| CX3CR1 | 3.3068961 | 0.048466995 | 0.253461012 |
| DPEP1 | 3.305046907 | 0.011356246 | 0.0975418 |
| CASC15 | 3.286999611 | 2.20E-05 | 0.000668936 |
| BARX1 | 3.282895896 | 0.047524877 | 0.250381317 |
| PRTG | 3.281838033 | 5.12E-06 | 0.000190747 |
| GSC | 3.272639315 | 3.78E-07 | 1.88E-05 |
| RP11-9M16.2 | 3.270251944 | 0.025102882 | 0.165726587 |
| GOLGA8O | 3.253541276 | 0.012729509 | 0.105420901 |
| RNF212 | 3.252238485 | 4.70E-08 | 3.07E-06 |
| CILP2 | 3.247631898 | 0.000127342 | 0.002958223 |
| DLX2 | 3.240084514 | 0.000299087 | 0.005905819 |
| MFAP5 | 3.240000342 | 3.69E-06 | 0.000143393 |
| EVI2B | 3.234626801 | 0.000687764 | 0.011436086 |
| KCNJ15 | 3.221352878 | 2.50E-06 | 0.00010154 |
| LAMA4 | 3.208868924 | 2.12E-05 | 0.000645657 |
| OLFML2B | 3.207595857 | 3.56E-13 | 7.10E-11 |
| ROBO4 | 3.203096582 | 5.85E-10 | 6.14E-08 |
| KCNK15-AS1 | 3.197945373 | 0.017706344 | 0.132303056 |
| UNC5B | 3.196451865 | 5.23E-09 | 4.26E-07 |
| RP3-395M20.8 | 3.183832291 | 0.002632929 | 0.032233286 |
| PGF | 3.177122062 | 1.26E-05 | 0.000414665 |
| SLC7A8 | 3.17273193 | 3.62E-06 | 0.000141561 |
| EBF1 | 3.172588594 | 3.47E-05 | 0.000977694 |
| RP11-343B18.2 | 3.169709592 | 0.019442104 | 0.140777061 |
| ENPP5 | 3.166647701 | 0.00578827 | 0.059602419 |
| LINC01138 | 3.164954941 | 0.000483137 | 0.008589184 |
| FAM78B | 3.163343307 | 0.005288293 | 0.055544202 |
| CADPS2 | 3.157777369 | 7.40E-10 | 7.64E-08 |
| ANO4 | 3.156827531 | 3.55E-05 | 0.000997905 |
| PAPPA2 | 3.154550503 | 0.012798183 | 0.105829828 |

| | | | |
|---|---|---|---|
| ACP5 | 3.149756972 | 0.000186568 | 0.004020558 |
| RP4-668J24.2 | 3.14973489 | 0.011548698 | 0.09878973 |
| RP1-142L7.5 | 3.147445808 | 0.002116247 | 0.027406278 |
| SPATA22 | 3.129246068 | 0.023732128 | 0.160683513 |
| ESM1 | 3.117163282 | 0.001162745 | 0.017231692 |
| OR2A1 | 3.111534992 | 0.02648632 | 0.171605699 |
| GRM1 | 3.108122216 | 0.022307262 | 0.153602235 |
| PCDHB3 | 3.103935002 | 0.000180311 | 0.003944158 |
| APOL3 | 3.10132413 | 6.62E-07 | 3.12E-05 |
| EMCN | 3.097144707 | 0.007887834 | 0.074459256 |
| PWP2 | 3.096874636 | 0.00124561 | 0.018156935 |
| RP11-334A14.2 | 3.094761619 | 0.030969573 | 0.189620567 |
| FAM84A | 3.092119838 | 7.00E-07 | 3.28E-05 |
| DRAXIN | 3.0861329 | 0.008322606 | 0.07755173 |
| AC002480.3 | 3.083273219 | 9.09E-05 | 0.002221231 |
| EBF2 | 3.081364785 | 3.02E-05 | 0.000874841 |
| GJA5 | 3.067673829 | 0.049310468 | 0.255778566 |
| RNU1-91P | 3.062345078 | 0.008781779 | 0.080574932 |
| MAFB | 3.056495583 | 0.00117389 | 0.017353631 |
| RP11-154F14.2 | 3.053018599 | 0.040635492 | 0.226480829 |
| GLIPR1L2 | 3.05012445 | 0.007002524 | 0.069157902 |
| RP11-672A2.4 | 3.049465741 | 1.27E-05 | 0.000415489 |
| RP11-466A19.1 | 3.04637315 | 0.003577133 | 0.041280986 |
| AC069513.4 | 3.045132366 | 0.048081984 | 0.252404111 |
| COL14A1 | 3.044907322 | 2.03E-05 | 0.000623285 |
| SMPD5 | 3.03458509 | 0.036791877 | 0.212461228 |
| VEGFD | 3.033365952 | 0.032836258 | 0.197197323 |
| EPHB3 | 3.032462458 | 8.39E-09 | 6.46E-07 |
| RASSF2 | 3.025217892 | 2.51E-09 | 2.20E-07 |
| AEBP1 | 3.021496348 | 0.017778075 | 0.132537706 |
| RP11-121P12.1 | 3.020467539 | 0.000116748 | 0.002748079 |
| RGN | 3.020356972 | 4.73E-06 | 0.000178029 |
| RP11-863P13.3 | 3.018576665 | 0.004053915 | 0.045537527 |
| FRMD7 | 3.01146108 | 0.032897231 | 0.197347405 |
| RNF157 | 3.009961306 | 7.86E-06 | 0.000276751 |
| ACSL5 | 3.009712394 | 1.55E-06 | 6.55E-05 |
| SLC5A9 | 3.003242561 | 0.008615946 | 0.079452136 |
| EVI2A | 3.000805565 | 0.000928933 | 0.014483964 |
| GPX3 | 2.991729795 | 0.000114282 | 0.002698725 |
| PTPRN | 2.980786299 | 5.72E-13 | 1.10E-10 |
| LINC02126 | 2.976819095 | 0.049754738 | 0.256870794 |
| RP11-562I5.2 | 2.972139204 | 0.03024104 | 0.186881108 |
| RP11-397O8.7 | 2.971014675 | 0.0054433 | 0.056702338 |
| PRRG4 | 2.968417782 | 0.047370239 | 0.2503067 |
| SALL1 | 2.967409061 | 0.022466952 | 0.154314339 |
| MRO | 2.960661415 | 0.047509218 | 0.250381317 |

| | | | |
|---|---|---|---|
| CADPS | 2.959494767 | 0.000221654 | 0.004658924 |
| THBS4 | 2.956559112 | 0.001135821 | 0.016916254 |
| CHRD | 2.955814413 | 0.000293883 | 0.005818784 |
| RNU7-57P | 2.953125485 | 0.002528509 | 0.031286193 |
| FPR3 | 2.944978593 | 0.034400988 | 0.202982484 |
| LINC00517 | 2.942622037 | 0.014830523 | 0.117667999 |
| TMEM204 | 2.941294064 | 7.58E-25 | 5.74E-22 |
| BMF | 2.939377095 | 1.08E-05 | 0.000363168 |
| C2 | 2.93233533 | 0.000408369 | 0.007541073 |
| COL6A2 | 2.930811148 | 2.45E-19 | 1.31E-16 |
| ACSM3 | 2.928088533 | 0.029824272 | 0.18508766 |
| NDNF | 2.919795693 | 0.001539583 | 0.021417477 |
| LAMA2 | 2.913026695 | 1.79E-08 | 1.28E-06 |
| RASA4B | 2.910088176 | 1.50E-12 | 2.55E-10 |
| STAC2 | 2.909854725 | 0.021862754 | 0.15130129 |
| LAPTM5 | 2.901892065 | 0.004401462 | 0.048405903 |
| C10orf11 | 2.901808438 | 0.002945048 | 0.035158072 |
| IL34 | 2.899352242 | 0.013620171 | 0.110789513 |
| NPAS3 | 2.898622774 | 0.000583202 | 0.010011186 |
| CCDC13 | 2.893684067 | 0.000622372 | 0.010584186 |
| GPR158 | 2.889840923 | 0.004860667 | 0.052041632 |
| FOXO6 | 2.886611419 | 0.015407559 | 0.12079751 |
| C15orf59 | 2.88626132 | 2.85E-05 | 0.000832748 |
| DLX5 | 2.883289783 | 0.036572498 | 0.211751771 |
| VSTM4 | 2.879453946 | 2.39E-12 | 3.94E-10 |
| HAS2 | 2.878133229 | 0.02814166 | 0.178122257 |
| RP11-680H20.2 | 2.862530984 | 0.030396228 | 0.187385041 |
| AFAP1L2 | 2.860710453 | 0.007838111 | 0.074212932 |
| FAM19A5 | 2.859677195 | 0.047530541 | 0.250381317 |
| PROM2 | 2.859451766 | 0.007918797 | 0.074655163 |
| GAS7 | 2.85245885 | 5.82E-07 | 2.77E-05 |
| PCDHB5 | 2.852302685 | 4.05E-07 | 2.02E-05 |
| TSKS | 2.851961516 | 0.011571259 | 0.098928398 |
| NHS | 2.847051818 | 5.38E-08 | 3.42E-06 |
| PDGFRB | 2.843483973 | 5.76E-08 | 3.62E-06 |
| AP000473.5 | 2.840982685 | 0.028517872 | 0.179416425 |
| AC017002.1 | 2.84003377 | 0.015695843 | 0.122365107 |
| MXRA5 | 2.837638656 | 3.10E-10 | 3.39E-08 |
| HSD11B1 | 2.834388537 | 0.017055508 | 0.129200185 |
| PPP1R14A | 2.833953434 | 0.000135106 | 0.003098913 |
| STEAP2 | 2.827579653 | 4.85E-07 | 2.35E-05 |
| COL6A3 | 2.820203174 | 4.23E-14 | 9.56E-12 |
| IL13RA2 | 2.815989237 | 0.045229348 | 0.242720509 |
| NOVA1 | 2.814278146 | 0.001440349 | 0.020332045 |
| IGFBP5 | 2.810533464 | 0.001045767 | 0.015954122 |
| FAM106A | 2.8093189 | 0.000336877 | 0.006454497 |

| | | | |
|---|---|---|---|
| HLA-DPA1 | 2.808330983 | 4.75E-08 | 3.09E-06 |
| COL5A3 | 2.808291598 | 2.03E-17 | 8.25E-15 |
| DAPK2 | 2.807815915 | 0.000439624 | 0.007993543 |
| SLC6A15 | 2.805940248 | 0.001315657 | 0.018953644 |
| KLF14 | 2.797714267 | 0.014318499 | 0.114846846 |
| ACVRL1 | 2.787425252 | 1.03E-07 | 6.02E-06 |
| LINC00856 | 2.781841811 | 0.00066681 | 0.011158261 |
| MSC | 2.758174797 | 0.000590463 | 0.010114555 |
| LINC01121 | 2.757860646 | 0.04080318 | 0.226904983 |
| NCAPGP1 | 2.753572152 | 0.015863871 | 0.123237109 |
| CNGA3 | 2.749519279 | 0.019814053 | 0.142752281 |
| EYA4 | 2.742098218 | 0.00155677 | 0.021560869 |
| RP11-815J4.6 | 2.737290977 | 0.001779929 | 0.023955873 |
| RP11-20D14.6 | 2.73618574 | 0.022076465 | 0.152443651 |
| RP11-361F15.5 | 2.734962947 | 0.042237395 | 0.23287358 |
| LINC01176 | 2.733175998 | 0.042096524 | 0.232388915 |
| ABCB4 | 2.731989224 | 0.011545854 | 0.09878973 |
| AC007405.6 | 2.730434562 | 0.000303281 | 0.005961811 |
| SFMBT2 | 2.72909073 | 0.016015443 | 0.124063128 |
| TNFSF9 | 2.720109843 | 7.59E-06 | 0.000268506 |
| ITGA9 | 2.718757008 | 4.28E-06 | 0.0001628 |
| SORCS2 | 2.714270325 | 5.37E-07 | 2.57E-05 |
| SRRM3 | 2.710849072 | 8.73E-07 | 4.02E-05 |
| RP11-34P13.13 | 2.710237574 | 0.04776499 | 0.251287375 |
| AK4P3 | 2.709515272 | 0.010472167 | 0.091565727 |
| TDRD1 | 2.706051293 | 0.042134694 | 0.232445076 |
| SPESP1 | 2.70310063 | 0.008351385 | 0.077632498 |
| RASL11B | 2.699722937 | 0.000800597 | 0.012801386 |
| B3GALT2 | 2.699265629 | 6.09E-05 | 0.001573421 |
| PTGIS | 2.698167204 | 1.90E-05 | 0.000590514 |
| GPAM | 2.693980992 | 1.22E-16 | 4.26E-14 |
| DMGDH | 2.686404108 | 1.40E-07 | 8.01E-06 |
| HCG4P5 | 2.68380855 | 1.50E-05 | 0.00048325 |
| HTR2B | 2.683680655 | 0.023245065 | 0.158173149 |
| CACNB4 | 2.681200033 | 0.007797121 | 0.073984961 |
| TMEM100 | 2.677403158 | 0.002896519 | 0.034742385 |
| PGM5 | 2.67279858 | 0.024497987 | 0.163503755 |
| GCNT4 | 2.667674441 | 0.002474566 | 0.030774962 |
| CLEC3B | 2.667560868 | 0.001396081 | 0.019862014 |
| RP11-211G23.2 | 2.666853292 | 0.013391018 | 0.109561739 |
| ACE | 2.6616344 | 6.71E-07 | 3.16E-05 |
| CACNA1C | 2.655655022 | 4.11E-05 | 0.001122365 |
| DENND5B-AS1 | 2.654647941 | 0.035251164 | 0.206428548 |
| CCDC144B | 2.651096065 | 0.00029015 | 0.005759749 |
| HSPB2 | 2.642919904 | 7.37E-07 | 3.44E-05 |
| NTM | 2.641798327 | 6.89E-07 | 3.23E-05 |

| | | | |
|---|---|---|---|
| CFD | 2.641779923 | 0.002078105 | 0.027007983 |
| NCALD | 2.641735455 | 1.73E-05 | 0.000548421 |
| RP11-666A8.7 | 2.633903557 | 0.028795147 | 0.180801367 |
| LINC00623 | 2.625394454 | 0.032744057 | 0.196859176 |
| FMN1 | 2.623696859 | 4.43E-06 | 0.000167694 |
| VMO1 | 2.619760528 | 0.019256972 | 0.14021223 |
| CCDC152 | 2.610952595 | 0.00030174 | 0.005942158 |
| ADAMTSL3 | 2.609850752 | 0.004664032 | 0.050525225 |
| RP4-555D20.1 | 2.608083853 | 0.049449602 | 0.256083616 |
| TSPAN18 | 2.607706255 | 0.000222177 | 0.004664226 |
| C8orf31 | 2.605497347 | 9.98E-07 | 4.54E-05 |
| KCND2 | 2.604454017 | 0.000273431 | 0.005518423 |
| TRIB3 | 2.604368931 | 0.002422 | 0.030362194 |
| C12orf56 | 2.600249038 | 0.012909432 | 0.10642883 |
| MYL4 | 2.598623983 | 0.020230798 | 0.144473713 |
| DBP | 2.595074267 | 0.000621354 | 0.010575068 |
| PCDH18 | 2.591532779 | 6.70E-06 | 0.000240488 |
| FOXD2 | 2.583062179 | 1.29E-05 | 0.000422118 |
| SMIM25 | 2.582234135 | 0.044245653 | 0.239255886 |
| DAAM2 | 2.582132603 | 0.000645322 | 0.010881682 |
| SLC22A23 | 2.581602032 | 0.000376292 | 0.00706772 |
| NRCAM | 2.576195512 | 0.007425124 | 0.071758753 |
| IGFN1 | 2.568876169 | 0.025121892 | 0.1657516 |
| PECAM1 | 2.56868778 | 0.012573344 | 0.104601452 |
| FERMT3 | 2.560217211 | 2.73E-08 | 1.88E-06 |
| RP11-42I10.1 | 2.559199392 | 0.001558051 | 0.021565003 |
| C9orf106 | 2.552189423 | 0.030338416 | 0.187260511 |
| HTRA1 | 2.549290604 | 2.62E-06 | 0.000105125 |
| PATJ | 2.547655507 | 5.41E-10 | 5.74E-08 |
| CTC-297N7.7 | 2.538770413 | 0.036598131 | 0.211788396 |
| FOXP2 | 2.528794017 | 0.000142978 | 0.003260551 |
| YAP1P1 | 2.527303246 | 0.002418161 | 0.030331401 |
| WNT9A | 2.52718807 | 5.47E-06 | 0.000200851 |
| SATB2 | 2.524565747 | 4.29E-09 | 3.56E-07 |
| POSTN | 2.519718514 | 5.82E-05 | 0.001515922 |
| FGF14 | 2.516366958 | 9.51E-09 | 7.22E-07 |
| IFI30 | 2.514547299 | 0.000361192 | 0.006807411 |
| ARHGEF16 | 2.506195442 | 0.006381411 | 0.064336116 |
| NLRP10 | 2.506076 | 0.00045558 | 0.008185513 |
| SGCD | 2.504562992 | 1.41E-12 | 2.43E-10 |
| BMPR1B | 2.502147139 | 5.97E-07 | 2.84E-05 |
| TULP2 | 2.501648216 | 0.025451379 | 0.167421993 |
| RP5-1021I20.1 | 2.499782652 | 0.016667187 | 0.127305245 |
| CYP1B1-AS1 | 2.498855445 | 0.001730374 | 0.023447151 |
| FBXO39 | 2.498770244 | 0.028032872 | 0.177690014 |
| PCDHB6 | 2.490460765 | 0.015274563 | 0.12019131 |

| | | | |
|---|---|---|---|
| NTRK2 | 2.489767956 | 0.000115428 | 0.002722863 |
| RP11-212I21.3 | 2.485310393 | 1.86E-07 | 1.03E-05 |
| ERICH2 | 2.478582308 | 0.000519009 | 0.00909427 |
| NPR3 | 2.477266293 | 0.000484125 | 0.008599793 |
| LRP1-AS | 2.477171602 | 0.0273324 | 0.174945626 |
| PCDHB10 | 2.475010393 | 0.000202027 | 0.004298797 |
| DLEU7 | 2.472428913 | 0.010496077 | 0.091738226 |
| METTL15P1 | 2.471386859 | 0.043425067 | 0.236685496 |
| LINC00578 | 2.469550388 | 0.020324945 | 0.144675095 |
| AC131056.3 | 2.466592227 | 0.030345114 | 0.187260511 |
| HERC2P10 | 2.46153553 | 0.006753734 | 0.067072623 |
| EGR3 | 2.460601358 | 1.27E-05 | 0.000415489 |
| OPCML | 2.46048539 | 0.004391424 | 0.048362984 |
| COL6A1 | 2.460294734 | 1.88E-15 | 5.16E-13 |
| ACKR4 | 2.458715033 | 0.002146056 | 0.027661679 |
| RGPD2 | 2.458548914 | 0.005380393 | 0.056157183 |
| PAX9 | 2.45836342 | 0.004149812 | 0.046400903 |
| COL16A1 | 2.457501709 | 1.96E-05 | 0.000606157 |
| CNIH3 | 2.453018848 | 3.19E-11 | 4.24E-09 |
| PTPN20 | 2.451311043 | 0.021640494 | 0.150142048 |
| TMEM169 | 2.44542612 | 2.66E-05 | 0.000782501 |
| RP11-12A20.7 | 2.438737513 | 0.000798906 | 0.012801386 |
| PIWIL4 | 2.438032057 | 7.77E-07 | 3.61E-05 |
| RP11-225H22.4 | 2.436023311 | 0.020303686 | 0.144597558 |
| CSDC2 | 2.43508904 | 3.17E-07 | 1.64E-05 |
| LTBP2 | 2.434081277 | 1.93E-07 | 1.05E-05 |
| FKBP9P1 | 2.432652895 | 0.000359346 | 0.006784283 |
| FAM86GP | 2.432366027 | 0.008871616 | 0.08108 |
| PPARGC1A | 2.425719297 | 0.039081395 | 0.220857201 |
| HOXB8 | 2.421933875 | 0.007488675 | 0.072240598 |
| MSC-AS1 | 2.420354324 | 0.000435406 | 0.007933504 |
| RARRES3 | 2.420320793 | 0.013300839 | 0.109000304 |
| TNFRSF1B | 2.419894447 | 1.46E-06 | 6.33E-05 |
| MMP2 | 2.419466986 | 2.94E-07 | 1.54E-05 |
| AC002480.4 | 2.419238116 | 0.017684302 | 0.132261545 |
| IL15RA | 2.416013456 | 1.42E-05 | 0.000459694 |
| ZNF385D | 2.415762833 | 4.30E-06 | 0.000163246 |
| RAB3IL1 | 2.410496337 | 8.04E-05 | 0.001996077 |
| SLIT3 | 2.409924504 | 0.000212347 | 0.004485355 |
| CKB | 2.403220403 | 0.046248244 | 0.246799799 |
| LINC00862 | 2.398047761 | 0.022031132 | 0.152274407 |
| FOXC1 | 2.39663693 | 0.000182238 | 0.003962622 |
| MXRA5Y | 2.390186459 | 0.011003795 | 0.095160739 |
| RFX8 | 2.38919521 | 6.68E-06 | 0.000240395 |
| FAM105A | 2.378155787 | 0.01165008 | 0.099333621 |
| EPHB1 | 2.377740088 | 0.00031643 | 0.006168523 |

| | | | |
|---|---|---|---|
| EMP1 | 2.376623872 | 8.46E-09 | 6.49E-07 |
| FIBIN | 2.374191866 | 0.001077911 | 0.016308414 |
| NDRG1 | 2.372357955 | 2.21E-11 | 2.98E-09 |
| ADCY4 | 2.364107999 | 0.017334011 | 0.130653638 |
| RP11-417L19.5 | 2.364094815 | 0.000127212 | 0.002958223 |
| BCL2 | 2.360817902 | 5.65E-05 | 0.001486544 |
| RP11-400K9.4 | 2.357956194 | 0.001898435 | 0.025195325 |
| NLRP2 | 2.357624068 | 0.004370298 | 0.048154492 |
| RP11-495P10.1 | 2.354086858 | 0.014922304 | 0.118182491 |
| LRRN4CL | 2.34894669 | 0.001176295 | 0.017365791 |
| HAS2-AS1 | 2.348862743 | 0.015476947 | 0.121047482 |
| HMOX1 | 2.345468197 | 0.00113489 | 0.016913875 |
| SLC30A3 | 2.342764742 | 0.048753985 | 0.254199388 |
| ZNF385B | 2.34214402 | 0.01011395 | 0.089503766 |
| AXIN2 | 2.329782815 | 1.04E-08 | 7.82E-07 |
| ADAM32 | 2.329557137 | 3.35E-05 | 0.000953694 |
| SLC22A15 | 2.328657882 | 2.23E-08 | 1.56E-06 |
| RP11-640L9.2 | 2.327318404 | 0.039343319 | 0.221994275 |
| AUTS2 | 2.326802953 | 4.65E-36 | 7.28E-33 |
| MCOLN3 | 2.326202232 | 0.002754173 | 0.033344948 |
| PIWIL2 | 2.324724234 | 0.034703607 | 0.204218812 |
| TSPAN12 | 2.324207976 | 0.00025738 | 0.005281945 |
| RP11-403I13.8 | 2.322868318 | 0.004217357 | 0.046940829 |
| OLFML1 | 2.321517021 | 1.08E-05 | 0.000363168 |
| NOTCH3 | 2.319083347 | 0.002231693 | 0.028429669 |
| RP11-755E23.2 | 2.317890889 | 0.05019242 | 0.258115638 |
| PACRG | 2.317632717 | 0.009520884 | 0.085707487 |
| RPL23AP49 | 2.311588835 | 0.004187504 | 0.046695485 |
| PCDH1 | 2.310276819 | 0.001251466 | 0.018222224 |
| TDRD6 | 2.310056298 | 0.006049994 | 0.061789924 |
| APOL6 | 2.309027905 | 5.18E-08 | 3.33E-06 |
| TRPA1 | 2.302579063 | 0.000468233 | 0.00834451 |
| TTLL9 | 2.300556638 | 0.040397686 | 0.225520714 |
| HHIP | 2.299483402 | 0.012463485 | 0.104082199 |
| MMP11 | 2.297082915 | 0.005632785 | 0.058343742 |
| FBXO32 | 2.29667275 | 0.002102271 | 0.027241362 |
| AC006126.3 | 2.294817713 | 0.014892628 | 0.118032682 |
| STEAP1 | 2.293561968 | 1.09E-05 | 0.000365082 |
| SYT12 | 2.289635507 | 0.037160301 | 0.213982729 |
| IRX3 | 2.288241488 | 1.20E-13 | 2.45E-11 |
| SP6 | 2.285884396 | 0.031343674 | 0.191323741 |
| F2R | 2.285676979 | 2.57E-08 | 1.79E-06 |
| PKD1P1 | 2.284658586 | 0.002167867 | 0.02789365 |
| NDUFA4L2 | 2.280769714 | 0.000120064 | 0.00282008 |
| RHD | 2.276382241 | 0.028362074 | 0.1787438 |
| ZMAT1 | 2.272419584 | 0.001317426 | 0.018964365 |

| | | | |
|---|---|---|---|
| TSC22D3 | 2.271838697 | 3.63E-06 | 0.000141561 |
| CXCL12 | 2.271604333 | 3.34E-12 | 5.23E-10 |
| WNT7B | 2.267969076 | 0.024291119 | 0.162816549 |
| NR5A2 | 2.267872988 | 0.025645598 | 0.168044544 |
| HLA-H | 2.264102675 | 0.000743119 | 0.012157011 |
| HECW1 | 2.263831034 | 0.007138509 | 0.069961577 |
| INMT | 2.260755715 | 0.023999141 | 0.161898262 |
| PLPP3 | 2.260441004 | 3.18E-08 | 2.15E-06 |
| AOC3 | 2.258662024 | 0.004162246 | 0.046492546 |
| EDA2R | 2.25484293 | 7.57E-06 | 0.000268453 |
| PCDHB7 | 2.248653116 | 7.22E-05 | 0.001833399 |
| RGCC | 2.247632709 | 0.030126827 | 0.186437892 |
| USP32P2 | 2.246718601 | 0.000285633 | 0.005696555 |
| PALM2 | 2.245910206 | 0.000320625 | 0.006219153 |
| SEMA7A | 2.240535654 | 0.001708771 | 0.023211777 |
| AC093627.10 | 2.239528199 | 0.018478398 | 0.13626188 |
| FZD3 | 2.237890486 | 0.001553903 | 0.021560869 |
| AC144831.1 | 2.236414073 | 2.28E-17 | 8.94E-15 |
| BAIAP2L2 | 2.224495097 | 0.000237297 | 0.00492044 |
| MAMDC2 | 2.223595935 | 0.017638117 | 0.132063145 |
| CPAMD8 | 2.22329575 | 0.009041699 | 0.08227961 |
| SH3BGR | 2.223259944 | 8.47E-05 | 0.002083432 |
| SOBP | 2.22192702 | 5.51E-12 | 8.34E-10 |
| ZNF391 | 2.221866178 | 1.81E-05 | 0.000565196 |
| MYEOV | 2.218970021 | 0.012152419 | 0.102341558 |
| RAP1GAP | 2.217532791 | 0.000321848 | 0.006237363 |
| HOXD3 | 2.216185296 | 8.89E-08 | 5.30E-06 |
| ANPEP | 2.215339934 | 1.03E-06 | 4.65E-05 |
| LRRC32 | 2.213859956 | 0.000147321 | 0.003345678 |
| TLR4 | 2.213767203 | 1.16E-14 | 2.87E-12 |
| ISL2 | 2.210605358 | 0.042908481 | 0.234931164 |
| ANGPTL4 | 2.210522331 | 0.004732084 | 0.051088803 |
| RP4-635E18.6 | 2.210212432 | 0.032083512 | 0.193950976 |
| RENBP | 2.207665987 | 0.042602647 | 0.234205432 |
| TBX4 | 2.204695623 | 0.045836618 | 0.245244001 |
| DACT3 | 2.198804676 | 2.48E-07 | 1.32E-05 |
| LSP1 | 2.197000642 | 0.019764932 | 0.142585692 |
| CUBN | 2.19419981 | 9.98E-06 | 0.000339505 |
| SLC44A3 | 2.194058812 | 0.004789193 | 0.051452162 |
| THBS1 | 2.19303992 | 1.47E-06 | 6.38E-05 |
| DOK1 | 2.189492408 | 1.79E-09 | 1.64E-07 |
| PCDHB2 | 2.188229397 | 0.042394871 | 0.233389881 |
| TNNC2 | 2.188224312 | 0.014473035 | 0.115584071 |
| TRIB2 | 2.18597843 | 5.59E-09 | 4.53E-07 |
| RP11-794G24.1 | 2.179074406 | 0.047383667 | 0.2503067 |
| RP11-867G23.13 | 2.17665366 | 0.013865689 | 0.112328468 |

| | | | |
|---|---|---|---|
| KRT86 | 2.176299777 | 0.01803374 | 0.13397365 |
| MFAP3L | 2.167174025 | 6.36E-09 | 5.05E-07 |
| GRASP | 2.166852359 | 0.010903415 | 0.094582491 |
| BHMT2 | 2.158582313 | 0.000618192 | 0.010537604 |
| FLG | 2.151695311 | 0.017610187 | 0.131944083 |
| ISM1 | 2.148129353 | 0.016507589 | 0.126490911 |
| GALNT12 | 2.145884076 | 0.009780659 | 0.087219139 |
| P2RX7 | 2.14526348 | 0.01763184 | 0.132061214 |
| RP11-111F16.2 | 2.144010693 | 0.000590607 | 0.010114555 |
| RHOQP2 | 2.14362249 | 0.006120935 | 0.062280886 |
| AKR1C1 | 2.142192602 | 0.003913572 | 0.044324187 |
| CLDN14 | 2.136458733 | 0.001541666 | 0.021432866 |
| IFITM10 | 2.134233579 | 0.000310953 | 0.00607453 |
| LRP1 | 2.13423278 | 0.000442534 | 0.008030043 |
| PPL | 2.134046221 | 0.033729108 | 0.200202698 |
| C1S | 2.130890804 | 0.002649353 | 0.032361635 |
| CMAHP | 2.130442198 | 1.55E-06 | 6.55E-05 |
| NRXN2 | 2.128959479 | 0.007215355 | 0.070413902 |
| SULT1A1 | 2.12599532 | 0.020765458 | 0.146763085 |
| KCNK2 | 2.12111849 | 2.87E-08 | 1.97E-06 |
| STK4-AS1 | 2.119893637 | 0.036648461 | 0.211840923 |
| CLGN | 2.116989079 | 0.000769718 | 0.012466074 |
| MAP6 | 2.112626993 | 1.57E-07 | 8.84E-06 |
| JMY | 2.106330752 | 0.000226924 | 0.004735717 |
| LINC01422 | 2.105404642 | 0.007700572 | 0.073545993 |
| CGREF1 | 2.104821993 | 0.005139328 | 0.054350934 |
| BGN | 2.102137402 | 0.00030572 | 0.005993638 |
| SYNDIG1 | 2.100922278 | 0.004041962 | 0.045426512 |
| CTD-2517O10.6 | 2.098479408 | 0.024401519 | 0.163257249 |
| ALDH1A2 | 2.097600635 | 0.046098027 | 0.246117914 |
| ATF5 | 2.095105276 | 3.91E-14 | 8.94E-12 |
| EGFLAM | 2.089980665 | 2.03E-06 | 8.44E-05 |
| GABRB3 | 2.089791712 | 0.023994963 | 0.161898262 |
| LINC01503 | 2.084954719 | 0.010258658 | 0.090383309 |
| MIRLET7BHG | 2.081649937 | 3.71E-05 | 0.001035228 |
| EPB41L3 | 2.079829167 | 2.56E-06 | 0.0001034 |
| MAP4K1 | 2.077779428 | 0.035791998 | 0.208553748 |
| NMNAT2 | 2.077762232 | 0.000159351 | 0.003578148 |
| LINC01018 | 2.077293129 | 0.01824187 | 0.134834957 |
| DOCK9-AS2 | 2.070494831 | 0.028391547 | 0.178826802 |
| PTPRE | 2.069593194 | 0.00467067 | 0.050525225 |
| ALOX12P2 | 2.065966281 | 0.000535765 | 0.009335665 |
| FAM149A | 2.064794983 | 8.15E-06 | 0.000285237 |
| RP11-182J1.18 | 2.064599633 | 0.013685863 | 0.111117859 |
| KLF4 | 2.063921428 | 4.90E-05 | 0.001312175 |
| CCDC151 | 2.062265201 | 0.007482422 | 0.07221706 |

| | | | |
|---|---|---|---|
| NPIPA1 | 2.062254094 | 2.85E-05 | 0.000832748 |
| PARP10 | 2.052818464 | 1.67E-08 | 1.21E-06 |
| SLC31A2 | 2.052526671 | 0.000447977 | 0.008085542 |
| VDR | 2.049757163 | 3.87E-05 | 0.001073205 |
| IRAK3 | 2.046696378 | 0.000692929 | 0.011498853 |
| HOXB3 | 2.045851459 | 3.92E-06 | 0.000151001 |
| ZNF521 | 2.045428849 | 2.58E-05 | 0.000766852 |
| PTGER1 | 2.04488706 | 0.007689095 | 0.073468361 |
| OTUD7A | 2.040739384 | 0.00492921 | 0.052602521 |
| SYNE3 | 2.040733196 | 1.12E-18 | 5.32E-16 |
| PCDHB15 | 2.040245459 | 0.007015027 | 0.069228819 |
| RPLP0P2 | 2.038943191 | 0.021256875 | 0.148513798 |
| MAB21L3 | 2.038773827 | 0.014006192 | 0.113037159 |
| AP001189.4 | 2.03604401 | 0.000168115 | 0.003740469 |
| MYOSLID | 2.035228228 | 7.29E-05 | 0.001841335 |
| SPAAR | 2.03225967 | 0.013940313 | 0.11276644 |
| SKOR1 | 2.028037159 | 0.049401177 | 0.256009164 |
| SYN2 | 2.02787596 | 0.033680668 | 0.200180366 |
| ABCC9 | 2.026721506 | 0.000392882 | 0.007279608 |
| MESP2 | 2.02541857 | 0.006839031 | 0.067827604 |
| TNFRSF11A | 2.023453488 | 0.043618492 | 0.237327003 |
| CLEC14A | 2.019229827 | 0.000993421 | 0.015369309 |
| RP11-134G8.7 | 2.015981725 | 0.014105344 | 0.113473793 |
| PDPN | 2.015327663 | 0.042721625 | 0.234508874 |
| FMNL1 | 2.013283946 | 0.030722172 | 0.188421308 |
| C1R | 2.01320797 | 0.012011674 | 0.101467885 |
| INSR | 2.008352981 | 2.22E-05 | 0.000675049 |
| GRB14 | 2.007701153 | 0.028285604 | 0.178415634 |
| TRPV4 | 2.002487611 | 2.21E-09 | 1.96E-07 |
| RP11-999E24.3 | 1.999598963 | 1.21E-05 | 0.00040163 |
| NOC2LP1 | 1.99834325 | 0.005627015 | 0.058311504 |
| OSCAR | 1.998301306 | 0.002100776 | 0.027238079 |
| GPC6 | 1.998270874 | 0.00191453 | 0.025317028 |
| FAM27E3 | 1.996574007 | 0.012288267 | 0.10321462 |
| AC027612.6 | 1.99656142 | 0.000448173 | 0.008085542 |
| RNF125 | 1.995616745 | 0.002512907 | 0.031145849 |
| LINC01119 | 1.993575312 | 0.000161715 | 0.003623805 |
| RP11-723O4.9 | 1.99311579 | 0.026900971 | 0.173261095 |
| ELFN1 | 1.992330316 | 0.024949652 | 0.165358018 |
| CCPG1 | 1.992026476 | 2.07E-05 | 0.00063574 |
| AK8 | 1.991482639 | 0.024262185 | 0.162672316 |
| PLAC9 | 1.989591157 | 2.02E-08 | 1.43E-06 |
| RASD1 | 1.989190113 | 0.00148136 | 0.02080543 |
| OR2S1P | 1.98827892 | 0.047043176 | 0.249524465 |
| RP11-420L9.2 | 1.986768067 | 0.028917633 | 0.181330921 |
| HLA-A | 1.9843252 | 0.001116949 | 0.016714612 |

| | | | |
|---|---|---|---|
| AC068282.3 | 1.983519631 | 0.018558828 | 0.136656684 |
| GRIA1 | 1.982126435 | 0.029981157 | 0.185798483 |
| RGS16 | 1.978029758 | 0.002132151 | 0.027563189 |
| LY96 | 1.976087429 | 9.23E-05 | 0.002249034 |
| WNT5A-AS1 | 1.973433767 | 2.05E-05 | 0.000627654 |
| LINC00982 | 1.973355124 | 0.025643051 | 0.168044544 |
| DDIT4 | 1.972155778 | 0.002859525 | 0.034392691 |
| TMEM59L | 1.971550416 | 0.001394654 | 0.019862014 |
| AC007099.1 | 1.968552275 | 0.021829694 | 0.151215605 |
| RP11-326C3.11 | 1.965850759 | 0.012565137 | 0.104586456 |
| WBP1LP2 | 1.965012158 | 0.013651535 | 0.11093552 |
| SYTL3 | 1.964468457 | 0.001964046 | 0.025785298 |
| LARGE1 | 1.963616956 | 5.78E-08 | 3.62E-06 |
| CHRNA9 | 1.95850411 | 0.025107171 | 0.165726587 |
| TGFBI | 1.958085995 | 0.000183003 | 0.00396712 |
| LINC00950 | 1.956869518 | 0.007739958 | 0.073748233 |
| PTPRZ1 | 1.954132887 | 0.0304751 | 0.1876404 |
| CLDN7 | 1.953823626 | 0.02953181 | 0.183807429 |
| AC016999.2 | 1.950769024 | 0.000180506 | 0.003944158 |
| RMDN2-AS1 | 1.947332119 | 0.038897217 | 0.220213455 |
| PTER | 1.94516118 | 3.48E-07 | 1.77E-05 |
| VSTM2L | 1.940341885 | 2.90E-05 | 0.000845546 |
| KB-1995A5.5 | 1.940259314 | 0.043099139 | 0.235288639 |
| SOX13 | 1.938047766 | 0.00024647 | 0.005077045 |
| SLC37A1 | 1.937203364 | 0.001385295 | 0.019798443 |
| IQCH-AS1 | 1.929692251 | 0.00028447 | 0.005678525 |
| INSIG1 | 1.928992799 | 0.011655464 | 0.099333621 |
| ADTRP | 1.922971504 | 0.016312595 | 0.125452455 |
| VWA5A | 1.920278486 | 0.003038506 | 0.035992843 |
| PTPN22 | 1.91934839 | 0.000149638 | 0.003380811 |
| RP11-573G6.4 | 1.918302337 | 0.000245493 | 0.005061688 |
| MEDAG | 1.91770717 | 1.30E-05 | 0.000424559 |
| FYB | 1.91612942 | 0.004888633 | 0.052290019 |
| HOXB4 | 1.915266619 | 0.002627081 | 0.032233286 |
| FHIT | 1.912709694 | 0.027161914 | 0.174233353 |
| PCK2 | 1.908463507 | 4.89E-06 | 0.000183708 |
| ADAMTS2 | 1.907801568 | 4.52E-10 | 4.83E-08 |
| GNG7 | 1.906510569 | 0.029617278 | 0.18421997 |
| RP5-1180D12.1 | 1.902402262 | 2.51E-06 | 0.000101797 |
| MKNK2 | 1.902110469 | 3.39E-05 | 0.000960176 |
| FAM196B | 1.897106015 | 1.50E-05 | 0.00048325 |
| LINC01232 | 1.894152804 | 4.07E-05 | 0.001116073 |
| RP11-434E6.5 | 1.891568465 | 0.006622614 | 0.066129683 |
| RP11-864N7.2 | 1.890539072 | 0.004229167 | 0.04702456 |
| SH3PXD2A | 1.888823521 | 7.63E-05 | 0.001914648 |
| TRIM47 | 1.885205752 | 0.001275818 | 0.018498945 |

| | | | |
|---|---|---|---|
| CORO2B | 1.883553131 | 0.000661261 | 0.011099265 |
| PTPRM | 1.882444881 | 7.44E-05 | 0.001870396 |
| SNAI1 | 1.879728036 | 0.001064774 | 0.016176604 |
| ITGA10 | 1.87595643 | 0.000328772 | 0.006333147 |
| FTH1P7 | 1.874392886 | 0.00725534 | 0.070678348 |
| TNFRSF14 | 1.873497951 | 0.007528205 | 0.072499453 |
| FRAT1 | 1.872045462 | 0.003829921 | 0.043534101 |
| ITGA11 | 1.871125897 | 0.001215393 | 0.017799267 |
| RP4-614C10.2 | 1.867850697 | 3.90E-06 | 0.000150624 |
| PTGIR | 1.862241793 | 5.81E-09 | 4.67E-07 |
| DACT1 | 1.861238631 | 3.26E-06 | 0.000128445 |
| ENPP4 | 1.85948549 | 0.001059369 | 0.016127988 |
| SAMD11 | 1.857245476 | 0.004349904 | 0.048026263 |
| AC007255.8 | 1.852708799 | 0.024675713 | 0.164339948 |
| RP11-399B17.1 | 1.851560136 | 0.002696545 | 0.032864886 |
| C1QTNF3 | 1.851442295 | 0.009906044 | 0.088054618 |
| RP11-227G15.12 | 1.849614135 | 0.033753763 | 0.200294845 |
| SLC1A2 | 1.848075252 | 0.000391366 | 0.007269921 |
| ATP10A | 1.84598534 | 2.29E-05 | 0.00069337 |
| RP11-46F15.2 | 1.845297788 | 0.041422647 | 0.229593235 |
| PAMR1 | 1.841002321 | 0.000919165 | 0.01435206 |
| HEY1 | 1.840752195 | 0.024137574 | 0.162382735 |
| IFI27 | 1.840234465 | 0.001826504 | 0.024462669 |
| RP11-11N9.4 | 1.838805902 | 0.000204861 | 0.004342259 |
| F8 | 1.837156958 | 3.64E-07 | 1.84E-05 |
| MT1M | 1.837156455 | 0.044368733 | 0.239744154 |
| NATD1 | 1.836424095 | 0.009217386 | 0.083454817 |
| KLF8 | 1.835930263 | 0.000241446 | 0.004997025 |
| CTD-2033D15.1 | 1.83393057 | 5.73E-05 | 0.001499565 |
| RP11-307C18.1 | 1.833335107 | 0.04454401 | 0.240454356 |
| ITGB2 | 1.832577026 | 0.00077226 | 0.01247558 |
| PODNL1 | 1.831672105 | 4.76E-05 | 0.00127962 |
| DNER | 1.831226872 | 0.020368322 | 0.144936831 |
| LMO2 | 1.830828851 | 0.028919864 | 0.181330921 |
| RP11-760H22.2 | 1.828812823 | 0.004913354 | 0.052477688 |
| HAPLN3 | 1.828309311 | 0.000871109 | 0.01371916 |
| GHDC | 1.828120092 | 0.036249362 | 0.210521657 |
| EPOP | 1.828000685 | 0.001075074 | 0.016298888 |
| LRP4 | 1.82293429 | 2.75E-05 | 0.000807411 |
| RP11-46C24.7 | 1.817343804 | 0.001804765 | 0.024245515 |
| AC144831.3 | 1.817160677 | 1.25E-06 | 5.50E-05 |
| LGALS9 | 1.814160379 | 0.046033052 | 0.245950583 |
| FBLN2 | 1.813105806 | 0.00034233 | 0.006536444 |
| EXTL1 | 1.813086216 | 0.04046126 | 0.225749971 |
| THBS2 | 1.81045547 | 0.008125817 | 0.076181275 |
| HOXB-AS2 | 1.809415964 | 0.011136609 | 0.095960302 |

| | | | |
|---|---|---|---|
| TBX18 | 1.808020061 | 4.93E-05 | 0.001318405 |
| KCTD12 | 1.805179594 | 0.002533321 | 0.031327579 |
| CYP11A1 | 1.803474721 | 0.000560959 | 0.009701252 |
| FLJ22447 | 1.795069139 | 0.000105875 | 0.002519173 |
| BACE2 | 1.791362339 | 0.000373028 | 0.007012422 |
| FOXD2-AS1 | 1.790671422 | 0.000852842 | 0.013469862 |
| A4GALT | 1.787640153 | 2.78E-12 | 4.52E-10 |
| GUCY1A2 | 1.785470017 | 6.32E-06 | 0.000228665 |
| LA16c-380F5.1 | 1.784954231 | 0.017814298 | 0.132702911 |
| FAM87B | 1.78338463 | 0.001756611 | 0.023714786 |
| C16orf45 | 1.782187255 | 1.24E-15 | 3.59E-13 |
| POLR2J3 | 1.780790923 | 8.65E-07 | 3.99E-05 |
| RP11-141C7.2 | 1.779725522 | 0.006525005 | 0.065423016 |
| TOX2 | 1.779419254 | 0.033984432 | 0.201119631 |
| RP11-134G8.5 | 1.778887755 | 0.005671596 | 0.058635004 |
| RP11-54O7.3 | 1.778189826 | 0.018518595 | 0.136466552 |
| TNS2 | 1.776300825 | 1.23E-06 | 5.42E-05 |
| SCD | 1.775888144 | 0.017645309 | 0.132071915 |
| SERPINE2 | 1.770901113 | 0.007784977 | 0.073902042 |
| SERTAD4 | 1.769927649 | 0.01659572 | 0.127033114 |
| SEMA3E | 1.767923194 | 0.04733837 | 0.2503067 |
| OLFM1 | 1.765500393 | 3.41E-05 | 0.000964526 |
| GLI1 | 1.757086587 | 0.001649185 | 0.022570073 |
| CYP7B1 | 1.756753456 | 0.003012326 | 0.035759964 |
| GLIS2 | 1.756620437 | 0.005552127 | 0.057644371 |
| CPED1 | 1.756522888 | 0.00044703 | 0.00808487 |
| GMNC | 1.753980933 | 0.03323799 | 0.198414971 |
| LINC01239 | 1.753095173 | 0.050389627 | 0.258887033 |
| XXbac-BPG248L24.12 | 1.750197733 | 0.009673863 | 0.086510676 |
| PCDHB8 | 1.749194631 | 0.045023683 | 0.242149931 |
| PCBP3 | 1.746646383 | 0.028064589 | 0.17770787 |
| FNDC5 | 1.744038811 | 0.000624057 | 0.01058821 |
| BEX4 | 1.741681572 | 0.013014146 | 0.107090896 |
| SLC25A27 | 1.74136076 | 0.013482741 | 0.109988099 |
| RNA5SP283 | 1.739021133 | 0.003905544 | 0.044256112 |
| PTPRB | 1.738982775 | 0.000731888 | 0.012000122 |
| LRRC17 | 1.734883844 | 0.014324423 | 0.114846846 |
| AC092614.2 | 1.734028834 | 0.033015368 | 0.197677713 |
| SRPX | 1.733777153 | 0.001128415 | 0.016851718 |
| LAMA1 | 1.733619282 | 0.048266776 | 0.25283585 |
| SETBP1 | 1.73305259 | 1.24E-05 | 0.00040903 |
| TENM3 | 1.732000586 | 3.63E-05 | 0.001014679 |
| HHIP-AS1 | 1.731012531 | 0.007686352 | 0.073468361 |
| ERFE | 1.73085082 | 2.92E-05 | 0.000849271 |
| RP11-100E13.1 | 1.730036988 | 0.001021283 | 0.015648182 |
| AC090587.5 | 1.728889398 | 0.014103791 | 0.113473793 |

| | | | |
|---|---|---|---|
| RP11-723O4.2 | 1.726948561 | 0.024443823 | 0.163384882 |
| MUC20 | 1.726184776 | 0.013167467 | 0.108068795 |
| POLR2J2 | 1.723850659 | 0.016896697 | 0.128351706 |
| UNC5B-AS1 | 1.722917956 | 0.04456332 | 0.240499415 |
| RORA | 1.721426662 | 1.87E-05 | 0.000582171 |
| TEX26-AS1 | 1.720675463 | 0.021197688 | 0.14843118 |
| PLAU | 1.717114887 | 1.39E-09 | 1.33E-07 |
| TPTE2P6 | 1.716489341 | 0.027004208 | 0.173477811 |
| FBLN7 | 1.715063469 | 2.32E-06 | 9.47E-05 |
| MOXD1 | 1.714598341 | 0.000754719 | 0.012291786 |
| ZNF395 | 1.713627332 | 2.69E-09 | 2.32E-07 |
| RP4-756G23.5 | 1.713493507 | 0.000355428 | 0.006721884 |
| NPL | 1.713385444 | 0.004462857 | 0.048928611 |
| KY | 1.712099864 | 0.030563105 | 0.1878659 |
| BACH2 | 1.711887005 | 0.007815115 | 0.074059604 |
| ANGPTL1 | 1.710839085 | 0.017484118 | 0.13128213 |
| FAM20C | 1.709117829 | 0.000390346 | 0.007269446 |
| TWIST2 | 1.706566156 | 3.34E-10 | 3.61E-08 |
| ENG | 1.706557489 | 0.000180449 | 0.003944158 |
| CNTN3 | 1.706544602 | 0.005075108 | 0.053795236 |
| OTUD1 | 1.705307377 | 0.001473983 | 0.020715076 |
| SLFN5 | 1.700224589 | 8.79E-06 | 0.00030456 |
| CTD-2033D15.3 | 1.697531585 | 0.006174504 | 0.06266466 |
| ICAM2 | 1.695331453 | 0.015834975 | 0.123099813 |
| ADM2 | 1.695063352 | 0.010144913 | 0.089669261 |
| CTD-2033D15.2 | 1.692955113 | 0.028539743 | 0.179451096 |
| AC108142.1 | 1.69273236 | 0.000438398 | 0.007981397 |
| SH3KBP1 | 1.692648852 | 1.25E-10 | 1.48E-08 |
| HS3ST3A1 | 1.690201066 | 8.43E-05 | 0.002075896 |
| LAMB3 | 1.689732891 | 1.78E-05 | 0.000560496 |
| RP11-810P12.7 | 1.688541272 | 0.019822481 | 0.142766115 |
| SLCO3A1 | 1.685523834 | 2.71E-06 | 0.000108467 |
| RP11-632L2.2 | 1.68263281 | 0.029866302 | 0.185296081 |
| NXPH4 | 1.682536212 | 0.023459238 | 0.159383327 |
| YPEL3 | 1.67981323 | 2.07E-08 | 1.46E-06 |
| RP11-1072A3.3 | 1.679110451 | 9.94E-05 | 0.002392828 |
| ZNF658B | 1.679079492 | 0.024233217 | 0.162577469 |
| PRRX1 | 1.677260477 | 9.20E-05 | 0.002245334 |
| FRMPD4 | 1.676908022 | 0.004824002 | 0.051699536 |
| PLBD1 | 1.674248296 | 5.71E-07 | 2.72E-05 |
| CASS4 | 1.669548496 | 0.038812519 | 0.219847419 |
| PNPLA3 | 1.669481947 | 0.019977554 | 0.143506087 |
| IL16 | 1.668903115 | 0.028359964 | 0.1787438 |
| NCF2 | 1.668343294 | 0.02127398 | 0.148525398 |
| IRS2 | 1.664999362 | 0.028005383 | 0.177669779 |
| KLHL13 | 1.655609569 | 0.002299503 | 0.029092557 |

| | | | |
|---|---|---|---|
| MIR497HG | 1.655023088 | 0.001524923 | 0.021271171 |
| NR1D1 | 1.654096061 | 0.00016436 | 0.003668083 |
| PABPC5 | 1.653648788 | 0.000439794 | 0.007993543 |
| ECM1 | 1.653335276 | 5.19E-06 | 0.000193029 |
| KIRREL3 | 1.651607972 | 0.000342398 | 0.006536444 |
| MGST1 | 1.647802197 | 3.18E-08 | 2.15E-06 |
| CTC-251D13.1 | 1.645785292 | 0.034549949 | 0.203532971 |
| CCDC80 | 1.643339049 | 0.000445782 | 0.008076084 |
| RP1-79C4.4 | 1.641251558 | 0.018382055 | 0.135688597 |
| CREG1 | 1.638539876 | 0.000173395 | 0.003834624 |
| IRF1 | 1.637985611 | 0.003793597 | 0.043188346 |
| MAP2 | 1.636619497 | 0.000165458 | 0.003688835 |
| PCDHB14 | 1.635086271 | 0.012261869 | 0.103104977 |
| MIR4767 | 1.635045217 | 0.015345011 | 0.120529485 |
| ARHGAP28 | 1.634073543 | 0.020391322 | 0.145053444 |
| GDF6 | 1.632628856 | 0.026669466 | 0.172385014 |
| PROS1 | 1.631600926 | 0.00046798 | 0.00834451 |
| PLSCR4 | 1.631485361 | 0.004432696 | 0.048670912 |
| RP11-203J24.8 | 1.627118395 | 0.006672894 | 0.066420119 |
| TBC1D8 | 1.623532633 | 9.32E-06 | 0.00032109 |
| TNFAIP8L3 | 1.622188326 | 9.22E-06 | 0.000318017 |
| PRICKLE1 | 1.620138726 | 0.003964214 | 0.044759102 |
| ARHGAP26 | 1.619922902 | 0.020917475 | 0.147457446 |
| CRACR2A | 1.61686687 | 1.51E-06 | 6.47E-05 |
| LINC00471 | 1.614956677 | 0.044036153 | 0.238378179 |
| DSP | 1.613448881 | 0.001533895 | 0.021365458 |
| MIR99AHG | 1.610954661 | 4.76E-05 | 0.00127962 |
| RP11-588H23.3 | 1.609879856 | 0.021206205 | 0.148443432 |
| UPK3BL | 1.608230215 | 7.02E-07 | 3.28E-05 |
| ZNF883 | 1.60624511 | 8.21E-05 | 0.002031291 |
| XAF1 | 1.605820091 | 0.000292754 | 0.005801669 |
| PBX4 | 1.604628194 | 0.021804564 | 0.15108924 |
| CCL28 | 1.602837346 | 0.050123409 | 0.257821183 |
| PIK3IP1 | 1.602826184 | 0.006118178 | 0.062280886 |
| CTD-2334D19.1 | 1.601993291 | 0.00457338 | 0.049940672 |
| SESN3 | 1.601515035 | 0.014881322 | 0.117985703 |
| FAP | 1.598189302 | 0.007860022 | 0.074292613 |
| CRYM | 1.595978009 | 0.041738638 | 0.23096899 |
| TREX2 | 1.593931779 | 0.011541691 | 0.09878973 |
| VCAN | 1.592929155 | 1.72E-06 | 7.23E-05 |
| NBL1 | 1.590715654 | 4.47E-07 | 2.19E-05 |
| COL1A1 | 1.590509067 | 0.000154891 | 0.003485133 |
| NUPR1 | 1.590200192 | 0.001878325 | 0.025049664 |
| LDHD | 1.588044468 | 0.001997472 | 0.026124745 |
| ZNF423 | 1.587255499 | 0.004768188 | 0.051402709 |
| RP11-522I20.3 | 1.586176255 | 0.000319902 | 0.006210628 |

| | | | |
|---|---|---|---|
| HYAL1 | 1.582232699 | 0.000749278 | 0.012223213 |
| PTPRG | 1.582030928 | 1.51E-06 | 6.47E-05 |
| OAF | 1.581980278 | 1.02E-06 | 4.64E-05 |
| GAA | 1.580964575 | 0.00973626 | 0.086968273 |
| MYO7B | 1.580683269 | 0.043593378 | 0.237249201 |
| RP11-1415C14.4 | 1.580004333 | 0.008359527 | 0.077642381 |
| CSF1R | 1.578410855 | 0.000186093 | 0.004014516 |
| RNASEH2B-AS1 | 1.5765835 | 0.037305557 | 0.214524066 |
| ETV1 | 1.575146453 | 0.000177179 | 0.00390257 |
| SPDYE2 | 1.574877303 | 0.046830291 | 0.248695938 |
| SNX18 | 1.573477051 | 7.37E-05 | 0.001854754 |
| FAM156A | 1.572083705 | 0.026659177 | 0.172369297 |
| YPEL2 | 1.569536125 | 0.009202278 | 0.083352425 |
| FBLN5 | 1.56487834 | 6.69E-05 | 0.001715522 |
| RP11-342D11.2 | 1.560743619 | 0.009633952 | 0.086335637 |
| RP11-875O11.1 | 1.558634327 | 0.017698733 | 0.132291244 |
| GRAMD4 | 1.558119887 | 0.000805241 | 0.012856898 |
| LTBP3 | 1.556972477 | 0.017419103 | 0.130992272 |
| DOK6 | 1.555297909 | 2.69E-06 | 0.000107771 |
| STXBP5-AS1 | 1.555005545 | 0.000357567 | 0.006756501 |
| RP11-482M8.1 | 1.553495229 | 0.021017977 | 0.147698332 |
| ALPL | 1.552518377 | 0.038212891 | 0.21780057 |
| DNM3OS | 1.552277395 | 5.73E-06 | 0.00020969 |
| AMZ1 | 1.550699102 | 0.00823991 | 0.076987714 |
| TEF | 1.549640359 | 5.77E-09 | 4.66E-07 |
| CPNE8 | 1.549293433 | 4.86E-08 | 3.15E-06 |
| CD248 | 1.545283881 | 0.001260988 | 0.018296007 |
| C1QTNF5 | 1.54373904 | 0.020190901 | 0.144423861 |
| AC000403.4 | 1.540954137 | 0.000345502 | 0.006573829 |
| PRCD | 1.5398009 | 0.009127956 | 0.082781773 |
| PPFIA4 | 1.536523729 | 0.037825282 | 0.216660847 |
| KCTD14 | 1.536357478 | 0.005270691 | 0.055430695 |
| ACSS3 | 1.536021327 | 0.00016316 | 0.003648734 |
| SLAMF8 | 1.531122774 | 0.030786707 | 0.188685782 |
| CMB9-22P13.1 | 1.530027807 | 0.000341761 | 0.006536444 |
| RP11-848P1.2 | 1.528970323 | 0.030054647 | 0.186148742 |
| TRANK1 | 1.525098273 | 0.000274596 | 0.005531763 |
| RP11-139H15.6 | 1.515088454 | 0.044006212 | 0.238378179 |
| KAZALD1 | 1.514051678 | 0.007355046 | 0.071269872 |
| SERINC2 | 1.513101586 | 0.001967968 | 0.025821347 |
| JHDM1D-AS1 | 1.512461902 | 0.001942381 | 0.025623539 |
| CREG2 | 1.512358464 | 0.031444511 | 0.191675981 |
| HOXB5 | 1.510844051 | 0.000260882 | 0.005328886 |
| HOXB2 | 1.510350406 | 2.43E-07 | 1.30E-05 |
| LGALS3 | 1.508999039 | 5.11E-09 | 4.18E-07 |
| NR4A1 | 1.508934025 | 0.023192325 | 0.157961261 |

| | | | |
|---|---|---|---|
| RP11-44N22.3 | 1.506108436 | 0.049285665 | 0.255778566 |
| AC125232.1 | 1.505437741 | 0.012325005 | 0.103358551 |
| C8orf46 | 1.502240719 | 0.008170626 | 0.076503287 |
| RP11-1252D15.1 | 1.499664132 | 0.027336749 | 0.174945626 |
| CHST11 | 1.498176473 | 4.75E-05 | 0.00127962 |
| PCDHB13 | 1.497653853 | 0.025662634 | 0.168055781 |
| ADAM22 | 1.496054383 | 0.003660061 | 0.041951109 |
| PRX | 1.495228217 | 0.002333192 | 0.029416995 |
| PDE4A | 1.492438311 | 6.07E-06 | 0.000220573 |
| CCDC3 | 1.492154497 | 0.019207244 | 0.139943052 |
| GPNMB | 1.488104693 | 0.02008068 | 0.143870006 |
| AF131217.1 | 1.486014446 | 0.044964474 | 0.241898024 |
| LNX1 | 1.485950141 | 0.000130547 | 0.0030127 |
| FN1 | 1.485350847 | 0.003651503 | 0.0418968 |
| LINC01301 | 1.483140551 | 0.00988033 | 0.087921408 |
| ADAMTS4 | 1.482384964 | 2.65E-05 | 0.000780154 |
| TMEM155 | 1.48108618 | 0.018563083 | 0.136656684 |
| FMN2 | 1.479236045 | 0.031701525 | 0.192663855 |
| PLEKHF1 | 1.477621137 | 0.002566351 | 0.03164733 |
| MIR503HG | 1.47292036 | 4.47E-05 | 0.001218111 |
| HS3ST3B1 | 1.472875155 | 0.007386662 | 0.071457432 |
| FLT3LG | 1.468589591 | 0.002170664 | 0.027913269 |
| PPIL6 | 1.466600469 | 0.023839769 | 0.161219748 |
| MN1 | 1.465959996 | 0.001345226 | 0.019301217 |
| ZCCHC14 | 1.46550258 | 0.002891115 | 0.034696549 |
| LINC01614 | 1.46340356 | 0.000876134 | 0.013762081 |
| FTCDNL1 | 1.463304146 | 0.003619962 | 0.041665647 |
| MYLK4 | 1.463276879 | 0.000452727 | 0.008147594 |
| RP11-318A15.2 | 1.462210822 | 0.035552345 | 0.207598441 |
| HOXA2 | 1.461245697 | 0.004425974 | 0.048621445 |
| LHFP | 1.455267168 | 7.42E-10 | 7.64E-08 |
| CTSF | 1.453715813 | 0.018420705 | 0.135927824 |
| CTSD | 1.453170201 | 0.00226685 | 0.028712562 |
| TRPS1 | 1.45253751 | 3.99E-05 | 0.001100635 |
| NME5 | 1.452056786 | 0.002945514 | 0.035158072 |
| PORCN | 1.451337197 | 0.010838402 | 0.094247623 |
| RP3-512B11.3 | 1.450049427 | 0.00045895 | 0.008232588 |
| RP11-631N16.2 | 1.449986123 | 0.017187601 | 0.129842147 |
| RP11-426C22.5 | 1.449879818 | 0.025018699 | 0.165440714 |
| HOMER2 | 1.448864201 | 0.001064187 | 0.016176604 |
| FMNL3 | 1.448453307 | 2.07E-08 | 1.46E-06 |
| ALDH1L2 | 1.448424111 | 1.21E-07 | 7.01E-06 |
| NPAS2 | 1.447553251 | 0.018681277 | 0.137158589 |
| SPHK1 | 1.442669106 | 0.000103361 | 0.00247007 |
| RP11-434E6.4 | 1.442194228 | 0.001556197 | 0.021560869 |
| IL3RA | 1.435569519 | 0.01931851 | 0.140305928 |

| | | | |
|---|---|---|---|
| CXXC5 | 1.435297689 | 6.67E-08 | 4.09E-06 |
| COL1A2 | 1.43356032 | 7.63E-06 | 0.000269438 |
| ITPRIPL2 | 1.433070563 | 9.64E-06 | 0.000329326 |
| CYP4V2 | 1.433020715 | 9.00E-07 | 4.13E-05 |
| DEPTOR | 1.431524052 | 0.039127337 | 0.221038592 |
| FBN1 | 1.430765528 | 0.005748175 | 0.05923131 |
| AZIN2 | 1.430375135 | 0.014624649 | 0.116498017 |
| ZMIZ1-AS1 | 1.430214453 | 0.026213565 | 0.170450846 |
| BCL2L11 | 1.429277073 | 0.033547526 | 0.199610965 |
| FCER1G | 1.427861327 | 0.004591703 | 0.050016277 |
| AK5 | 1.426536204 | 0.015161502 | 0.11955896 |
| PCDH7 | 1.424865812 | 0.005244604 | 0.055182788 |
| AKNA | 1.424292979 | 0.003957936 | 0.044734264 |
| DNM3 | 1.421570808 | 0.00707868 | 0.069637703 |
| TCN2 | 1.421362291 | 0.011101882 | 0.095736278 |
| FAM102B | 1.420975601 | 8.09E-05 | 0.002005289 |
| FRG1CP | 1.419010623 | 0.020247137 | 0.144496321 |
| SLC15A3 | 1.416959726 | 0.036960745 | 0.213099825 |
| PRKAR2A-AS1 | 1.416246174 | 0.010336889 | 0.090855114 |
| AC006978.6 | 1.415229954 | 0.006725395 | 0.066882012 |
| ITPR3 | 1.414939834 | 3.13E-09 | 2.66E-07 |
| RADIL | 1.412534815 | 0.000127487 | 0.002958223 |
| FAT2 | 1.410740883 | 0.003138414 | 0.036976652 |
| RP11-540A21.2 | 1.410663207 | 0.002601794 | 0.031994483 |
| FZD1 | 1.410649976 | 1.95E-10 | 2.22E-08 |
| ADAMTSL4 | 1.410013924 | 0.000148272 | 0.003353385 |
| CYP2A7 | 1.409343457 | 0.032896425 | 0.197347405 |
| GTF2IP9 | 1.408927076 | 0.032997653 | 0.197677713 |
| NFIX | 1.406829555 | 8.05E-06 | 0.000282216 |
| INPP4B | 1.402091632 | 0.001939187 | 0.0255968 |
| PCDHGB3 | 1.400989577 | 0.009605126 | 0.086200097 |
| HSD3B7 | 1.400280035 | 0.000244814 | 0.005052421 |
| KLF13 | 1.400092981 | 5.58E-10 | 5.89E-08 |
| C11orf52 | 1.399951909 | 0.042617745 | 0.234205432 |
| SMG7-AS1 | 1.398661094 | 0.013021004 | 0.107107159 |
| FIBCD1 | 1.397567768 | 1.07E-06 | 4.78E-05 |
| MRC2 | 1.397325661 | 0.001013679 | 0.015583812 |
| C14orf159 | 1.393825972 | 1.80E-05 | 0.000564167 |
| CDHR3 | 1.392202714 | 0.008885237 | 0.081150844 |
| RP5-1198O20.4 | 1.391145508 | 0.011626406 | 0.099245172 |
| LOXL3 | 1.389004543 | 0.000127563 | 0.002958223 |
| TIAM1 | 1.387772926 | 7.09E-05 | 0.001802904 |
| FLRT1 | 1.385606154 | 0.004340558 | 0.047947207 |
| PNRC1 | 1.385136209 | 0.005960171 | 0.061014576 |
| MSX1 | 1.383129699 | 6.73E-06 | 0.000241245 |
| ADGRA2 | 1.383085407 | 0.001890002 | 0.025144244 |

| | | | |
|---|---|---|---|
| KLHL24 | 1.382546161 | 0.026779824 | 0.172691291 |
| IFIT2 | 1.382480621 | 0.006669474 | 0.066416213 |
| SESN2 | 1.382433933 | 0.006261209 | 0.063386434 |
| ABCA8 | 1.380081652 | 0.034118799 | 0.20157054 |
| PSMB9 | 1.378827944 | 0.002217723 | 0.028286281 |
| CYP2E1 | 1.378477788 | 0.003592658 | 0.04139482 |
| DDR2 | 1.377822525 | 0.00018565 | 0.004012599 |
| LINC01116 | 1.376323758 | 1.99E-05 | 0.00061484 |
| ASAP3 | 1.374745402 | 0.000299072 | 0.005905819 |
| PCOLCE | 1.374405227 | 0.027729258 | 0.176530603 |
| TCP11L2 | 1.373352001 | 0.002222514 | 0.028330915 |
| KIAA1549L | 1.373008747 | 0.001313414 | 0.018953644 |
| MXI1 | 1.372463551 | 0.000263922 | 0.005375968 |
| PRR15 | 1.370802815 | 0.008168938 | 0.076503287 |
| MMP14 | 1.370078806 | 0.000522743 | 0.009152378 |
| CPM | 1.369165774 | 0.037265443 | 0.214405791 |
| CCT6B | 1.366878433 | 0.011983709 | 0.101348734 |
| PLXND1 | 1.366037394 | 1.75E-09 | 1.63E-07 |
| ANGPTL2 | 1.365336992 | 0.011740881 | 0.099662004 |
| AK4P1 | 1.36503287 | 0.033851583 | 0.200658206 |
| GPX7 | 1.364629951 | 0.004401241 | 0.048405903 |
| POPDC3 | 1.363008144 | 0.006571601 | 0.065769975 |
| TAF4B | 1.359861194 | 3.01E-05 | 0.000872376 |
| RP11-517I3.2 | 1.358269732 | 5.72E-05 | 0.001499565 |
| RASSF5 | 1.357622014 | 0.002476146 | 0.030777168 |
| GNAO1 | 1.357323688 | 0.027463975 | 0.175398795 |
| PPP1R3G | 1.356703355 | 0.00062074 | 0.010572823 |
| RP3-428L16.2 | 1.355652094 | 8.77E-05 | 0.002148125 |
| HOXD8 | 1.354290601 | 5.76E-06 | 0.000210706 |
| ANO7 | 1.354141898 | 0.011725161 | 0.099622998 |
| KIAA1456 | 1.352600421 | 0.01799409 | 0.133769684 |
| MAPK13 | 1.351754436 | 0.004929838 | 0.052602521 |
| ACSS2 | 1.350557382 | 0.035880711 | 0.208904202 |
| SOX9 | 1.348302908 | 0.007130244 | 0.069956746 |
| M1AP | 1.346884314 | 0.030102939 | 0.186383431 |
| ZNF658 | 1.346293311 | 0.00194661 | 0.025648485 |
| AMACR | 1.344081727 | 0.000463157 | 0.008287714 |
| RP11-134L10.1 | 1.34357792 | 0.007130056 | 0.069956746 |
| RPS28P7 | 1.343442826 | 0.014511775 | 0.115809136 |
| GFRA1 | 1.343141493 | 0.011169018 | 0.096201776 |
| WDR81 | 1.341493083 | 0.001069667 | 0.016228466 |
| PQLC2L | 1.340150827 | 0.021613869 | 0.150138325 |
| METRNL | 1.338239359 | 2.83E-08 | 1.95E-06 |
| LFNG | 1.33712651 | 0.000712073 | 0.011779757 |
| PARP15 | 1.333592285 | 0.011848422 | 0.100320603 |
| NR4A3 | 1.332372047 | 0.038200964 | 0.217789174 |

| | | | |
|---|---|---|---|
| GYPE | 1.328572116 | 0.002427655 | 0.030398344 |
| TACR2 | 1.328107867 | 0.002732295 | 0.03320836 |
| TSNARE1 | 1.326048578 | 0.014914774 | 0.118165514 |
| ASNS | 1.324607966 | 3.07E-05 | 0.000887546 |
| CALHM2 | 1.322557762 | 4.32E-07 | 2.13E-05 |
| KATNAL2 | 1.322205076 | 2.27E-06 | 9.30E-05 |
| RP11-848P1.9 | 1.320748686 | 0.00034094 | 0.006526649 |
| GALNT15 | 1.319541706 | 0.022734772 | 0.15556938 |
| RXRA | 1.318240481 | 1.86E-08 | 1.33E-06 |
| AC106786.1 | 1.318142586 | 0.009187013 | 0.083248528 |
| PSAT1 | 1.317382104 | 0.001729249 | 0.023446394 |
| AGAP9 | 1.315314574 | 0.033076447 | 0.19782745 |
| TIMP2 | 1.314870427 | 0.000895033 | 0.014015162 |
| ORAI3 | 1.313354938 | 0.024336878 | 0.162973878 |
| LINC01881 | 1.312220292 | 0.00153276 | 0.021363209 |
| SLC6A9 | 1.30782106 | 0.018984407 | 0.138734151 |
| SNHG5 | 1.307413214 | 0.004818343 | 0.051699536 |
| CUEDC1 | 1.306861678 | 0.000598901 | 0.010224665 |
| AC116366.6 | 1.305855246 | 0.023865819 | 0.161296471 |
| BOC | 1.305723357 | 0.004078948 | 0.045748441 |
| CBS | 1.305059371 | 0.001698145 | 0.023096032 |
| SNX9 | 1.302916141 | 3.37E-07 | 1.73E-05 |
| RP3-402G11.26 | 1.302449521 | 0.039924868 | 0.223783022 |
| PITPNC1 | 1.300402364 | 0.000334453 | 0.006422206 |
| SPATA13 | 1.298451845 | 0.017688731 | 0.132261545 |
| TTC39A | 1.297120743 | 0.00619633 | 0.062845624 |
| PITPNM3 | 1.294603373 | 0.021477215 | 0.149481959 |
| RP11-517P14.2 | 1.292431879 | 0.000534217 | 0.009316102 |
| ALS2CR11 | 1.292152148 | 0.020207874 | 0.144424273 |
| LYRM9 | 1.29183401 | 0.003505213 | 0.04062196 |
| CYBRD1 | 1.290318919 | 0.000318382 | 0.006186598 |
| CTD-2516F10.2 | 1.288661582 | 0.02562268 | 0.168044544 |
| MEGF8 | 1.288293495 | 0.02328845 | 0.158370125 |
| ZNF843 | 1.288025331 | 0.04426255 | 0.239288276 |
| PLCL1 | 1.28720154 | 0.008614717 | 0.079452136 |
| ZP3 | 1.286675909 | 0.037890333 | 0.216823244 |
| JDP2 | 1.286037669 | 0.000153695 | 0.003467669 |
| GADD45B | 1.284726835 | 0.000391169 | 0.007269921 |
| PLCL2 | 1.282944244 | 0.000204391 | 0.004336481 |
| CBLB | 1.282823888 | 0.001147504 | 0.017055514 |
| WASF3 | 1.279860819 | 0.00110105 | 0.016555748 |
| AIM1 | 1.278563557 | 0.000731161 | 0.011997158 |
| LRRK2 | 1.277937574 | 0.000112561 | 0.002660954 |
| RP11-147L13.15 | 1.273333074 | 0.039783045 | 0.223587204 |
| STEAP1B | 1.272079109 | 0.007343655 | 0.071190942 |
| MILR1 | 1.271075098 | 0.000747549 | 0.012211263 |

| | | | |
|---|---|---|---|
| DIRAS1 | 1.269380716 | 0.002747003 | 0.033294894 |
| STXBP5 | 1.268759854 | 9.45E-08 | 5.57E-06 |
| KCNG1 | 1.268144213 | 0.008325022 | 0.07755173 |
| ARHGEF6 | 1.267872101 | 0.000687366 | 0.011436086 |
| ZNRF2 | 1.265860867 | 0.000383083 | 0.007164592 |
| MXRA8 | 1.264700255 | 3.71E-06 | 0.000144129 |
| ADAMTS7 | 1.2645413 | 0.004906079 | 0.0524255 |
| KITLG | 1.262073304 | 0.017725364 | 0.132365287 |
| ZNF365 | 1.260830124 | 0.043036926 | 0.235154194 |
| LINC00899 | 1.256320141 | 0.037377267 | 0.214863232 |
| DHX58 | 1.255345435 | 0.009979919 | 0.088603585 |
| EIF4BP6 | 1.25378767 | 0.033623436 | 0.200008388 |
| CARD6 | 1.253460295 | 0.003753788 | 0.042890942 |
| XPOT | 1.253252032 | 6.06E-05 | 0.001566639 |
| NSMAF | 1.252570826 | 0.001343052 | 0.01928264 |
| RP11-54C4.3 | 1.25242566 | 0.008715906 | 0.080171714 |
| RP11-228B15.4 | 1.252365043 | 0.00974073 | 0.086972787 |
| BICC1 | 1.251826069 | 3.10E-05 | 0.000893732 |
| TSC22D1-AS1 | 1.251242065 | 0.028015426 | 0.177682111 |
| ARHGAP27 | 1.250140308 | 0.020798321 | 0.146900696 |
| HOXB6 | 1.249227207 | 0.000800508 | 0.012801386 |
| ID2-AS1 | 1.248923717 | 0.036536764 | 0.211656595 |
| RNF24 | 1.248495363 | 0.001147196 | 0.017055514 |
| PEX11G | 1.247171121 | 0.009893439 | 0.087978222 |
| SLC1A4 | 1.244854584 | 0.000495262 | 0.008755087 |
| CD68 | 1.24261955 | 0.000505969 | 0.008904677 |
| FAM110B | 1.241949189 | 0.002312455 | 0.029215932 |
| TRAPPC6A | 1.240647667 | 0.036397186 | 0.21117685 |
| SKI | 1.239539587 | 1.31E-05 | 0.000425467 |
| CCDC188 | 1.239090848 | 0.037499747 | 0.215358494 |
| BHLHE40 | 1.235413325 | 0.021224829 | 0.148489914 |
| ADGRD1 | 1.234009497 | 0.007713728 | 0.073639586 |
| PPP1R3B | 1.230352679 | 0.049505452 | 0.256083616 |
| SDHAP2 | 1.229443263 | 0.006282911 | 0.063541994 |
| PHEX | 1.229210991 | 0.046679438 | 0.24807498 |
| FGFR2 | 1.226617238 | 0.010603208 | 0.09245357 |
| RNF187 | 1.22523687 | 0.004211182 | 0.046895889 |
| SLC2A5 | 1.222526894 | 0.016042414 | 0.12414056 |
| CABLES1 | 1.222100511 | 0.000793334 | 0.01275965 |
| WNT5A | 1.221193093 | 0.015126544 | 0.119369108 |
| MDFIC | 1.219724493 | 5.38E-09 | 4.38E-07 |
| METTL7A | 1.218335213 | 0.021739641 | 0.150686963 |
| LINC00997 | 1.218202303 | 0.01001586 | 0.088743105 |
| CALB2 | 1.217484825 | 0.000330691 | 0.006363774 |
| CSF1 | 1.212173634 | 0.050517373 | 0.259149115 |
| HCG11 | 1.212129318 | 0.000232025 | 0.004824791 |

| | | | |
|---|---|---|---|
| SLC4A4 | 1.211303392 | 0.030368566 | 0.187352533 |
| ABTB1 | 1.210716141 | 2.60E-06 | 0.000104528 |
| ULK1 | 1.207515585 | 0.001109273 | 0.01663378 |
| ZNF503 | 1.2074873 | 2.97E-06 | 0.000118563 |
| FOXO4 | 1.206763457 | 0.013413433 | 0.109608745 |
| ZMIZ1 | 1.202996986 | 5.32E-06 | 0.000196133 |
| PLCG2 | 1.20005207 | 0.043635238 | 0.237359251 |
| OSBPL8 | 1.198488635 | 0.000254852 | 0.00523987 |
| BRI3 | 1.198224883 | 0.002610971 | 0.032071379 |
| ABHD14B | 1.19570726 | 0.000100806 | 0.002422221 |
| CAPS2 | 1.194542876 | 0.009590804 | 0.086195439 |
| B4GALT1-AS1 | 1.194176407 | 0.047752229 | 0.251280501 |
| DOCK10 | 1.192083589 | 0.000529855 | 0.009262112 |
| PDLIM2 | 1.191264005 | 2.58E-07 | 1.36E-05 |
| CLEC2D | 1.19064919 | 0.008469951 | 0.078468662 |
| OSER1-AS1 | 1.188999108 | 0.037203613 | 0.214162386 |
| TCEA1 | 1.186172998 | 0.000122841 | 0.002873003 |
| IER5L | 1.186026562 | 0.00140001 | 0.019905008 |
| MIR635 | 1.185020886 | 0.012829951 | 0.106016967 |
| NECAB2 | 1.184293289 | 0.026707602 | 0.17247906 |
| KLF11 | 1.181897547 | 0.000390035 | 0.007269446 |
| MOB3A | 1.1802674 | 1.71E-07 | 9.54E-06 |
| S1PR2 | 1.177434808 | 0.000372172 | 0.007002317 |
| ANKRD9 | 1.176847213 | 1.29E-06 | 5.63E-05 |
| ARHGAP6 | 1.174250478 | 0.023485947 | 0.159466017 |
| KDM7A | 1.173766682 | 2.02E-05 | 0.000622769 |
| CAPN3 | 1.172545985 | 0.036405819 | 0.21117685 |
| RP11-1398P2.1 | 1.171745773 | 0.04796817 | 0.251933378 |
| EML1 | 1.170457036 | 0.000623309 | 0.010587239 |
| CERCAM | 1.169177764 | 0.000136654 | 0.003126084 |
| CCBE1 | 1.168258283 | 0.033974024 | 0.20111229 |
| MTURN | 1.167958978 | 3.59E-05 | 0.001008264 |
| ABCC2 | 1.167231521 | 0.027845055 | 0.176908432 |
| PSAP | 1.165330612 | 0.048825474 | 0.254305139 |
| EFCAB6 | 1.164583006 | 0.011592087 | 0.099029286 |
| CTD-3035K23.7 | 1.163157665 | 0.002845535 | 0.034243195 |
| FZD7 | 1.157329299 | 0.004036627 | 0.045417909 |
| CD302 | 1.156928692 | 0.000194935 | 0.004172173 |
| CYTH3 | 1.156497631 | 4.06E-05 | 0.001115449 |
| KIAA1614 | 1.156414531 | 1.25E-06 | 5.50E-05 |
| EVA1A | 1.156049523 | 6.24E-05 | 0.00160702 |
| ATF4 | 1.155575867 | 0.002977671 | 0.035444459 |
| SLC25A34 | 1.153937446 | 0.025139598 | 0.165768709 |
| TPP1 | 1.153213914 | 0.013956611 | 0.112815084 |
| STAC3 | 1.152009021 | 0.012460542 | 0.104082199 |
| ARID5A | 1.151841304 | 0.000273391 | 0.005518423 |

| | | | |
|---|---|---|---|
| ADAMTS10 | 1.149764896 | 0.018150457 | 0.134431033 |
| EMP2 | 1.148792127 | 0.037946627 | 0.217015927 |
| SREBF1 | 1.148437284 | 0.030736803 | 0.18845835 |
| TIMP4 | 1.148303394 | 0.001451066 | 0.020458534 |
| CLTCL1 | 1.147992835 | 0.014524202 | 0.115856263 |
| FAM129A | 1.147450763 | 0.048346347 | 0.253131782 |
| LAYN | 1.145520492 | 0.024909012 | 0.165241581 |
| ZNF358 | 1.142802705 | 0.02981655 | 0.18508766 |
| TCF4 | 1.141563431 | 5.25E-05 | 0.00139577 |
| CPEB1 | 1.140423479 | 0.035893883 | 0.208925449 |
| RUNX1 | 1.137802219 | 0.000230713 | 0.004802073 |
| BTG1 | 1.137652459 | 0.005043569 | 0.053607469 |
| MGLL | 1.137294574 | 0.035515465 | 0.207579292 |
| FANK1 | 1.133481124 | 0.03665549 | 0.211840923 |
| CFAP69 | 1.130831546 | 0.015719276 | 0.122373842 |
| COL5A1 | 1.128466728 | 0.01688229 | 0.128351706 |
| CRTC3 | 1.126874823 | 0.001884296 | 0.025114031 |
| NPIPP1 | 1.126695889 | 1.91E-05 | 0.000592727 |
| PDGFRA | 1.124811316 | 0.003465182 | 0.040221778 |
| SERINC5 | 1.120961529 | 0.001019507 | 0.015640525 |
| TMC7 | 1.12074186 | 0.022116636 | 0.152576972 |
| ZCCHC24 | 1.119210984 | 0.001897401 | 0.025195325 |
| CDH13 | 1.117437743 | 0.007964574 | 0.07495788 |
| RGS17 | 1.116718526 | 0.044817269 | 0.241276377 |
| NR1D2 | 1.116506232 | 6.85E-06 | 0.000244646 |
| NID1 | 1.116313454 | 0.000654611 | 0.011012924 |
| ABCA2 | 1.116230325 | 0.033367249 | 0.199020377 |
| VEGFA | 1.115880262 | 0.044364484 | 0.239744154 |
| CPS1 | 1.111018931 | 0.013831426 | 0.112175163 |
| HOXB7 | 1.110996369 | 0.00063303 | 0.010690854 |
| RP11-705C15.3 | 1.109582796 | 0.001122094 | 0.016780162 |
| CTSL | 1.104211295 | 1.02E-07 | 5.95E-06 |
| PHGDH | 1.103008726 | 0.005214363 | 0.054943657 |
| SLFN11 | 1.102483544 | 0.00083414 | 0.013222091 |
| HOXB-AS1 | 1.102279673 | 0.01733671 | 0.130653638 |
| TSHZ3 | 1.101988356 | 3.80E-06 | 0.000147169 |
| ZNF667-AS1 | 1.101592143 | 0.009604582 | 0.086200097 |
| TMEM140 | 1.096353761 | 0.006281916 | 0.063541994 |
| BEST1 | 1.095905389 | 0.027522556 | 0.175665765 |
| SIX4 | 1.09325378 | 2.42E-05 | 0.000728695 |
| AC113189.5 | 1.091004331 | 0.008730477 | 0.080198507 |
| PIP5KL1 | 1.090202302 | 0.049766031 | 0.256870794 |
| TENM4 | 1.0887427 | 0.026081249 | 0.170016491 |
| EHBP1 | 1.088262802 | 5.66E-05 | 0.001486544 |
| ZHX2 | 1.086496422 | 0.005097095 | 0.053993269 |
| SDAD1P1 | 1.085161707 | 0.002793322 | 0.033725861 |

| | | | |
|---|---|---|---|
| RP11-395L14.18 | 1.07955584 | 0.027446397 | 0.175398795 |
| TRIM38 | 1.079477988 | 1.11E-05 | 0.000372319 |
| ANTXR1 | 1.078943957 | 4.64E-05 | 0.001254294 |
| SEPSECS | 1.078315132 | 0.000302795 | 0.005957586 |
| MSX2 | 1.077116226 | 0.035143296 | 0.206086599 |
| LHPP | 1.076306061 | 0.000182254 | 0.003962622 |
| LAMC1 | 1.074983948 | 0.006497373 | 0.065175748 |
| RALA | 1.072583118 | 3.77E-07 | 1.88E-05 |
| ASB13 | 1.072286662 | 0.000770587 | 0.012466923 |
| SFT2D2 | 1.070805881 | 0.000478553 | 0.008514594 |
| LRRC37A16P | 1.070395793 | 0.035683211 | 0.208136349 |
| FAM21EP | 1.070177629 | 0.048266084 | 0.25283585 |
| SIAE | 1.070082926 | 0.041661838 | 0.230707503 |
| LBX2 | 1.068573397 | 0.013364755 | 0.109401491 |
| TESK2 | 1.067765451 | 0.03573264 | 0.208318539 |
| PLA2G6 | 1.065705483 | 0.02578643 | 0.168765723 |
| RP11-426C22.4 | 1.063988909 | 0.047925708 | 0.251891276 |
| CTB-63M22.1 | 1.061090918 | 0.033929566 | 0.200957567 |
| FAM21FP | 1.060090636 | 0.042236941 | 0.23287358 |
| FAM196A | 1.05868028 | 0.022818167 | 0.156091346 |
| CD109 | 1.057931789 | 0.000126783 | 0.002952611 |
| EXOSC6 | 1.05766619 | 0.003213104 | 0.037714856 |
| B3GLCT | 1.056441207 | 0.000276237 | 0.005549532 |
| PLEKHH2 | 1.056412342 | 0.014989577 | 0.118458694 |
| FGFBP3 | 1.054477763 | 0.010843331 | 0.094247623 |
| VEGFB | 1.05405228 | 0.042961678 | 0.234982189 |
| CEBPB | 1.053748971 | 0.0466254 | 0.247968008 |
| EIF4BP3 | 1.052474801 | 0.045408493 | 0.243443678 |
| MAP2K5 | 1.052298905 | 0.03032693 | 0.187253639 |
| C4orf32 | 1.051753487 | 0.000727501 | 0.011946046 |
| TBC1D16 | 1.050556104 | 0.030511841 | 0.187761224 |
| CTD-2012J19.3 | 1.049583784 | 0.035520679 | 0.207579292 |
| OLFM2 | 1.048800577 | 0.032297702 | 0.194923517 |
| LIPC | 1.046218918 | 0.048417437 | 0.253322619 |
| HSPG2 | 1.046161123 | 0.047859337 | 0.251602714 |
| LRRC6 | 1.045241136 | 0.02531757 | 0.166741773 |
| MAMLD1 | 1.04296456 | 0.027828133 | 0.176908432 |
| ARHGAP31 | 1.041157518 | 0.000260356 | 0.00532311 |
| GRN | 1.040591326 | 0.031157498 | 0.190473963 |
| TTC39B | 1.040138441 | 8.16E-05 | 0.002020736 |
| LINC00649 | 1.039352648 | 0.04969122 | 0.256681418 |
| RPS6KA2 | 1.038940137 | 5.63E-05 | 0.001483617 |
| FAT1 | 1.03735547 | 0.020536827 | 0.145804824 |
| LINC00910 | 1.037239193 | 0.01395102 | 0.112811456 |
| ARNTL | 1.036968915 | 0.005680056 | 0.058694804 |
| XXyac-YX65C7_A.2 | 1.033804267 | 0.013292877 | 0.108975762 |

| | | | |
|---|---|---|---|
| LGALS3BP | 1.032856641 | 0.03781848 | 0.216660847 |
| CDK14 | 1.032547159 | 0.003551039 | 0.041044618 |
| LRP3 | 1.032542219 | 4.82E-07 | 2.34E-05 |
| PLBD2 | 1.032162215 | 0.021492609 | 0.14954166 |
| CHST15 | 1.032002108 | 1.38E-05 | 0.000446055 |
| TSC22D1 | 1.031725839 | 0.017767374 | 0.132533371 |
| ABC7-42389800N19.1 | 1.031293523 | 0.002359466 | 0.029728703 |
| ABTB2 | 1.027412888 | 9.65E-05 | 0.002330911 |
| RP11-317N8.5 | 1.026185557 | 0.024699054 | 0.164395582 |
| PIGBOS1 | 1.025526335 | 0.005380727 | 0.056157183 |
| TRIM25 | 1.024875042 | 0.003316042 | 0.038736603 |
| ADD3 | 1.024215319 | 0.017002067 | 0.128839843 |
| FAM83H | 1.023407134 | 0.015256046 | 0.120088676 |
| FADS3 | 1.023195345 | 0.035691902 | 0.208136349 |
| SUSD6 | 1.021234499 | 0.010894963 | 0.094546559 |
| OR2A1-AS1 | 1.018444573 | 0.002544002 | 0.031424732 |
| BVES | 1.017569327 | 0.002815829 | 0.03394157 |
| EVC2 | 1.016656807 | 0.004606399 | 0.050052091 |
| FTH1 | 1.015714209 | 0.03973934 | 0.223497223 |
| HDAC5 | 1.012966615 | 3.59E-08 | 2.38E-06 |
| MID1IP1 | 1.012416973 | 0.00035432 | 0.00670671 |
| WDFY3-AS2 | 1.011790237 | 0.003646368 | 0.041881689 |
| TK2 | 1.010598364 | 0.002764925 | 0.033449351 |
| KLHL35 | 1.010546961 | 0.012887489 | 0.106288086 |
| LDLRAD3 | 1.010139855 | 0.00117513 | 0.017360277 |
| RP11-412D9.4 | 1.009739175 | 0.022681803 | 0.155280501 |
| TMEM97 | 1.009445257 | 0.04468488 | 0.240782872 |
| DOCK11 | 1.008819607 | 0.020531373 | 0.145804824 |
| STEAP3 | 1.006349469 | 0.000144944 | 0.003298535 |
| KANK2 | 1.005373634 | 2.46E-05 | 0.000736797 |
| CCDC146 | 1.004956621 | 0.030446583 | 0.187517442 |
| HEXA | 1.004574742 | 0.01286496 | 0.106215085 |
| DIS3L | 1.002521152 | 0.00017292 | 0.003827977 |
| DCN | 1.002513254 | 0.002442671 | 0.030481976 |
| RCOR3 | 1.001685267 | 0.000141906 | 0.003239481 |
| SSH3 | -1.001055886 | 3.61E-05 | 0.001012603 |
| GNG2 | -1.001114028 | 0.026922446 | 0.173261095 |
| FAM86B1 | -1.002406166 | 0.043462884 | 0.236832777 |
| SHB | -1.002666802 | 0.000229134 | 0.004773744 |
| LPXN | -1.003379917 | 0.004657887 | 0.050486524 |
| SLC16A3 | -1.004614408 | 0.000735156 | 0.012035706 |
| ZNF678 | -1.004855708 | 0.017217196 | 0.130020946 |
| SMG1P6 | -1.005894756 | 0.0182368 | 0.134834957 |
| TAGLN2 | -1.005930426 | 0.014460918 | 0.115580951 |
| B4GALT5 | -1.008405451 | 0.001092549 | 0.016484414 |
| AMOT | -1.009067383 | 0.003356906 | 0.039130604 |

| | | | |
|---|---|---|---|
| CHSY1 | -1.010773908 | 0.000258388 | 0.00529768 |
| PON2 | -1.011469825 | 0.001205781 | 0.017717087 |
| PAPSS1 | -1.012100506 | 0.000275889 | 0.00554761 |
| RP4-613B23.1 | -1.013135254 | 0.046473237 | 0.247759388 |
| TRPV3 | -1.013675042 | 0.000984621 | 0.015254672 |
| PTOV1-AS1 | -1.017552363 | 5.09E-05 | 0.001357283 |
| CATSPER2 | -1.017735245 | 0.036913938 | 0.212941883 |
| NEXN-AS1 | -1.018276576 | 0.0494977 | 0.256083616 |
| PRR19 | -1.019649229 | 0.034693028 | 0.204211338 |
| SPEG | -1.020012154 | 0.00166895 | 0.022770226 |
| KLF5 | -1.020777988 | 0.012310785 | 0.103310512 |
| MBP | -1.021009599 | 0.04572079 | 0.244759076 |
| GSTM3 | -1.02107847 | 8.50E-05 | 0.002086926 |
| EFNA4 | -1.021192897 | 0.002726791 | 0.033159838 |
| SNRPN | -1.021464484 | 3.58E-09 | 3.01E-07 |
| SSFA2 | -1.022495232 | 0.001669 | 0.022770226 |
| PLB1 | -1.023896223 | 0.028510696 | 0.179416425 |
| CCDC50 | -1.026411388 | 0.000326322 | 0.006303494 |
| AC145124.2 | -1.02691751 | 0.023478064 | 0.159461848 |
| SAMD3 | -1.026950258 | 0.047458913 | 0.250381317 |
| KB-1732A1.1 | -1.028039322 | 0.028233037 | 0.178289107 |
| ITM2C | -1.029068699 | 1.61E-05 | 0.000511081 |
| SLC4A3 | -1.029172148 | 0.001523519 | 0.021271171 |
| MPP5 | -1.029336595 | 0.002748924 | 0.033299771 |
| SDF2L1 | -1.029821027 | 0.047733371 | 0.25124153 |
| NUAK2 | -1.030135769 | 0.00557508 | 0.057850781 |
| ARVCF | -1.031283439 | 0.03664182 | 0.211840923 |
| PRKCZ-AS1 | -1.031723141 | 0.020121223 | 0.144107278 |
| NOL6 | -1.032838499 | 0.000460912 | 0.008261016 |
| MAPRE2 | -1.033289527 | 0.000266663 | 0.005416711 |
| Sep-06 | -1.033539558 | 0.014551218 | 0.11599732 |
| KIF17 | -1.036197651 | 0.019084002 | 0.139276393 |
| SPRY3 | -1.038052258 | 0.00805086 | 0.075659759 |
| TMED5 | -1.040382246 | 3.12E-06 | 0.000123632 |
| PFKP | -1.042443525 | 0.036753674 | 0.212296499 |
| MGST2 | -1.043031098 | 0.033490986 | 0.199436819 |
| FNBP1L | -1.043455827 | 2.20E-05 | 0.000668936 |
| DCBLD1 | -1.043889367 | 0.001187593 | 0.017498617 |
| TRABD2A | -1.044217935 | 0.04105344 | 0.228007686 |
| TMEM35A | -1.044303128 | 0.034994469 | 0.205544487 |
| EID3 | -1.044834492 | 0.039899785 | 0.223783022 |
| ADAM15 | -1.04518112 | 0.000136173 | 0.003118325 |
| MPP6 | -1.045472181 | 0.000498897 | 0.00879808 |
| KBTBD8 | -1.052139327 | 0.037770387 | 0.216459446 |
| RN7SL471P | -1.053088878 | 0.01772674 | 0.132365287 |
| ABC7-42404400C24.1 | -1.053783347 | 0.006579841 | 0.065822411 |

| | | | |
|---|---|---|---|
| HMGN3 | -1.05700655 | 0.026055804 | 0.169920398 |
| FAM49B | -1.057053043 | 3.01E-05 | 0.000872376 |
| AGFG2 | -1.058271468 | 0.000172682 | 0.003826564 |
| BAG2 | -1.059028384 | 0.005128228 | 0.054270654 |
| RP11-490G2.2 | -1.059835712 | 0.03525532 | 0.206428548 |
| TNIK | -1.060315482 | 0.004969224 | 0.052942566 |
| PWAR6 | -1.061733451 | 0.002395393 | 0.030080213 |
| ULBP2 | -1.065620491 | 0.010392894 | 0.091117337 |
| ITGA4 | -1.066557248 | 0.002632968 | 0.032233286 |
| RP11-557N21.1 | -1.067733682 | 0.033422876 | 0.199139342 |
| GDAP1 | -1.068061276 | 0.028282724 | 0.178415634 |
| NEFH | -1.069094948 | 0.034420537 | 0.203043222 |
| PHLDA2 | -1.070257436 | 0.000452145 | 0.008147594 |
| MAP4K2 | -1.070272313 | 0.000240934 | 0.004991129 |
| DUSP1 | -1.072968932 | 0.050476042 | 0.259088302 |
| NACAD | -1.07471008 | 0.002091981 | 0.027156136 |
| WBSCR27 | -1.078800858 | 0.008483457 | 0.078560604 |
| FSCN1 | -1.079564281 | 0.011668463 | 0.099333621 |
| MET | -1.080853455 | 0.042680625 | 0.234374854 |
| PPME1 | -1.081668904 | 0.000512886 | 0.008994161 |
| SEMA3C | -1.082136422 | 0.007717422 | 0.073642801 |
| CTPS1 | -1.082943105 | 0.012413149 | 0.103819923 |
| BMPER | -1.085228715 | 0.021560021 | 0.149915606 |
| PICK1 | -1.085770724 | 4.37E-07 | 2.15E-05 |
| DISC1 | -1.087149341 | 0.002879963 | 0.034600566 |
| LINC01719 | -1.087764528 | 0.006348049 | 0.064058643 |
| ALDH3B1 | -1.091372474 | 1.10E-05 | 0.000369369 |
| TMEM102 | -1.091459259 | 0.038372104 | 0.218329717 |
| RP11-66B24.4 | -1.091864359 | 0.001283299 | 0.01858285 |
| AP1G2 | -1.09215537 | 0.014439629 | 0.115514702 |
| CHAC2 | -1.095222976 | 0.013653291 | 0.11093552 |
| FZD5 | -1.096084608 | 0.013138898 | 0.107915071 |
| SKP2 | -1.096863138 | 0.004477344 | 0.049038426 |
| PAPSS2 | -1.097157 | 1.75E-05 | 0.000553855 |
| MCC | -1.097211508 | 0.000821342 | 0.013056962 |
| GGH | -1.097342292 | 0.029706721 | 0.184619276 |
| RP11-714G18.1 | -1.097867053 | 0.028283186 | 0.178415634 |
| CCDC169 | -1.099176699 | 0.042806593 | 0.234714083 |
| BOLA2 | -1.099945396 | 0.006435563 | 0.064733325 |
| LRRC7 | -1.10010672 | 0.041346002 | 0.22934225 |
| HIST1H4I | -1.101317838 | 0.044039874 | 0.238378179 |
| HSP90AA2P | -1.101428839 | 0.042731343 | 0.234508874 |
| CTD-2587M2.1 | -1.101878566 | 0.019226583 | 0.140037446 |
| DPF1 | -1.102238298 | 0.035549421 | 0.207598441 |
| HIST1H2BK | -1.102435349 | 0.02766848 | 0.176297157 |
| RP11-259N19.1 | -1.104690098 | 0.002016179 | 0.026312271 |

| | | | |
|---|---|---|---|
| ARSJ | -1.107932502 | 0.012708698 | 0.105343893 |
| EFNA5 | -1.109163487 | 0.022680894 | 0.155280501 |
| LRRC37A15P | -1.10968662 | 0.018090762 | 0.134124754 |
| KIAA1549 | -1.10978565 | 0.009042581 | 0.08227961 |
| MICA | -1.110151691 | 0.002452365 | 0.030585551 |
| BZW2 | -1.110700763 | 0.000431316 | 0.007885181 |
| RALGPS2 | -1.112343749 | 0.031955466 | 0.193390072 |
| SERPINI1 | -1.113147981 | 0.038033554 | 0.217286485 |
| ETS2 | -1.113686995 | 0.002464685 | 0.030669466 |
| GAB3 | -1.114987145 | 0.000769967 | 0.012466074 |
| CNN1 | -1.115473585 | 0.016272086 | 0.125255097 |
| NECTIN2 | -1.117071376 | 8.24E-09 | 6.37E-07 |
| BST1 | -1.117814239 | 0.039821685 | 0.223715268 |
| CTD-2373N4.3 | -1.118877066 | 0.009607584 | 0.086200097 |
| CERKL | -1.11997982 | 0.018060147 | 0.134033663 |
| PRR34-AS1 | -1.126884272 | 0.019483435 | 0.141018672 |
| NBEAL2 | -1.129750497 | 2.82E-05 | 0.000827204 |
| DENND3 | -1.132160824 | 0.01742893 | 0.130992272 |
| RAB20 | -1.132572642 | 0.022535438 | 0.154687871 |
| RGS14 | -1.134828653 | 0.001100384 | 0.016555748 |
| HACD1 | -1.135080898 | 0.001853924 | 0.024769421 |
| PSPHP1 | -1.135192583 | 2.46E-05 | 0.000737633 |
| KRT17 | -1.136374664 | 0.003852521 | 0.043700417 |
| TLE4 | -1.136576496 | 1.77E-06 | 7.39E-05 |
| FBLIM1 | -1.138504425 | 0.000919118 | 0.01435206 |
| TNFAIP3 | -1.141571401 | 0.009673227 | 0.086510676 |
| ZNF714 | -1.142726008 | 0.016987558 | 0.128774378 |
| FAM213B | -1.143562357 | 2.63E-05 | 0.000777036 |
| ADAM9 | -1.14465296 | 2.56E-06 | 0.0001034 |
| SLC25A18 | -1.144985627 | 0.022367624 | 0.153921244 |
| PKN3 | -1.147203124 | 0.011789453 | 0.099937026 |
| FRK | -1.147216655 | 0.041722718 | 0.230964669 |
| TNFRSF21 | -1.147241183 | 0.029953417 | 0.185679026 |
| NBEA | -1.148159347 | 0.000244299 | 0.005046542 |
| SPOCD1 | -1.149249317 | 0.000623517 | 0.010587239 |
| SHTN1 | -1.149545605 | 2.26E-05 | 0.000683304 |
| NKILA | -1.154320387 | 0.011748261 | 0.099664873 |
| RP11-256L6.3 | -1.157463941 | 0.032035393 | 0.193766874 |
| RRP7A | -1.158681905 | 0.000193953 | 0.004155207 |
| ADAMTS15 | -1.159591486 | 0.009558916 | 0.085979294 |
| NDUFA12 | -1.16127079 | 0.000108269 | 0.002570568 |
| RAPGEFL1 | -1.162296461 | 0.006701854 | 0.066678128 |
| FAXC | -1.163161611 | 0.01255093 | 0.1045339 |
| GADD45A | -1.163771879 | 0.001707154 | 0.023204183 |
| THY1 | -1.164107694 | 3.27E-07 | 1.69E-05 |
| PPM1J | -1.165336868 | 0.025631509 | 0.168044544 |

| | | | |
|---|---|---|---|
| DOCK4 | -1.169946801 | 0.000183485 | 0.003973644 |
| HN1 | -1.17216958 | 0.009527438 | 0.085731315 |
| CDH24 | -1.172661537 | 0.013284213 | 0.108945447 |
| FABP5 | -1.173249527 | 0.012953799 | 0.10663431 |
| SH3RF3-AS1 | -1.173682038 | 0.032960427 | 0.197618433 |
| RP11-1149O23.3 | -1.176405334 | 0.049904719 | 0.257178698 |
| AC022007.5 | -1.179617368 | 0.004324895 | 0.047822352 |
| BFSP1 | -1.180456333 | 0.031924834 | 0.193311347 |
| PAPPA | -1.18258799 | 0.003655459 | 0.041920263 |
| CALM2 | -1.183457025 | 0.004315296 | 0.047822352 |
| TXNRD1 | -1.183769526 | 1.13E-05 | 0.000377558 |
| ODC1 | -1.184466024 | 0.009457639 | 0.085243089 |
| PRELID2 | -1.185738144 | 0.001238295 | 0.018067908 |
| TRO | -1.188182455 | 0.002386334 | 0.030000805 |
| PIP4K2C | -1.188832567 | 7.64E-10 | 7.83E-08 |
| LRRC3 | -1.189794592 | 0.000154882 | 0.003485133 |
| ACOT1 | -1.189838643 | 0.004741837 | 0.051168918 |
| PRR7 | -1.190048194 | 0.001403503 | 0.019941735 |
| EVA1C | -1.19012636 | 0.003363898 | 0.039191288 |
| AC005540.3 | -1.193723516 | 0.038588214 | 0.218972643 |
| TYW1B | -1.194352524 | 0.037395831 | 0.21487421 |
| CD9 | -1.19780065 | 0.035538378 | 0.207598441 |
| TMEM144 | -1.199056619 | 0.000756952 | 0.012318994 |
| CFH | -1.199968688 | 0.007812213 | 0.074059604 |
| FAM86B3P | -1.201970433 | 0.006423775 | 0.064674056 |
| RP11-3D4.3 | -1.20399942 | 0.024943827 | 0.165358018 |
| AFF2 | -1.210371381 | 0.00448996 | 0.049152069 |
| SLC29A1 | -1.210483613 | 7.31E-05 | 0.001844997 |
| ISOC1 | -1.211375045 | 3.63E-07 | 1.84E-05 |
| RAD18 | -1.212140956 | 0.011340491 | 0.09748734 |
| INPP5F | -1.213021907 | 9.42E-05 | 0.002283046 |
| FSIP2 | -1.215570597 | 0.050548395 | 0.259180979 |
| ACP6 | -1.216292055 | 0.004139017 | 0.046303804 |
| ITSN1 | -1.219446233 | 5.86E-08 | 3.64E-06 |
| ATP2C1 | -1.219548169 | 5.51E-11 | 6.91E-09 |
| TMEM2 | -1.220229496 | 2.94E-05 | 0.000853468 |
| LINC02210 | -1.223784374 | 1.16E-05 | 0.000386687 |
| PSEN2 | -1.22428976 | 3.14E-07 | 1.63E-05 |
| LINC01311 | -1.225460407 | 0.046588428 | 0.247845913 |
| MARCKSL1 | -1.226414539 | 0.000876362 | 0.013762081 |
| STX11 | -1.229674908 | 0.016137304 | 0.124435915 |
| ISG15 | -1.230329641 | 0.019288998 | 0.140305928 |
| CXCR6 | -1.231418924 | 0.039742122 | 0.223497223 |
| ATP11A | -1.236311727 | 1.00E-06 | 4.54E-05 |
| FAM86HP | -1.236948014 | 0.001809877 | 0.02428445 |
| LMO3 | -1.237719835 | 0.045037767 | 0.242166307 |

| | | | |
|---|---|---|---|
| CNKSR2 | -1.240740166 | 0.018077775 | 0.134073772 |
| C3orf35 | -1.241063262 | 0.049217557 | 0.255740112 |
| BHLHE41 | -1.242095482 | 0.008206348 | 0.076750699 |
| PANX2 | -1.243127663 | 1.83E-07 | 1.02E-05 |
| ANKRD34A | -1.244917044 | 3.16E-05 | 0.000908965 |
| MT-TH | -1.245193785 | 0.049275958 | 0.255778566 |
| NRXN3 | -1.246564853 | 0.018814025 | 0.137853651 |
| ZNF117 | -1.247624514 | 0.009755495 | 0.087069182 |
| HMOX2 | -1.249053873 | 2.21E-06 | 9.11E-05 |
| TES | -1.249614313 | 7.10E-06 | 0.000252719 |
| PLA2G4C | -1.250940558 | 0.00518251 | 0.054652687 |
| GRK3 | -1.251128841 | 2.01E-06 | 8.38E-05 |
| TRIM5 | -1.251753507 | 2.03E-07 | 1.10E-05 |
| LRP2BP | -1.251908996 | 0.000417602 | 0.007660001 |
| COL8A1 | -1.252102522 | 0.014999104 | 0.118491298 |
| STRIP2 | -1.252915979 | 0.002183477 | 0.02799465 |
| TMEM106A | -1.253438803 | 0.033127965 | 0.1980276 |
| IPO5P1 | -1.25398959 | 0.042842336 | 0.234851369 |
| RANBP17 | -1.2549512 | 9.36E-06 | 0.000321901 |
| CTD-3126B10.4 | -1.255282844 | 0.010009382 | 0.088721541 |
| FRY | -1.255730555 | 0.00180794 | 0.024273313 |
| NDRG4 | -1.256354527 | 0.001955292 | 0.025701135 |
| ERRFI1 | -1.256371097 | 0.011291194 | 0.09710161 |
| TAF7L | -1.258307676 | 0.028668317 | 0.180207892 |
| CTB-13F3.1 | -1.25860492 | 0.012933873 | 0.106510249 |
| FAM50B | -1.260502759 | 2.08E-07 | 1.12E-05 |
| FLNB-AS1 | -1.261704865 | 0.014846798 | 0.117754537 |
| HIST2H2BB | -1.263200011 | 0.008052946 | 0.075659759 |
| SNCAIP | -1.263850887 | 0.044640739 | 0.240655647 |
| FAM60A | -1.265701415 | 0.000280701 | 0.005623759 |
| KCNAB3 | -1.265731532 | 0.024080602 | 0.162248235 |
| PLS3 | -1.266225358 | 0.003427281 | 0.039887372 |
| RP11-989E6.13 | -1.266864539 | 0.01936017 | 0.140450864 |
| ATP10D | -1.267027861 | 3.10E-05 | 0.000893732 |
| LNCSRLR | -1.269443932 | 0.008582699 | 0.079212136 |
| MID1 | -1.270683138 | 0.004543784 | 0.049691693 |
| IVNS1ABP | -1.27446663 | 1.71E-08 | 1.23E-06 |
| RP4-584D14.5 | -1.274938581 | 0.029471358 | 0.183624726 |
| VAT1L | -1.276712163 | 0.016627018 | 0.127210598 |
| RP11-356I2.4 | -1.278291643 | 0.028502994 | 0.179416425 |
| THSD1 | -1.279359879 | 0.000893373 | 0.013999159 |
| FAXDC2 | -1.282774889 | 0.033108325 | 0.197964143 |
| TINAGL1 | -1.282925644 | 0.025137414 | 0.165768709 |
| TUBB2A | -1.283147678 | 0.001885776 | 0.025118489 |
| STXBP2 | -1.283163239 | 0.025445009 | 0.167421993 |
| PWAR5 | -1.283966733 | 0.020299861 | 0.144597558 |

| | | | |
|---|---|---|---|
| CAND2 | -1.284435211 | 0.015677025 | 0.122339405 |
| HPSE | -1.28519143 | 0.044615404 | 0.240602932 |
| SAMD10 | -1.286038862 | 0.002640487 | 0.032271317 |
| ANKLE1 | -1.286362695 | 0.022596195 | 0.155056401 |
| TGFB2 | -1.28789689 | 0.035512185 | 0.207579292 |
| PTK2B | -1.28860792 | 0.017099279 | 0.12926781 |
| LINC00854 | -1.291992091 | 0.026005559 | 0.169744108 |
| GATA6-AS1 | -1.292445195 | 0.014701921 | 0.11694371 |
| FUCA1 | -1.293527192 | 9.87E-05 | 0.002380339 |
| FAM198B | -1.294004732 | 0.001043314 | 0.01593154 |
| NRP2 | -1.296432753 | 0.016119871 | 0.124423126 |
| KLHL3 | -1.296705599 | 0.011478682 | 0.098423514 |
| RASL11A | -1.297342502 | 0.049421042 | 0.256009164 |
| CARD16 | -1.297401021 | 0.020519826 | 0.145778478 |
| NME9 | -1.297640472 | 0.02926774 | 0.182667334 |
| BDNF-AS | -1.29818899 | 0.001083653 | 0.016362464 |
| FTH1P16 | -1.300854869 | 0.039909163 | 0.223783022 |
| KIAA0895 | -1.302265934 | 0.007933066 | 0.074757558 |
| KRT80 | -1.304406887 | 0.033216964 | 0.198358285 |
| PLEKHH1 | -1.307126401 | 0.015043079 | 0.118795923 |
| HIST2H3PS2 | -1.307307044 | 0.013454944 | 0.10989373 |
| HOXA11 | -1.309632108 | 5.09E-06 | 0.000189932 |
| SFXN2 | -1.310579248 | 0.008270635 | 0.077143363 |
| S1PR3 | -1.311498851 | 0.013885545 | 0.112406309 |
| CCDC68 | -1.314569315 | 0.008827379 | 0.080858057 |
| MAMSTR | -1.31944496 | 0.022262333 | 0.153340995 |
| COBLL1 | -1.323254917 | 0.000326409 | 0.006303494 |
| INPP5J | -1.324391322 | 0.041973141 | 0.231937592 |
| SYT15 | -1.325536905 | 0.000109726 | 0.002602335 |
| PLOD2 | -1.326667074 | 0.000762473 | 0.012381304 |
| KALRN | -1.327857687 | 0.000383035 | 0.007164592 |
| RP11-448G15.3 | -1.330650688 | 0.015948558 | 0.123676024 |
| RAB3A | -1.330913177 | 0.002263396 | 0.028685375 |
| PSD | -1.332752894 | 0.018648955 | 0.137031128 |
| PSORS1C1 | -1.334126041 | 0.000561167 | 0.009701252 |
| CITF22-1A6.3 | -1.336471681 | 0.010469625 | 0.091565727 |
| LGALSL | -1.340419345 | 0.000126253 | 0.002943404 |
| FAM86FP | -1.343617046 | 0.00674271 | 0.066993465 |
| PLEK2 | -1.346636398 | 0.047321633 | 0.2503067 |
| RP11-549B18.1 | -1.348355736 | 0.005331891 | 0.055833423 |
| RP11-428J1.4 | -1.352580578 | 0.017589617 | 0.13183499 |
| PDCD1LG2 | -1.35374895 | 0.002304614 | 0.029140411 |
| KRT8P46 | -1.355213259 | 0.031727622 | 0.192755626 |
| AP000892.6 | -1.356709847 | 2.62E-09 | 2.27E-07 |
| ESPNL | -1.357963006 | 0.000385558 | 0.007198607 |
| TUBB3 | -1.359144635 | 0.039004158 | 0.220591185 |

| | | | |
|---|---|---|---|
| RP11-15H20.6 | -1.359277344 | 0.003628825 | 0.041731761 |
| JAM2 | -1.361332579 | 0.000718686 | 0.01184563 |
| HOXA11-AS | -1.36305191 | 0.003851785 | 0.043700417 |
| GATA6 | -1.364852575 | 0.017515431 | 0.131368728 |
| SEMA3B | -1.367293476 | 5.61E-05 | 0.001479912 |
| ASGR1 | -1.368131297 | 0.046668259 | 0.24807498 |
| SLFNL1-AS1 | -1.368284914 | 0.003089385 | 0.036496995 |
| PGM5P2 | -1.370086597 | 0.006667048 | 0.066416213 |
| DGCR11 | -1.371447011 | 0.007741917 | 0.073748233 |
| ANK1 | -1.37312817 | 0.007174208 | 0.070137154 |
| DISP2 | -1.373710565 | 0.0189508 | 0.138534704 |
| ZNF850 | -1.37560723 | 5.88E-05 | 0.00152634 |
| TNFRSF10A | -1.376295266 | 1.49E-06 | 6.41E-05 |
| CFAP46 | -1.377785973 | 0.021226382 | 0.148489914 |
| LL21NC02-1C16.2 | -1.378417892 | 0.016953193 | 0.12866032 |
| ZNF793 | -1.382350813 | 0.003771713 | 0.043050904 |
| SDC4 | -1.38666154 | 3.76E-05 | 0.001041901 |
| CYB5R2 | -1.389801472 | 5.98E-05 | 0.001551493 |
| JAZF1-AS1 | -1.39113722 | 0.049803165 | 0.25689674 |
| TOM1L1 | -1.391780233 | 0.000851484 | 0.013458112 |
| ZDHHC11 | -1.398384178 | 0.013483825 | 0.109988099 |
| RP11-298I3.4 | -1.399357869 | 0.011353574 | 0.0975418 |
| ADAMTS9 | -1.399724458 | 0.008384276 | 0.077839291 |
| AC015933.2 | -1.404460935 | 0.046348137 | 0.247191227 |
| NAP1L3 | -1.408812211 | 5.68E-05 | 0.001491052 |
| IFNE | -1.408933048 | 0.031505395 | 0.191937061 |
| TUBBP1 | -1.409446656 | 0.026733899 | 0.172547301 |
| FAM43A | -1.409948964 | 0.012331581 | 0.103374182 |
| RP11-185E8.2 | -1.41015897 | 0.010309693 | 0.090711789 |
| ERV3-1 | -1.410189107 | 0.001802432 | 0.024229018 |
| RNF217-AS1 | -1.413215214 | 0.009182688 | 0.083243722 |
| CCNB3 | -1.415542466 | 0.028896842 | 0.181330921 |
| C3orf70 | -1.423558136 | 0.002779315 | 0.033593721 |
| PPIL1 | -1.42476256 | 0.000182078 | 0.003962622 |
| GPC2 | -1.428050568 | 0.006475097 | 0.065011749 |
| ZNF439 | -1.431534835 | 4.08E-05 | 0.001116073 |
| RP11-395B7.4 | -1.433828261 | 0.020715446 | 0.146551258 |
| TSPAN9 | -1.436022681 | 0.000662648 | 0.011105562 |
| TIAM2 | -1.43629656 | 0.006599623 | 0.065990217 |
| RP1-140K8.5 | -1.440902764 | 0.003926805 | 0.044451105 |
| SLC9A3R2 | -1.441297742 | 0.000213088 | 0.004494933 |
| CDKL2 | -1.445160686 | 0.005161898 | 0.054495534 |
| PFKFB3 | -1.446044281 | 0.001166041 | 0.017249224 |
| MFSD2A | -1.449538954 | 9.87E-09 | 7.47E-07 |
| REEP2 | -1.450742697 | 0.00458661 | 0.049985616 |
| C11orf45 | -1.45225347 | 0.000299861 | 0.005910467 |

| | | | |
|---|---|---|---|
| JPH2 | -1.452605514 | 0.030791098 | 0.188685782 |
| KCNMB4 | -1.454884337 | 0.000410638 | 0.007562403 |
| CTD-2286N8.2 | -1.456136498 | 0.032318686 | 0.19496845 |
| SNHG14 | -1.456453437 | 3.74E-07 | 1.88E-05 |
| AC009487.6 | -1.45727441 | 0.02799912 | 0.177669779 |
| RDH10 | -1.457579132 | 0.01174338 | 0.099662004 |
| PRR5 | -1.457946768 | 1.76E-05 | 0.000555259 |
| TCAF2 | -1.459183009 | 0.000408932 | 0.007545118 |
| HOGA1 | -1.460470581 | 0.007065484 | 0.06957028 |
| SYNE2 | -1.460546477 | 0.000527133 | 0.009221886 |
| MSI2 | -1.465975785 | 2.49E-10 | 2.76E-08 |
| TUBB2B | -1.466457541 | 1.03E-06 | 4.65E-05 |
| PPP1R36 | -1.466773798 | 0.025021984 | 0.165440714 |
| TMEM182 | -1.471414306 | 3.25E-06 | 0.000128113 |
| SNHG25 | -1.475020611 | 0.023738502 | 0.160683513 |
| CDK15 | -1.47739319 | 1.62E-05 | 0.000514489 |
| CELSR2 | -1.478537172 | 0.017788594 | 0.132556444 |
| GLS | -1.481589826 | 4.09E-06 | 0.000157194 |
| TRIM24 | -1.48246398 | 1.51E-18 | 6.76E-16 |
| PLXNB3 | -1.485877309 | 0.000717827 | 0.011845399 |
| EMBP1 | -1.486614844 | 0.039858555 | 0.223783022 |
| ACVR1C | -1.490336108 | 0.044609596 | 0.240602932 |
| STK26 | -1.49071145 | 5.71E-08 | 3.60E-06 |
| LINC00472 | -1.494767877 | 0.002184649 | 0.02799465 |
| JCHAIN | -1.496048925 | 0.031815445 | 0.192983624 |
| MT-TR | -1.496069248 | 0.033170672 | 0.198174894 |
| CTXN1 | -1.496147052 | 0.018633224 | 0.137031128 |
| RP11-96L14.7 | -1.497638079 | 0.026146571 | 0.170161586 |
| RAB7B | -1.499090357 | 0.028745381 | 0.180588824 |
| ZNF711 | -1.499420744 | 0.00834039 | 0.077628971 |
| AP000648.5 | -1.499816763 | 5.34E-05 | 0.001418689 |
| BRSK2 | -1.500242884 | 0.049591283 | 0.256346269 |
| AAMDC | -1.500651042 | 6.38E-07 | 3.02E-05 |
| STAB1 | -1.503546451 | 0.028305708 | 0.178491127 |
| SLFNL1 | -1.504141082 | 0.003963716 | 0.044759102 |
| GALE | -1.505324726 | 1.87E-07 | 1.03E-05 |
| UTS2B | -1.505876295 | 0.035028565 | 0.205689685 |
| EAF2 | -1.505914213 | 0.001516421 | 0.021229889 |
| USP43 | -1.507096078 | 0.01751446 | 0.131368728 |
| OCIAD2 | -1.50751706 | 5.04E-06 | 0.000188453 |
| XDH | -1.507639735 | 0.002701321 | 0.032904821 |
| LMOD1 | -1.509374075 | 2.60E-05 | 0.000770518 |
| RHPN2 | -1.511972895 | 0.011373573 | 0.097618723 |
| RP4-635A23.6 | -1.514571204 | 0.016746617 | 0.127611131 |
| PPM1H | -1.516003476 | 0.007845826 | 0.074222396 |
| RP11-1149O23.2 | -1.516231216 | 0.008721688 | 0.080180846 |

| | | | |
|---|---|---|---|
| TRIM6 | -1.516589223 | 0.000281219 | 0.005629006 |
| LINC01775 | -1.518421691 | 0.046787084 | 0.248586836 |
| MDK | -1.51877418 | 0.002249018 | 0.02853612 |
| SGPP2 | -1.521123008 | 0.048711357 | 0.254133115 |
| KCTD16 | -1.521562147 | 0.011532991 | 0.09878973 |
| HIST1H1C | -1.527855091 | 0.039788697 | 0.223587204 |
| ARHGDIB | -1.528966104 | 0.000752185 | 0.012259617 |
| SERPINB9 | -1.532436838 | 0.006546697 | 0.065580565 |
| CCL20 | -1.53284223 | 0.025209086 | 0.166077153 |
| PGM2L1 | -1.532910081 | 0.00042232 | 0.007737492 |
| C10orf107 | -1.533540364 | 0.019663917 | 0.141950315 |
| LETM2 | -1.533964125 | 1.51E-06 | 6.47E-05 |
| LINC01679 | -1.534172343 | 0.009477691 | 0.085353686 |
| PIM2 | -1.536330545 | 0.000462983 | 0.008287714 |
| MEX3A | -1.536339564 | 0.000153799 | 0.003467669 |
| KIAA1324 | -1.537317674 | 3.38E-05 | 0.000958452 |
| ACTA2-AS1 | -1.538828225 | 0.014464116 | 0.115580951 |
| EPHA2 | -1.538970905 | 7.28E-12 | 1.08E-09 |
| DPP4 | -1.546243591 | 0.016847186 | 0.128242042 |
| WNT2B | -1.547426895 | 0.00018104 | 0.003949739 |
| HTR7 | -1.548747775 | 0.000540163 | 0.009404846 |
| FAM133A | -1.550696131 | 0.031703734 | 0.192663855 |
| VWA7 | -1.550881778 | 0.034029097 | 0.201221111 |
| NOS3 | -1.55637593 | 0.004870662 | 0.052123211 |
| STK32A | -1.557908719 | 0.036621567 | 0.211840923 |
| CFAP45 | -1.559415362 | 0.035643047 | 0.208017337 |
| GUCY1B3 | -1.559680774 | 0.016738841 | 0.127611131 |
| IL31RA | -1.560975989 | 0.002783061 | 0.033620475 |
| ZNF488 | -1.562401387 | 0.024121099 | 0.162339479 |
| ACTA2 | -1.564531189 | 0.020045575 | 0.143665412 |
| ATG9B | -1.565238075 | 0.044601978 | 0.240602932 |
| MMRN2 | -1.566027369 | 0.042138484 | 0.232445076 |
| RP11-77K12.9 | -1.569080462 | 0.017390014 | 0.130965369 |
| RP11-4B16.4 | -1.579558947 | 0.020037809 | 0.143661528 |
| NEK5 | -1.580750871 | 0.000269748 | 0.005459168 |
| RP11-274H2.5 | -1.582320158 | 0.007083683 | 0.069655692 |
| PERM1 | -1.582490017 | 0.040100054 | 0.224602904 |
| CENPV | -1.583162328 | 2.98E-07 | 1.56E-05 |
| PLEKHN1 | -1.585929846 | 0.022183434 | 0.152851182 |
| ADAM21 | -1.586964593 | 0.043252168 | 0.235918962 |
| CEP70 | -1.589575345 | 5.80E-08 | 3.63E-06 |
| GCNT1 | -1.595415689 | 0.003196477 | 0.03758002 |
| SHANK1 | -1.595941184 | 0.002181725 | 0.027989877 |
| ARL9 | -1.596476101 | 0.020000882 | 0.143597273 |
| RAB11FIP1 | -1.598400275 | 4.56E-05 | 0.001237575 |
| AKR1B1 | -1.59950008 | 1.79E-05 | 0.000562013 |

| | | | |
|---|---|---|---|
| SMAD3 | -1.599958404 | 7.14E-09 | 5.57E-07 |
| CRYZP1 | -1.599977206 | 0.031593844 | 0.192369069 |
| ZNF804A | -1.600338259 | 0.004602383 | 0.050052091 |
| ARHGEF28 | -1.600399504 | 0.004370244 | 0.048154492 |
| SLC14A1 | -1.601283195 | 0.014820454 | 0.117667999 |
| RAMP1 | -1.602829207 | 0.004188734 | 0.046695485 |
| ARFGEF3 | -1.604133556 | 0.001356324 | 0.019435029 |
| CSRP1 | -1.604151662 | 6.11E-05 | 0.001575677 |
| FAM46C | -1.605416802 | 0.002485217 | 0.03085495 |
| AC034220.3 | -1.607269372 | 0.007316809 | 0.070979581 |
| PKP3 | -1.616497435 | 0.012205031 | 0.102693317 |
| GBGT1 | -1.617107969 | 0.003738807 | 0.042742021 |
| PNMA2 | -1.617861998 | 0.000172224 | 0.00382027 |
| RP11-554A11.4 | -1.621311273 | 0.014079192 | 0.113429786 |
| PDCD6IPP2 | -1.623457696 | 0.000749395 | 0.012223213 |
| TNFSF13B | -1.626260546 | 0.002030721 | 0.026470557 |
| TGFB2-AS1 | -1.627378108 | 0.03914372 | 0.221038592 |
| LYN | -1.629743742 | 1.55E-06 | 6.55E-05 |
| PABPC4L | -1.630123261 | 4.25E-06 | 0.000161786 |
| EPHB6 | -1.631066209 | 0.024885082 | 0.16514896 |
| ST3GAL5 | -1.63672585 | 7.36E-06 | 0.000261334 |
| MECOM | -1.637006839 | 1.25E-09 | 1.21E-07 |
| MBNL3 | -1.639698443 | 2.09E-05 | 0.000639278 |
| TJP2 | -1.64148914 | 4.12E-07 | 2.04E-05 |
| GSTT2B | -1.643722695 | 0.004669584 | 0.050525225 |
| PCLO | -1.650957926 | 0.001672349 | 0.022801738 |
| HTRA4 | -1.653647087 | 0.025636726 | 0.168044544 |
| LMNTD2 | -1.65423254 | 0.02621484 | 0.170450846 |
| DNASE1L1 | -1.655279073 | 1.14E-08 | 8.48E-07 |
| ST8SIA4 | -1.665660295 | 0.049463498 | 0.256083616 |
| S100A2 | -1.666560043 | 0.008345194 | 0.077632498 |
| RP11-14D22.1 | -1.667636429 | 0.011692859 | 0.099479759 |
| SYNGR1 | -1.669855962 | 0.000227093 | 0.004735717 |
| RP11-392P7.6 | -1.670497907 | 5.91E-06 | 0.000215513 |
| OAS3 | -1.673738925 | 0.002901541 | 0.034783611 |
| FRMD4B | -1.67833666 | 0.039906688 | 0.223783022 |
| SH2D2A | -1.679119298 | 0.005325966 | 0.055806419 |
| TSPAN15 | -1.682446742 | 0.000556923 | 0.009650697 |
| C9orf47 | -1.684548856 | 0.026135287 | 0.170161586 |
| CTBP2P8 | -1.685923187 | 0.042537764 | 0.234117778 |
| FAM19A3 | -1.686768142 | 0.018918689 | 0.1383922 |
| TPD52L1 | -1.687859564 | 0.004058746 | 0.045568465 |
| LPAR3 | -1.693579021 | 0.020705999 | 0.14653417 |
| RP11-1072C15.6 | -1.696293536 | 0.005061398 | 0.053744891 |
| PDE10A | -1.698289448 | 0.000877008 | 0.013762379 |
| CDCP1 | -1.701078697 | 0.000795601 | 0.012786743 |

| | | | |
|---|---|---|---|
| ANOS1 | -1.70392867 | 0.042771035 | 0.234577741 |
| ABCA3 | -1.705878323 | 0.007781437 | 0.073902042 |
| RP11-169K17.4 | -1.706495345 | 0.024023456 | 0.16191293 |
| HIST1H3E | -1.708724665 | 0.00196264 | 0.025782279 |
| SLC25A4 | -1.708771959 | 2.12E-10 | 2.40E-08 |
| RP11-345P4.6 | -1.71082204 | 0.000159801 | 0.003584564 |
| PALM3 | -1.712296406 | 0.027559777 | 0.175808777 |
| AC009237.11 | -1.713582902 | 0.01372873 | 0.111424669 |
| RHOF | -1.713819341 | 2.94E-05 | 0.000853468 |
| C15orf48 | -1.714529556 | 0.006087715 | 0.062088467 |
| MMP24 | -1.716713465 | 0.000296568 | 0.005866636 |
| NHLH1 | -1.719357176 | 0.048862206 | 0.254375668 |
| RP1-102K2.9 | -1.719433216 | 0.026284375 | 0.17070584 |
| TNNT1 | -1.720806901 | 0.020993932 | 0.147698332 |
| PLEKHA2 | -1.720937133 | 1.39E-12 | 2.42E-10 |
| GMPR | -1.721517298 | 0.000305139 | 0.005987607 |
| ADCY2 | -1.722483124 | 0.025797381 | 0.168787042 |
| RP11-440D17.4 | -1.723933883 | 0.037718151 | 0.216329619 |
| MIR137HG | -1.725312456 | 0.000387294 | 0.007224885 |
| SARM1 | -1.727796497 | 1.57E-09 | 1.49E-07 |
| SYNPO2 | -1.73235241 | 0.000318139 | 0.006186598 |
| INTS6L | -1.733985336 | 1.34E-05 | 0.000434602 |
| PM20D2 | -1.734115198 | 3.35E-11 | 4.41E-09 |
| AQP1 | -1.738594165 | 0.016315329 | 0.125452455 |
| CEND1 | -1.740309877 | 9.34E-05 | 0.002269947 |
| CDRT1 | -1.742900207 | 0.015775729 | 0.122682716 |
| FAM86B2 | -1.744092156 | 0.026703757 | 0.17247906 |
| BAMBI | -1.745708196 | 0.000578362 | 0.009943648 |
| RPL13AP20 | -1.746553004 | 0.015709514 | 0.122371396 |
| FSD1 | -1.753510937 | 1.28E-06 | 5.59E-05 |
| FBXO41 | -1.754562803 | 8.03E-06 | 0.000281693 |
| SLC9C1 | -1.756546196 | 0.016381808 | 0.125746713 |
| IL27RA | -1.758041124 | 4.03E-05 | 0.001108671 |
| EPHB2 | -1.76440957 | 0.000823372 | 0.013079749 |
| FAR2 | -1.766108869 | 6.15E-08 | 3.80E-06 |
| HOXD9 | -1.767645401 | 1.74E-09 | 1.62E-07 |
| HHEX | -1.767779147 | 0.030432243 | 0.187501544 |
| SORD2P | -1.769082009 | 0.042864594 | 0.2348687 |
| ARHGEF37 | -1.770021859 | 0.000121574 | 0.002849455 |
| FILIP1L | -1.771745105 | 1.00E-05 | 0.000341212 |
| ACHE | -1.772599301 | 0.022839184 | 0.156186415 |
| IGF2BP1 | -1.772869037 | 0.002518242 | 0.031194345 |
| RP3-331H24.7 | -1.773161632 | 0.003348561 | 0.039054088 |
| RP11-981G7.6 | -1.776967547 | 0.041170678 | 0.228600945 |
| PIK3R3 | -1.777145336 | 1.43E-06 | 6.22E-05 |
| PITX2 | -1.777576127 | 4.05E-11 | 5.20E-09 |

| | | | |
|---|---|---|---|
| HEPHL1 | -1.78312728 | 0.016189618 | 0.124751613 |
| TRIM14 | -1.78375628 | 0.000353046 | 0.0066935 |
| RP11-109L13.1 | -1.784722665 | 0.016376155 | 0.125746713 |
| TRIM46 | -1.786860576 | 3.86E-08 | 2.55E-06 |
| LINC00968 | -1.792721843 | 0.005389961 | 0.056226797 |
| RP11-483P21.6 | -1.793559317 | 0.045097991 | 0.242371321 |
| LINC00310 | -1.793727658 | 0.021235752 | 0.148490692 |
| DGUOK-AS1 | -1.795896961 | 0.020956228 | 0.14763575 |
| ARHGEF19 | -1.797175842 | 0.000175883 | 0.00387792 |
| CDH18 | -1.798520452 | 0.012314568 | 0.103310512 |
| RP11-380J14.1 | -1.798849045 | 0.000810683 | 0.012934373 |
| RP11-70L8.5 | -1.799398592 | 0.002238252 | 0.028448878 |
| DFNA5 | -1.807437285 | 4.83E-05 | 0.001294627 |
| RP11-277P12.20 | -1.809678421 | 0.000547926 | 0.009517337 |
| ASRGL1 | -1.809786915 | 0.005075948 | 0.053795236 |
| NCKAP5 | -1.812617333 | 0.00280776 | 0.033862912 |
| UNC13D | -1.815560472 | 0.006777767 | 0.067280836 |
| SLC17A9 | -1.817744385 | 0.000284304 | 0.005678525 |
| S1PR5 | -1.820038035 | 1.71E-06 | 7.18E-05 |
| RP11-424I19.2 | -1.826598211 | 0.021005515 | 0.147698332 |
| GBP1 | -1.828764946 | 6.93E-05 | 0.001769939 |
| LAMA3 | -1.831665793 | 0.005496732 | 0.057177485 |
| TNFRSF11B | -1.83441945 | 0.006931758 | 0.068592199 |
| ARHGAP5-AS1 | -1.835828414 | 2.45E-09 | 2.16E-07 |
| KIAA1644 | -1.838440279 | 3.23E-05 | 0.000923739 |
| IGFBP7-AS1 | -1.840844379 | 0.03591288 | 0.208980571 |
| ITGB1BP2 | -1.84278221 | 0.006232477 | 0.06315472 |
| MB21D2 | -1.843312161 | 7.48E-08 | 4.51E-06 |
| PTPRQ | -1.843943689 | 0.000452509 | 0.008147594 |
| BNC1 | -1.84410664 | 0.000182794 | 0.003966498 |
| PALMD | -1.847105577 | 0.015524121 | 0.121318506 |
| RHOU | -1.850518176 | 7.25E-05 | 0.001833596 |
| CLUHP3 | -1.853418244 | 7.24E-05 | 0.001833596 |
| KLHL4 | -1.85446894 | 0.005292194 | 0.055550309 |
| GPR39 | -1.856382026 | 0.002174567 | 0.027914367 |
| CYFIP2 | -1.856519945 | 0.000222494 | 0.004666417 |
| CBX2 | -1.862898778 | 0.000410903 | 0.007562403 |
| DEF6 | -1.866339523 | 0.001076538 | 0.016298888 |
| RP11-229P13.25 | -1.868945288 | 0.039299156 | 0.221859207 |
| CCDC85C | -1.872330435 | 1.04E-10 | 1.26E-08 |
| NEBL | -1.872889817 | 0.007602946 | 0.073026898 |
| RP11-118F19.1 | -1.873882563 | 0.007491447 | 0.072240598 |
| CYP2B7P | -1.884881419 | 0.005684814 | 0.058716312 |
| RP11-320G24.1 | -1.888104559 | 0.006100501 | 0.062189955 |
| PTGS2 | -1.889799048 | 1.13E-06 | 5.06E-05 |
| HIST1H2BC | -1.892572061 | 0.022874036 | 0.156327287 |

| | | | |
|---|---|---|---|
| RAB27B | -1.89374082 | 0.000873426 | 0.013742566 |
| WFIKKN2 | -1.894921077 | 0.03561275 | 0.207895823 |
| SYNM | -1.899805128 | 3.07E-06 | 0.000122238 |
| SHC3 | -1.90268122 | 0.001612215 | 0.022147008 |
| ARHGAP4 | -1.911436949 | 0.001001436 | 0.015438355 |
| CREB5 | -1.914501124 | 2.43E-07 | 1.30E-05 |
| DRP2 | -1.9164444 | 1.44E-05 | 0.000463812 |
| AFP | -1.920545546 | 7.80E-05 | 0.001945732 |
| LXN | -1.922101051 | 0.000130089 | 0.003010424 |
| XXbac-BPG181B23.7 | -1.922525374 | 0.00044019 | 0.007994116 |
| CD274 | -1.925180945 | 3.42E-05 | 0.000964978 |
| MYCL | -1.925552666 | 8.22E-05 | 0.002031291 |
| AC008063.2 | -1.92630757 | 0.020658767 | 0.146528302 |
| HOXD-AS2 | -1.926805965 | 1.37E-09 | 1.32E-07 |
| AQP3 | -1.929421876 | 0.000554166 | 0.009610506 |
| ZNF114 | -1.932749785 | 0.0272596 | 0.174604704 |
| TLDC2 | -1.938832647 | 0.017752572 | 0.132497962 |
| APBA2 | -1.941927956 | 1.10E-16 | 3.88E-14 |
| SLC12A7 | -1.942999233 | 0.007522699 | 0.072478249 |
| EPN2-AS1 | -1.944275794 | 0.036428674 | 0.211253568 |
| RP11-649A18.4 | -1.946039953 | 0.001226449 | 0.017949186 |
| TM6SF2 | -1.950983981 | 5.16E-07 | 2.48E-05 |
| SLC27A6 | -1.951753479 | 0.021250276 | 0.148513798 |
| ITGA3 | -1.956782204 | 3.33E-07 | 1.71E-05 |
| RP11-159D12.3 | -1.95778276 | 0.033813317 | 0.200539753 |
| SLC15A2 | -1.960492327 | 0.000498504 | 0.00879808 |
| SLC2A1-AS1 | -1.964474499 | 0.004126898 | 0.046238959 |
| KRBOX1 | -1.968038727 | 0.006250425 | 0.063306473 |
| SERPINA1 | -1.970932899 | 0.011179882 | 0.096257554 |
| TMEM25 | -1.972364336 | 4.46E-11 | 5.69E-09 |
| PIK3AP1 | -1.972866119 | 0.000626998 | 0.010613495 |
| HMGA2 | -1.979498593 | 0.00013051 | 0.0030127 |
| SNORA71C | -1.982615958 | 0.042052519 | 0.232204419 |
| MIR3653 | -1.986865464 | 0.047176617 | 0.249809465 |
| SLC38A11 | -1.988794756 | 0.004604896 | 0.050052091 |
| HIST1H4H | -1.994069438 | 0.000331944 | 0.006382279 |
| RP11-379F4.4 | -1.994920794 | 0.019311127 | 0.140305928 |
| SOWAHD | -1.995080649 | 0.022075801 | 0.152443651 |
| STON1 | -1.997645076 | 1.05E-13 | 2.19E-11 |
| DIO2 | -1.998404326 | 0.002945598 | 0.035158072 |
| AP001505.10 | -2.011746563 | 0.007266724 | 0.070726438 |
| KRT18P34 | -2.015612921 | 0.002429545 | 0.030404653 |
| RPL7AP28 | -2.018555011 | 0.000282263 | 0.005644752 |
| BST2 | -2.018725957 | 9.63E-05 | 0.00232833 |
| AC090616.2 | -2.02153902 | 0.031033782 | 0.189907703 |
| LINC00327 | -2.024142661 | 0.002135548 | 0.027570761 |

| | | | |
|---|---|---|---|
| SLC9A3R1 | -2.025000441 | 3.44E-07 | 1.76E-05 |
| CARD10 | -2.025394204 | 0.00010512 | 0.002506664 |
| NIPA1 | -2.026059455 | 4.24E-09 | 3.54E-07 |
| CTC-296K1.4 | -2.026503077 | 0.008664451 | 0.079798792 |
| CSRNP3 | -2.027945586 | 5.84E-05 | 0.001518845 |
| ADAM19 | -2.028710642 | 7.06E-22 | 4.30E-19 |
| IFI6 | -2.031771047 | 0.002391151 | 0.030044137 |
| AC007362.3 | -2.032471017 | 0.0004131 | 0.007583752 |
| AMIGO2 | -2.038836021 | 7.84E-05 | 0.001951392 |
| CCND2 | -2.039222461 | 0.026727034 | 0.172547301 |
| BDNF | -2.040419426 | 0.00034422 | 0.00656081 |
| C4BPB | -2.040917636 | 0.010985866 | 0.095147226 |
| RP4-569M23.2 | -2.0420093 | 0.004243959 | 0.04716513 |
| FAM189A1 | -2.04481543 | 0.045518714 | 0.243822852 |
| LUZP2 | -2.047072874 | 0.008660765 | 0.079798348 |
| SNORD6 | -2.047159299 | 0.037893168 | 0.216823244 |
| RP11-757F18.5 | -2.049153079 | 0.000264886 | 0.005390605 |
| FOXE1 | -2.049166571 | 0.039733757 | 0.223497223 |
| ATP7B | -2.051483779 | 3.97E-09 | 3.32E-07 |
| RP11-701H24.5 | -2.055669666 | 0.02105503 | 0.147773945 |
| SOCS1 | -2.056823812 | 9.96E-06 | 0.000339238 |
| RAMP2 | -2.057555048 | 0.00695401 | 0.068781367 |
| IL11 | -2.064024743 | 4.55E-05 | 0.001237575 |
| CARNS1 | -2.071479654 | 0.000145116 | 0.003299015 |
| C3orf52 | -2.074143776 | 0.000422532 | 0.007737492 |
| LINC00920 | -2.082515278 | 0.006617915 | 0.066112855 |
| CSPG4 | -2.083075845 | 3.72E-07 | 1.87E-05 |
| BRWD1-AS2 | -2.086234813 | 0.024203783 | 0.162577469 |
| PEG10 | -2.091805501 | 2.49E-05 | 0.000745692 |
| SLC26A8 | -2.092867708 | 0.010857785 | 0.094330859 |
| TPPP | -2.095110359 | 0.00131498 | 0.018953644 |
| EFHC2 | -2.0966218 | 0.029201026 | 0.182406639 |
| RAC3 | -2.096779126 | 5.12E-07 | 2.47E-05 |
| GLIPR1 | -2.098641114 | 0.000138908 | 0.003174337 |
| MARK1 | -2.100598074 | 0.000180262 | 0.003944158 |
| GNA14 | -2.100634732 | 0.004713137 | 0.050924545 |
| TLR1 | -2.102587601 | 0.001628624 | 0.022333402 |
| TMEM255B | -2.105753591 | 3.36E-08 | 2.26E-06 |
| SAMD12 | -2.106581429 | 5.07E-05 | 0.001354742 |
| RBM47 | -2.110459745 | 1.62E-05 | 0.000514489 |
| OXTR | -2.111271843 | 0.000629023 | 0.010639559 |
| C22orf15 | -2.112901327 | 0.045819743 | 0.245228965 |
| GRIK2 | -2.113257079 | 2.11E-05 | 0.000644653 |
| MYOZ1 | -2.113873469 | 0.023686886 | 0.160532256 |
| RP5-1125A11.6 | -2.114429806 | 0.018053215 | 0.134033663 |
| RP11-428K3.1 | -2.117116513 | 0.01228614 | 0.10321462 |

| | | | |
|---|---|---|---|
| TIMM8AP1 | -2.118951901 | 0.001409487 | 0.020013806 |
| RRAD | -2.125809555 | 0.011250251 | 0.096825424 |
| MELTF | -2.127594558 | 2.39E-06 | 9.73E-05 |
| FHL1 | -2.130244537 | 0.000171899 | 0.003819052 |
| CELF2-AS1 | -2.132629685 | 0.011550536 | 0.09878973 |
| PHKA1 | -2.134699328 | 6.89E-09 | 5.40E-07 |
| RAB11FIP4 | -2.138870347 | 0.017659291 | 0.132131487 |
| LINC01828 | -2.143490597 | 0.030554849 | 0.1878659 |
| RP11-363E6.3 | -2.14561672 | 0.046355397 | 0.247191227 |
| GATA3 | -2.146882267 | 0.034124942 | 0.20157054 |
| CH17-360D5.2 | -2.149983176 | 0.012861415 | 0.106215085 |
| STXBP5L | -2.153666358 | 0.005145426 | 0.054350934 |
| PACSIN3 | -2.153846753 | 1.03E-10 | 1.25E-08 |
| PIK3C2B | -2.155986433 | 0.001129604 | 0.016857995 |
| RP11-210M15.2 | -2.164684733 | 0.023236216 | 0.158161992 |
| HNRNPA1P33 | -2.165743139 | 0.003651049 | 0.0418968 |
| DOCK2 | -2.168952922 | 0.00010746 | 0.002554133 |
| RP11-2E11.9 | -2.174606064 | 0.000530322 | 0.0092629 |
| ICOSLG | -2.174941398 | 0.003321487 | 0.038779552 |
| GTSE1-AS1 | -2.176254987 | 0.029145631 | 0.182291392 |
| MAGEL2 | -2.178578632 | 0.013574235 | 0.110538816 |
| GCSAM | -2.182390201 | 0.000268833 | 0.005445676 |
| RP1-102K2.8 | -2.183995892 | 0.00727353 | 0.070761291 |
| CAMK2N1 | -2.190990424 | 0.000189028 | 0.004069578 |
| SNTG2 | -2.197219751 | 0.000573074 | 0.009868208 |
| ADORA2B | -2.202103599 | 1.33E-12 | 2.33E-10 |
| EPCAM | -2.209146949 | 0.003826555 | 0.043518383 |
| PTGER2 | -2.212959305 | 0.011073325 | 0.095527563 |
| RNFT2 | -2.219878628 | 1.08E-05 | 0.000363933 |
| LYL1 | -2.224397448 | 6.03E-08 | 3.74E-06 |
| MLLT11 | -2.225519388 | 2.51E-09 | 2.20E-07 |
| PDE3B | -2.225859193 | 2.50E-05 | 0.00074673 |
| C1orf115 | -2.227994975 | 0.00132683 | 0.019074707 |
| ATP2B4 | -2.230475921 | 0.000507786 | 0.008926131 |
| RPS6KL1 | -2.232790712 | 0.000453254 | 0.008150397 |
| C1QTNF2 | -2.23802167 | 0.008263511 | 0.077109699 |
| EZR | -2.23972959 | 2.90E-06 | 0.000115968 |
| TRPM2 | -2.250537099 | 0.000635545 | 0.01072506 |
| VTN | -2.251322292 | 0.010123538 | 0.089552494 |
| FREM1 | -2.255590547 | 0.007206755 | 0.070392608 |
| PTHLH | -2.257608239 | 7.76E-05 | 0.001941827 |
| AC104667.3 | -2.259120078 | 0.020484238 | 0.145572792 |
| ANKEF1 | -2.265003654 | 1.80E-09 | 1.64E-07 |
| RP11-154D6.1 | -2.265216857 | 7.05E-05 | 0.001795277 |
| NPW | -2.267848081 | 0.018651424 | 0.137031128 |
| ICAM5 | -2.268220215 | 0.000797133 | 0.01279703 |

| | | | |
|---|---|---|---|
| OBSCN | -2.268834211 | 0.001003354 | 0.01544673 |
| MYEF2 | -2.279346744 | 1.97E-08 | 1.40E-06 |
| AREG | -2.291482502 | 0.039551484 | 0.222824978 |
| TMSB15A | -2.291699458 | 0.001369576 | 0.019586542 |
| LINC01776 | -2.294168225 | 0.001204665 | 0.017713096 |
| RPL36P4 | -2.294367729 | 0.000342643 | 0.006536444 |
| CCDC81 | -2.298603024 | 1.23E-05 | 0.00040693 |
| ADAM23 | -2.300620728 | 0.000147686 | 0.003347053 |
| RP11-583F2.2 | -2.31583898 | 0.036924538 | 0.212947036 |
| CTB-96E2.6 | -2.318033967 | 0.003585273 | 0.041331432 |
| CDH15 | -2.320744679 | 0.006656616 | 0.066378564 |
| RP11-130F10.1 | -2.323500511 | 0.047841232 | 0.251567823 |
| SYNGR2 | -2.323650755 | 1.54E-11 | 2.14E-09 |
| COL4A2-AS1 | -2.325916707 | 0.011113225 | 0.095796435 |
| RBM44 | -2.334695878 | 0.001506599 | 0.02110585 |
| PLEKHG3 | -2.337840913 | 2.59E-08 | 1.79E-06 |
| DOCK8 | -2.340215027 | 0.029027812 | 0.181842418 |
| TMEFF2 | -2.34325237 | 0.016254074 | 0.125160361 |
| CDC20P1 | -2.343922636 | 0.007318617 | 0.070979581 |
| SLC22A3 | -2.344810188 | 0.003410744 | 0.03971598 |
| TBC1D8-AS1 | -2.347635046 | 0.004368336 | 0.048154492 |
| KB-68A7.1 | -2.347708191 | 0.003939673 | 0.044573769 |
| GALNT3 | -2.349240004 | 0.004037055 | 0.045417909 |
| ERBB4 | -2.350298325 | 0.00031689 | 0.006168523 |
| RP4-758J24.6 | -2.350423802 | 0.023258288 | 0.158214052 |
| AP1M2 | -2.351466412 | 0.044965819 | 0.241898024 |
| TRHDE | -2.352756412 | 0.000467631 | 0.00834451 |
| SC22CB-1E7.1 | -2.35953175 | 0.004023604 | 0.045313048 |
| FSTL3 | -2.36436192 | 2.09E-13 | 4.21E-11 |
| HGF | -2.366000573 | 0.000571902 | 0.009855765 |
| POPDC2 | -2.366450067 | 0.002332729 | 0.029416995 |
| ADARB1 | -2.366911032 | 1.09E-09 | 1.08E-07 |
| APLP1 | -2.371486586 | 4.49E-07 | 2.19E-05 |
| RP11-496I9.1 | -2.37213538 | 0.012650817 | 0.105007046 |
| TRHDE-AS1 | -2.378004172 | 1.24E-05 | 0.000407648 |
| C3AR1 | -2.378444637 | 1.04E-05 | 0.000352804 |
| PPP1R14C | -2.381136903 | 0.008988854 | 0.081858643 |
| SORBS1 | -2.382749573 | 1.70E-07 | 9.51E-06 |
| LINC02057 | -2.388789529 | 0.04073828 | 0.226716488 |
| RP13-143G15.4 | -2.389023662 | 0.001585355 | 0.021873908 |
| FOXF1 | -2.392396801 | 4.73E-06 | 0.000178029 |
| NRGN | -2.394987618 | 0.00334405 | 0.039022218 |
| RP11-472B18.1 | -2.40124595 | 0.043674767 | 0.237456513 |
| SEMA3D | -2.40543405 | 6.86E-05 | 0.00175304 |
| SSTR1 | -2.406403395 | 7.69E-05 | 0.00192855 |
| SLC47A2 | -2.410624804 | 0.037884358 | 0.216823244 |

| | | | |
|---|---|---|---|
| HOXC11 | -2.411074751 | 1.23E-10 | 1.47E-08 |
| RASL10B | -2.413799246 | 2.61E-05 | 0.000774023 |
| PLK2 | -2.417472012 | 7.14E-13 | 1.33E-10 |
| PLS1 | -2.433184054 | 3.43E-06 | 0.000134597 |
| RP5-1063M23.3 | -2.435176583 | 0.000186105 | 0.004014516 |
| CHD7 | -2.435898195 | 1.10E-09 | 1.08E-07 |
| SPAG17 | -2.436347204 | 0.010687172 | 0.093111664 |
| EGLN3 | -2.442803567 | 0.017235032 | 0.130066092 |
| DIRAS3 | -2.443887833 | 1.29E-05 | 0.000421625 |
| E2F8 | -2.444852247 | 0.026086038 | 0.170016491 |
| NAALAD2 | -2.445210003 | 0.000116671 | 0.002748079 |
| ATP8B3 | -2.451269172 | 3.51E-08 | 2.34E-06 |
| RP11-61L23.2 | -2.460549025 | 0.011878961 | 0.100501597 |
| RP11-93B14.9 | -2.463755911 | 0.026963727 | 0.173367601 |
| FABP4 | -2.464973021 | 0.001505418 | 0.021102789 |
| RP11-404P21.3 | -2.4674723 | 0.015766238 | 0.122682716 |
| CDH20 | -2.480499788 | 0.004782577 | 0.051445666 |
| ST6GAL2 | -2.481955529 | 0.000490908 | 0.00870054 |
| RP11-650L12.2 | -2.483692644 | 0.019314528 | 0.140305928 |
| PPP4R4 | -2.492676661 | 0.020307461 | 0.144597558 |
| STK33 | -2.493456754 | 4.03E-08 | 2.65E-06 |
| ERC2 | -2.498790421 | 0.018729235 | 0.137372771 |
| TAGLN2P1 | -2.506641437 | 0.026258242 | 0.170631907 |
| EPHX4 | -2.523879814 | 0.011665394 | 0.099333621 |
| TRIM29 | -2.528657792 | 0.000124158 | 0.002900728 |
| AC092597.3 | -2.530643086 | 0.017243901 | 0.130088277 |
| SYNPO2L | -2.531229584 | 0.004074505 | 0.045721991 |
| PANCR | -2.534084476 | 0.007783629 | 0.073902042 |
| PROSER2-AS1 | -2.53793905 | 0.015412172 | 0.12079751 |
| HIST1H2BG | -2.538138848 | 0.002251694 | 0.028553561 |
| PDK4 | -2.538271143 | 0.038395298 | 0.218329717 |
| ICA1 | -2.540250797 | 0.007841443 | 0.074212932 |
| RSPO2 | -2.543089656 | 0.046968642 | 0.249309962 |
| TMEM14EP | -2.545582738 | 0.029066011 | 0.181977785 |
| AKAP5 | -2.547836846 | 3.99E-05 | 0.001100635 |
| OGFRL1 | -2.548720749 | 1.10E-24 | 8.04E-22 |
| RASAL3 | -2.548914383 | 0.008954655 | 0.081614969 |
| HES4 | -2.552186793 | 0.000328811 | 0.006333147 |
| RPS6KA1 | -2.554431505 | 3.11E-14 | 7.18E-12 |
| RAB17 | -2.560810826 | 0.000908958 | 0.014223047 |
| SYNPO | -2.562617681 | 9.56E-06 | 0.000327566 |
| DAPK1 | -2.563234519 | 2.21E-11 | 2.98E-09 |
| FUT1 | -2.571218986 | 0.036639885 | 0.211840923 |
| RP11-627G18.2 | -2.583420177 | 0.00011704 | 0.002752018 |
| CTB-96E2.10 | -2.584047539 | 0.005323133 | 0.055806419 |
| AC124789.1 | -2.591744485 | 2.15E-17 | 8.57E-15 |

| | | | |
|---|---|---|---|
| NEURL1B | -2.59263314 | 0.002069497 | 0.026928008 |
| PTGES3L | -2.593648391 | 0.000268399 | 0.005441892 |
| DNAH11 | -2.595852703 | 2.13E-07 | 1.15E-05 |
| RDH10-AS1 | -2.600165257 | 2.60E-05 | 0.000771797 |
| RARB | -2.608261687 | 2.31E-10 | 2.57E-08 |
| RP5-875H18.9 | -2.614013327 | 0.020696126 | 0.14653417 |
| HIST1H2AI | -2.62977749 | 0.043571252 | 0.237187625 |
| ARHGEF26 | -2.635191144 | 1.17E-12 | 2.08E-10 |
| RP11-798K3.2 | -2.640005911 | 0.008495838 | 0.078608898 |
| ITPRIPL1 | -2.643522465 | 0.005019703 | 0.053405548 |
| EPHA4 | -2.644069167 | 4.90E-14 | 1.08E-11 |
| CD74 | -2.646294605 | 1.81E-05 | 0.000564167 |
| SYT9 | -2.649593685 | 0.028373486 | 0.178764367 |
| CSNK2A3 | -2.652648508 | 0.000775282 | 0.012505986 |
| TNFRSF9 | -2.653939967 | 0.00019621 | 0.004187222 |
| HBEGF | -2.655449359 | 3.35E-07 | 1.72E-05 |
| SMCO3 | -2.665363225 | 0.00026564 | 0.005400938 |
| PKD1L1 | -2.675005095 | 8.52E-06 | 0.000297003 |
| RP1-118J21.5 | -2.678127692 | 0.002363221 | 0.029744318 |
| CTD-2314G24.2 | -2.689549031 | 0.039922322 | 0.223783022 |
| COLEC10 | -2.690013743 | 0.006636194 | 0.066235133 |
| SH3GL3 | -2.704762517 | 3.73E-05 | 0.001039233 |
| SOX6 | -2.706244 | 2.07E-08 | 1.46E-06 |
| HIST1H1PS1 | -2.706336526 | 0.012681003 | 0.105217789 |
| BDKRB1 | -2.708525765 | 9.01E-07 | 4.13E-05 |
| TMEM37 | -2.710650027 | 0.001598688 | 0.022002524 |
| RP11-82L18.2 | -2.710871021 | 8.00E-05 | 0.001988411 |
| RP11-90O23.1 | -2.717258109 | 0.002605384 | 0.032020679 |
| EPHA3 | -2.719536687 | 0.032337169 | 0.19496845 |
| GDF5 | -2.72228806 | 4.56E-05 | 0.001237575 |
| RN7SL589P | -2.725537471 | 0.0313129 | 0.191189092 |
| ADGRF4 | -2.728430426 | 0.000344637 | 0.00656307 |
| ATF4P4 | -2.728806037 | 0.00027931 | 0.005606126 |
| C10orf35 | -2.740094645 | 5.30E-06 | 0.000195814 |
| GTF2A1L | -2.750208568 | 1.46E-10 | 1.71E-08 |
| CRB1 | -2.754659309 | 0.021063627 | 0.147775455 |
| ANXA8L1 | -2.755998109 | 3.35E-12 | 5.23E-10 |
| ART4 | -2.765304703 | 0.0296946 | 0.184596238 |
| CDH10 | -2.771347059 | 4.93E-14 | 1.08E-11 |
| OGDHL | -2.771494905 | 0.005051184 | 0.05366241 |
| RP11-480I12.5 | -2.780718538 | 0.029996677 | 0.185842166 |
| SNORD116-24 | -2.781465457 | 0.0167327 | 0.127611131 |
| NTF3 | -2.782219288 | 0.000457152 | 0.008207045 |
| F12 | -2.78296023 | 0.00869885 | 0.080081988 |
| KCNS1 | -2.783051591 | 0.002174294 | 0.027914367 |
| HAPLN4 | -2.792867121 | 0.000873866 | 0.013742566 |

| | | | |
|---|---|---|---|
| DES | -2.802485245 | 0.005362081 | 0.056042559 |
| ANXA10 | -2.809513902 | 1.20E-06 | 5.33E-05 |
| AC078899.1 | -2.822791778 | 0.015849365 | 0.123168037 |
| TESMIN | -2.825176923 | 0.00104356 | 0.01593154 |
| RASSF7 | -2.827950571 | 2.51E-17 | 9.65E-15 |
| IGF2 | -2.82841327 | 2.17E-06 | 8.97E-05 |
| MPP7 | -2.833748323 | 3.40E-05 | 0.000963844 |
| MIR27B | -2.847051122 | 0.007096545 | 0.069750897 |
| IGSF5 | -2.849296362 | 0.005441659 | 0.056702338 |
| CTC-296K1.3 | -2.849574461 | 0.000259456 | 0.005314604 |
| SYTL5 | -2.859315281 | 0.000257174 | 0.005281945 |
| CDKL4 | -2.862192873 | 0.036254482 | 0.210521657 |
| CNTN6 | -2.865592484 | 0.023799253 | 0.16099538 |
| ANO2 | -2.874953874 | 0.032778039 | 0.196955524 |
| BCO2 | -2.875288492 | 4.06E-07 | 2.02E-05 |
| RN7SL68P | -2.881082562 | 0.03010953 | 0.186383431 |
| AF127577.13 | -2.88140936 | 0.005184258 | 0.054652687 |
| ELOVL2 | -2.883443218 | 4.58E-05 | 0.001240157 |
| AC023590.1 | -2.883584833 | 0.039060926 | 0.220798401 |
| DPYSL4 | -2.883761665 | 7.53E-19 | 3.84E-16 |
| RP11-380D23.1 | -2.886923534 | 0.031834667 | 0.192983624 |
| FCHO1 | -2.887416346 | 0.000994778 | 0.01537724 |
| LYPLAL1-AS1 | -2.8881422 | 0.014646062 | 0.11662625 |
| B3GALT5-AS1 | -2.889329956 | 0.018885243 | 0.138193613 |
| RGS2 | -2.89272233 | 1.56E-08 | 1.13E-06 |
| IGF2BP3 | -2.896781127 | 3.19E-05 | 0.000913365 |
| KLHL23 | -2.899012963 | 1.60E-08 | 1.16E-06 |
| RAB38 | -2.904088212 | 0.010212886 | 0.090052368 |
| AKAP6 | -2.904314421 | 4.49E-07 | 2.19E-05 |
| Mar-03 | -2.904649188 | 5.51E-11 | 6.91E-09 |
| FAM155A | -2.907946401 | 1.79E-09 | 1.64E-07 |
| MCHR1 | -2.916077306 | 0.016094465 | 0.124423126 |
| FAM134B | -2.920915092 | 0.000584881 | 0.010032148 |
| HIST1H3D | -2.927732052 | 0.02146365 | 0.149445305 |
| LIF | -2.936108564 | 0.000494058 | 0.008740846 |
| TM4SF18 | -2.939406674 | 0.025844681 | 0.168995708 |
| LRRC1 | -2.943301439 | 7.90E-30 | 9.63E-27 |
| ALDH1A3 | -2.943745364 | 1.89E-11 | 2.59E-09 |
| CYP2J2 | -2.947735794 | 0.024113976 | 0.162339479 |
| ADAP2 | -2.952524073 | 0.002233646 | 0.028429669 |
| SDK1 | -2.952759095 | 2.34E-07 | 1.26E-05 |
| TSPAN13 | -2.958792497 | 0.000353317 | 0.0066935 |
| CHRM2 | -2.965236408 | 0.000210634 | 0.004456019 |
| RP11-164P12.5 | -2.968464278 | 0.019413075 | 0.140695941 |
| CSRP2 | -2.96974349 | 8.95E-10 | 9.09E-08 |
| RP11-680F8.1 | -2.971652807 | 8.39E-05 | 0.002068486 |

| | | | |
|---|---|---|---|
| KNDC1 | -2.975201549 | 0.000225619 | 0.004713928 |
| KIT | -2.977829554 | 0.000679719 | 0.011348311 |
| CPNE5 | -2.986259696 | 0.001418783 | 0.020096673 |
| ASPHD1 | -3.004080923 | 2.41E-18 | 1.06E-15 |
| SORT1 | -3.014880131 | 2.79E-40 | 7.64E-37 |
| TMEM108 | -3.016103534 | 0.001567943 | 0.021688227 |
| WFDC21P | -3.023980209 | 1.41E-09 | 1.35E-07 |
| STRA6 | -3.024750377 | 6.09E-06 | 0.000220768 |
| RARRES1 | -3.025432726 | 0.019443636 | 0.140777061 |
| TRIM36 | -3.026260166 | 8.48E-08 | 5.07E-06 |
| AP1S3 | -3.028008608 | 5.50E-16 | 1.72E-13 |
| HAND2-AS1 | -3.02817216 | 2.98E-12 | 4.77E-10 |
| PTN | -3.030378009 | 1.42E-12 | 2.43E-10 |
| TYRP1 | -3.030489613 | 1.17E-07 | 6.81E-06 |
| ABCG2 | -3.044797027 | 8.15E-08 | 4.90E-06 |
| RP11-454P21.1 | -3.045263451 | 0.02363375 | 0.160320721 |
| NPTX1 | -3.045460641 | 0.003136656 | 0.036975798 |
| HOTAIR | -3.051530826 | 3.64E-17 | 1.38E-14 |
| LINC00426 | -3.055612222 | 0.005308841 | 0.055698399 |
| SEMA6A | -3.058543917 | 3.32E-10 | 3.61E-08 |
| RASGRF1 | -3.060848898 | 0.050031524 | 0.257590139 |
| LYPD6 | -3.064341935 | 4.54E-12 | 7.01E-10 |
| CORO1A | -3.074547587 | 0.000104582 | 0.002496547 |
| PYGM | -3.075670642 | 0.000842416 | 0.013332875 |
| INTS4P1 | -3.077841532 | 0.043897264 | 0.238193962 |
| CCNJL | -3.082971246 | 1.23E-12 | 2.17E-10 |
| RP11-567M16.2 | -3.093127223 | 0.0183821 | 0.135688597 |
| NYAP1 | -3.097049798 | 2.21E-10 | 2.48E-08 |
| RP11-145A3.1 | -3.103625984 | 0.00100823 | 0.015510909 |
| HTR1B | -3.105057978 | 2.29E-05 | 0.00069337 |
| SYT1 | -3.106081957 | 6.56E-08 | 4.03E-06 |
| ANKRD18B | -3.106475915 | 6.57E-09 | 5.18E-07 |
| ISYNA1 | -3.108512206 | 1.67E-07 | 9.39E-06 |
| RP11-79N23.1 | -3.109396536 | 0.020978571 | 0.147698295 |
| ARSE | -3.122993429 | 6.73E-13 | 1.27E-10 |
| HSD17B6 | -3.124647421 | 2.59E-08 | 1.79E-06 |
| KCTD4 | -3.127590982 | 0.001500023 | 0.021054062 |
| SULT1B1 | -3.12851421 | 7.76E-05 | 0.001941827 |
| CD80 | -3.135286755 | 0.027888673 | 0.177134249 |
| MAB21L2 | -3.139835228 | 0.042737099 | 0.234508874 |
| FAM221A | -3.145619212 | 1.02E-05 | 0.000346583 |
| RP11-161M6.6 | -3.161280303 | 0.000133113 | 0.003061032 |
| CDK18 | -3.165386539 | 0.001393298 | 0.019861066 |
| BIRC3 | -3.17145119 | 1.18E-07 | 6.83E-06 |
| C7orf69 | -3.17199455 | 0.001686862 | 0.022985328 |
| SH3RF2 | -3.172155191 | 4.98E-09 | 4.09E-07 |

| | | | |
|---|---|---|---|
| RASGRF2 | -3.175465388 | 3.76E-07 | 1.88E-05 |
| MYRFL | -3.17811145 | 0.006927035 | 0.068576395 |
| DBNDD1 | -3.183288617 | 6.52E-07 | 3.08E-05 |
| LRMP | -3.188438764 | 0.025341657 | 0.16685032 |
| BTBD11 | -3.189036259 | 2.99E-08 | 2.04E-06 |
| GARNL3 | -3.190498327 | 7.33E-06 | 0.000260493 |
| SCN5A | -3.193363916 | 0.013492667 | 0.109988099 |
| MOV10L1 | -3.194515622 | 1.54E-06 | 6.55E-05 |
| MDFI | -3.202068569 | 0.000147687 | 0.003347053 |
| AC012531.1 | -3.203125913 | 0.00495094 | 0.052802003 |
| LINC01778 | -3.20359688 | 0.014306817 | 0.114799906 |
| CHRNA5 | -3.206561032 | 1.13E-05 | 0.000377283 |
| TM4SF1 | -3.210639688 | 0.011760886 | 0.099733407 |
| RGS4 | -3.214289107 | 2.97E-09 | 2.54E-07 |
| IL1A | -3.219069537 | 9.71E-06 | 0.000331396 |
| LINC02056 | -3.226143706 | 0.007024785 | 0.069293944 |
| AC019181.3 | -3.227243205 | 0.028795696 | 0.180801367 |
| NDRG2 | -3.227725465 | 6.35E-06 | 0.000229563 |
| BX842568.2 | -3.236532088 | 0.001154205 | 0.017143496 |
| C1orf145 | -3.236761946 | 0.002244008 | 0.02850553 |
| VWDE | -3.241666729 | 0.003094027 | 0.036512518 |
| GALNTL6 | -3.249270906 | 0.045203127 | 0.242639149 |
| ARSI | -3.253197686 | 2.70E-15 | 7.06E-13 |
| CAMK2A | -3.25407795 | 1.94E-12 | 3.28E-10 |
| GCA | -3.256411331 | 4.58E-13 | 8.89E-11 |
| NEDD4L | -3.256722505 | 6.47E-15 | 1.63E-12 |
| HSPA2 | -3.257635412 | 7.23E-09 | 5.62E-07 |
| RP11-38L15.2 | -3.26175573 | 0.031161047 | 0.190473963 |
| PAQR9 | -3.271519624 | 0.004436096 | 0.048683875 |
| MYOCD | -3.276425181 | 6.84E-09 | 5.38E-07 |
| KRT18 | -3.280351728 | 1.39E-13 | 2.83E-11 |
| CH507-9B2.1 | -3.281422223 | 0.01709979 | 0.12926781 |
| SIM2 | -3.289198834 | 1.03E-09 | 1.03E-07 |
| GBP4 | -3.304026805 | 6.05E-05 | 0.001566639 |
| EPPK1 | -3.322181968 | 7.23E-05 | 0.001833596 |
| IGSF11 | -3.335766038 | 0.041819705 | 0.231209852 |
| CA3 | -3.342476015 | 0.002235444 | 0.028429669 |
| JUP | -3.342849725 | 5.34E-12 | 8.13E-10 |
| CTB-109A12.1 | -3.344593223 | 0.000445808 | 0.008076084 |
| WNK3 | -3.346579683 | 1.26E-15 | 3.59E-13 |
| ADGRL3 | -3.348047961 | 6.94E-08 | 4.23E-06 |
| EDARADD | -3.351784888 | 0.000161904 | 0.003624343 |
| HTR1D | -3.366855564 | 0.000384492 | 0.007184824 |
| RP11-168K11.3 | -3.37177136 | 0.000203939 | 0.00433322 |
| RP11-1090M7.1 | -3.372389918 | 0.032756677 | 0.196881089 |
| IL1RAPL1 | -3.376705382 | 0.001191544 | 0.017543685 |

| | | | |
|---|---|---|---|
| ARHGEF3 | -3.391116265 | 9.60E-12 | 1.40E-09 |
| NPM1P24 | -3.395394818 | 0.03692409 | NA |
| RP11-100G15.7 | -3.39642324 | 0.024978006 | 0.165391472 |
| PDGFRL | -3.40880966 | 3.17E-05 | 0.000908965 |
| HAND2 | -3.412979742 | 2.43E-11 | 3.25E-09 |
| LONRF2 | -3.421410978 | 0.000221712 | 0.004658924 |
| SH3TC1 | -3.430052471 | 6.90E-08 | 4.21E-06 |
| DUSP8 | -3.432503113 | 2.39E-07 | 1.28E-05 |
| GSTT2 | -3.439195725 | 0.001315434 | 0.018953644 |
| TOR4A | -3.443337198 | 7.65E-30 | 9.63E-27 |
| RP3-523E19.2 | -3.459005929 | 0.024451208 | 0.163384882 |
| RAB37 | -3.460524476 | 0.019298507 | 0.140305928 |
| FLRT3 | -3.462467288 | 1.09E-16 | 3.88E-14 |
| MCTP2 | -3.464403403 | 1.12E-09 | 1.09E-07 |
| RP11-883A18.3 | -3.467042155 | 0.034684289 | 0.204211338 |
| B4GALNT4 | -3.469841391 | 1.39E-08 | 1.03E-06 |
| LYPD6B | -3.470807157 | 3.18E-05 | 0.000910596 |
| ZNF185 | -3.474117534 | 2.07E-15 | 5.61E-13 |
| PARD6B | -3.477665309 | 0.002580707 | 0.031788633 |
| RP11-739P1.2 | -3.483481466 | 0.007754547 | 0.073836486 |
| KCNQ3 | -3.486663585 | 1.22E-11 | 1.74E-09 |
| PRSS35 | -3.496370395 | 1.31E-09 | 1.27E-07 |
| RP11-662B19.2 | -3.497029982 | 0.039419085 | NA |
| NPY4R | -3.498463541 | 0.000261358 | 0.005333647 |
| COL4A5 | -3.507615654 | 2.00E-09 | 1.79E-07 |
| FBXO2 | -3.50785442 | 1.76E-09 | 1.63E-07 |
| FAM90A25P | -3.524303046 | 0.047547113 | 0.250381317 |
| CTC-340A15.2 | -3.528142521 | 0.003276749 | 0.038359301 |
| SLC16A12 | -3.535529469 | 0.018257888 | 0.134907894 |
| RP5-998N21.7 | -3.535901116 | 0.024227497 | 0.162577469 |
| CEACAM22P | -3.541192579 | 0.044021545 | 0.238378179 |
| RGS5 | -3.544318171 | 6.95E-11 | 8.56E-09 |
| MGAT5B | -3.54957814 | 8.32E-08 | 4.98E-06 |
| AC027612.1 | -3.55186288 | 0.026802002 | 0.172783523 |
| TAGLN3 | -3.552022565 | 0.02129158 | 0.148525398 |
| PLCH2 | -3.55665681 | 0.001111398 | 0.01665427 |
| ADAMTS3 | -3.556847129 | 6.26E-16 | 1.91E-13 |
| PKP1 | -3.57043098 | 0.000718132 | 0.011845399 |
| HIST1H2BH | -3.571865673 | 0.004611473 | 0.05008242 |
| RP11-420K14.6 | -3.576228863 | 0.019610324 | 0.141610036 |
| DAW1 | -3.588847074 | 1.83E-09 | 1.66E-07 |
| PRG2 | -3.599546734 | 0.011004777 | 0.095160739 |
| LINC00900 | -3.601389244 | 0.005813264 | 0.059733667 |
| IGFBP2 | -3.607423291 | 8.95E-08 | 5.31E-06 |
| FILIP1 | -3.61051315 | 2.47E-16 | 8.10E-14 |
| LINC01629 | -3.618948154 | 0.008873784 | 0.08108 |

| | | | |
|---|---|---|---|
| MYZAP | -3.619099662 | 1.93E-09 | 1.74E-07 |
| BCHE | -3.62134273 | 2.66E-15 | 7.03E-13 |
| SLC16A9 | -3.623946869 | 7.40E-09 | 5.74E-07 |
| NFE2L3 | -3.62524251 | 5.32E-14 | 1.15E-11 |
| RP5-1074L1.1 | -3.630558232 | 0.014126184 | 0.113599792 |
| TFEC | -3.646755766 | 0.014937738 | 0.118262035 |
| HFM1 | -3.654595216 | 0.021888783 | 0.151433653 |
| FAM110C | -3.657179512 | 0.045149221 | 0.242527818 |
| RP11-642A1.1 | -3.658185811 | 0.032856291 | NA |
| APOA1 | -3.666894186 | 0.007551547 | 0.072692338 |
| SCIN | -3.667348002 | 9.53E-06 | 0.000327158 |
| RDM1 | -3.669908152 | 0.038966224 | 0.220490334 |
| ANO5 | -3.672577768 | 0.007592401 | 0.072989525 |
| CNTNAP2 | -3.676876998 | 6.35E-08 | 3.91E-06 |
| SPTBN2 | -3.677336795 | 1.03E-18 | 5.03E-16 |
| CARD11 | -3.68072788 | 1.96E-37 | 3.59E-34 |
| CHMP4C | -3.6822209 | 0.000597688 | 0.010211902 |
| MMP23A | -3.683816702 | 0.040367016 | NA |
| RP11-6O2.4 | -3.683888551 | 0.041185938 | NA |
| DPYD-AS1 | -3.684001241 | 0.018855302 | 0.138020558 |
| ASXL3 | -3.710529541 | 0.00094192 | 0.014676021 |
| GNG4 | -3.712170005 | 8.91E-05 | 0.002178774 |
| FGF9 | -3.720285641 | 0.02806809 | 0.17770787 |
| RPL37AP1 | -3.727267246 | 0.030152352 | NA |
| NELL2 | -3.728860641 | 0.020826478 | 0.146997004 |
| MIR30C2 | -3.729435522 | 0.029530043 | 0.183807429 |
| C1QL4 | -3.735160101 | 0.024472635 | 0.163384882 |
| SNX18P7 | -3.753995708 | 0.000132 | 0.003038639 |
| PDLIM3 | -3.755362991 | 7.04E-13 | 1.32E-10 |
| ANXA3 | -3.762441867 | 1.07E-23 | 7.33E-21 |
| COX6B2 | -3.766874778 | 0.006465537 | 0.064945487 |
| XKR5 | -3.78301361 | 0.000101506 | 0.002433698 |
| ST6GALNAC3 | -3.785136938 | 9.00E-14 | 1.90E-11 |
| F11R | -3.795742995 | 1.04E-06 | 4.68E-05 |
| MRPL9P1 | -3.80026868 | 0.038144232 | NA |
| PDIA2 | -3.803914248 | 0.011825387 | 0.100164223 |
| PPP2R2B | -3.810634769 | 0.001895344 | 0.025184777 |
| ERICH5 | -3.814004846 | 8.88E-09 | 6.76E-07 |
| MGARP | -3.818717497 | 3.54E-11 | 4.62E-09 |
| TCF7L1-IT1 | -3.820975667 | 0.02755715 | NA |
| ARMC4 | -3.842358876 | 6.70E-11 | 8.31E-09 |
| GLIS1 | -3.844915274 | 3.51E-30 | 4.81E-27 |
| RP4-564M11.2 | -3.850801705 | 0.000684872 | 0.011408288 |
| PDZRN4 | -3.852632634 | 0.00016364 | 0.003655738 |
| NIPAL4 | -3.853075048 | 0.000992482 | 0.015365606 |
| KLHL31 | -3.853840122 | 0.011255799 | 0.096835184 |

| | | | |
|---|---|---|---|
| IGSF3 | -3.857964035 | 3.88E-11 | 5.00E-09 |
| MATN2 | -3.85847559 | 5.91E-25 | 4.63E-22 |
| LINC01468 | -3.860488655 | 0.000224889 | 0.004703159 |
| SLC2A8 | -3.861862458 | 7.83E-12 | 1.15E-09 |
| CRISPLD1 | -3.899259235 | 6.08E-16 | 1.88E-13 |
| FRRS1L | -3.920552205 | 3.37E-05 | 0.000958348 |
| SEPT7P7 | -3.940842086 | 0.044807862 | NA |
| LANCL3 | -3.943873457 | 0.00146421 | 0.020617356 |
| MIR3192 | -3.947291869 | 0.023217262 | NA |
| GALNT9 | -3.963149813 | 5.20E-06 | 0.000193029 |
| RP11-22P4.2 | -3.9693589 | 0.045502532 | NA |
| TLL1 | -3.970440304 | 9.08E-08 | 5.37E-06 |
| SEL1L2 | -3.971696768 | 0.025557117 | 0.167865875 |
| MSLN | -3.98847945 | 0.005224037 | 0.055019165 |
| STOX2 | -4.012428613 | 2.89E-12 | 4.66E-10 |
| HID1 | -4.02772925 | 0.004566575 | 0.049891196 |
| LLGL2 | -4.034135659 | 1.27E-07 | 7.31E-06 |
| LINC00670 | -4.035555466 | 1.15E-06 | 5.11E-05 |
| RP11-408A13.3 | -4.037652628 | 0.040606097 | NA |
| HNRNPA1P68 | -4.038131056 | 0.029819535 | 0.18508766 |
| COL25A1 | -4.039778223 | 2.02E-09 | 1.81E-07 |
| RP5-1023B21.1 | -4.054133442 | 0.001395948 | 0.019862014 |
| CTD-2020K17.3 | -4.054851589 | 0.045247744 | NA |
| BMP4 | -4.05817266 | 8.12E-42 | 2.55E-38 |
| GALNT18 | -4.076684855 | 9.97E-10 | 1.00E-07 |
| RP4-671O14.7 | -4.085875464 | 0.022456538 | 0.154291117 |
| ANXA8 | -4.09322021 | 7.23E-15 | 1.80E-12 |
| HRASLS | -4.097393238 | 3.89E-08 | 2.57E-06 |
| RP11-54O7.18 | -4.102747594 | 0.015379032 | 0.120667101 |
| PTPN3 | -4.112170916 | 8.52E-14 | 1.82E-11 |
| TLR2 | -4.124385782 | 0.045274454 | 0.24290315 |
| LINGO2 | -4.125141551 | 0.01103369 | 0.095309409 |
| HNRNPA1P35 | -4.137376231 | 0.007065335 | 0.06957028 |
| ZDHHC11B | -4.142587618 | 0.031278516 | NA |
| PLEKHA7 | -4.143225743 | 2.08E-10 | 2.36E-08 |
| RP11-80H5.7 | -4.146545126 | 0.016570705 | 0.126885909 |
| LRRN3 | -4.147278316 | 7.98E-07 | 3.70E-05 |
| PGR | -4.149159167 | 0.006429352 | 0.064700519 |
| CCDC190 | -4.156575264 | 0.000209533 | 0.004437013 |
| VSIG1 | -4.161395899 | 0.008656955 | 0.07979676 |
| LINC01579 | -4.18119669 | 0.022047361 | 0.152338587 |
| RP11-739P1.3 | -4.188670064 | 0.004805645 | 0.051603641 |
| GSDMA | -4.194504714 | 0.003282814 | 0.0384098 |
| LRRN4 | -4.200831531 | 0.000800275 | 0.012801386 |
| OPTC | -4.203102836 | 0.032059247 | NA |
| GPR183 | -4.210289157 | 0.007300546 | 0.070954126 |

| | | | |
|---|---|---|---|
| BEGAIN | -4.210627735 | 6.20E-05 | 0.001598033 |
| RP11-164P12.4 | -4.224312327 | 0.024956714 | 0.165358018 |
| EGF | -4.227863575 | 5.95E-14 | 1.28E-11 |
| CYP4F26P | -4.229972031 | 5.13E-05 | 0.001367151 |
| AC073065.3 | -4.235860196 | 0.043130127 | NA |
| RP11-58A12.3 | -4.241617674 | 0.006130047 | 0.062317409 |
| DNAJC22 | -4.24946842 | 1.10E-11 | 1.57E-09 |
| SPINT1 | -4.255828187 | 0.002464435 | 0.030669466 |
| RFLNA | -4.257531635 | 3.89E-13 | 7.63E-11 |
| DHRS9 | -4.258328497 | 0.02211652 | NA |
| ABLIM2 | -4.258856791 | 0.006063633 | 0.061900405 |
| HMGB1P50 | -4.262571396 | 0.034547358 | NA |
| COL4A6 | -4.273412773 | 3.23E-08 | 2.17E-06 |
| RP11-381O6.1 | -4.277145356 | 0.000125366 | 0.002925823 |
| AC069542.1 | -4.290395576 | 0.010692201 | 0.0931185 |
| OOEP | -4.312320132 | 0.024169666 | 0.162548786 |
| FAM160A1 | -4.31265691 | 2.42E-09 | 2.14E-07 |
| MYBPHL | -4.314550312 | 0.028592173 | NA |
| KCNH2 | -4.327617802 | 0.004195149 | 0.046741076 |
| GPRC5A | -4.334739671 | 9.53E-28 | 8.71E-25 |
| C20orf141 | -4.336010693 | 0.017142388 | NA |
| ADD2 | -4.336127901 | 9.98E-05 | 0.002399993 |
| VCAM1 | -4.337974712 | 7.21E-08 | 4.38E-06 |
| RP11-65J3.14 | -4.359377413 | 0.026564593 | 0.171916486 |
| R3HDM2P1 | -4.363577879 | 0.048082388 | NA |
| RP11-566K19.6 | -4.380028902 | 9.45E-07 | 4.31E-05 |
| TPD52 | -4.38292901 | 8.60E-06 | 0.000299191 |
| IQGAP2 | -4.38586557 | 3.06E-14 | 7.13E-12 |
| RP11-346C4.3 | -4.397234089 | 1.55E-05 | 0.000495122 |
| RP11-285E9.6 | -4.413867082 | 0.007153536 | 0.070028677 |
| SLC15A1 | -4.415382902 | 0.047762722 | NA |
| GPR88 | -4.419429599 | 0.008923202 | 0.081395929 |
| RP11-63G10.4 | -4.421620731 | 0.016352751 | 0.12561157 |
| CDH8 | -4.43072131 | 0.000348623 | 0.006621718 |
| SOX11 | -4.431184226 | 3.13E-08 | 2.12E-06 |
| TTLL11-IT1 | -4.442173107 | 0.016107169 | 0.124423126 |
| DUX4L51 | -4.445365816 | 0.000366407 | 0.006899765 |
| DPY19L2P1 | -4.44653928 | 2.00E-12 | 3.36E-10 |
| JPH1 | -4.452712346 | 4.84E-09 | 3.99E-07 |
| C5orf46 | -4.464886517 | 1.98E-06 | 8.27E-05 |
| ATRNL1 | -4.465781545 | 0.000494054 | 0.008740846 |
| FAM189A2 | -4.476276442 | 0.00194595 | 0.025648485 |
| XK | -4.481590862 | 0.005855388 | 0.060053999 |
| LINC01515 | -4.48402855 | 1.84E-07 | 1.02E-05 |
| RP11-368L12.1 | -4.48423251 | 0.00214289 | 0.027637109 |
| SLC27A2 | -4.484969287 | 0.007303942 | NA |

| | | | |
|---|---|---|---|
| RP11-465B22.3 | -4.515619014 | 0.000135184 | 0.003098913 |
| HS6ST3 | -4.520422616 | 0.005477879 | 0.057008398 |
| PITX1 | -4.525972395 | 5.69E-33 | 8.33E-30 |
| GRIP1 | -4.537943737 | 1.09E-10 | 1.30E-08 |
| ZPLD1 | -4.538486629 | 0.007210515 | 0.070397985 |
| NIPAL1 | -4.554616423 | 1.93E-23 | 1.29E-20 |
| RP5-1011O1.2 | -4.557941598 | 0.004401941 | 0.048405903 |
| KIF5C | -4.559185976 | 0.000545918 | 0.009489969 |
| MIR7156 | -4.564083572 | 0.036837798 | NA |
| WFDC13 | -4.569765868 | 0.004532851 | NA |
| MAST4-AS1 | -4.571289043 | 0.003614229 | 0.041621502 |
| CCDC33 | -4.629204267 | 0.038573579 | 0.218946231 |
| CSMD3 | -4.635616634 | 0.003687872 | 0.042225754 |
| TMPRSS11B | -4.639142669 | 0.031525888 | NA |
| URAD | -4.639142669 | 0.031525888 | NA |
| NCAM1 | -4.646069425 | 6.75E-29 | 7.05E-26 |
| RPL5P1 | -4.647230768 | 0.002522696 | 0.031231886 |
| TMEM151A | -4.650604613 | 2.58E-09 | 2.24E-07 |
| C1orf226 | -4.67167819 | 5.85E-09 | 4.68E-07 |
| LRRC37A7P | -4.689535344 | 0.000801577 | 0.012807725 |
| C12orf54 | -4.699685224 | 0.023048277 | NA |
| PAK6 | -4.70215201 | 0.004188929 | 0.046695485 |
| BX842568.4 | -4.730301676 | 0.002585517 | 0.031830005 |
| THSD7A | -4.735327614 | 1.42E-09 | 1.35E-07 |
| EXPH5 | -4.745558151 | 3.26E-11 | 4.30E-09 |
| MAL2 | -4.752836365 | 0.001207359 | 0.017717087 |
| LINC01583 | -4.757221382 | 0.000193183 | 0.004142755 |
| CTC-327F10.4 | -4.761121766 | 0.016441933 | 0.126053705 |
| RP11-632K5.2 | -4.769467545 | 0.006346393 | 0.064058643 |
| PRND | -4.771335962 | 0.038962033 | NA |
| RP11-272L14.2 | -4.772745387 | 0.003510339 | 0.040659886 |
| IL1B | -4.790797126 | 9.95E-13 | 1.79E-10 |
| FLT1 | -4.796119233 | 8.45E-11 | 1.04E-08 |
| KRT8P3 | -4.805707887 | 1.27E-08 | 9.44E-07 |
| CNTN5 | -4.81023069 | 6.36E-10 | 6.64E-08 |
| NETO1 | -4.821837247 | 2.63E-05 | 0.000777036 |
| SLCO4A1 | -4.831350252 | 1.27E-06 | 5.55E-05 |
| SORL1 | -4.838776847 | 5.19E-15 | 1.34E-12 |
| SBK2 | -4.843644709 | 0.050399999 | NA |
| DSG2-AS1 | -4.846931279 | 0.007112781 | NA |
| AGR2 | -4.853624587 | 0.017605242 | NA |
| LINC01508 | -4.857536105 | 0.01771966 | NA |
| AC010974.3 | -4.870036252 | 0.006123615 | 0.062280886 |
| AC009238.8 | -4.893672849 | 0.00766219 | 0.073414147 |
| DOK2 | -4.896151971 | 5.15E-05 | 0.001371612 |
| HPSE2 | -4.898162522 | 0.018698409 | NA |

| | | | |
|---|---|---|---|
| FAM124B | -4.899252164 | 0.004714548 | 0.050924545 |
| TRBC2 | -4.943294291 | 8.96E-08 | 5.31E-06 |
| SOX17 | -4.947540331 | 0.001688025 | 0.022986902 |
| DUSP4 | -4.993583408 | 1.69E-15 | 4.68E-13 |
| ADAMTS19 | -4.998390227 | 0.000509864 | 0.008955477 |
| AC012531.2 | -5.009971062 | 0.000723953 | 0.011905609 |
| RP11-148L24.1 | -5.032295312 | 0.000578138 | 0.009943648 |
| RP11-95G6.1 | -5.033628442 | 0.010267117 | 0.09042152 |
| GABRB2 | -5.039407152 | 0.000819448 | 0.013036294 |
| ACTBL2 | -5.053347393 | 1.56E-08 | 1.14E-06 |
| RP1-170O19.14 | -5.054543643 | 5.60E-06 | 0.00020561 |
| LINC00520 | -5.059026638 | 0.000541118 | 0.009413988 |
| PADI1 | -5.064447564 | 1.71E-10 | 1.98E-08 |
| L1CAM | -5.07146802 | 4.54E-05 | 0.001236991 |
| PRKCB | -5.07346141 | 0.002504878 | 0.031063884 |
| CNGA1 | -5.078366904 | 0.00064887 | 0.010930845 |
| TXK | -5.085238808 | 0.000997259 | 0.015385287 |
| EFNB2 | -5.099870671 | 5.93E-61 | 6.51E-57 |
| RP11-438B23.2 | -5.104587786 | 0.021454342 | 0.149445305 |
| AP000697.6 | -5.109254731 | 0.00628596 | 0.063541994 |
| CDH7 | -5.118934997 | 0.000490987 | 0.00870054 |
| GDF5OS | -5.119784587 | 0.001021433 | 0.015648182 |
| KB-1269D1.8 | -5.131710406 | 0.022668428 | 0.155280501 |
| SAMD12-AS1 | -5.150056799 | 0.001163285 | 0.017231692 |
| Dec-01 | -5.150541243 | 5.79E-05 | 0.001512047 |
| MDGA2 | -5.158824064 | 4.23E-06 | 0.000161511 |
| TESC | -5.15903349 | 0.002019569 | 0.026340847 |
| SLPI | -5.181235508 | 0.002314415 | 0.029215932 |
| LYPD1 | -5.19865645 | 1.43E-11 | 2.00E-09 |
| RP11-644A7.2 | -5.20601176 | 0.008107813 | 0.07604498 |
| ADAMTS20 | -5.232105314 | 0.003136415 | 0.036975798 |
| RP5-1011O1.3 | -5.244649263 | 0.006049388 | 0.061789924 |
| CD200 | -5.256598153 | 7.78E-13 | 1.42E-10 |
| CTB-47B11.3 | -5.262152847 | 1.02E-05 | 0.000344595 |
| GNAZ | -5.289389071 | 3.33E-42 | 1.22E-38 |
| CPA4 | -5.298109353 | 4.70E-11 | 5.96E-09 |
| TFAP2A-AS1 | -5.310761411 | 0.000173689 | 0.003837249 |
| PCDH10 | -5.314523918 | 2.28E-16 | 7.68E-14 |
| MLC1 | -5.321740149 | 0.000286622 | 0.005711087 |
| TSPAN8 | -5.339057617 | 5.86E-05 | 0.001523886 |
| RP6-65G23.3 | -5.347610465 | 0.000192171 | 0.004125097 |
| SLC24A3 | -5.36539763 | 2.19E-21 | 1.30E-18 |
| MAP3K9 | -5.370011626 | 3.89E-19 | 2.03E-16 |
| SMIM1 | -5.379723356 | 7.32E-05 | 0.001844997 |
| IP6K3 | -5.390016223 | 2.03E-05 | 0.000624403 |
| HSD3B1 | -5.414070638 | 0.021617288 | 0.150138325 |

| | | | |
|---|---|---|---|
| IL21-AS1 | -5.421666101 | 0.000662317 | 0.011105562 |
| RP11-713C5.1 | -5.435856887 | 0.003093344 | 0.036512518 |
| KCNK13 | -5.515559732 | 7.33E-05 | 0.001844997 |
| CNTN1 | -5.556035238 | 1.27E-28 | 1.27E-25 |
| RTN1 | -5.557721749 | 2.20E-23 | 1.42E-20 |
| PKP2 | -5.564367688 | 0.000174968 | 0.003861612 |
| RP11-553K8.5 | -5.566297465 | 0.000774938 | 0.012505986 |
| KRT8P36 | -5.637960789 | 2.71E-07 | 1.43E-05 |
| BEST3 | -5.664467515 | 5.51E-08 | 3.49E-06 |
| PKHD1L1 | -5.666765945 | 0.001518563 | 0.021246328 |
| LINC00890 | -5.68064463 | 0.000466814 | 0.008339551 |
| FAM84B | -5.725826475 | 1.66E-14 | 4.00E-12 |
| AC073046.25 | -5.732696149 | 0.000466435 | 0.008339551 |
| OR51E2 | -5.745716297 | 0.000290171 | 0.005759749 |
| LINC01501 | -5.761794858 | 0.002839352 | 0.034187543 |
| ARAP2 | -5.769551078 | 1.99E-29 | 2.19E-26 |
| PRR9 | -5.796201718 | 0.000659087 | 0.011071251 |
| ODAM | -5.800392094 | 0.000911041 | 0.014245482 |
| ECEL1P2 | -5.82266569 | 0.000668689 | 0.011181179 |
| DKFZP434K028 | -5.834453309 | 0.001441167 | 0.020332045 |
| SAMD5 | -5.83872 | 4.90E-20 | 2.75E-17 |
| GPR143 | -5.848824684 | 1.08E-05 | 0.000362916 |
| NPFFR2 | -5.852506496 | 0.000195741 | 0.004181275 |
| NAT2 | -5.854863689 | 0.000184111 | 0.003983271 |
| EGFEM1P | -5.941954891 | 9.38E-05 | 0.002275963 |
| RP11-706O15.3 | -5.95019652 | 4.22E-06 | 0.000161247 |
| TMEM88 | -5.981415921 | 1.63E-16 | 5.59E-14 |
| HOXD13 | -6.000553864 | 0.000182581 | 0.003965804 |
| ZSWIM5P3 | -6.164765551 | 1.47E-06 | 6.38E-05 |
| CTNNA3 | -6.178531781 | 5.54E-05 | 0.001465924 |
| RP11-336N8.4 | -6.187994141 | 1.89E-05 | 0.00058819 |
| RP11-706O15.7 | -6.199822284 | 9.60E-05 | 0.002325514 |
| RP11-9G1.3 | -6.245082313 | 4.79E-07 | 2.33E-05 |
| ARSH | -6.246890629 | 0.000127875 | 0.002962327 |
| HOXD11 | -6.287519352 | 1.32E-18 | 6.14E-16 |
| SLITRK5 | -6.356298016 | 2.78E-05 | 0.000816341 |
| LIX1 | -6.514610667 | 7.75E-06 | 0.000273349 |
| ZDHHC8P1 | -6.552742415 | 1.16E-07 | 6.75E-06 |
| SHROOM2 | -6.588728916 | 3.82E-37 | 6.45E-34 |
| AC010890.1 | -6.616289665 | 5.91E-06 | 0.000215513 |
| ST6GALNAC5 | -6.653005276 | 2.55E-58 | 1.86E-54 |
| GUCY1A3 | -6.660971967 | 8.50E-05 | 0.002086926 |
| AC068490.1 | -6.670113156 | 1.79E-05 | 0.000560641 |
| SPOCK3 | -6.695818599 | 6.32E-15 | 1.61E-12 |
| KCNA4 | -6.697244815 | 5.29E-07 | 2.54E-05 |
| KCNN2 | -6.784771468 | 1.86E-11 | 2.57E-09 |

| | | | |
|---|---|---|---|
| HOXD10 | -6.820322517 | 9.46E-30 | 1.09E-26 |
| GPRC5D | -6.872187351 | 8.29E-07 | 3.84E-05 |
| NOS1AP | -6.916374065 | 2.82E-07 | 1.48E-05 |
| ACTG2 | -6.937355311 | 1.03E-17 | 4.27E-15 |
| PARM1 | -6.961210554 | 7.74E-20 | 4.25E-17 |
| KDR | -7.012464169 | 1.68E-07 | 9.41E-06 |
| NDST3 | -7.025824519 | 7.31E-08 | 4.43E-06 |
| KRT8 | -7.143325363 | 2.20E-27 | 1.86E-24 |
| FDCSP | -7.19526954 | 1.57E-06 | 6.64E-05 |
| BRINP3 | -7.327656525 | 1.13E-06 | 5.06E-05 |
| DSG3 | -7.379259955 | 5.26E-08 | 3.37E-06 |
| DPP10 | -7.401358127 | 1.86E-07 | 1.03E-05 |
| GCNT2 | -7.514114549 | 5.72E-08 | 3.60E-06 |
| CADM1 | -7.526821212 | 9.18E-46 | 4.03E-42 |
| GPR37 | -7.530337531 | 5.28E-08 | 3.38E-06 |
| CHRM3 | -7.665797163 | 1.04E-09 | 1.04E-07 |
| CPXM1 | -7.812524402 | 1.58E-05 | 0.000503812 |
| VANGL2 | -7.816788258 | 1.53E-06 | 6.53E-05 |
| TBX20 | -7.842222094 | 9.66E-17 | 3.53E-14 |
| HOXA13 | -7.968092424 | 1.68E-27 | 1.47E-24 |
| NPPB | -8.055079341 | 8.16E-16 | 2.39E-13 |
| ATP6V0A4 | -8.105874957 | 1.66E-10 | 1.94E-08 |
| CXADR | -8.131420052 | 2.42E-16 | 8.05E-14 |
| MYRF | -8.213851162 | 8.03E-74 | 1.76E-69 |
| TFAP2A | -8.222512437 | 1.93E-38 | 3.84E-35 |
| GRID2 | -8.234369377 | 3.19E-09 | 2.70E-07 |
| SLC35F3 | -8.332814599 | 2.21E-10 | 2.48E-08 |
| ALDH1A1 | -8.351334126 | 2.66E-09 | 2.29E-07 |
| SORCS1 | -8.364956993 | 0.018700974 | 0.137257268 |
| FENDRR | -8.540005224 | 4.28E-10 | 4.60E-08 |
| RP11-706O15.5 | -8.660654772 | 7.54E-13 | 1.39E-10 |
| HOTTIP | -8.83712431 | 1.29E-11 | 1.82E-09 |
| LRRTM3 | -8.872557876 | 3.12E-12 | 4.96E-10 |
| SULT1E1 | -9.438168931 | 3.09E-07 | 1.61E-05 |
| DSC2 | -9.74555595 | 1.62E-14 | 3.94E-12 |
| EEF1A2 | -10.26319677 | 2.74E-14 | 6.47E-12 |
| DSC3 | -12.31529268 | 4.19E-39 | 1.02E-35 |
