# Supplementary material for: Adipose tissue-derived human mesenchymal stromal cells can better suppress complement lysis, engraft and inhibit acute graft-versus-host disease in mice
Source: Stem Cell Res Ther. 2023 Jun 25;14:167. doi: 10.1186/s13287-023-03380-x (PMC10291819; doi:10.1186/s13287-023-03380-x)
Supplement: Supplementary file 12 — Additional file 12: Fig. S5. Blue = isotype control. Red = anti-CD59. [file 13287_2023_3380_MOESM12_ESM.pdf]

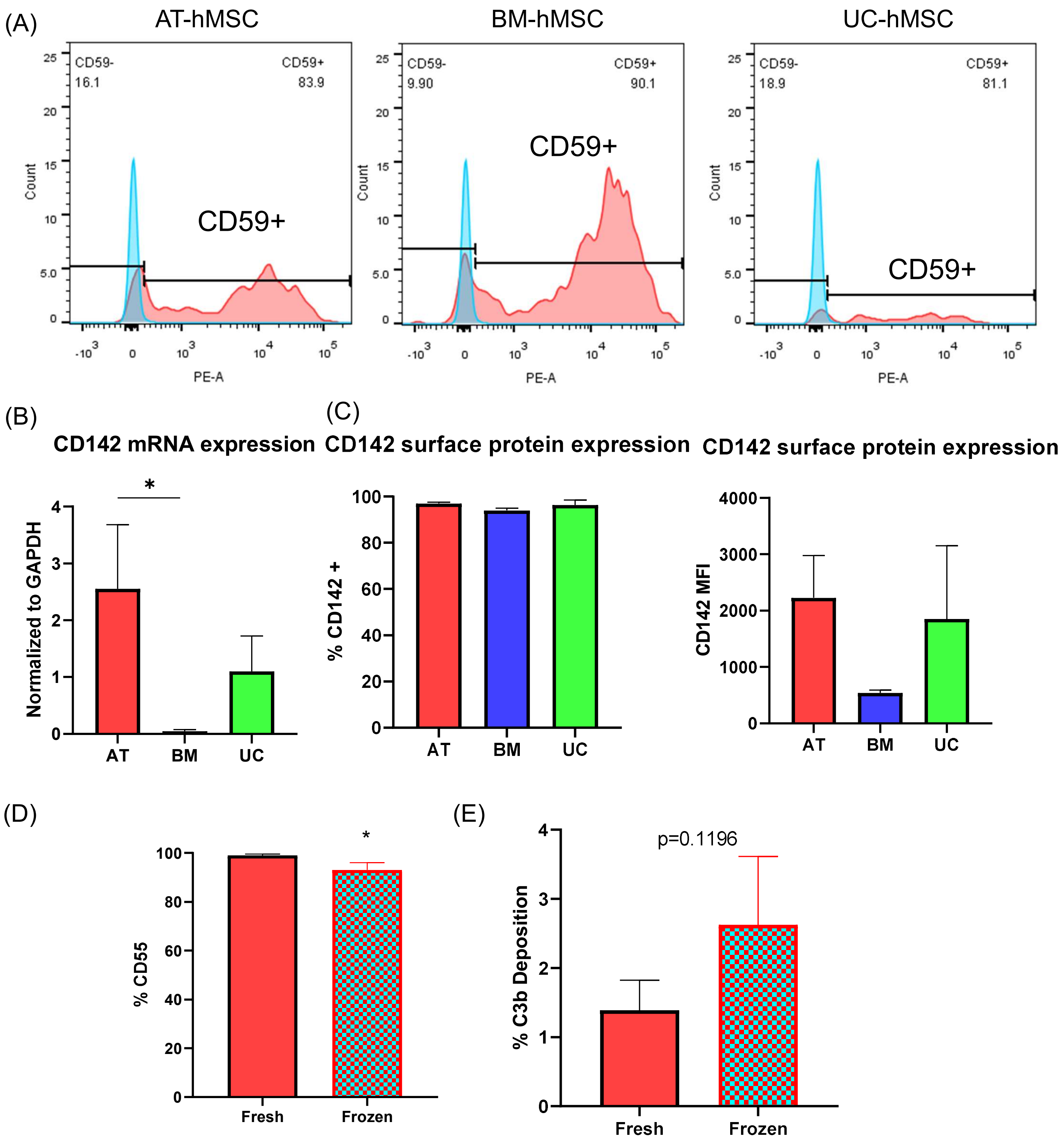

Figure S5. Analysis of complement regulators (A) Flow cytometry analysis of CD59 expression in hMSCs. Blue = isotype control. Red = anti-CD59. (B) The level of CD142 was measured by qPCR, normalized to GAPDH. (C) The surface expression of CD142 was determined by flow cytometry to show the % CD142 and the median fluorescence intensity. (D) The expression of CD55 in fresh and frozen AT-hMSCs was measured by flow cytometry. (E) C3b deposition was measured by flow cytometry after incubation with serum. N=3.
